
JOURNAL INFORMATION
==============================
NLM Title Abbreviation: Mol Cytogenet
Journal ID: Mol Cytogenet
NLM Journal ID: 101317942

Molecular Cytogenetics

EISSN: 1755-8166
Publisher: BMC

ARTICLE INFORMATION
==============================
PMCID: PMC5506601
DOI: 10.1186/s13039-017-0319-3
Article ID: 319
Article version: 1
Subjects: Meeting Abstracts

Meeting abstracts from the 11th European Cytogenetics Conference

Florence, Italy. 01–04 July 2017

Electronic publication date: 2017 Jun 29
Publication date: 2017
Volume: 10
Issue: Suppl 1
Issue sponsor: Publication of this supplement has not been supported by any sponsorship.
Electronic Location ID: 20
Copyright: © The Author(s). 2017
License: Open AccessThis article is distributed under the terms of the Creative Commons Attribution 4.0 International License (http://creativecommons.org/licenses/by/4.0/), which permits unrestricted use, distribution, and reproduction in any medium, provided you give appropriate credit to the original author(s) and the source, provide a link to the Creative Commons license, and indicate if changes were made. The Creative Commons Public Domain Dedication waiver (http://creativecommons.org/publicdomain/zero/1.0/) applies to the data made available in this article, unless otherwise stated.
License URL: https://creativecommons.org/licenses/by/4.0/

Conference name: 11th European Cytogenetics Conference
Conference Acronym: ECA 2017
Conference location: Florence, Italy
Conference date: 01-04 July 2017
issue-copyright-statement: © The Author(s) 2017

==============================
*Molecular Cytogenetics* 2017, **10**(Suppl 1):20
DOI 10.1186/s13039-017-0319-3

MEETING ABSTRACTS Open Access

### Meeting abstracts from the 11th European Cytogenetics Conference

Florence, Italy. 01–04 July 2017

Published: 29 June 2017

### Invited Lecture Abstracts

### L1
### Chromosomes to Circulating DNA

Yuk Ming Dennis Lo
The Chinese University of Hong Kong Li Ka Shing Institute of Health Sciences, Hong Kong, China
*Molecular Cytogenetics* 2017, **10(Suppl 1):**L1

In recent years, there have been an intense interest in the diagnostic applications of circulating DNA. One source of such interest is in the rapid adoption of noninvasive prenatal testing (NIPT) using cell-free fetal DNA in maternal plasma. One area of focus of my laboratory is to explore the limit of NIPT. In this regard, we have recently completed a 'second generation' noninvasive fetal genome from maternal plasma. For this work, we have sequenced the plasma DNA of a pregnant woman to a depth of 270X haploid genome coverage. This represents the deepest that a single plasma DNA sample has been sequenced to date. Using this approach, together with a novel bioinformatics pipeline, we are able to deduce, for the first time, fetal de novo mutations on a genomewide level with a sensitivity of 85% and a positive predictive value of 74%. We are also able to determine the maternal inheritance of the fetus with a 90-fold increase in resolution when compared with previous attempts. Finally, we have shown that plasma DNA molecules have preferred ending sites. Interestingly, fetal-derived and maternal-derived plasma DNA molecules have different sets of such preferred ending sites. This latter discovery has opened up many new avenues of investigation and has created new applications, e.g. for determining the fraction of fetal DNA without using genetic polymorphisms or DNA methylation markers. NIPT also serves as a model for developing noninvasive diagnostics in many other fields, e.g. oncology and transplantation.

### L2
### Genomic and functional overlap between somatic and germline chromosomal rearrangements

Wigard Kloosterman
University Medical Center Utrecht, Utrecht, The Netherlands
*Molecular Cytogenetics* 2017, **10(Suppl 1):**L2

Structural genomic variants form a common type of genetic alteration underlying human genetic disease and phenotypic variation. Despite major improvements in genome sequencing technology and data analysis, the detection of structural variants still poses challenges, particularly when variants are of high complexity. Emerging long-read single- molecule sequencing technologies provide new opportunities for detection of structural variants. We demonstrate sequencing of the genomes of two patients with congenital abnormalities using the ONT MinION at 11x and 16x mean coverage, respectively. We developed a bioinformatic pipeline - NanoSV - to efficiently map genomic structural variants (SVs) from the long-read data. Using NanoSV, we readily detected all *de novo* rearrangements involving multiple chromosomes originating from complex chromothripsis events. Genome-wide surveillance of SVs, revealed 3,253 (**33%**) novel variants that were missed in short-read data of the same sample, the majority of which are duplications < 200 bp in size. Long sequencing reads enabled efficient phasing of genetic variations, allowing the construction of genome-wide maps of phased SVs. We employed read-based phasing to show that all *de novo* chromothripsis breakpoints occurred on paternal chromosomes and we resolved the long-range structure of the chromothripsis. Our work demonstrates the value of long-read sequencing for genetic analyses in life sciences research and clinical diagnostics.

### L3
### Chromosome sequencing: the fifth and final era of cytogenetics

Malcolm Ferguson-Smith, Jorge Pereira, Fumio Kasai
Cambridge Resource Centre for Comparative Genomics, Cambridge University Department of Veterinary Medicine, Madingley Road, Cambridge CB3 0ES, UK
*Molecular Cytogenetics* 2017, **10(Suppl 1):**L3

The modern history of cytogenetics fits into five eras; chromosome spreading, chromosome banding, chromosome painting, chromosome arraying and now chromosome sequencing. T.C. Hsu, the centenary of whose birth we celebrate, was a pioneer of the first. His use of hypotonic fluid to spread chromosomes was key to the emergence of human cytogenetics and to the collection of animal karyotypes published in his Atlas of Mammalian Chromosomes edited with Kurt Benirschke. Forty-five years later, chromosome sequencing achieved the ultimate resolution by defining chromosome disease in terms of base pairs. It relies on sorting and collecting chromosomes in fluid suspension by flow cytometry and, like chromosome painting, on DNA amplification of the sorted samples. Chromosome sorting is currently the most precise method used for measuring chromosome and genome size. Ten thousand cleanly-sorted chromosomes provide sufficient DNA for Next Generation sequencing in both plants and animals. Our collaborations demonstrate the potential of this approach. For example, sequence from sorted gorilla Y chromosomes reveal, remarkably, that human and gorilla Ys are similar and that chimpanzee has lost half the Y genes present in both the other species. In two species of anole lizards, one with large heteromorphic sex chromosomes and the other with small homomorphic sex chromosomes, sequence from the ancestral Y chromosome has been fused with sequence from three microchromosomes to form a large Y chromosome. Sequencing B chromosomes in two deer species demonstrate the inclusion of different

© The Author(s). 2017 **Open Access** This article is distributed under the terms of the Creative Commons Attribution 4.0 International License (http://creativecommons.org/licenses/by/4.0/), which permits unrestricted use, distribution, and reproduction in any medium, provided you give appropriate credit to the original author(s) and the source, provide a link to the Creative Commons license, and indicate if changes were made. The Creative Commons Public Domain Dedication waiver (http://creativecommons.org/publicdomain/zero/1.0/) applies to the data made available in this article, unless otherwise stated.

regions of several autosomes associated with marked sequence variation. Sequence of a sorted 9;14 translocation chromosome in a human endometrial carcinoma not only characterized the exact breakpoint but also identified an expressed fusion gene. Our study of alpaca and other camelids has used sequence from sorted alpaca chromosomes to determine the identity of alpaca evolutionary rearrangements by comparison with the published human genome and the human:dromedary comparative map. These examples illustrate some of the possibilities of sorting for chromosome sequencing to be expected in the future.

### L4
### Comparative cytogenomics
Pat Heslop-Harrison
University of Leicester, Department of Genetics, Leicester LE1 7RH, UK
*Molecular Cytogenetics* 2017, **10(Suppl 1):**L4

TC Hsu (17 April 1917 – 9 July 2003) has been called “the father of mammalian cytogenetics”. In 1952, he developed the hypotonic solution spreading method for mammalian chromosomes following a fortunate error in making up a solution, paving the way for Tjio and Levan to report the human chromosome number as 2n = 46 in 1956. TC’s career started in China, as an insect geneticist, before settling in the US, mostly working in Texas, and moving to the exciting world of mammalian cytogenetics. He established one of the first “Frozen Zoos” with cultures of animals from A to Z (aardvark to zebra), and used numerous species to study chromosome biology and comparative evolution. At the end of his lengthy career, he was using in situ hybridization to understand the nature of non-telomeric heterochromatin and organization of chromosomes. Now, with the new methods available including high-resolution in situ hybridization, chromosome sorting, high volume sequencing and bioinformatics, we can learn about the evolution of chromosomes, comparing and contrasting diverse genotypes, species, families and even the kingdoms of plants, fungi and animals, to build a picture of key events in evolution. Many of the same changes may be seen in abnormal karyotypes and disease, normally deleterious. However, the occasional chromosomal or whole-genome changes, beyond those from mutation and recombination, can provide the novel variation leading to evolutionary success, arguably over evolutionary time giving rise to all modern lineages. The field of comparative cytogenomics is developing rapidly (see www.cytogenomics.org and www.molcyt.com) and able to show how species have evolved in the past and letting us consider paths for their evolutionary future.

### L5
### Interchromosomal core duplicons drive both evolutionary instability and disease susceptibility of the Chromosome 8p23.1 region
Mario Ventura
Department of Biology-Genetics University of Bari, Bari, Italy
*Molecular Cytogenetics* 2017, **10(Suppl 1):**L5

This abstract is not included here as it has already been published.

### L6
### L1 retrotransposons can evade somatic repression and initiate tumorigenesis in normal human colon tissues
Scott E. Devine
University of Maryland School of Medicine, Baltimore, MD 21201, USA
*Molecular Cytogenetics* 2017, **10(Suppl 1):**L6

This abstract is not included here as it has already been published.

### L7
### A fil rouge links numerical to structural chromosome abnormalities via chromothripsis
Orsetta Zuffardi, Edibe Nehir Kurtas
Department of Molecular Medicine, University of Pavia, Pavia, Italy
**Correspondence:** Orsetta Zuffardi
*Molecular Cytogenetics* 2017, **10(Suppl 1):**L7

Non-disjunction at maternal meiosis is the primary cause of spontaneous abortions as documented by extensive epidemiological studies showing trisomies in more than 50% of sporadic miscarriages.
However in a number of trisomic products of conception trisomy rescue may occur restoring the normal number of chromosomes, eventually leading to a more favorable condition for survival. We hypothesize that most constitutional supernumerary marker chromosomes (sSMC) are the relic of the supernumerary chromosome, resulting from a chromothripsis event leading to partial trisomy rescue. According to this model, chromothripsis is initiated by anaphase lagging of the supernumerary chromosome followed by its massive fragmentation within a micronucleus. The loss of some fragments and the gluing together of others might be the final outcome of the original supernumerary chromosome. To investigate the correctness of this hypothesis, we are sequencing a number of sSMCs by paired end 30x whole genome and analysing the haplotype of the sSMCs and the chromosomes from which they originate in the trios. The results in the first seven cases show that most sSMCs are formed by non-contiguous regions of the original chromosome or by contiguous ones with portions repositioned in inverted order after NHEJ, as it is expected for chromothripsis events. Moreover, though the parental origin of the sSMC resulted to be either maternal or paternal, the chromosomal portions outside the sSMC itself resulted to be biparental in the case of sSMCs of maternal origin or in hetero/isodisomy for sSMCs of paternal origin. These data demonstrate a link between numerical and structural anomalies and that the devastating effect of trisomies may not limited to prenatal life.
KEYWORDS: chromothripsis, small supernumerary marker chromosome (sSMC), whole genome paired-end sequencing, maternal meiotic non-disjunction.

### L8
### Intellectual disability in female patients with a skewed X-inactivation pattern
Fieremans N[1,2], Van Esch H[3], Holvoet M[3], Van Goethem G[4], Devriendt K[3], Rosello M[5], Mayo S[5], Martinez F[5], Jhangiani S[6], Muzny DM[6], Gibbs RA[6], Lupski JR[6,7,8,9], Vermeesch JR[3], Marynen P[1] and Froyen G[1]
[1]Human Genome Laboratory, Department of Human Genetics, KU Leuven, Belgium; [2]Human Genome Laboratory, VIB Center for the Biology of Disease, Leuven, Belgium; [3]Center for Human Genetics, University Hospitals Leuven, KU Leuven, Leuven, Belgium; [4]Department of Neurology, University Hospital of Antwerp (UZA), Antwerp, Belgium; [5]Genetics Unit, Hospital Universitario y Politecnico La Fe, Valencia, Spain; [6]Human Genome Sequencing Center, Baylor College of Medicine, Houston, Texas; [7]Department of Molecular and Human Genetics, Baylor College of Medicine, Houston, TX, USA; [8]Department of Pediatrics, Baylor College of Medicine, Houston, TX, USA; [9]Texas Children's Hospital, Houston, TX, USA
**Correspondence:** Froyen G
*Molecular Cytogenetics* 2017, **10(Suppl 1):**L8

This abstract is not included here as it has already been published.

### L9
### The CRISPR/CAS9 world
Giovanni Perini
University of Bologna, Department of Pharmacy and Biotechnology, Bologna, Italy
*Molecular Cytogenetics* 2017, **10(Suppl 1):**L9

Precise editing and regulation of genomic information is essential to understanding the function of a given gene. During the past decade, technological breakthroughs have made genome editing and regulation significantly easier. One very recent technology has adapted the CRISPR/CAS (clustered regularly interspaced short palindromic repeats /CRISPR-associated protein) bacterial immune system as a simple RNA guided platform for efficient and specific genome editing in diverse organisms, thus creating revolutionary tools for biomedical research and new possibilities for treating genetic disorders.
Here I will revise the most recent molecular approaches based on the CRISPR/CAS9 methodology to target peculiar chromosome regions or to generate specific chromosome rearrangements in mammalian cells in order to model genetic disorders and to address basic questions in chromosome biology.

### L10
### Human haploid embryonic stem cells: derivation and application in editing the human chromosomes
Nissim Benvenisty
The Azrieli Center for Stem Cells and Genetic Research, The Hebrew University, Israel
*Molecular Cytogenetics* 2017, **10(Suppl 1):**L10

This abstract is not included here as it has already been published.

### L11
### Chromatin Domains and Diseases
Stefan Mundlos
Max Planck Institute for Molecular Genetics, Berlin, Germany
*Molecular Cytogenetics* 2017, **10(Suppl 1):**L11

Deletions, duplications, inversions, translocations and insertions, collectively referred to as structural variations (SV), are thought to account for approximately 5% of genomic variability. Classical cytogenetics and array-CGH have been used as a standard diagnostic procedure to detect SVs. Besides affectng gene dosage, SVs have the potential to disrupt the integrity of the genome, causing changes in the regulatory architecture that lead to pathogenic alterations of gene expression levels and patterns. Recent findings have demonstrated that the genome is folded in a complex genetically determined manner and that this folding is directly related to gene regulation. Chromosome conformation capture is the technology that has been key to discover and investigate chromatin folding in a locus-specific (4C) or even a genome-wide (Hi-C) manner. Studies using Hi-C have shown that the genome is separated into blocks or regulatory activity called topologically associated domains (TADs).
To study the effect of structural variations we used CRISPR/Cas genome editing, to generate mice with rearrangements that are associated with limb malformations in humans. Mutant mouse limb tissue, as well as patient-derived fibroblasts, were used to generate chromosome conformation capture assays from specific view points (4C) and covering the entire locus (capture Hi-C). We show that deletions can result in the fusion of neighbouring TADs with consecutive ectopic regulation of genes. This effect depends on the presence/absence of boundary elements that separate TADs from each other (1). If the deletion does not include a boundary element, TADs will stay separated and no gene misexpression occurs. We investigated the effect of duplications at the Sox9 locus. We show that the difference in observed phenotypes can be explained by the effect of the duplications on TAD configuration. Duplications that span a TAD boundary can result in the formation of novel chromatin domains (called Neo-TAD). The formation of Neo-TADs explains the divergent effects of overlapping duplications at the SOX9 locus. Duplications that do not cross TAD boundaries (intra-TAD) do not change the overall organisation but to increased interaction with the target gene (Sox9). In contrast, duplications that cross boundaries result in the formation of new chromatin domains (called Neo-TADs) that are isolated from the rest of the genome. If genes get trapped in such a Neo-TAD, they will adopt the regulatory activity from the duplication. In the case of Cooks syndrome, this results in misexpression of Kcnj2 in a Sox9 pattern which causes the phenotype (2).
Our results demonstrate the functional importance of TADs for orchestrating gene expression via genome architecture and indicate criteria for predicting the pathogenicity of human structural variants, particularly in non-coding regions of the human genome (3).

1: Lupiáñez DG, et al. Disruptions of topological chromatin domains cause pathogenic rewiring of gene-enhancer interactions. Cell. 2015 May 21;161(5):1012–25.
2: Franke M, et al. Formation of new chromatin domains determines pathogenicity of genomic duplications. Nature. 2016 Oct 13;538(7624):265–269.
3: Lupiáñez DG, Spielmann M, Mundlos S. Breaking TADs: How Alterations of Chromatin Domains Result in Disease. Trends Genet. 2016 Apr;32(4):225–37.

### L12
### The nanoscale structure of chromatin fibers in somatic and stem cells
Maria Pia Cosma
Center for Genomic Regulation (CRG) and ICREA, Barcelona, Spain
*Molecular Cytogenetics* 2017, **10(Suppl 1):**L12

One of the interests of my group is to dissect how the Wnt pathway controls somatic cell reprogramming and pluripotency in embryonic stem cells. Recently, by using quantitative super-resolution nanoscopy, we identified a novel model of chromatin fiber assembly and the relation among the decoded structure and naïve pluripotency. We showed that nucleosomes arrange in groups of various sizes along the chromatin fiber, which we named 'nucleosome clutches' (in analogy with clutches of eggs). Clutches are interspersed with nucleosome-free regions. Interestingly, we found that ground-state pluripotent stem cells have, on average, clutches that are less dense and contain fewer nucleosomes. We are now investigating the remodeling of the chromatin fiber during the transition of cells undergoing differentiation and reprogramming and studying how the fiber is modified at nanoscale level.

### L13
### Mechanistic aspects of chromothripsis
Peter Lichter, Manasi Ratnaparkhe, Kendra Maass, Thorsten Kolb, Aurélie Ernst
Division Molecular Genetics, German Cancer Research Center (DKFZ) Heidelberg, Germany
**Correspondence:** Peter Lichter
*Molecular Cytogenetics* 2017, **10(Suppl 1):**L13

Processes causing genomic instability are known as potential drivers of tumor etiology and/or progression. Whole genome sequencing was instrumental to uncover a previously unrecognized phenomenon of genomic instability termed chromothripsis, where single chromosomes or parts thereof are shattered into tens to hundreds of fragments and the subsequent impaired repair process results in highly aberrant chromosomes. Typically, the loss of multiple fragments during this process yields in DNA copy number profiles oscillating between two or three copy numbers states. Notably, chromothripsis is associated with bad prognosis in a number of tumor entities.
Several studies revealed that chromothripsis occurs context-specific, as it takes place with high prevalence in certain tumor sub-entities that are linked to specific gene mutations or signaling pathway activations. We and others accumulated evidence for cellular processes that contribute to the formation of chromothriptic chromosomes, such as telomere attrition, poly-ploidization, micronuclei formation or DNA repair deficiency.
Recently we studied patients with the inherited tumor predisposition syndrome Ataxia Telangiectasia (AT), who are born with mutated copies of the ATM gene resulting in deficient DNA double-strand break repair and show a high frequency of acute lymphoblastic leukemia (ALL). We demonstrated that the genomic landscape of ALL of AT-patients is distinct from sporadic ALL and, most of all, tightly linked to chromothripsis. Interestingly, this event specifically involved chromosomes with acrocentric morphology, i.e. the chromosomes that constitute the nucleoli with their short arms containing clusters of r-DNA. Hypotheses as to how this could be mechanistically explained will be discussed. The study showed that compromised DNA damage response is tightly linked to chromothriptic events.
The recent status of our attempts to recapitulate the chromothripsis event in vitro and in vivo will be presented.

### L14

### Acute promyelocytic leukemia: from genetics to therapy

Francesco Lo-Coco
Department of Biomedicine and Prevention, University Tor Vergata, Rome, Italy
*Molecular Cytogenetics* 2017, **10(Suppl 1):**L14

This abstract is not included here as it has already been published.

### L15

### Circulating tumor DNA as a liquid biopsy for cancer

Heitzer, E.
Institute of Human Genetics, Medical University of Graz, Graz, Austria
*Molecular Cytogenetics* 2017, **10(Suppl 1):**L15

Tumor genomes are constantly changing, especially under the selective pressure of a given therapy. However, genomic alterations and the dynamics of tumor evolution in metastatic cancers remain incompletely characterized due to the challenge of repeated tumor sampling. The establishment of biomarkers from easily assessable biofluids like blood or plasma is beneficial compared tissue biopsy as repeated sampling is easily achievable. In recent years, cell-free circulating tumor DNA (ctDNA) has become a highly sensitive biomarker for monitoring response to treatment, the detection of minimal residual disease and recurrence, or the identification of resistance mechanisms and novel emerging targets. In contrast to tissue biopsies, the analysis of ctDNA enables a continuous and comprehensive profiling of the genetic composition of a tumor and its associated metastases.
Most of the early studies on ctDNA have employed PCR techniques and its derivatives thereof. However, in recent years several approaches for a genomewide analysis of plasma DNA were developed. While the establishment of somatic copy number alterations (SCNA) can be easily done via low-coverage WGS in a fast and cost-effective manner given a tumor fraction of more than 5-10%, the identification of mutations at the nucleotide level for large gene panels is much more expensive and time-consuming since it requires a much higher sequencing depth, in particular for the detection of underrepresented mutations. In order to investigate the clinical usefulness of ctDNA and to be able to integrate it into clinical trials, the analysis of selected cancer driver genes, which are associated with progression and treatment resistance is preferentially performed. In this talk the current state of the art of ctDNA analysis and its potential clinical applications will be discussed. Moreover, data from our ongoing studies will be presented. We have analyzed several hundred plasma DNA samples from breast, prostate, colon, and lung cancer patients, in which we could monitor tumor dynamics /evolution and treatment response.

### L16

### Ageing and leukemogenesis

George S Vassiliou
Wellcome Trust Sanger Institute, Cambridge, CB10 1SA, UK
*Molecular Cytogenetics* 2017, **10(Suppl 1):**L16

The mutational origins of clonal myeloid disorders can be traced back for years or even decades. Most of these disorders, including acute myeloid leukaemia (AML), myeloproliferative disorders (MPD), and particularly myelodysplastic syndromes (MDS) become more common with age. This age-related increase in incidence, also seen in most cancers, has been attributed to the stochastic accrual of mutations with time. This explanation is intuitive, particularly for cancers in which external mutagens do not play a major role. However, mutation accrual alone cannot account for several disease-specific observations. For example, the age-related rise in incidence of myeloid disorders is not linear and rises sharply after the 6$^{th}$ decade. Also, it is unclear why MDS become more common than AML only after the seventh decade of life.
Studies of the phenomenon of Clonal Haemopoiesis of Indeterminate Potential (CHIP) give new insights into these observations by revealing that whilst CHIP becomes commoner with age, the types of mutations driving the phenomenon differ between age groups. For example, the distribution of *DNMT3A* mutations driving CHIP changes with age, with *DNMT3A* R882H becoming less common after the 8$^{th}$ decade. Also, CHIP associated with mutations affecting spliceosome genes is rare in those younger than 60 years, but become commoner in those older than 70 years and overtakes other mutation types after the 8$^{th}$ decade. These observations suggest that the incidence and type of myeloid malignancies at different ages may be influenced by the prevailing selection pressures operating at the CHIP stage. These pressures are shaped by age-related changes that are cell-intrinsic (haemopoietic stem cell) or cell-extrinsic (haemopoietic microenvironment/niche).
Another intriguing observation is that a significant proportion of clonal haemopoiesis in those aged 60 years or older carries no identifiable mutational drivers. In fact the bulk of circulating blood cells in centenarians is often generated by a small number of stem cells. Is this due to stem cell attrition, clonal drift or selection pressures imposed by the haemopoietic microenvironment? Recent findings that CHIP is associated with adverse outcomes outside the haemopoietic compartment, such as cardiovascular disease, raise the possibility that CHIP may be a surrogate marker of organismal ageing rather than of a conventional oncogenic process. The study and early detection of these phenomena will not only help us distinguish CHIP from clonal haematological malignancies, but has the potential to also help prevent blood and other cancers and preserve healthy ageing.

### L17

### Whole Genome Duplications in Vertebrate Evolution

V.A. Trifonov[1,2], A.I. Makunin[1], S.A. Romanenko[1,2], L.S. Biltueva[1], V.R. Beklemisheva[1], M.A. Pobedintseva[1,2], D.Yu. Prokopov[1,2], D. A. Andreyushkova[1], A.S. Graphodatsky[1,2]
[1]Institute of Molecular and Cellular Biology SB RAS, Novosibirsk, Russia;
[2]Novosibirsk State University, Russia
**Correspondence:** V.A. Trifonov (vlad@mcb.nsc.ru)
*Molecular Cytogenetics* 2017, **10(Suppl 1):**L17

Almost 50 years ago S. Ohno has suggested that vertebrate ancestors have undergone two whole genome duplication events (WGD). Since then a large body of evidence (especially from to genome sequencing projects) has been accumulated that further supported the theory and considerably broadened our understanding of genome evolution. It was suggested that WGDs might have long-termed evolutionary advantages by increasing functional genomic complexity.

Besides, it was demonstrated that some phylogenetic lineages have become highly sensitive to whole genome copy changes (mammals), while others are characterized by an increased propensity to polyploidization (amphibians and fish). Here we compare the distribution of WGD events across studied vertebrate taxa and argue that the most WGD-rich taxon is Acipenseriformes, where five whole genome polyploidy events occurred, and the process is still ongoing. We hypothesize that this might be connected to relaxed pachytene check point in this group and that this group represents a convenient model for studying the role of polyploidy in vertebrates.
Financial support: RSF grants No. 14-14-275 and 16-14-10009.

### L18

### Reconstruction of ancestral karyotypes and identification of chromosome evolution

Martin A. Lysak
CEITEC – Central European Institute of Technology, Masaryk University, Brno 625 00, Czech Republic
*Molecular Cytogenetics* 2017, **10(Suppl 1):**L18

The ever increasing amount of paleogenomic data along with improved large-scale phylogenies are allowing us to reconstruct a tentative structure of ancestral genomes, infer ancestral chromosome numbers, and to determine the directionality of chromosomal and karyotype evolution in land plants. Genome and transcriptome sequencing revealed a wealth of family- and lineage-specific whole-genome duplication (WGD) events, which had a major impact on plant genome complexity as well as on the mode and tempo of karyotype evolution. Crucifers (Brassicaceae) and grasses (Poaceae) represent two families of land plants with the most detailed understanding of karyotype evolution. Comparative cytomolecular maps along with the whole-genome sequence data available for over 50 crucifer species allowed us to reconstruct ancestral genomes and infer prevalent trends of genome evolution in crucifers (320 genera, over 3,600 species). The genome evolution in the whole family was shaped by a shared WGD called the Alpha-WGD. Following the initial re-diploidization of the polyploid ancestral genome, several quasi-diploid genomes evolved together with the diversification of main crucifer lineages. The ancestral genomes remained conserved in a number of extant species, whereas in other groups they were reshuffled by inversions and translocations with or without descending dysploidy. Genomes of crucifer plants were also shaped by several younger clade- and genus-specific WGDs. In taxa which experienced recent WGDs, the extent and tempo of genome reshuffling towards re-diploidized genomes exceed that observed in „ old quasi-diploids". Cyclic rounds of polyploidization and re-diploidization seem to be important drivers of genetic, physiological and ecological diversification, and ultimately of speciation. By contrast, some important clade diversifications and numerous speciation events were not associated with major chromosome rearrangements.

### L19

### The untimely separation of chromosomes and kinetochores in human oocytes may explain the maternal age effect

Agata Zielinska
Max Planck Institute of Biophysical Chemistry, Meiosis, Goettingen, Germany
*Molecular Cytogenetics* 2017, **10(Suppl 1):**L19

Aneuploidy in human eggs is the leading cause of pregnancy loss and Down's syndrome. Aneuploidy results from chromosome segregation errors that occur during the development of an egg from a progenitor cell called an oocyte. For unknown reasons, human eggs frequently contain the wrong number of chromosomes. Moreover, this high rate of aneuploidy increases dramatically with advancing maternal age. Here, we present novel insights into the architecture of chromosomes in human eggs that may predispose human oocytes to segregation errors. High resolution imaging of over 2,500 sister kinetochores revealed that the majority of chromosomes in human oocytes feature altered kinetochore geometry in contrast to oocytes of other mammalian species. Surprisingly, we found that sister kinetochores in human oocytes were frequently not fused and behaved as separate functional units during the first meiotic division. Interestingly, kinetochores in oocytes from older women were more likely to have separated kinetochores. Importantly, this altered kinetochore configuration allowed the chromosomes to adopt non-conventional orientations on the spindle and promoted erroneous attachments to the spindle. We further found that cohesion between chromosome arms was weakened. Consistently, the fraction of bivalents that precociously dissociated into univalents increased in an age-dependent manner. Together, our data reveal multiple age-related changes in chromosome architecture that could explain why aneuploidy in human oocytes increases with advanced maternal age.

### L20

### Epimutations as a novel cause of congenital disorders

Mafalda Barbosa[1,2], Ricky Joshi[1], Paras Garg[1], Nihir Patel[1], William Gibson[1], Corey Watson[1], Alejandro Martin[1], Fatima Lopes[3], Lisenka Vissers[4], Silvia de Rubeis[2], Jennifer Reichert[2], Patricia Maciel[3], Tjitske Kleefstra[4], Han G. Brunner[4], Joseph D. Buxbaum[2], Bruce Gelb[1], Andrew J. Sharp[1]
[1]Department of Genetics and Genomic Sciences, The Mindich Child Health & Development Institute, Icahn School of Medicine at Mount Sinai, NY, USA; [2]The Seaver Autism Center for Research and Treatment, Icahn School of Medicine at Mount Sinai, NY, USA; [3]Life and Health Sciences Research Institute, School of Health Sciences, University of Minho; ICVS/3B's PT Government Associate Laboratory, Braga/Guimarães, Portugal; [4]Radboud University Medical Center, Department of Human Genetics; Center for Molecular Life Sciences, Nijmegen, The Netherlands
**Correspondence:** Mafalda Barbosa
*Molecular Cytogenetics* 2017, **10(Suppl 1):**L20

The application of genome sequencing in patients with neurodevelopmental disorders and/or multiple congenital anomalies (ND/CA) can typically identify pathogenic mutations in less than 50% of the patients. This fact suggests that some patients harbor genomic lesions that are undetectable by conventional sequencing technologies. We hypothesized that some cases of ND/CA are instead caused by epigenetic aberrations that lead to a deregulation of normal genome function.
We have performed DNA methylation profiling in 500 individuals with ND/CA, who had all been previously tested by microarray, exome- and/or whole genome sequencing and were found to be negative for disease-causing mutations. We screened patient methylation profiles for large epigenetic changes covering clusters of CpGs (termed epimutations) that were absent from a population of 1,500 normal controls, identifying 173 epimutations in 118 of the cases tested (23%). Targeted validations showed an ~90% true positive rate, and revealed that these epimutations represent large methylation changes specifically on one allele. Testing of parental samples of patients with ND/CA showed that 47% of these epimutations were de novo events, which in comparison to the rate observed in unaffected control pedigrees was 3.6-fold higher ($p = 0.01$).
We identified seven recurrent epimutations in our disease cohort, two of which (FMR1, MEG3) have prior known disease associations, validating our method for detecting pathogenic epimutations. In two patients with congenital heart defects we identified a recurrent hypo-methylation defect of MOV10L1, a gene with an embryonic heart-specific isoform that interacts with the master cardiac transcription factor NKX2.5. Finally we also identified loss of methylation defects at two known imprinted loci that have no prior disease associations (NAA60/ZNF597and L3MBTL1), suggesting these as potentially novel imprinting disorders.
To expand on these findings, we have performed a variety of large-scale sequencing, expression and population studies, providing insights into the causes, functional consequences and biology of epimutations. We find that epimutations are frequently associated with extreme outlier and mono-allelic gene expression, with an impact comparable to loss-of-function mutations, and in some cases occur secondary to cis-linked regulatory mutations, providing a rationale for interpreting non-coding genetic variants.
Overall, our study indicates that methylation profiling likely has a diagnostic yield comparable to that of CNV screening by microarray, with 10% of the patients who were refractory to conventional mutation screening approaches presenting pathogenic epigenetic defects. We propose that epigenome profiling represents a promising method for the study of human disease that complements sequence-based approaches.

### L21

### Transgenerational epigenetic effects

Yuri Dubrova
University of Leicester Department of Genetics Leicester, UK
*Molecular Cytogenetics* 2017, **10(Suppl 1):**L21

This abstract is not included here as it has already been published.

### L22

### Ten years external quality assessment in leukemia cytogenetics in Germany – what did we learn?

Harald Rieder
Institute for Human Genetics, University Clinic Duesseldorf, Germany
*Molecular Cytogenetics* 2017, **10(Suppl 1):**L22

External quality assessment (EQA) is an integral part of quality control in diagnostic laboratories. An interlaboratory test (IT) system using viable cells was established for EQA in leukemia cytogenetics in Germany in 2004. The test was based on surrogate leukemia samples which was generated by using an admixture of a leukemia cell lines with an abnormal karyotype showing target chromosome aberrations, a lymphoblastoid cell line with a normal karyotype, and peripheral blood of a healthy donor. The laboratories were requested to perform a chromosome banding analysis of the surrogate sample following routine operating procedures and to send a written report including a critical evaluation of the cytogenetic findings to the IT center. This approach covered the entire process of chromosome banding analysis including the cultivation of the cells, chromosome preparation, banding and analysis as well interpretation of the findings. The reports were evaluated by a central review committee with respect to the detection of the target chromosome aberrations which were present in the sample, and to the correctness of the karyotype according to the International System for Human Cytogenomic Nomenclature (ISCN). The appraisal of the cytogenetic changes was also included in the assessment. Twelve interlaboratory tests using cell lines of acute myeloid (n = 4), acute T- or B-cell lymphoblastic (n = 5), Burkitt´s type acute lymphoblastic (n = 2), and chronic myeloid leukemia (n = 1) have been performed. The formal requirements for cytogenetic reports were met by almost all laboratories. The abnormal clone was detected on average by 91.5% (range 70-100% per IT) of the participating laboratories. Formal errors of the karyotypes were present in 58% (range 22-76% per IT) of the reports and were most frequent in ITs with cell lines with complex karyotypes. The introduction of inadequate spaces in the karyotype was most frequent, which was most probably done to improve the legibility of the karyotypes. However, karyotype errors which distorted the observed cytogenetic changes were also frequent and encompassed missing or incorrect chromosome band designation, single cell aberrations, and improper descriptions of the chromosome aberrations. The appraisal of the findings was appropriate in 83% of the IT. Overall, over the years there was an improvement of the analytical performance as well as of the quality of the cytogenetic reports. However, further efforts should be undertaken to increase the detection rate of chromosome abnormalities and to ameliorate the description of the karyotypes according to the ISCN.

### L23

### External quality assessment in constitutionnal array-CGH and consequences for routine practice, the French experience

Martine Doco-Fenzy[1,2,3], Jean-Michel Dupont[1,2,4], Isabelle Luquet[1,5], Christine Terre[1,6], Cyril Sarrauste de Menthières[1], Marie-Christine Combrisson[1], Damien Sanlaville[1,2,7]
[1]Association des Cytogénéticiens de Langue Française (ACLF), France; [2]Achro-puces network, France; [3]Service de Génétique CHU Reims, HMB, UFR de médecine, EA 3801, REIMS, France; [4]Service de Génétique CHU Cochin, Paris, France; [5]Service de Cytogénétique CHU Toulouse, France; [6]Service de Cytogénétique CHU Versailles, France; [7]Service de Génétique CHU Lyon, France
**Correspondence:** Martine Doco-Fenzy
*Molecular Cytogenetics* 2017, **10(Suppl 1):**L23

The Association des Cytogénéticiens de Langue Française (ACLF) provides external quality assessment (EQA) schemes in cytogenetics for the French speaking laboratories since 2005. The constitutional assessment in pre and postnatal field was first retrospective, based on the reviewing of metaphase pictures captured by each laboratories. After 2007 the EQA became prospective based on the uploading of the same pictures by all participants from the website http://www.eaclf.org.
In 2010 the ACLF EQA pilot committee composed of 9 ACLF members proposed to set up a pilot study for array-CGH. The pilot was a postnatal case. The issue of this Array EQA was to propose an assessment of analytical and post-analytical process for the laboratories. Indeed this EQA was based on the analysis of the same DNA sended by the scheme organizer to the participating laboratories. The laboratories registered, then received the DNA, made the analysis and answered on the website by filling a form. They had to load their report established the same way as any report in their laboratory in routine practice. Since then about 30 laboratories registered each year to this EQA. An overview of the reports and participation is organized each year ending with a meeting where the feedback of participating laboratories is collected. At this occasion the marking and assessing criteria based on the reference of national guidelines are discussed. The edition of the guidelines is managed by the quality committee of the French Achro-puces network (Prof D Sanlaville). The guidelines for DNA microarrays and array-CGH have been written intending to homogenize the pre- to post-analytical process and particularly the interpretation phase. The improvement of the guidelines were motivated notably because of the round trip process between EQA assessors and participants. Long discussion happen between assessors about the interpretation of Copy Number Variations. The process of upgrading guidelines is not easy, they have to be regularly revised according to the national and international background and they impact the routine practice. Both assessors and participants have not always the sufficient distance to propose consensual modifications because of the rapid evolution of the arrays technologies. We will give examples of discussions most often happening during EQA assessment and consequently the impact on the routine DNA array practice and reports.

### L24

### Noninvasive prenatal screening or advanced diagnostic testing: caveat emptor

Mark I Evans
Mt. Sinai School of Medicine, Obstetrics & Gynecology, New York, USA
*Molecular Cytogenetics* 2017, **10(Suppl 1):**L24

The past few years have witnessed extraordinary advances in prenatal genetic practice led by 2 major technologic advances; next gen sequencing of cell free DNA and microarray analysis of CVS and amniotic fluid samples resulting in increased cytogenetic resolution. Non-invasive prenatal screening (NIPS) has evolved from suboptimal techniques for fetal cells in maternal blood to cell free fetal DNA and RNA to finally, with next generation sequencing (and also targeted probes and targeted sequencing), to have high performance sensitivity and specificity for Trisomy 21 (DS). There is lesser performance for other common aneuploidies and now also several microdeletion disorders. These tests were launched with relatively small studies and few publications. Very aggressive marketing has captured increasing market share. Most large cytogenetic labs and clinical programs have noted significant decreases in patients receiving counseling and undergoing diagnostic procedures. Many patients and doctors regard NIPS as a “replacement” for CVS or amniocentesis – a conclusion which is not supported by the evidence.
In patients with an ultrasound abnormality and a normal karyotype, the abnormal CNV rate is 6-8%. For patients with normal history, ultrasound, and karyotype, the abnormal CNV is approximately 1% - comparable to classic chromosomal risk for a 38 year old. At age 30 year, the chance that aCGH finds a clinically serious CNV is literally 10 X that NIPS finds DS.
As such, we offer every patient, regardless of their age, the opportunity to have diagnostic procedures (CVS or amnio) and aCGH to investigate for CNVs. The risks of diagnostic procedures are equally safe, and in experienced hands < 1/500.
There is never “no risk.” The question is where does the patient want to put that risk, i.e. risk of having a child with a serious problem vs risk of a complication to find out?
NIPS is very sensitive for DS, but very expensive for what it does. In some areas, the cost approaches the total reimbursement for 9 months of pregnancy care. The cost to find a DS case that would have been missed by free β, PAPP-A, and NT is > $3 Million. With expansion to other disorders, performance drops dramatically.
We believe patients must be counselled far more completely than common practice. For those patients who want the “most thorough” evaluation, offering CVS or amnio with aCGH and high quality ultrasound will maximize identification of problems and give patients more complete knowledge of the health of their fetus.

### L25

### Performance evaluation and first clinical application of a new paired-end MPSS approach for cfDNA screening of common aneuploidies

Cirigliano V., Ordoñez E., Rueda L., Nicolas S., Castilla I., Grau M., Puertollano C., Cañadas MP
Department of Molecular Genetics and Cytogenetics. Labco Diagnostics-Synlab Group. 08950, Esplugues de Llobregat, Barcelona, Spain
**Correspondence:** Cirigliano V
*Molecular Cytogenetics* 2017, **10(Suppl 1):**L25

**Background**
Paired-end MPSS allows digital counting of plasma cfDNA while also measuring each fragment length by sequencing it’s both extremities. As fetal cfDNA is known to be shorter, size differences can be used to determine fetal fraction (FF). Also, in case of fetal aneuploidy, counting differences can appear higher if confirmed on shorter fragments only.
NeoBona is the first paired-end MPSS screening test providing this double check of chromosome counting statistics through a novel bioinformatics approach.
We evaluated its performance on samples with known outcome and report its first clinical application on a large cohort of average risk pregnancies.
**Methods**
Retrospective blind study of 1730 plasmas from 1st trimester pregnancies, including 66 confirmed T21, 34 T18, 13 T13 and 8 sex chromosomes aneuploidies.
Samples were screened using NeoBona test to determine the likelihood of aneuploidy (Tscore) based on FF, counting statistics and cfDNA size distribution. Cut-offs were applied at Tscores to classify normal and aneuploid cases.
The test was then used on 19151 consecutive pregnancies (575 twins), regardless risk category, XY aneuploidy screening was requested in 57% of cases. Samples were reported within 5 days from collection.
**Results**
Results were obtained in 98,9% archived plasmas. All T21 and T13 were detected (100% sensitivity and specificity) as also 33/34 T18. All 5 cases 45,X and 3 47,XXY were identified without false positive results. Samples at low FF were correctly scored, in some cases even below 1%.
The NeoBona test provided results in 99.2% clinical cases, in 334 after redraw (1.7%). A total of 288 T21, 63 T18 and 27 T13 were reported, in 23 cases with FF between 1 and 3%. Invasive procedures were performed to confirm results in 99% of cases. Five false positive results were observed for T21, 2 T18 and 3 for T13 (FPR 0.03%, 0.01% and 0.02%). One T21 was missed, 7338 pregnancies still ongoing. XY aneuploidies were reported in 39 cases. Vanishing twins were suspected in 3 cases and no XY result reported, 2 maternal aneuploidies were also identified. Follow-up was available for 11 cases with 4 FP results (FPR 0.13%).
**Conclusions**
Paired-end MPSS and the novel bioinformatics approach of NeoBona allowed detecting aneuploidies with confidence even at fetal fractions below 1% with reduced FPR. Removing the need of a lower limit of FF allowed cfDNA analysis to be successful on a high proportion of clinical cases, thus extending the benefits of cfDNA screening to a larger population of pregnancies.

### L26

### Should preimplantation genetic screening (PGS) be implemented to routine IVF practice?

Raoul Orvieto
Infertility and IVF Unit, Department of Obstetrics and Gynecology, Sheba Medical Center (Tel Hashomer), Sackler Faculty of Medicine, Tel Aviv University, Tel Aviv, Israel
*Molecular Cytogenetics* 2017, **10(Suppl 1):**L26

This abstract is not included here as it has already been published.

### L27

### Duplication, Structure and the Evolution of the Human Genome

Evan E. Eichler
Department of Genome Sciences and Howard Hughes Medical Institute, University of Washington, Seattle, WA 98195, USA
*Molecular Cytogenetics* 2017, **10(Suppl 1):**L27

Human duplicated sequences show extraordinary sequence complexity and are important sources for gene innovation and rearrangement associated with neurocognitive and neurodevelopmental diseases. I will present an overview of the evolution of great ape segmental duplications and their potential to generate neofunctional paralogs through segmental duplication fusion and truncation. I will highlight examples of novel genes that have evolved specifically within the human lineage where functional data suggest they have contributed to unique neuroadaptive aspects of humans, including an increased density of excitatory/inhibitory synapses and the expansion of the frontal cortex. Using single-molecule, real-time (SMRT) sequencing technology, I will show how such complex regions can now be resolved and demonstrate how radically these regions have changed even between closely related species such as chimpanzee and human leading to complex inversion and the unprecedented shuffling of gene order. Paradoxically, this duplication architecture complexity has led to a high background rate of copy number variation mutations associated with neurodevelopmental disease (e.g., autism, intellectual disability and epilepsy) in the human species suggesting that novel adaptations and increased disease burden are inextricably linked.

### Oral Abstracts

### O1

### Unravelling structural chromosomal rearrangements by whole genome sequencing results of the ANI project a French collaborative study including 55 patients with intellectual disability and or congenital malformations

Caroline Schluth-Bolard [1], Flavie Diguet [1], Pierre-Antoine Rollat-Farnier [2], Alexandra Afenjar [3], Florence Amblard [4], Jeanne Amiel [5], Joris Andrieux [6], Marc-antoine Belaud-Rotureau [7], Brigitte Benzacken [8], Sophie Blesson [9], Marie-Noëlle Bonnet-Dupeyron [10], Patrick Callier [11], Yline Capri [12], Nicolas Chatron [1], Emilie Chopin [13], Patrick Collignon [14], Marie-Pierre Cordier [2], Bénédicte Demeer [15], Annabelle Chaussenot [16], Florence Demurger [17], Françoise Devillard [4], Martine Doco-Fenzy [18], Céline Dupont [12], Jean-Michel Dupont [19], Sophie Dupuis-Girod [2], Laurence Faivre [20], Brigitte Gilbert-Dussardier [21], Anne-Marie Guerrot [22], Marie Houlier [5], Bertrand Isidor [23], Sylvie Jaillard [7], Eric Jeandidier [24], Boris Keren [25], Valérie Kremer [26], Didier Lacombe [27], Cédric Le Caignec [28], Aziza Lebbar [19], Marine Lebrun [29], Gaétan Lesca [1], James Lespinasse [30], Valérie Malan [31], Michèle Mathieu-Dramard [15], Julie Masson [1], Alice Masurel-Paulet [20], Cyril Mignot [3], Chantal Missirian [32], Anne Moncla [32], Fanny Morice-Picard [27], Sébastien Moutton [27], Gwenaël Nadeau [30], Céline Pebrel-Richard [33], Sylvie Odent [17], Véronique Paquis [16], Laurent Pasquier [17], Nicole Philip [34], Morgane Plutino [16], Linda Pons [1], Marie-France Portnoï [3], Fabienne Prieur [29], Jacques Puechberty [35], Audrey Putoux [1], Marlène Rio [5], Caroline Rooryck-thambo [27], Massimiliano Rossi [1], Isabelle Rouvet [13], Catherine Sarret [33], Véronique Satre [4], Jean-pierre Siffroi [3], Anne-Claude Tabet [12], Marianne Till [2], Renaud Touraine [29], Annick Toutain [9], Stéphanie Valence [3], Michel Vekemans [31], Alain Verloes [12], Sandra Whalen [3], Patrick Edery [1], Damien Sanlaville [1]

[1]Centre De Recherche En Neurosciences De Lyon, Inserm U1028; Cnrs Umr5292; Ucbl1; Gendev Team, Service De Génétique, Hospices Civils De Lyon, Bron, France; [2]Hospices Civils De Lyon, Service De Génétique, Bron, France; [3]Hôpital D'enfants Armand Trousseau, Département De Génétique Médicale, Paris, France; [4]Chu Grenoble, Laboratoire De Génétique Chromosomique, Hôpital Couple Enfant, Grenoble, France; [5]Hôpital Necker-Enfants Malades, Service De Génétique Médicale, Paris, France; [6]Chru Lille, Institut De Génétique Médicale, Hôpital Jeanne De Flandre, Lille, France; [7]Chu Pontchaillou, Laboratoire De Cytogénétique Et De Biologie Cellulaire, Rennes, France; [8]Hôpital Jean Verdier, Laboratoire De Cytogénétique, Bondy, France; [9]Chru Tours, Service De Génétique, Tours, France; [10]Ch Valence, Service De Cytogénétique, Valence, France; [11]Chu Dijon, Laboratoire De Cytogénétique, Dijon, France; [12]Hôpital Robert Debré, Département De Génétique, Paris, France; [13]Hospices Civils De Lyon, Centre De Biotechnologie Cellulaire, Bron, France; [14]Chi Toulon, Service De Génétique Médicale, Toulon, France; [15]Chu Amiens, Centre D'activité De Génétique Clinique, Pôle Pédiatrie, Hôpital Nord, Amiens, France; [16]Chu Nice, Service De Génétique Médicale, Nice, France; [17]Chu Rennes, Service De Génétique Clinique, Rennes, France; [18]Chu Reims, Service De Génétique, Reims, France; [19]Hôpital Cochin, Laboratoire De Cytogénétique Constitutionnelle, Paris, France; [20]Chu Dijon, Centre De Référence Anomalies Du Développement Et Syndromes Malformatifs, Dijon, France; [21]Ea 3808 Université De Poitiers, Génétique Médicale, Chu La Milétrie, Poitiers, France; [22]Chu Rouen, Unité De Génétique Clinique, Rouen, France; [23]Chu Nantes, Service De Génétique Médicale, Unité De Génétique Clinique, Nantes, France; [24]Hôpital Emile Muller, Service De Génétique, Mulhouse, France; [25]Gh Pitié-Salpêtrière, Département De Génétique, Paris, France; [26]Chu Strasbourg, Laboratoire De Cytogénétique, Strasbourg, France; [27]Chu Bordeaux, Service De Génétique Médicale, Bordeaux, France; [28]Chu Nantes, Service De Génétique Médicale, Institut De Biologie, Nantes, France; [29]Chu Hôpital Nord, Service De Génétique Clinique, Chromosomique Et Moléculaire, Saint-etienne, France; [30]Ch Général, Laboratoire De Génétique Chromosomique, Chambéry, France; [31]Hôpital Necker Enfants Malades, Service De Cytogénétique, Paris, France; [32] Ap-Hm, Département De Génétique Médicale, Laboratoire De Génétique Chromosomique, Marseille, France; [33] Chu Estaing, Service De Cytogénétique Médicale, Clermont-ferrand, France; [34] Ap-Hm, Département De Génétique Médicale, Unité De Génétique Clinique, Marseille, France; [35]Hôpital Arnaud De Villeneuve, Service De Génétique, Montpellier, France

**Correspondence:** Caroline Schluth-Bolard

*Molecular Cytogenetics* 2017, **10(Suppl 1):**O1

#### Introduction

Apparently balanced chromosomal rearrangements (ABCR) associated with abnormal phenotype are rare events, but may be challenging for genetic counseling. Abnormal phenotype may be explained either by cryptic genomic imbalances detectable by array-CGH or by gene disruption or position effect. However, breakpoint cloning using conventional methods is laborious and not performed routinely. Recently, Whole Genome Sequencing (WGS) proved to be a powerful and rapid technique to characterize ABCR breakpoints at the molecular level.

#### Methods

The ANI project is a French collaborative study that aims at characterizing ABCR in patients presenting with intellectual disability and/or congenital anomalies. We included 55 patients (41 reciprocal translocations, 4 inversions, 2 insertions, 8 complex chromosomal rearrangements). Array-CGH showed no pathogenic imbalance. Breakpoints were characterized by paired-end WGS and confirmed by Sanger sequencing. Expression studies of disrupted and neighboring genes were performed on blood cells.

#### Results

At this time, 45 patients were analyzed by WGS. Breakpoints were characterized for 39 patients (86%). The rearrangements showed unexpected complexity, since 186 breakpoints were identified against 97 breakpoints according to karyotype and included 6 cases of chromoanagenesis. Sixty-seven breakpoints disrupted a gene (36%). In 14/39 patients, WGS allowed a diagnosis, either by gene disruption (11) or by position effect (3), thus a diagnostic rate of 35%.

#### Conclusion

These preliminary results showed the diagnostic relevance of this approach. We will present the complete results of this study, including molecular characteristics of the breakpoints and expression studies.

This work is supported by ANR and DGOS (PRTS 2013 grants).

### O2

### Chromothripsis as a mechanism driving genomic instability mediating brain diseases

Ivan Iourov [1], Svetlana Vorsanova [2], Thomas Liehr [3], Maria Zelenova [1], Oxana Kurinnaia [4], Kirill Vasin [1], Yuri Yurov [1]

[1]Mental Health Research Center, Moscow, Russia; [2]Russian National Research Medical University Named After N.i. Pirogov, Separated Structural Unit "clinical Research Institute of Pediatrics, Molecular Cytogenetics Of Neuropsychiatric Diseases, Moscow, Russia; [3]Institute of Human Genetics, Fsu, Molecular Cytogenetics, Jena, Germany; [4]Moscow State University of Psychology And Education, Molecular Brain Genetics, Moscow, Russia

**Correspondence:** Ivan Iourov

*Molecular Cytogenetics* 2017, **10(Suppl 1):**O2

Recently, it has been shown that brain diseases (Alzheimer's disease, autism) are likely to be mediated by genomic instability (GIN) affecting the brain. However, there is still no consensus on extents of the contribution and molecular mechanisms, by which GIN occurs. Using SNP-array molecular karyotyping (resolution >1000 bp), FISH and interphase multicolor banding (ICS-MCB), we have addressed genomic variations in 502 children with autism and intellectual disability (ID) as well as in post-mortem brain samples of autism and Alzheimer's disease patients (12 and 10 samples, respectively). We have found that 7 (1.4%) out of 502 children with autism/ID demonstrate chromothripsis, hallmarked by oscillating copy number variations, random joins and heterozygosity retention. These patients also exhibited other GIN types (mosaic aneuploidy and non-specific gross structural chromosome rearrangements). Almost exactly the same patterns of chromothripsis and other GIN types were detected in 3 autistic brain samples. The Alzheimer's disease brain exhibited chromothripsis in 2 samples. Interestingly, that chromothripsis was detected in brain samples hallmarked by high levels of somatic mosaicism for aneuploidy and acentric chromosomal fragments. Molecular cytogenetic analyses of 25 control brain samples have not revealed chromothripsis. According to our findings, chromothripsis seems to be a relatively common mechanism for GIN associated with brain diseases. Additionally, chromothripsis is demonstrated for the first time to affect the diseased brain without cancerization. Accordingly, one may suggest that molecular alterations to genome stability maintenance machinery resulting in tissue-specific GIN (or chromosome instability) could be an underappreciated cause of a wide spectrum of complex diseases as it is the case for a number of cases of common brain disorders.

Supported by the Russian Science Foundation (Grant #14-35-00060); studying the adult brain is supported by ERA.Net RUS Plus Programme.

### O3

### Whole genome characterization of array defined clustered CNVs reveals two distinct complex rearrangement subclasses generated through either non homologous repair or template switching

Jesper Eisfeldt [1], Lusine Nazaryan-Petersen [2], Johanna Lundin Lundin [1], Maria Pettersson [1], Daniel Nilsson [1], Josephine Wincent [1], Agne Lieden [1], Francesco Vezzi [3], Valteri Wirta [4], Max Käller [4], Tina Duelund [5], Rayan Houssari [5], Laura Pignata [5], Mads Bak [2], Niels Tommerup [2], Elisabeth Syk Lundberg [1], Zeynep Tümer [5], Anna Lindstrand [1]

[1]Karolinska Institutet, Department of Molecular Medicine And Surgery, Stockholm, Sweden University of Copenhagen, Wilhelm Johannsen Center For Functional Genome Research, Institute of; [2]Cellular And Molecular Medicine, Copenhagen, Denmark; [3]Stockholm University, Scilifelab, Department of Biochemistry And Biophysics, Stockholm, Sweden; [4]Kth Royal Institute of Technology, Scilifelab, School of Biotechnology, Stockholm, Sweden; [5]Copenhagen University Hospital, Kennedy Center, Department of Clinical Genetics, Copenhagen, Denmark

**Correspondence:** Anna Lindstrand

*Molecular Cytogenetics* 2017, **10(Suppl 1):**O3

#### Introduction

Clustered copy number variants (CNVs) as detected by chromosomal microarray are often reported as germline chromothripsis. However, such cases might need further investigations by massive parallel whole genome sequencing (WGS) in order to properly define the underlying complex rearrangement.

#### Methods

22 carriers of clustered CNVs, previously referred to the Departments of Clinical Genetics at the Karolinska University Hospital (Stockholm, Sweden) or Kennedy Center, Rigshospitalet (Copenhagen, Denmark) for a clinical chromosome microarray due to congenital developmental disorders, were sequenced using either Paired-End or Mate Pair libraries. To utilize the WGS data for structural variant analysis, a WGS caller (TIDDIT), a pipeline (FindSV) and a visualization program to picture the rearrangement end-product were developed.

#### Results

By combining read depth and discordant read pair analysis 154 junctions were characterized (range 4–26; median = 5) and an overall connectivity picture is given in 21 cases. These rearrangements were sub-classified depending on the patterns observed:

(1) Cases with clustered deletions only (e.g. del-nml-del-nml-del) often had additional hidden structural rearrangements, such as insertions and inversions, that may be the result of multiple simultaneous double-strand DNA breaks followed by non-homologous repair typical to chromothripsis.

(2) Cases with only duplications (e.g. dup-nml-dup) or combinations of deletions and duplications (e.g. del-nml-dup-del-nml-dup-nml-del), demonstrated a pattern of inversions, deletions and duplications more consistent with serial template switching during DNA replication (chromoanasynthesis).

#### Conclusion

Multiple copy number changes clustered on a single chromosome may arise through both chromothripsis and chromoanasynthesis.

### O4

### Defining haplotypes of complex structural variation using multicolour fibre FISH amylase CNV study

Sandra Louzada [1], Nzar A.a. Shwan [2], Beiyan Fu [1], John A.l. Armour [2], Fengtang Yang [1]

[1]Wellcome Trust Sanger Institute, Molecular Cytogenetics, Cambridge, UK; [2]University of Nottingham, Medical School, Queen's Medical Centre, School of Life Sciences, Nottingham, UK

**Correspondence:** Sandra Louzada

*Molecular Cytogenetics* 2017, **10(Suppl 1):**O4

In spite of the rapid development in genome technology, the characterization and validation of complex structural variants (SVs) involving tandem low copy repeats and multi-allelic gene families remains a challenge. Recently we have been exploring the potential of combed DNA-fibres in the characterisation of complex SVs in human populations by multicolour fibre-FISH. The human amylase gene cluster harbours the salivary (AMY1) and pancreatic amylase genes (AMY2A and AMY2B). Early comparative genomic studies revealed copy number variations (CNV) in the amylase genes, which were shown to be linked with human adaptation to dietary starch intake, and implicated in predisposition to obesity. We have employed an array of high-resolution measurement methods, such as, whole-genome sequencing, paralogue ratio test (PRT), and optical mapping, together with fibre-FISH to characterise the amylase SV haplotypes in different populations. The results validate haplotype structures for AMY1 and show that pancreatic amylase genes underwent at least five independent rearrangements to create new haplotypes, one of which contains five copies each of the AMY2A and AMY2B genes in sub-Saharan African population. Fibre-FISH not only enabled a direct visualization of the SV haplotype structure by delineating the order, orientation, and absolute copy number of amylase genes, but also provided direct evidence for an inversion event that accompanies higher order expansion of AMY2A and AMY2B and a non-homologous junction where the upstream sequence of AMY2A interrupts one copy of AMY1. Such rearrangements were not detected by other methodologies initially, thus vindicating the use of fibre-FISH as the method of choice for validating complex SVs.

### O5

### Balanced X autosome translocation suggests association of AMMECR1 disruption with hearing loss short stature bone and heart alterations

Mariana Moyses-Oliveira [1], Richard Fish [2], Giuliana Giannuzzi [3], Ender Karaca [4], Zeynep Akdemir [4], Florence Petit [5], Vera Meloni [1], Maria Soares [6], Leslie Kulikowski [7], Adriana Di Battista [1], Malu Zamariolli [1], Thomas Liehr [8], Nadezda Kosyakova [8], Gianna Carvalheira [1], Joris Andrieux [9], Marguerite Neerman-Arbez [2], James Lupski [4], Maria Isabel Melaragno [1], Alexandre Reymond [3]

[1]Universidade Federal De São Paulo, Department of Morphology And Genetics, São Paulo, Brazil; [2]University of Geneva, Department of Genetic Medicine And Development, Geneva, Switzerland; [3]University of Lausanne, Center For Integrative Genomics, Lausanne, Switzerland; [4]Baylor College of of Medicine, Department of Molecular And Human Genetics, Houston, TX, USA; [5]Hôpital Jeanne De Flandre, Clinique De Génétique, Lille, France; [6]Universidade Federal De São Paulo, Department of Psychobiology, São Paulo, Brazil; [7]University of São Paulo, Department of Pathology, São Paulo, Brazil; [8]Friedrich Schiller University, Institute of Human Genetics, Jena, Germany; [9]Hôpital Jeanne De Flandre, Institut De Génétique Médicale, Lille, France

**Correspondence:** Mariana Moyses-Oliveira

*Molecular Cytogenetics* 2017, **10(Suppl 1):**O5

Female balanced X-autosome translocations have been associated with absence of functional copies of the gene mapping at the breakpoint through disruption of the derivative chromosome and inactivation of the normal X-chromosome. We report on a nine year-old girl with karyotype 46,X,t(X;9)(q23;q12)dn, disproportionate short stature, septal atrial defect, scoliosis, bone dysplasia, hearing loss, and normal cognition. Array-CGH and breakpoint sequencing confirmed the full complement of genetic material, whereas replication banding and HUMARA assay showed preferential inactivation of the normal X-chromosome. Both junction points were determined at the nucleotide level. The autosomal breakpoint affects a heterochromatic region, while the X-chromosome breakpoint was mapped between the AMMECR1 and RGAG1 genes. Whereas expression of the latter was unmodified, RT-qPCR showed absence of AMMECR1 expression in blood and lymphoblastoid cells of the proband. Concordantly, we identified an individual with nonsense mutation in AMMECR1 and short stature, septal atrial defect, radioulnar synostosis, hearing loss, and psychomotor and speech delay. We also describe three individuals with missense mutation and one with AMMECR1 duplication and overlapping phenotypes. The encoded protein contains evolutionary conserved nucleic acid-binding RAGNYA folds and localizes to the nucleus. AMMECR1 is co-expressed with genes implicated in cell cycle and translation regulation, five of which were previously associated with growth and bone alteration syndromes. Our knockdown of the zebrafish orthologous gene resulted in animals with features reminiscent of the patients' phenotype such as shorter tails, thinner bodies, kinked tail-ends, poorly defined somites, pericardial edema, tachycardia and hydrocephaly. Part of the knockdown phenotypes were rescued by co-injection of mRNA from the human ortholog. Our results suggest that AMMECR1 is potentially involved in cell cycle control and linked to a new syndrome(s) characterized by hearing loss, short stature, bone and heart alterations.
**Consent to publish:** The authors confirm that written informed consent was received by the patient for publication.

### O6

### Monitoring Guide RNA Synthesis for CRISPR Cas9 Genome Editing Workflow

Isabell Pechtl [1], Melissa Liu [1], Arunkumar Padmanaban [2]
[1]Agilent Technologies Inc., Marketing, Waldbronn, Germany; [2]Agilent Technologies Inc., Application Development, Bangalore, India
**Correspondence:** Isabell Pechtl
*Molecular Cytogenetics* 2017, **10(Suppl 1):**O6

Bacterial clustered regularly interspaced short palindromic repeats (CRISPR) - associated protein 9 (Cas9) system has increased in popularity as a genome editing tool for targeted mutations, insertions, deletions and gene knock-out studies. CRISPR genome editing has also proved superior to Zinc-finger nucleases (ZFN) and transcription activator- like effector nucleases (TALENs) due to its simplicity and easy programmability. In CRISPR, a guide RNA (gRNA) is used to recognize and introduce a double standard break (DSB) in a target DNA. The DNA repair mechanism triggered after the break is then exploited to introduce an insertion/deletion (indel) in the case of non-homologous end joining (NHEJ), or precise genetic modification if a homology-directed repair (HDR) pathway is triggered.
A critical part of the CRISPR/Cas9 tool is the design and synthesis of the gRNA that comprises T7 promoter sequence, target sequence, and protospacer adjacent motifs (PAM). Monitoring the transcription of the gRNA is critical to the workflow to ensure successful gene editing. Here we present an automated electrophoresis approach for monitoring the synthesis, integrity, and functional activity of gRNAs created for a CRISPR-Cas workflow.

### O7

### Chromosome territories in mice spermatogenesis: a new three-dimensional methodology

Mireia Solé [1], Joan Blanco [1], Debora Gil [2], Oliver Valero [3], Gothami Fonseka [4], Richard Frodsham [4], Francesca Vidal [1], Zaida Sarrate [1]
[1]Universitat Autònoma de Barcelona Departament de Biologia Cel · lular, Fisiologia i Immunologia Cerdanyola del Vallès, Spain; [2]Computer Vision Center - Universitat Autònoma de Barcelona Computer Science Department Cerdanyola del Vallès, Spain; [3]Universitat Autònoma de Barcelona Servei d'Estadística Aplicada Cerdanyola del Vallès-Spain; [4]Technopark Cytocell Ltd Cambridge, UK
**Correspondence:** Mireia Solé
*Molecular Cytogenetics* 2017, **10(Suppl 1):**O7

Chromosomes occupy specific nuclear regions called chromosome territories (CT) which are arranged in cell-type specific non-random patterns and are involved in genome regulation. In spermatogenic cells, a non-random distribution of chromosomes has been demonstrated in pachytene and metaphase I spermatocytes and in spermatozoa. However, most studies have been carried out using two-dimensional strategies, in particular spermatogenic stages and evaluating few chromosomes.
To overcome these limitations, we have developed an in situ fluorescent hybridization (FISH)-based protocol to approach the three-dimensional study of CTs along spermatogenesis. Testicular tissue from fertile mice C57BL6/6 J was enzymatically disaggregated. To preserve the nuclear structure, cell suspensions were spread out on polylysine-coated slides and fixed with paraformaldehyde. Subsequently, slides were frozen in liquid nitrogen and treated with pepsin. Three successive FISH rounds were carried out using the customized kit Chromoprobe Multiprobe® OctoChrome™ Murine System (Cytocell Ltd, Cambridge, UK) designed to identify the entire mouse karyotype. This kit uses seven different combinations of three whole chromosome painting probes directly labeled in three different fluorochromes. Afterwards, SYCP3 and H1T were identified by immunofluorescence staining to categorize among pre-meiotic cells, meiotic figures (discriminating all stages from leptotene to metaphase I and metaphase II), post-meiotic cells and spermatozoa. Serial optical sections of all cell types were obtained with a TCS-SP5 confocal microscope coupled to an imaging analysis system (LAS AF-1.8.1). After processing images by ImageJ, Matlab developed scripts were used to align, normalize and process nuclei in order to determine chromosome volume and proportion, chromosome radial position and chromosome relative position.
The application of the methodology developed allows the establishment of CTs throughout all spermatogenic stages providing a new basis to study the relationship between chromosome positioning and genome regulation.
Financial support: CF-180034 (UAB, Spain), DPI2015-65286-R/SAF2016-77165-P (MINECO, Spain) and CERCA Programme (Generalitat de Catalunya, Spain). M Solé is recipient of a grant from UAB (PIF/2015).

### O8

### A geography of clones mapping the tumour genome over anatomic space in children with cancer

Jenny Karlsson [1], Anders Valind [1], Caroline Jansson [1], Ingrid Øra [2], David Gisselsson [1]
[1]Lund University, Clinical Genetics, Lund, Sweden; [2]Lund University, Paediatrics, Lund, Sweden
**Correspondence:** David Gisselsson
*Molecular Cytogenetics* 2017, **10(Suppl 1):**O8

**Objectives**
To chart genetic intratumour diversity in childhood cancer over multiple anatomic locations and time points during treatment in order to (1) delineate common routes of cancer cell evolution, (2) gain information on intrapatient variability of clinical biomarkers, and (3) assess clinical correlations to evolutionary patterns.
**Methods**
Patients were included based on the availability of two or more informative samples from the primary tumour, taken with a minimum intersample distance of 10 mm. A total of 55 patients with neuroblastoma (n = 24), Wilms tumour (n = 23) or rhabdomyosarcoma (n = 8) were subjected to multiregional analysis of tumour tissue with high resolution whole genome genotyping arrays (all patients) complemented by whole exome sequencing and targeted deep DNA sequencing (n = 20). Between two and 20 tumour samples were analyzed per patient resulting in total of 240 informative tumour samples.
**Results**
The majority of cases exhibited intratumour genetic diversity with branching evolution, including variability of several suggested clinical biomarkers. There were also features of convergent evolution in branches of evolutionary trees, including trisomies and monosomies, as well as TP53 mutations in Wilms tumours, CDKN2A deletions in neuroblastoma, and CDK4 amplifications in rhabdomyosarcoma. Subclones were the major arena of genome evolution. We observed four major trajectories of cancer cell evolution including (1) subclonal variation, (2) subclone coexistence, (3) subclonal explosions, and (4) clonal sweeps. These trajectories exhibited a striking correlation to clinical risk groups.

**Conclusion**

Even in very young patients, cancer is a genetically dynamic disease over space and time. On the one hand, intratumour diversity is a significant source of error in genetic biomarker assessment. On the other hand, specific evolutionary patterns may be useful as future clinical predictors.

### O9

### Novel recurrent chromosome anomalies in Shwachman Diamond syndrome

Emanuela Maserati [1], Roberto Valli [1], Lucia Nacci [2], Annalisa Frattini [3], Federico Grilli [1], Francesco Pasquali [1]

[1]University Of Insubria, Department of Medicine And Surgery, Varese, Italy; [2]University of Pavia, Department of Molecular Medicine, Pavia, Italy; [3]National Council of Research, Irgb, Milano, Italy

**Correspondence:** Emanuela Maserati

*Molecular Cytogenetics* 2017, **10(Suppl 1):**O9

Clonal chromosome anomalies are frequently acquired in the bone marrow (BM) of patients with Shwachman-Diamond syndrome (SDS), and two are the most frequent: an isochromosome of the long arm of chromosome 7, i(7)(q10), and an interstitial deletion of the long arm of chromosome 20, del(20)(q). Patients with SDS have a risk of developing myelodysplasia (MDS) and/or acute myeloid leukaemia (AML), and the presence of chromosome changes was studied in relation with this risk. Starting in 1999 we have monitored the cytogenetic picture of a cohort of 92 Italian patients with SDS by all suitable cytogenetic and molecular methods. Clonal anomalies in BM were present in 41/92 patients. The i(7)(q10) was observed in 16 patients, and the del(20)(q) in 15, both these changes in four, but in independent clones. So, the most frequent clonal anomalies were found in 35 patients. Other, different, clonal anomalies were found in the BM of 13 patients, in eight cases in the absence of i(7)(q10) or del(20)(q), in five cases in association with one of these changes. In these less common clonal anomalies, the distribution of the chromosomes involved was markedly disparate, and some of them were novel and recurrent:

- structural rearrangements of chromosome 7, mainly unbalanced (deletions, inversions or translocations), were present in five of our 13 patients, three of whom developed MDS/AML.
- a further complex rearrangement of the more common del(20)(q), leading to duplicated and deleted portions, was identical in two patients, with almost identical a-CGH profiles, neither developed MDS/AML.
- an unbalanced translocation t(3;6), with partial trisomy of the long arm of chromosome 3 and partial monosomy of the long arm of chromosome 6, was not identical but very similar in two patients, one of whom developed MDS/AML.

### O10

### Deletion 13q characterised by SNP microarray profiling of a large cohort of CLL patients

Nicole Chia [1], Dr Melody Caramins [2]

[1]Qml Pathology, Genetics, Murrarrie, Australia; [2]Sds Pathology, Genomic Diagnostics, Sydney, Australia

**Correspondence:** Nicole Chia

*Molecular Cytogenetics* 2017, **10(Suppl 1):**O10

The investigation of Chronic Lymphocytic Leukemia (CLL) by conventional cytogenetic analysis combined with fluorescence in-situ hybridisation (FISH) has been regarded as the gold standard. Detection and characterisation of non-random rearrangements is hindered in part by the limitation of cell culturing and the targeted approach of FISH testing. More recently the implementation of molecular karyotyping by high resolution Single Nucleotide Polymorphism (SNP) microarray platforms has augmented the investigation protocol of haematological malignancies in diagnostic pathology.

Deletion 13q is reported in up to 50% of patients with CLL and has historically been reported in association with low risk disease. Molecular karyotyping has revealed the diversity and complexity of rearrangements within the del13q region. This has culminated in the classification of the 13q deletion into type I (exclusive of Rb1) and type II (inclusive of Rb1). However uncertainty exists with regard to the prognostic significance, particularly with respect to the large, type II deletion.

To evaluate this we investigated the molecular karyotype of 500 CLL patients. We ascertained the incidence of del13q type I and type II, complexity of deletions, association with additional karyotypic changes and cnLOH chromosome 13. Our results show that there is no significant difference in the incidence or association with additional karyotypic changes, but type II deletion is associated with complex 13q deletions ($p = 0.0001$). Furthermore there is no significant difference with respect to cytogenetic progression in follow-up testing of 43 patients. This study highlights the benefit of SNP microarray analysis in characterising deletion anatomy and the need for re-evaluation of risk stratification of non-random rearrangements.

### O11

### The transcriptome plasticity of genome amplification in cancer

Gemma Macchia [1], Alberto L'abbate [1], Doron Tolomeo [1], Grazia Visci [1], Giulia Daniele [1], Angelo Lonoce [1], Lucia Anna Muscarella [2], Clelia Tiziana Storlazzi [1]

[1]University of Bari "aldo Moro", Biology, Bari, Italy; [2]Irccs Casa Sollievo Della Sofferenza, Laboratorio Di Genetica Medica, San Giovanni Rotondo, Italy

**Correspondence:** Gemma Macchia

*Molecular Cytogenetics* 2017, **10(Suppl 1):**O11

Genome amplification, in the form of homogeneously staining regions, double minutes, and ring/giant rod-shaped markers, is a pivotal event in many tumors. It was recently shown that amplifications as extrachromosomal DNA are present in nearly half of all tumors, representing a driving force towards their accelerated evolution. To achieve a better understanding of the implications of genomic amplifications we focused on their structure and impact upon transcription. Amplified cancer-associated genes are often overexpressed as a direct consequence of the copy number gain. We analyzed the whole genome (WGS) and transcriptome (RNA-seq) sequencing data of nine small cell lung carcinoma (SCLC), seven neuroblastoma (NB) and three well-differentiated liposarcoma (WDLPS) cell lines, all carrying genomic amplifications. A widespread heterogeneity of the amplicon arrangement was detected in most of the samples, disclosing the progressive evolution of their structure through cell divisions. By integrating the WGS (structural variation calling) and RNA-seq (chimeric transcripts detection) data, we detected a burst of chimeras derived from post-transcriptional events (i.e. cis- or trans-splicing) involving amplified genes. Notably, we found PVT1 (8q24) and RLF (1p34.2) as hotspots for cis- or trans-splicing events in SCLC and NB samples with MYC and MYCL1 amplifications, respectively. In WDLPS cell lines, we found extremely complex fusion genes, such as those involving three partners or assembled by multiple interposed non-contiguous, non-collinear genomic fragments (spliced out in the mature transcript). Our results strongly indicate that the "amplification/overexpression" paradigm does not cover all aspects of the genomic amplification impact upon transcription. The extraordinary transcriptome plasticity herein described, enriching the genetic repertoire of cancer cells with genomic amplifications, likely provides a selective advantage and might have a crucial role in cancer establishment and progression.

### O12

### Prospective diagnostic evaluation of genetic abnormalities in childhood acute lymphoblastic leukemia with hub gene FISH screening and array analyses

Karin Nebral [1], Sabrina Haslinger [1], Andrea Inthal [1], Margit Koenig [1], Brigitte Grimm [1], Soeren Mai [1], Maya-Marisol Plank [1], Andishe Attarbaschi [2], Georg Mann [2], Oskar A. Haas [1]
[1]Ccri, Children's Cancer Research Institute, Cytogenetics And Molecular Cytogenetics, Vienna, Austria; [2]St. Anna Children's Hospital, Medical University, Pediatric Hematology And Oncology, Vienna, Austria
**Correspondence:** Karin Nebral
*Molecular Cytogenetics* 2017, **10(Suppl 1):**O12

The systematic definition of genomic alterations in childhood ALL is not only an indispensable prerequisite for its predictive and prognostic stratification but increasingly also one, on which individualized therapies are based on. Of particular interest in this context are novel classes of recurrent gene fusions, such as the therapeutically targetable tyrosine kinase and JAK2-pathway activating ones, and the more elusive group whose gene expression pattern and/or vaguely defined combinations of mainly gene region-specific copy number alterations (CNA). Based on these necessities we developed a diagnostic strategy that combines hierarchical FISH screening for the systematic identification of all relevant gene fusions with an array-based evaluation of CNA. Cytogenetic preparations serve merely as backup for further studies. To evaluate the feasibility of our particular workflow we prospectively analyzed 117 cases so far, 95 newly diagnosed and 10 relapsed B- as well as 12 T-cell precursor ALL. With this approach we were able to classify all but one B-cell cases in a meaningful manner: 41 hyperdiploid (including one with a hypo- and hyerdiploid clone), 21 ETV6-RUNX1, 7 KMT2A, 6 TCF3-PBX1, 2 BCR-ABL1, 3 dic(9;20), 5 IGH-rearranged, 4 P2RY8-CRLF2, 3 PAX5, and, 14 B-other (including various types of ZNF384, JAK2, ETV6 fusions) ones. FISH screening was positive in 105/117 cases and provided already the most essential diagnostic clues in the vast majority of them. CNA were present in 113/117 patients, including 21 IKZF1 and 2 ERG deletions, which will serve as stratifying markers in future. In consideration of the amount of achievable information, our standardized diagnostic approach is very well suited for this purpose, because it is applicable to individual cases, robust, cost-efficient, requires only little material and thereby also serves as discovery platform.

### O13

### Apparently identical heterozygous neocentromeres in two closely related Cercopithecini species

Giorgia Chiatante [1], Oronzo Capozzi [2], Luca Sineo [3], Roscoe Stanyon [1]
[1]University of Florence, Biology, Florence, Italy; [2]University of Bari, Biology, Bari, Italy; [3]University of Palermo, Scienze E Tecnologie Biologiche Chimiche E Farmaceutiche, Palermo, Italy
**Correspondence:** Roscoe Stanyon
*Molecular Cytogenetics* 2017, **10(Suppl 1):**O13

The Cercopithecini tribe (guenons) of the Old World monkeys, has experienced rapid chromosome evolution with diploid number from 2n = 48 to 72. All Cercopithecini have a syntenic association of chromosomes homologous to human 20 and 21. Chromosome painting and G-banding suggested that two isoforms of this chromosome exist with different centromere positions. In one form, the centromere appears at the fusion point of the association while in the other it is located in the segment homologous to chromosome 20. Here, we used a gamma of molecular cytogenetic methods including BAC-FISH to examine the 20/21 association in a number of Cercopithecini species. We discovered that individuals from two closely related species Cercopithecus mitis (CMI) and Cercopithecus petaurista (CPE) were heterozygous for centromere location. The centromere within the segment homologous to HSA20 is a neocentromere. In CMI this centromere appears completely devoid of satellite DNA, while in CPE it appears to have a repeat structure more similar to a normal, mature centromere. Our data allowed us to speculate on the evolutionary process related both to Cercopithecini phylogenomics as well as to neocentromere formation and maturation. One hypothesis is that these neocentromeres had a common origin and survived a speciation event. Yet, they apparently show significant difference in maturation. These heterozygous neocentromeres certainly represent a unique opportunity through future sequencing efforts to better understand the phenomena of neocentromere seeding and evolution.

### O14

### P1002 - Meiotic outcome in two carriers of Y autosome reciprocal translocations selective elimination of certain segregants

Harita Ghevaria[1], Roy Naja[1], Sioban Sengupta[1], Paul Serhal[2], Joy Delhanty [1]
[1]University College London, Institute For Women's Health, London, UK; [2]The Centre For Reproductive And Genetic Health, Director, London, UK
**Correspondence:** Harita Ghevaria
*Molecular Cytogenetics* 2017, **10(Suppl 1):**O14

This abstract is not included here as it has already been published.

### O15

### P1282 - Deletion encompassing DLK1 gene at 14q32 imprinted region in two new Temple syndrome cases

Neus Baena [1], Natalia Claver [1], Cinthia Aguilera [1], Elisabeth Gabau [2], Nuria Capdevila [2], Anna Ruiz [1], Miriam Guitart [1]
[1]Corporació Sanitaria Parc Tauli, Genetics Laboratory, Sabadell. Barcelona, Spain; [2]Corporació Sanitaria Parc Tauli, Clinical Genetic Unit, Abadell. Barcelona, Spain
**Correspondence:** Neus Baena
*Molecular Cytogenetics* 2017, **10(Suppl 1):**O15

**Introduction**

Temple syndrome (TS) is a rare condition caused by an abnormal expression of genes at the imprinted locus 14q32. Different molecular mechanisms cause TS: 1) Maternal uniparental disomy 14; 2) loss of paternal methylation at the intergenic differentially methylated region on chromosome 14q32.2; 3) paternal deletion involving the region of interest. Until now, nine cases of TS caused by a paternal deletion have been reported.

We report clinical and molecular features of two new patients affected by a neurodevelopmental disorder with deletion involving the 14q32 imprinted region. The deletions were identified by aCGH (8x60K ISCA, Agilent technologies).

**Methods**

The MS-MLPA (MS-MLPA probemix ME032-A1 UPD7/UPD14, MRC Holland) technique was used to determine the inheritance, methylation pattern and dose analysis.

The first patient is a three years old male showing short stature, psychomotor retardation and hypotonia. In the prenatal period he presented an intrauterine growth retardation and oligomamnios. The aCGH detected a 14q32.2 deletion of 1 Mb. The second patient is a two years old female harbouring mild psychomotor retardation and hypotonia. She has a 69 kb deletion.

**Results**

The deletion of 1 Mb (27 genes being the most relevant DLK1, MEG3 and RTL1) is de novo and the pattern of methylation was hypomethylated in the maternal allele, thus the paternal allele is deleted diagnosing a Temple Syndrome.

The 69 kb deletion (includes only DLK1) and the methylation pattern is normal. This deletion is inherited from her father and from her grandmother.

**Conclusions**

In the second case the loss of expression of the DLK1 gene in the paternal allele is responsible for the clinical features of psychomotor retardation and mild hypotonia suggesting a Temple syndrome. This small deletion narrowed the minimal region supporting that DLK1 loss of function is sufficient to cause the main features of Temple syndrome. In this family DLK1 deletion was inherited from the grandmother but only the father carrier has a 50% risk to give birth to children with Temple syndrome.

**Consent to publish:** The authors confirm that written informed consent was received by the patient for publication.

### O16

### External Quality Assessment of Clinical Genetics: experiences with the pilot assessments

Conny van Ravenswaaij-Arts [1], Christi van Asperen [2], Caroline Benjamin [3], Livia Garavelli [4], Borut Peterlin 5, Maartje Nielsen [2], Tommy de Ravel [6], Lisbeth Tranebjaerg [7], Kini Usha [8], Karin Writzl [5], Ros Hastings [9]

[1]Groningen University Medical Centre Dept of Genetics Groningen, The Netherlands; [2]Leiden University Medical Centre Dept of Medical Genetics Leiden, The Netherlands; [3]University of Central Lancashire College of Health & Wellbeing Preston, UK; [4]Istituto di Ricovero e Cura a Carattere Scientifico Dipartimento Ostetrico-Ginecologico e Pediatrico Reggio Emilia, Italy; [5]University Medical Center Ljubljana Clinical Institute of Medical Genetics Ljubljana-Slovenia; [6] KU Leuven Dept of Human Genetics Leuven, Belgium; [7]Bispebjerg Hospital/Rigshospitalet Professor of Medical Genetics and Genetic Audiology Copenhagen, Norway; [8]OUH Trust Dept Clinical Genetics Oxford, UK; [9]OUH Trust CEQAS Oxford, UK

**Correspondence:** Ros Hastings

*Molecular Cytogenetics* 2017, **10(Suppl 1):**O16

Quality assessment has long been associated with laboratory, but not clinical services. To address this obvious gap, in 2012 the ESHG Genetic Services Quality Committee explored the need for a European Quality Assessment (EQA) scheme for clinical genetics and counselling. European national societies of human genetics were surveyed and a need for EQA for clinical genetics was expressed. CEQAS was chosen as the EQA provider and three pilot EQAs have now been completed and evaluated.

Each year, the Clinical Genetics Working Group prepared four case scenarios in the fields of cardiogenetics, oncogenetics, monogenic disorders and dysmorphology. Each case scenario started with a referral letter and consisted of multiple stages, to reflect an episode of clinical care. At each stage further clinical and genetic test information was given and a number of questions presented. For each EQA, consensus answers were provided by two independent experts including at least two clinical geneticists and a patient organisation. Twelve pilot EQAs have been distributed over the last three years and in 2016, 42 genetic centres from 21 countries enrolled. All EQA submissions were reviewed by two clinical geneticists.

The EQA for clinical genetics and counselling proved to be highly educational. Learning objectives are included the summary letter. The EQA identified that some centres omitted to include the need:-

- for consent of the index patient;
- for a three generation family history to be taken;
- to discuss the implications of a recessive disorder;
- to give comprehensive reproductive options;
- for psycho-social support for the patient and family.

In addition, the assessors identified that some case scenarios tried to cover too many aspects and needed to be streamlined so that only 3–4 learning points were covered. This talk will present the data from the pilot EQAs and discuss some of the genetic counselling issues identified.

### O17

### The majority of uncommon chromosomal imbalances detected by NIPT are postzygotic (feto)placental mosaics

Van Den Bogaert Kris, Brison Nathalie, Dimitriadou Eftychia, Melotte Cindy, Dehaspe Luc, Destouni Aspasia, Breckpot Jeroen, Peeters Hilde, Van Esch Hilde, Van Buggenhout Griet, Vogels Annick, Legius Eric, De Ravel Thomy, Devriendt Koen, Vermeesch Joris R

[1]University Hospitals Leuven - Kuleuven, Department of Human Genetics, Leuven, Belgium

**Correspondence:** Van Den Bogaert Kris

*Molecular Cytogenetics* 2017, **10(Suppl 1):**O17

Using clinical analysis of over 20.000 pregnancies, we show that non-invasive prenatal testing (NIPT) by in-house optimized genomic imbalance profiling (GIPseq) increases the sensitivity for detection of fetal trisomy 21, 18 and 13 to 100%, 97,2% and 100% respectively without reducing the specificity, which exceeds 99,9% for each of these common trisomies. Furthermore, NIPT by GIPseq offers the advantage of detecting other genomic imbalances that are clinically relevant for fetal or maternal health. These findings include (i) other aneuploidies, (ii) fetal or maternal segmental imbalances and (iii) maternal cancer.

We show that, although uncommon aneuploidies mostly exist as confined placental mosaicism, some chromosomal imbalances can pass the developmental barrier, resulting in complex fetal segmental anomalies with severe clinical implications. Such anomalies can only be the consequence of pre-blastocystic cell lineages harboring different imbalances, which further evolve following the separation of the inner cell mass and the trophectoderm subpopulations. It has been shown that a small but significant number of trophectoderm biopsies analyzed by genome-wide aneuploidy detection methods during preimplantation genetic diagnosis (PGD) or screening (PGS) is poised with mosaic chromosomal imbalances. Within the PGD community there is currently a debate about the clinical importance of these imbalances.

Here we demonstrate that (1) most uncommon chromosomal imbalances originate post fertilization, (2) they can pass the developmental barrier and be present at different loads in the fetus and/or the placenta, (3) detection of imbalances in trophectoderm is of clinical importance and (4) detection of segmental imbalances via NIPT provides valuable information for pregnancy management.

### Poster Abstracts

### Clinical Cytogenetics

### 1.P1

### A rare familial paracentric inversion of the long arm of chromosome 8

Elisavet Koyvidi [1], Haroula Tsarouha [1], Sophia Zachaki [1], Anastasios Mitrakos [1], Konstantinos Sfakianoudis [2], Emmanouel Kanavakis [1], Ariadni Mavrou [1]

[1]Genesis Genoma Lab, Cytogenetics, Athens, Greece; [2]Genesis Athens Clinic, Gynaecological, Athens, Greece

**Correspondence:** Elisavet Koyvidi

*Molecular Cytogenetics* 2017, **10(Suppl 1):**1.P1

**Background**

The incidence of paracentric inversions (PAI) in the general population ranges from 0.09-0.49/1,000 and PAIs are generally considered to be harmless.

**Aim**

To present a rare case of a carrier of an 8q paracentric inversion.

### Case report
A 37-year-old man and his 30-year-old wife were referred for karyotype analysis due to a previous miscarriage at 33 weeks of gestation. The woman had gestational diabetes mellitus and was under insulin treatment.

### Methods/Results
Cytogenetic analysis was performed on peripheral blood lymphocytes by GTG banding. The wife had a normal karyotype 46,XX but the man's karyotype was 46,XY,inv(8)(q23.1q24.2). Karyotypic analysis of his parents revealed that his mother carried the same inversion. Molecular karyotype analysis performed using Agilent G3 4x180 K CGH + SNP microarray platform, which reported no duplication or loss in or near the inverted 8q region.

### Conclusions
Until now, only three 8q PAI cases have been published. Although the risk of a PAI carrier having an abnormal child is very low, caution is indicated in distinguishing inversions from insertions.

**Consent to publish:** The authors confirm that written informed consent was received by the patient for publication.

### 1.P2
### Chromosomal abnormalities in infertile couples referred for assisted reproduction techniques
Sophia Zachaki, Elisavet Kouvidi, Haroula Tsarouha, Ioanna Haralampous, Amelia Pantou, Emmanouel Kanavakis, Ariadni Mavrou
Genesis Genoma Lab, Cytogenetics, Athens, Greece
**Correspondence:** Sophia Zachaki
*Molecular Cytogenetics* 2017, **10(Suppl 1):**1.P2

### Background
The frequency of chromosomal abnormalities in infertile individuals is higher than in the general population, varying from1.3-15.0%.

### Aim
To determine the prevalence of chromosomal abnormalities in infertile couples undergoing assisted reproduction techniques (ART).

### Methods
Cytogenetic analysis was performed on peripheral blood lymphocytes using standard G-banding techniques in 283 infertile couples referred to Genesis Athens Clinic in the second half of 2016. In 218 cases both partners were tested and in 65 cases one only. The mean age of the individuals studied was 39.6 years (41.1 in men and 38.1 in women). At least 25 metaphases were analyzed and in cases of suspected mosaicism, 100 cells were counted. Chromosomal polymorphisms such as prominent satellites, increased heterochromatic regions and pericentric inversions of chromosome 9 were not included.

### Results
In 69/283 couples, one partner carried a chromosomal abnormality: Four women had a structural chromosomal abnormality (2 reciprocal translocations, 1 Robertsonian and 1 inversion). The most frequent finding was sex chromosome mosaicism. The mean age of women with a sex mosaic karyotype was significantly higher than those with a normal karyotype (40.6 vs 37.5 years). Six men were found to carry a structural chromosomal abnormality (4 reciprocal translocations and 2 inversions) and 4 had sex chromosome mosaicism.

### Conclusions
The frequency of structural chromosomal abnormalities in the population studied was in agreement with the one reported in the literature. The frequency of sex chromosome mosaicism was high in the study group, probably due to age-related X chromosome loss. Our results underline the importance of chromosomal analysis in infertile couples prior to ART treatment in order to select the appropriate protocol, including preimplantation genetic diagnosis for couples with structural chromosome abnormalities.

### 1.P3
### Two brothers with atypical 17q21.31 microduplication
Hana Zůnová, Markéta Havlovicová, Miroslava Hančárová, Drahue Novotná, Jana Paděrová, Zdeněk Sedláček, Jana Drábová
Charles University 2nd Faculty of Medicine And University Hospital Motol, Department of Biology And Medical Genetics, Prague, Czech Republic
**Correspondence:** Hana Zůnová
*Molecular Cytogenetics* 2017, **10(Suppl 1):**1.P3

### Introduction
17q21.31 microduplication syndrome is characterized by duplication of the MAPT region that is associated with the well-known 17q21.31 microdeletion syndrome. This region ranges from 440 to 680 kb and includes five genes, CRHR, MAPT, IMP5, STH and KANSL1. There are two haplotypes of the MAPT region, H1 and H2. H2 undergoes non-allelic homologous recombination (NAHR) that can cause deletions and duplications of the entire region.

### Methods
We present a 10-year-old boy and his 7-year-old brother. They were referred to genetic consultation due to delayed development, facial dysmorphism and autism spectrum disorder. DNA was extracted from peripheral blood and analyzed using array-CGH (aCGH) on the platform SurePrint CGH 8x60K G3 ISCA (Agilent Technologies) and the CytoGenomics software.

### Results
Array-CGH analyses of both probands showed the same 375 kb duplication of 17q21.31. The duplication included three genes, KANSL1, STH and MAPT. The mode of inheritance was determined as de novo. Neither of the parents was a carrier of the risk H2 allele.

### Conclusion
Phenotypic features of the probands are typical for the 17q21.31 duplication syndrome. However, the analysis showed a smaller duplication that did not affect all genes typically included in 17q21.31 duplications. The study of this family can help to understand the mechanism of pathogenesis of the syndrome. Both the size of the duplication and the absence of H2 alleles in the parents argue against NAHR as the mechanism of origin. Because of the presence of the same rare aberration in both brothers, we can presume germinal mosaicism in one of the parents.

**Supported by:** MZ 0064203, NF-CZ11-PDP-3-003-2014

**Consent to publish:** The authors confirm that written informed consent was received by the patient for publication.

### 1.P4
### Detection of a minor deletion affecting U1B upstream exon of the SNURF SNRPN gene in a survey for identification of Prader Willi Syndrome
Carmen Lucia Antao Paiva [1], Suely Rodrigues Dos Santos [1], Catielly Ferreira Rocha [2]
[1]Unirio, Department of Genetics And Molecular Biology, Rio De Janeiro, Brazil; [2]Unirio, Ppgneuro, Rio De Janeiro, Brazil
**Correspondence:** Carmen Lucia Antao Paiva
*Molecular Cytogenetics* 2017, **10(Suppl 1):**1.P4

### Introduction
Prader-Willi syndrome (PWS), one of the most frequent microdeletion syndromes, is a complex multi-organ disease caused by the lack of expression of paternal genes of 15q11-q13. It is a common cause of severe obesity associated with cognitive disabilities.

### Objective
The aim of this work was to investigate PWS using Methylation-Specific PCR (MS-PCR) and Multiplex Ligation Probe Amplification (MLPA), in order to find minor abnormalities of 15q11-q13 in MS-PCR-negative patients.

**Methods**
Thirty-five patients were tested with MS-PCR and MLPA (kit PWS-ME028 MRC-Holland)
**Results**
Twenty two patients had a negative MS-PCR testing; among them, MLPA analysis detected one patient with a minor deletion affecting U1B upstream exon of the SNURF/SNRPN gene.
**Discussion**
Koufaris et al. (2016) reported a deletion of SNURF/SNRPN U1B and U1B* upstream exons in one child with developmental delay and excessive weight. They did not find any published reports of patients with PWS-like symptoms that carried deletions specifically affecting the U1B–U1B* genomic region. They suggested that further case reports with mutations affecting U1B exons are needed to verify the biological significance of this region to PWS. Therefore, we report a case of a patient who showed a minor deletion affecting U1B upstream exon of the SNURF/SNRPN gene. One explanation for the negative MS-PCR testing is the following: the primers suggested by Martinez et al. (2006) to amplify a 487-bp fragment containing exon 1 was not able to cover the U1B region.
**Conclusion**
As far as we know, this is the second case reported with U1B deletion. Therefore, we may suggest that the loss of SNRPN U1B could be responsible for some features of the syndrome, although some other new cases are needed to definitively confirm the hypothesis.

### 1.P5

### Autism Spectrum Disorder (ASD) associated with a Non mosaic two isodicentric Y male Karyotype

Mira Ziv [1], Yarin Hadid [1], Hana Shapira [1], Jasmin Levitaz [1], Dalia Fridman [1], Svetlana Fridman [1], Adel Shalata [1]
[1]Bnai Zion, The Simon Winter Institute For Human Genetics, Haifa, Israel
**Correspondence:** Adel Shalata
*Molecular Cytogenetics* 2017, **10(Suppl 1):**1.P5

Tetrasomy Y is a very rare event, especially in the Non-mosaic form. In literature all patients show different degrees of mental retardation, various skeletal abnormalities and facial dysmorphism.
In this abstract we describe a 7 year old boy who is the first child of a young healthy non related Israeli Arab couple. He was born after a normal pregnancy and delivery. At the age of two years old he was diagnosed with Global Developmental Delay and recently, a neurodevelopmental re-evaluation changed the diagnosis to a moderate Autism.
His physical and neurological examination, except mild dysmorphic features including retrognathia and crowded teeth, was normal. The brain MRI study demonstrated several regions with prolonged relaxation time mainly in the white matter of the periventricular and subcortical regions of both hemispheres. There was no evidence for focal or diffuse contrast material intensities.
Ctyogenomic studies were performed on DNA extracted from a peripheral blood sample by using the 750 Kb SNP Affymertix Chip array. We detected complex results as follows: PAR1 (Xp22.33 or Yp11.31q11.23)X5, PAR2 (Xq28 or Yq12)X1, the other chromosomes showed normal copy number variations(CNVs). To understand the complex CNVs, G-banding karyotype and FISH studies (SRY, Cent X/Cen Y, and Sub telomere Specific X/Y) were performed. To sum up, the patient karyotype revealed a de novo 47,X,idic(Y)(pter → q12::q12 → pter)x2(SRYx4).
The uniqueness of our case is the presence of double abnormal isodicentric non-mosaic Y chromosomes. It is composed of the short arm and the proximal parts of the long arms of chromosome Y, in a living proband diagnosed with developmental delay and ASD.
The correlation of genotype-phenotype is still unknown and further cases and investigations are needed.

### 1.P6

### A case of an interstitial deletion of the long arm of chromosome 5

Valentyna Kurakova, Yuliya Shcherbak, Maryna Tsygankova, Sholpan Kulbalaeva, Vira Galagan
National Children Special Hospital "okhmatdet", The Center of Medical Genetics, Kyiv, Ukraine
**Correspondence:** Valentyna Kurakova
*Molecular Cytogenetics* 2017, **10(Suppl 1):**1.P6

Patients with various cardiac defects are often referred to The Center of Medical Genetics for consultation. It is known that heart defects may be related to chromosome syndromes. The patient (a girl, 4 months) was referred from The Center of Cardiology. Anamnesis: second pregnancy (the first - a healthy boy), birth weight 2400 g, length 48 cm. Prenatal ultrasound screening (at 26 weeks) showed a heart defect (membranous ventricular septal defect).
Phenotype: postnatal growth deficiency, heart defect (pulmonary artery stenosis, ductus arteriosus, ASD), cleft palate, ocular hypertelorism, arc-like eyebrows, thin lips, downturned corners of mouth. Absence of corpus callosum and ventricular dilatation were detected by MRI. Neurologic examination: retardation of psychomotor development, tube feeding.
The result of standard cytogenetic analysis (G-banding using trypsin; banding resolution of approximately 500): An interstitial deletion with breakage and reunion of bands 5q11.2 and 5q12. No microdeletion of 22q11.2 was detected using fluorescence in situ hybridization on locus 22q11.2 (HIRA). Karyotype: 46,XX, del(5)(q11.2q12)
Deletion of band 5q11.2 is considered to be a chromosomal syndrome. Several such clinical cases have been reported. Typically, the features of this syndrome are choanal atresia or stenosis, developmental delay, heart defects, external ear abnormalities and short stature. This rearrangement can also be found in patients who have been referred for microdeletion 22q11.2 and CHARGE syndrome, because the clinical presentations are similar.
Thus, children with congenital heart defects and development delay require cytogenetic testing with molecular cytogenetic diagnostic methods.
**Consent to publish:** The authors confirm that written informed consent was received by the patient for publication.

### 1.P7

### Unbalanced translocation (X;13) as a cause of two different phenotypes in siblings due to duplication versus haploinsuficiency of the SHOX gene and a skewed inactivation pattern

Katerina Adamova [1], Vaclava Curtisova [1], Marek Godava [1], Dita Cerna [2], Zuzana Dobsakova [3], Diana Grochova [3], Pavlina Capkova [1], Marcela Kvapilova [1], Hana Filipova [1]
[1]University Hospital And Palacky University, Department of Medical Genetics, Olomouc, Czech Republic; [2]University Hospital, Department of Medical Genetics, Ostrava, Czech Republic; [3]Cytogenetic Laboratory Brno, Laboratory of Molecular Genetics, Brno, Czech Republic
**Correspondence:** Katerina Adamova
*Molecular Cytogenetics* 2017, **10(Suppl 1):**1.P7

This abstract is not included here as it has already been published.

### 1.P8

### The Detection and Analysis of CNVs in Patients with Autistic Spectrum Disorders Using Various Strategies

Pavlina Capkova [1], Josef Srovnal [2], Zuzana Capkova [1], Vera Becvarova [3], Marie Trkova [3], Katerina Adamova [1], Sarka Vejvalkova [1], Radek Vodicka [1], Vaclava Curtisova [1]
[1]University Hospital Olomouc, Department of Medical Genetics, Olomouc, Czech Republic; [2]Faculty of Medicine And Dentistry, Palacky University, Institute of Molecular And Translational Medicine, Olomouc, Czech Republic; [3]Gennet, Centre For Fetal Medicine And Reproductive Genetics, Prague, Czech Republic
**Correspondence:** Pavlina Capkova
*Molecular Cytogenetics* 2017, **10(Suppl 1):**1.P8

**Background**
Autistic Spectrum Disorders (ASD) are heterogeneous and complex developmental diseases with a significant genetic background. Recurrent CNVs are known to be a frequent cause of ASD. Various platforms are used to detect pathogenic mutations in the genome of ASD patients.
Design: The performed study is focused on determination of the frequency of pathogenic mutations in these patients using MLPA, CMA and karyotyping.
**Methods**
The study involved 92 individuals with ASD predominantly from simplex families (89%). Systematic screening for pathogenic mutations involved karyotyping, screening for fragile X syndrome, screening for metabolic disorders, targeted MLPA test with probemixes Telomeres 3 and 5, Microdeletion 1 and 2, Autism 1, MRX (MRC, Holland) and chromosomal microarray analysis (CMA) (Illumina or Affymetrix).
**Results**
One chromosome aberration - r(22)- (1.08%) and FMR1 mutation (1.08%) were detected in the group of ASD patients. Pathogenic/likely pathogenic mutations were revealed in 16 (17.77%) ASD patients with normal karyotype (or balanced rearrangements) by CMA, in 9 (10%) by MLPA. More than 1 CNV (pathogenic/likely pathogenic/VOUS) were discovered in 3 patients. CMA detected 168 CNVs altogether - 86 rare CNVs (MAF < 1% , size above 10 kb and with at least 1 exon): 8 (4.76%) pathogenic, 12 (7.14%) likely pathogenic and 12 (7.14%) VOUS, 54 (32.14%) likely benign.
**Conclusion**
Concordat results were achieved in 58,8% patients with pathogenic/likely pathogenic findings - including r(22)- tested by MLPA and CMA. MLPA is useful as a cheap and quick tool for the targeted exclusion of recurrent CNVs in ASD patients. Despite the low detection rate (1.08%) karyotyping proved to be indispensable to discover the genesis of the aberration.
Supported by MH CZ – DRO (FNOL 00098892), IGA UP LF_2016_010.

### 1.P9
### Genetic profile of “macrozoopsermic” Tunisian patients
Wiem Ayed [1], Ihab Boukari [2], Wajih Hammami [2], Meriem Ben Khelifa [2], Hend Chaker [2], Yasmine El Ayeb [3], Chéma Triki [4], Meriem Chargui [5], Sonia Abdelhak [5], Ahlem Amouri [2]
[1]Institut Pasteur De Tunis, Faculté De Médecine De Tunis, Histology And Cytogenetics, Tunis, Tunisia; [2]Institut Pasteur De Tunis, Histology And Cytogenetics, Tunis, Tunisia; [3]Institut Pasteur De Tuis, Histology And Cytogenetics, Tunis, Tunisia; [4]Clinique Hannibal De Tunis, Unité De Procréation Médicale Assistée, Tunis, Tunisia; [5]Institut Pasteur De Tunis, Labortoire De Génomique Médicale Et Oncogénétique, Tunis, Tunisia
**Correspondence:** Wiem Ayed
*Molecular Cytogenetics* 2017, **10(Suppl 1):**1.P9

Infertility concerns a minimum of 70 million couples worldwide. An important proportion of cases are believed to have a genetic component, yet few causal genes have been identified so far. Teratospermia, a rare form of male infertility, is characterized by the presence of over 85% morphologically abnormal spermatozoa in sperm. Macrozoospermia is a very rare morphologic disorder of spermatozoa observed in less than 1% of infertile men. It can be defined as the presence of a very high percentage of spermatozoa with enlarged head, an irregular head shape, and multiple flagella. Meiotic segregation studies in 30 males revealed that over 90% of spermatozoa were aneuploid, mainly diploid. Different mutations in the AURKC gene were related to this sperm defect and two recurrent mutations (c.144delC and p.Y248*) were the most frequently described.
The aim of this study was to provide genetic characterization of fifteen Tunisian men with ‘macrozoospermia” refered to the Cytogenetic Department of Institut Pasteur de Tunis for infertility.
Molecular diagnosis was carried out for the AURKC gene in all the patients. Firstly, screening for recurrent mutations in exon 3 and 6 was performed by direct sequencing. Secondly, the whole AURKC gene was analyzed.
Direct sequencing of exon 3 revealed the presence of the c.144delC recurrent mutation at a homozygous state in 10 patients out of 15 (66%). In two other patients, the c.744C > G (p.Y248*) mutation in exon 6 was identified. The remaining five AURKC exons and their boundaries were then sequenced for the other three patients and no pathogenic variant was detected.
Our findings confirm that the recurrent deletion c.144delC is the most frequent mutation causing macrozoospermia in Tunisian patients. The prognosis for non-mutated men, normally with milder forms of the pathology, is more open. To better assess the reproductive potential of these patients, a FISH analysis should be performed on spermatozoa.
This diagnosis is important as the identification of AURKC mutations in patients indicates that all spermatozoa will be chromosomally abnormal and that intracytoplasmic sperm injection (ICSI) is very disappointing in this group.

### 1.P10
### Report of a new postnatal case with pentasomy X
Polina Verhovtsova [1], Priit Reedik [2], Airi Visamaa [1], Kairit Joost [1]
[1]Tartu University Hospital United Laboratories, Department of Clinical Genetics, Tallinn, Estonia; [2]Tallinn Children’s Hospital, Department of Neonatology, Tallinn, Estonia
**Correspondence:** Polina Verhovtsova
*Molecular Cytogenetics* 2017, **10(Suppl 1):**1.P10

Pentasomy X (49,XXXXX) is a very rare chromosomal abnormality with less than 30 cases described in medical literature. The disorder affects females and causes variable clinical problems with moderate to severe mental retardation and developmental delay being the most common features. The pathogenesis of pentasomy X is not clear at present, but it is thought to be caused by successive maternal or combined maternal and paternal meiotic nondisjunctions.
We report an infant born during the 36th gestational week who was referred for conventional cytogenetic analysis due to a dysmorphic phenotype. The infant’s birth weight was 2105 g (−1.8 SD), length was 47 cm (−1 SD) and head circumference was 31 cm (−1.5 SD). The patient had retrognathia, malformed ears, preauricular pits and bilateral clinodactyly of the fifth fingers. Cytogenetic analysis revealed that the karyotype of the patient was 49,XXXXX.
Conclusion. We report a case of pentasomy X in order to increase knowledge about this disorder because the rarity of the condition and few data available make genetic counselling complicated in terms of giving families detailed clinical information. Thus we consider it is very important to publish and collect data about all newly discovered pentasomy X cases.

### 1.P11
### Double two way reciprocal translocations in man with fertility failures A case presentation
Inguna Stalidzane [1], Silvija Ozolina [1], Egils Gasin [2], Didzis Gavars [3]
[1]E.gulbis Laboratory, Cytogenetic, Riga, Latvia; [2]Centre of Reproductive Medicin, Ginecology, Riga, Latvia; [3]E.gulbis Laboratory, Molecular Biology, Riga, Latvia
**Correspondence:** Inguna Stalidzane
*Molecular Cytogenetics* 2017, **10(Suppl 1):**1.P11

The occurrence of two independent reciprocal translocations in one person is extremely rare.
We report a case of a 27 years old phenotypically normal male referred for cytogenetic analysis with failed IVF treatments. His sperm test is normal with small displacements of sperm morphology.
The couple have had repeated spontaneous abortions in an early stage of pregnancy as well as two unsuccessful IVF procedures with the patient's semen. The karyotype of the female partner is normal: 46, XX. The patient's family have not been studied. It is known that the patient's sister also has reproductive problems.

Cytogenetic studies on G-banded metaphases indicated that the patient had an apparently four-way balanced complex translocation involving chromosomes 1, 3, 8 and 20 with at least four breakpoints.
Further investigation by fluorescence in situ hybridization (FISH) using whole chromosome paints, centromere probes, telomere probes as well as locus specific probes revealed two separate reciprocal translocations. The patient's karyotype was therefore: 46,XY,t(1;20)(p32.?1;q13.1),t(3;8)(q27;q21.2).
Investigation of the patient with routine analysis, standard karyotyping and FISH, is useful in such cases. Considering the complexity of preimplantation diagnosis in this case the couple agreed on an IVF procedure with the use of donor sperm. A pregnancy resulted in a birth of genetically normal twins.
**Consent to publish:** The authors confirm that written informed consent was received by the patient for publication.

### 1.P12

### MLPA analysis in a cohort of patients with intellectual disabilities

Ivana Skrlec [1], Mirna Andelic [1], Natasa Teran [2], Alenka Hodzic [2], Visnja Tomac [3], Sanela Stibi [1], Silvija Puseljic [3], Borut Peterlin [2], Jasenka Wagner [1]
[1]Faculty of Medicine, J. J. Strossmayer University of Osijek, Department of Medical Biology And Genetics, Osijek, Croatia; [2]University Medical Center Ljubljana, Clinical Institute of Medical Genetics, Ljubljana, Slovenia; [3]University Hospital Osijek, Pediatric Clinics, Osijek, Croatia
**Correspondence:** Ivana Skrlec
*Molecular Cytogenetics* 2017, **10(Suppl 1):**1.P12

Intellectual disability is a common neurodevelopment disorder which manifests as severely impaired cognitive development in early childhood and adolescence. In recent years advances in molecular cytogenetic and molecular genetics have shown that submicroscopic genome abnormalities are a frequent, until now under-recognized, cause of intellectual disability. The objectives of this study were to discover new genetic causes of intellectual disability using the MLPA method, to determine the prevalence of chromosomal aberrations detected using MLPA and the assessment of this technique as a screening method in the evaluation of clinically relevant and newly discovered variations in the number of copies of DNA associated with the intellectual disability.
We conducted a retrospective study of chromosomal changes in subtelomeric regions in patients with intellectual disabilities. 50 children (10 girls and 40 boys) participated in the study. They were referred for mental retardation/developmental delay and were evaluated at the Pediatric Clinics, University Hospital Osijek. Initial genetic testing that included karyotyping and molecular-genetic analysis for fragile X syndrome were performed. Since all patients had normal results of initial testing, MLPA testing using probes for the detection of subtelomeric changes (P036 and P070) was performed.
The study has shown pathological CNV changes in 5 patients (2 girls and 3 boys) or 10%. The changes were observed on chromosomes 15, 19, 21 and X, and are in accordance with the clinical features of the patients.
Our study has shown that the MLPA method is a useful screening method for patients with intellectual disability. The prevalence of chromosomal aberrations detected using this technique was higher than expected (10%), but this could be due to the small number of tested samples.

### 1.P13

### Interstitial deletion of the short arm of chromosome 8 in a girl with epilepsy and mild dysmorphic features

Andreja Zagorac [1], Andreja Osterc Koprivsek [2], Peter Gradisnik [2], Nadja Kokalj Vokac [1]
[1]University Medical Centre Maribor, Laboratory of Medical Genetics, Maribor, Slovenia; [2]University Medical Centre Maribor, Division of Paediatrics, Maribor, Slovenia
**Correspondence:** Andreja Zagorac
*Molecular Cytogenetics* 2017, **10(Suppl 1):**1.P13

Interstitial microdeletion and microduplication syndromes account for 50-60% of all submicroscopic chromosomal abnormalities detected in patients with intellectual disabilities and/or multiple congenital anomalies. In the literature, interstitial deletion 8p22 is reported in a small number of cases. In the majority of cases, the 8p22 deletion span into the subtelomeric region and is associated with a heterogeneous clinical phenotype.
We describe a 14-months old girl with a 1 Mb interstitial deletion of 8p22, born to non-consanguineous parents in a family negative for neurological diseases, malformations and developmental delay. From the 5th month of age she occasionally experienced brief unresponsiveness with hypotonia (focal complex epileptic seizures). At the age of 8 months she had one generalized tonic-clonic and two focal complex epileptic seizures. She presented mild dysmorphic features: down slanting eye fissures, epicantal folds, flat nasal bridge, low-set ears and only one groove of the right hand and mild axial hypotonia. Brain MRI showed presence of mild signal hyper intensity in T1 sequence subcortically, temporally and parietaly on both sides.
Array CGH analysis on peripheral blood using BlueGnome CytoChip Oligo 8x60K array and FISH analysis on cultured lymphocytes with BAC probes (RP11) were performed.
Array CGH revealed de novo 1 Mb interstitial deletion on the short arm of chromosome 8p22, including the genes SGCZ and microRNA 383: arr[GRCh37] 8p22(13981196–15000151)x1. Deletion was confirmed by FISH.
Cases with interstitial deletion of 8p22, including gene SGCZ and MIR383, present heterogeneous clinical phenotypes associated with neuropsychiatric disorders, mild intellectual disabilities, speech delay, slight psycomotor delay and minimal dysmorphic signs. The differences in the size of the deletion and the percentage of overlapping region of the deletion play an important role in manifested phenotype.
**Consent to publish:** The authors confirm that written informed consent was received by the patient for publication.

### 1.P14

### Microphthalmia with linear skin defects syndrome (MLS) in a girl with mosaic interstitial deletion of Xp22.2 22.31

Nadja Kokalj Vokač [1], Bojan Korpar [2], Andreja Zagorac [1], Boris Zagradinik [1]
[1]Laboratory of Medical Genetics, Universty Medical Centre Maribor, Maribor, Slovenia; [2]Department of Perinatology, Universty Medical Centre Maribor, Maribor, Slovenia
**Correspondence:** Nadja Kokalj Vokač
*Molecular Cytogenetics* 2017, **10(Suppl 1):**1.P14

Microphthalmia with linear skin defects is an rare neurodevelopmental X-dominant condition caused by heterozygous mutation or deletion of the critical HCCS gene in females and it is letal in males. A critical region for the MLS syndrome (OMIM 309801) has been defined to encompass the genes MID1, HCCS and ARGAP6. In our case, we describe a neonate with unilateral microphthalmia and facial skin lesions with a mosaic deletion of all three critical genes.
The girl was was born with elective cesarean section because of fetal paroxysosmal tachycardia. Clinical findings revealed anophthalmia of the left eye. The left eyelids were intact, the orbita was empty and the right eye was normal, without any abnormalities. She had typical linear skin defects on the left face, one on the left side of the neck, and two on the 3rd an 4th finger of the left hand. The other clinical findings and neurological exam where normal. Ultrasound of the brain and EEG were normal.
Molecular karyotyping using BlueGnome CytoChip Oligo 4x180K array was performed. To confirm the array results, FISH using RPCI11-768H20 BAC clone on cultivated lymphocytes was performed.
An approximatly 18% mosaic, 3,3 Mb deletion, was detected by Array-CGH. Mosaicism was confirmed by FISH in 29% of interphase and metaphase cells: 46,XX,ish del(p22.2p22.31)(RPCI11-768H20-)[60/205].arr[GRCh37] Xp22.31p22.2(8622553_11887361)x1[0.18].
Only two cases of mosaic MLS syndrome and only three cases of cryptic interstitial deletion covering HCCS gene have been described.

Our case support data that indicate the role of the HCCS gene in variable eye and skin abnormalities.

### 1.P15

### Identical phenotypes of inherited reciprocal chromosomal microdeletions and microduplications are explained by similar expression levels of genes affected by CNVs

Igor N Lebedev [1], Maria M Gridina [2], Alexei G Menzorov [2], Natalya M Matveeva [2], Inna E Pristyazhnyuk [2], Tatyana A Shnaider [2], Lyudmila P Nazarenko [3], Nikolay A Skryabin [1], Tatyana V Nikitina [1], Elena A Sazhenova [1], Anna A Kashevarova [1], Oleg L Serov [2]
[1]Research Institute of Medical Genetics, Tomsk National Research Medical Center, Russian Academy of Sciences, Laboratory of Cytogenetics, Tomsk, Russia; [2]Institute of Cytology And Genetics, Siberian Branch of Russian Academy of Sciences, Laboratory of Developmental Genetics, Novosibirsk Russia; [3]Research Institute of Medical Genetics, Tomsk National Research Medical Center, Russian Academy of Sciences, Laboratory of Hereditary Pathology, Tomsk, Russia
**Correspondence:** Igor N Lebedev
*Molecular Cytogenetics* 2017, **10(Suppl 1):**1.P15

Different genotype-phenotype correlations are considered for reciprocal CNVs. They include mirrored, identical, overlapping and unique phenotypes (Golzio, Katsanis, 2013). The understanding of the molecular bases of these relationships is strongly complicated by tissue-specific effects of chromosomal imbalances and the size of CNVs, usually involving multiple genes with different functions and complex interactions. Partially these problems can be overcome using current technologies of somatic cell reprogramming as well as high resolution molecular karyotyping (aCGH, SNP-arrays or NGS), allowing to identify submicroscopic chromosomal aberrations affecting a single gene. Here, we report the features of neuronal in vitro expression of CNTN6, which has been recently associated with autistic spectrum disorders and intellectual disability in a patient with 3p26.3 microduplication of paternal origin affecting CNTN6 only (Kashevarova et al., 2014). Neuronal cells were obtained by NGN2 differentiation of induced pluripotent stem cells (IPSCs) derived from skin fibroblasts from a patient with intellectual disability and dysmorphic features as well as from three healthy donors with normal karyotype. It was found, that expression of the duplicated allele of paternal origin was significantly reduced in spite of the increased CNTN6 copy number due to chromosomal microduplication. Moreover, the preferential expression of the maternal allele of CNTN6 was observed both in patients and donors neuronal cells derived from IPSCs. The obtained results support the reported mode of inheritance of chromosomal rearrangements affecting CNTN6 in several generations by parental origin of CNV (Moghadasi et al., 2014; Hu et al., 2015). In addition, they provide experimental evidence that some identical phenotypes in patients with reciprocal microdeletions and microduplications syndromes may be explained by haploinsufficiency due to a possible suppression effect of chromosomal microduplication at the gene expression level.

**Funding**
This study was supported by Russian Science Foundation, grant 14-15-00772.

### 1.P16

### 8q22.3 q24.23 duplication in a patient with oral frenulum and normal intellectual development

Hande Küçük Kurtulgan, Malik Ejder Yildirim, Burak Başer, Ilhan Sezgin
Cumhuriyet University, Medical Genetics, Sivas, Turkey
**Correspondence:** Hande Küçük Kurtulgan
*Molecular Cytogenetics* 2017, **10(Suppl 1):**1.P16

A 7-year-old girl was referred to our clinic for hypertrichosis and dysmorphic facial appearance. On physical examination, hypertrichosis, upslanted palpebral fissures, epicanthus, hypertelorism, microretrognathia, high and broad nasal root, distinct glabella, fine upper lip, broad and flat philtrum and clinodactyly were detected. The height, weight and head circumference of the patient was 128 cm, 2700 gr and 50 cm, respectively. She had a history of an operation for oral frenulum. Haemogram, routine biochemistry, hormone profiles, karyotype analysis and brain magnetic resonance imaging (MRI) and ophtalmology, otolaryngology, child psychiatry consultations were requested.
Haemogram, routine biochemistry, hormone profiles, brain MRI results and the ophthalmologic evaluation was normal. Chronic otitis media was detected on otolaryngologic examination. IQ test reported as 95. Chromosome analysis revealed a 46,XX,der(8)-add(8)(q24.1) karyotype. Karyotypes of mother, father and sister were normal. aCGH was done to determine where the extra material came from. A duplication of 35.9 Mb at 8q22.3-q24.23 was detected.
Our case had similar phenotypic features with 8q duplication cases, such as hypertrichosis, hypertelorism, microretrognati and long philtrum. But, to our knowledge, this is the first case of 8q duplication with oral frenulum and without intellectual disability.
**Consent to publish:** The authors confirm that written informed consent was received by the patient for publication.

### 1.P17

### Complex chromosomal rearrangement in an infertile male patient with cryptozoospermia

Albrecht Röpke [1], Yvonne Stratis [1], Judit Horvath [1], Frank Tüttelmann [1], Claudia Krallmann [2], Sabine Kliesch [2], Peter Wieacker [1]
[1] University Hospital Münster, Institute of Human Genetics, Münster, Germany; [2]University Hospital Münster, Department of Clinical And Surgical Andrology, Centre of Reproductive Medicine And Andrology, Münster, Germany
**Correspondence:** Albrecht Röpke
*Molecular Cytogenetics* 2017, **10(Suppl 1):**1.P17

Familial cases of complex chromosomal rearrangements (CCR) have rarely been described in the literature. Carriers of familial CCRs have a higher risk for miscarriage due to either malsegregation of the derivative chromosomes or generation of recombinant chromosomes. However, men carrying CCRs seem to be more often infertile than women, since spermatogenesis is more susceptible to chromosomal aberrations than female gametogenesis.
We report on an infertile male patient, who was referred to our andrology centre with a history of primary infertility. The patient had cryptozoospermia in several semen analyses, i.e. only very few spermatozoa could be identified after centrifugation. Hormone analysis repeatedly showed normal LH and FSH levels, but slightly reduced testosterone serum values.
Cytogenetic analysis including several FISH demonstrated a complex karyotype involving four chromosomal breakpoints on chromosome 1p22.1, 9p22 (two times) and 13q14.3. Chromosomal material of 9p22- > 9pter was found distal to 1p22.1, and the material of 13q14.3- > 13qter at 9p22. The derivative chromosome 13 showed distal to 13q14.3 a small insertion of material from 9p22 followed by chromosomal material of 1p22.1- > 1pter. This karyotype demonstrated two breakpoints in 9p22 with a distal breakpoint for the translocation to 1p22.1 and a second, more proximal breakpoint for the translocation to 13q14.3. The same karyotype was also found in the patient's mother.
After genetic counselling the couple decided for in vitro fertilization including intracytoplasmic sperm injection. Thus far, one attempt was made without a successful pregnancy.
In conclusion, we report on a rare maternally inherited CCR found in an infertile male patient. The meiotic problems in the formation of the putative hexavalent might well be the cause for the severely reduced sperm count.

### 1.P18

### The First Clinical CNV Database in Russia

Ilya Kanivets, Sergey Korostelev, Denis Pyankov, Anna Antonets, Fedor Konovalov, Philip Koshkin, Alena Shilova, Elena Aksenova
[1]Genomed Ltd, Genetic Diagnostic Lab, Moscow, Russia
**Correspondence:** Ilya Kanivets
*Molecular Cytogenetics* 2017, **10(Suppl 1):**1.P18

In Russia, the chromosomal microarray analysis (CMA) for clinical purposes is performed mostly by private laboratories. We use CMA for postnatal diagnosis in children with multiple congenital anomalies (MCA), developmental delay (DD) and dysmorphic facial features (DF), for prenatal diagnosis if chromosomal anomalies in fetus are suspected, and for testing products of conception and FFPE tumor samples.
Over the course of 5 years, we created the largest CNVs database in Russia that includes information on 6155 samples tested in our laboratory. Implementing this database not only solved the problem of storing CNVs data in a standardized form, but also made it possible to classify CNVs by type, size, gene content, pathogenicity, major clinical features in patient, the kind of the sample and the array type. In particular, we evaluated the prevalence of microdeletion syndromes and pathogenic CNVs in different patient groups. In a group of 3299 patients with MCA, DD and DF, pathogenic CNVs were detected in 565 cases (17.1%).
The database contains information on CNVs detected mainly in Russian populations, and it is used to determine the clinical significance of CNVs previously not described in other databases, e.g. DGV or DECIPHER, and as an additional tool for evaluation of pathogenicity of all other CNVs.

### 1.P19

### SIGU (Italian Society of Human Genetics) Cyto Genetics Genomics work group a study including 5059 patients referred for postnatal CMA (Chromosomal Microarray) analysis

Ilaria Catusi [1], Maria Paola Recalcati [1], Melissa Alfonsi [2], Alberta Alghisi [3], Stefania Cappellani [4], Rosario Casalone [5], Rossella Caselli [6], Caterina Ceccarini [7], Carlo Ceglia [8], Anna Maria Ciaschini [9], Domenico Coviello [10], Francesca Crosti [11], Annamaria D'aprile [7], Antonella Fabretto [4], Maria Garzo [1], Rita Genesio [12], Marzia Giagnacovo [13], Paola Granata [5], Ilaria Longo [6], Michela Malacarne [10], Giuseppina Marseglia [14], Annamaria Montaldi [3], Anna Maria Nardone [15], Chiara Palka [16], Vanna Pecile [4], Chiara Pessina [5], Diana Postorivo [15], Serena Redaelli [17], Alessandra Renieri [6], Chiara Rigon [18], Fabiola Tiberi [9], Mariella Tonelli [19], Chiara Valtorta [1], Nicoletta Villa [11], Anna Zilio [3], Daniela Zuccarello [18], Antonio Novelli [20], Lidia Larizza [1], Daniela Giardino [1]
[1]Irccs Istituto Auxologico Italiano, Lab. Di Citogenetica Medica E Genetica Molecolare, Milano, Italy; [2]Ospedale Ss Annunziata, U.o.c. Di Genetica Medica, Chieti, Italy; [3]Azienda Ulss 6, U.o.s. Genetica E Biologia Molecolare, Vicenza, Italy; [4]Irccs Burlo Garofolo, S.c. Genetica Medica, Trieste, Italy; [5]Asst Sette Laghi, Osp. Di Circolo E Fond. Macchi, Smel Specializzato Citogenetica E Genetica Medica, Varese, Italy; [6]Azienda Ospedaliera Universitaria Senese, U.o.c. Genetica Medica, Siena, Italy; [7]A.o.u. Ospedali Riuniti, Lab. Di Citogenetica, Foggia, Italy; [8]Aorn Sg Moscati, Uosd Genetica Medica, Avellino, Italy; [9]A.o.u. Ospedali Riuniti Umberto I-g.m.lancisi-g.salesi, Lab. Genetica Medica Sos Malattie Rare, Ancona, Italy; [10]E.o. Ospedali Galliera, Lab. Di Genetica Umana, Genova, Italy; [11]Ospedale San Gerardo - Asst Monza, U.s. Genetica Medica, Monza, Italy; [12]A.o.u. Federico Ii, U.o.c. Di Citogenetica, Napoli, Italy; [13]Asst Lariana - Ospedale Sant' Anna, Lab. Di Genetica, Como, Italy; [14]A.o.u. Careggi, S.o.d. Diagnostica Genetica, Firenze, Italy; [15]Policlinico Tor Vergata, U.o.c. Laboratorio Di Genetica Medica, Roma, Italy; [16]Università G. D'annunzio, Dipartimento Di Pediatria, Chieti-pescara, Italy; [17]Università Di Milano-bicocca, Dipartimento Di Medicina E Chirurgia, Monza, Italy; [18]A.o.u. Di Padova, U.o.c. Genetica E Epidemiologia Clinica, Padova, Italy; [19]Università Di Brescia, Lcgm Dipartimento Di Medicina Molecolare E Traslazionale, Brescia, Italy; [20]Ospedale Pediatrico Del Bambino Gesu', U.o.c. Laboratorio Di Genetica Medica, Roma, Italy
**Correspondence:** Ilaria Catusi
*Molecular Cytogenetics* 2017, **10(Suppl 1):**1.P19

Here, we present a study coordinated by the SIGU Cyto-Genetics/-Genomics work group, which includes Chromosomal Microarray (CMA) results from 5059 patients, originating from 17 primary referral sites in Italy.
All individuals had been referred for CMA analysis due to isolated or variably combined intellectual disability (ID), developmental delay (DD), autism spectrum disorder (ASD), congenital malformation(s) (M) and dysmorphic features (D).
Overall, 1846 non-polymorphic copy number variations (CNVs) were identified in 1479 individuals (29%), including 60% males and 40% females. CNVs were classified as pathogenic (pCNVs) in 39% of cases, corresponding to 557 individuals (11%), and in the remaining 61% as variants of uncertain clinical significance (VOUS). The latter group of CNVs included VOUS-likely pathogenic (10%), VOUS-likely benign (11%) and VOUS-no classification (40%) subgroups.
Out of all CNVs detected, duplications and deletions were equally represented, while out of pCNVs, deletions accounted for 63% of imbalances. The predominant chromosomes carrying pCNVs were chromosomes 15, 16, 17 and 22.
Considering clinical categories with at least 100 individuals, the higher diagnostic yield for pCNVs was found in patients with complex clinical phenotypes suggestive of a microdeletion/-duplication syndrome (24%), ID and M (19%), ID and D (19%), DD and D (14%) and isolated D (12%).
This study, surveying the largest Italian cohort of individuals referred for CMA analysis so far, confirms CMA as a first-tier analysis for individuals referred for major congenital anomalies (D/M) with or without ID/DD.

### 1.P20

### The role of classical cytogenetics for detection of autosomal structural changes after birth in the Romanian population

Mariela Sanda Militaru [1], Mihai Militaru [2], Alexandra Maris [3], Maria Stefanut [4], Iulia Blanaru [4], Diana Militaru [5], Eleonora Dronca [1]
[1]Umf "iuliu Hatieganu", Medical Genetics, Cluj-napoca, Romania; [2]Umf "iuliu Hatieganu", Pediatrics No.2, Cluj-napoca, Romania; [3]Children's Hospital, Intensive Care Unit, Cluj-napoca, Romania; [4]Genetic Center, Genetics, Cluj-napoca, Romania; [5]Faculty of Medicine, Student, Oradea, Romania
**Correspondence:** Mariela Sanda Militaru
*Molecular Cytogenetics* 2017, **10(Suppl 1):**1.P20

This abstract is not included here as it has already been published.

### 1.P21

### Strategy of identification and characterization of supernumerary marker chromosomes (SMC) a study about 29 cases

Wafa Slimani [1], Molka Kammoun [1], Hanene Hannachi [1], Afef Jalloul [1], Hayet Ben Hmida [2], Zohra Chioukh [2], Adnène Mlika [3], Mohamed Bibi [4], Thomas Liehr [5], Ali Saad [1], Soumaya Mougou-Zerelli [1]
[1]Laboratory of Human Cytogenetics, Molecular Genetics And Biology of Reproduction, Farhat Hached University Hospital, Human Cytogenetics, Sousse, Tunisia; [2]Hospital of Fattouma Bourguiba, Department of Neonatology, Monastir, Tunisia; [3]Farhat Hached University Hospital, Pediatric Department, Sousse, Tunisia; [4]Farhat Hached University Hospital, Department of Gynecology, Sousse, Tunisia; [5]Institute of Human Genetics, Human Genetics, Jena, Germany
**Correspondence:** Soumaya Mougou-Zerelli
*Molecular Cytogenetics* 2017, **10(Suppl 1):**1.P21

Twenty nine patients with SMC, including 24 children with different indications, 4 men and a woman with subfertility, were investigated systematically with R-banding. Based on the morphology of the SMC, the banding and the clinical indications, fluorescent in Situ hybridization (FISH) and/or array comparative genomic hybridization (array CGH) were performed for the characterization of the marker. Combining all techniques, we were able to identify and characterize eight types of SMC in 24 children and three different types in the subfertile population.

In the first group of 24 children, the SMC had originated from chromosomes 15 (7 cases), 18 (isochromosome 18p, 7 cases), a translocation t(11;22) (3 cases), chromosome 9 (2cases), chromosome 22 (2cases), and one case each from the centromeric region of chromosome 13 or 21 (1case), a translocation t(4;21) and a translocation t(5;21). In the subfertile group, we identified one SMC from chromosome 15, one derivative from the X chromosome and one derivative from chromosome 22.

Clinical data, conventional karyotyping and parental investigation allowed the orientation of the strategy for the identification of the marker.

Using FISH and Array CGH we were able to identify and characterize 28 out of 29 markers. Unlike previous studies where SMC 15 is the most frequent followed by SMC 22, in our population SMC derived from chromosomes 18 and 15 are the most frequent.

Subfertile people are usually phenotypically healthy but the presence of SMC probably disrupts the process of gametogenesis during meiosis.

### 1.P22

### Use of multiple chromosomal and molecular analyses in the elucidation of the etiology of multiple congenital anomalies

Mihaela Grămescu, Lavinia Caba, Monica Pânzaru, Lăcrămioara Butnariu, Elena Braha, Roxana Popescu, Setalia Popa, Cristina Rusu, Georgeta Cardos, Monica Zeleniuc, Violeta Martiniuc, Eusebiu Gorduza
Personal Genetic Laboratory, Medical Genetics, Iasi, Romania
**Correspondence:** Eusebiu Gorduza
*Molecular Cytogenetics* 2017, **10(Suppl 1):**1.P22

In 2015 and 2016, the geneticists of „Grigore T. Popa" Medicine and Pharmacy University Iaşi, evaluated 1950 patients with genetic/malformation disorders. In 335 patients (17.1%) karyotyping using GTG banding was performed. The majority of the patients had a classical chromosome disorders (Down syndrome – 95 cases [89 confirmed]; Turner syndrome – 39 cases [31 confirmed] or Klinefelter syndrome – 6 cases [4 confirmed]). In patients presenting with multiple congenital anomalies (107 cases) or a craniofacial dysmorphic features (88 cases) we identified 25 with an unbalanced chromosomal anomaly. In 14 patients with a normal karyotype, but a phenotype specific for a micro deletion syndrome, we confirmed the specific anomaly by FISH or/and MLPA (4 cases of velocardiofacial syndrome, 2 cases of Wolf-Hirschhorn syndrome, 3 cases of Prader-Willi syndrome, 2 cases of cri-du-chat syndrome, 2 cases of Williams syndrome and 1 case of Miller-Dieker syndrome). In 11 patients we applied array-CGH with Sure Print G3 ISCA V2 CGH 8x60K Array Kit (Agilent Technologies), NimbleGen MS 200 Microarray Scanner and NimbleGen MS 200 Software v1.1 and we found a genomic anomaly in 10 of these. The sequential use of different types of targeted chromosomal/molecular analyses (karyotype, FISH, MLPA, array-CGH) allowed us to certify the diagnosis in 163 of 335 patients (48.65%). This study was supported by founding of PN-II-PT-PCCA-2013-4-133 Program of UEFISCDI (National Romanian organism of research).

### 1.P23

### Complex approach to diagnosis of the familial insertional translocation (2;7)

Marina Minzhenkova [1], Zhanna Markova [1], Yulia Kozlova [1], Elena Dadali [2], Nadezda Shilova [1]
[1]Research Centre For Medical Genetics, Laboratory of Cytogenetics, Moscow, Russia; [2]Research Centre For Medical Genetics, Department of Genetic Counceling, Moscow, Russia
**Correspondence:** Marina Minzhenkova
*Molecular Cytogenetics* 2017, **10(Suppl 1):**1.P23

Insertional translocations (ITs) are relatively rare rearrangements, occurring with estimated frequency 1:5000 newborns, and more recent studies increased estimate of about 1:500. We report a familial case of IT.

aCGH using chromosomal microarray (CMA) revealed an ~16 Mb copy number gain of chromosome 7q31.33q34 material. Patient at age 18 months was referred for evaluation because of developmental delay, seizures of unknown etiology and slight dysmorphic features. Previously performed karyotype was normal. FISH analyses using WCP7 probe revealed that segment of chromosome 7 is inserted into chromosome 2. The size of inserted region measures 0.56% of the haploid autosomal length (HAL), which duplicated. Cytogenetic studies were initially performed on the proband's father and mother. A balanced insertion of chromosome 7 material (band q31.33 to q34) into chromosome 2 at band q35 was found in the father. mBAND 7 supported the G-band interpretation of an direct insertion of material of chromosome 7 into chromosome 2. Combined with these studies proband's karyotype was determined as 46,XY,der(2)ins(2;7)(q35;q31.33q34)pat. Additional relatives were studied. Cytogenetic studies previously performed on the proband's aunt at age 6 years because of mild developmental delay, short stature and slight dysmorphic features, showed a normal karyotype. At age 40 years aCGH revealed the same copy number gain of chromosome 7q31.33q34 material. FISH using WCP probes confirmed unbalanced IT. The proband`s grandmother was found to carry the balanced interchromosomal insertion: 46,XX,ins(2;7)(q35;q31.33q34). Thus two members of this family are duplicated for the region 7q31.33q34 due to unbalanced segregation of the insertion in two generations. Considering the small size of this insertional segment there is a high survivability of the conceptuses. The risk for recurrence in this family is high. In this way only complex approach allows to perform a complete quality diagnosis.

### 1.P24

### Cryptic microdeletion of 4q21 associated with a complex chromosome rearrangement clinical and molecular analysis based on a new case

Marlena Młynek, Marzena Kucharczyk, Małgorzata Krajewska-Walasek
Children's Memorial Health Institute, Department of Medical Genetics, Warsaw, Poland
**Correspondence:** Marlena Młynek
*Molecular Cytogenetics* 2017, **10(Suppl 1):**1.P24

The 4q21 microdeletion syndrome (MIM: 613509) is an emerging genomic disorder manifested as neonatal hypotonia, intellectual disability, absent or severely delayed speech, marked progressive growth retardation, variable brain malformation, and facial dysmorphism. To date, about 50 cases have been clinically and molecularly characterized. Although the boundaries and sizes of the 4q21 region deletions were different, a 1.37-Mb critical interval containing 5 genes (PRKG2, RASGEF1B, HNRNPD, HNRNPDL, and ENOPH1) was defined; these genes are considered to be the most promising candidates.

Here, we report on the case of a 17-year-old girl referred for genetic counseling because of psychomotor retardation, speech delay and subtle dysmorphic features (frontal bossing, broad forehead, hypertelorism, micrognathia). The pregnancy was complicated by intrauterine fetal hypotrophy. In the neonatal period, the patient presented with hypotonia, feeding problems and poor weight gain. At the clinical examination low body mass, joint laxity, hypertonia, psychomotor hyperactivity and seizures were noted.

Conventional G-banding karyotyping revealed a de novo complex chromosome rearrangement (CCR) involving three chromosomes (2;4;11)(p12;q21;p11.1). Whole-genome oligonucleotide microarray analysis revealed a 7.99-Mb cryptic interstitial deletion of the

4q13.3q21.21 region associated with the translocation breakpoint (chr4:73056485–81046914; GRCh37).
The phenotypic and genetic findings of our patient will be compared with those of previously reported patients. Although the deletion in the presented girl encompasses genes located more proximally, not included in the critical region for the 4q21 microdeletion syndrome, our proposita presents some of the common features described in the patients with this disorder. We indicate several candidate genes (SCARB2, FRAS1, PAQR3, ANTXR2) within the deleted region and define their role in the clinical manifestations of the syndrome, providing new data supporting further genotype-phenotype studies.
This study was supported by the NCN Grant No. 2016/21/B/NZ5/02541.
**Consent to publish:** The authors confirm that written informed consent was received by the patient for publication.

### 1.P25

### Phelan McDermid (22q13.3 microdeletion) syndrome a case report

Cristina Candeias [1], Manuela Mota Freitas [1], Natália Oliva-Teles [1], Joana Melo [2], Isabel Carreira [2], Teresa Saraiva [3], Ana Fortuna [4], Maria João Sá [4], Maria Da Luz Fonseca E Silva [5]
[1]Centro De Genética Médica Doutor Jacinto Magalhães, Centro Hospitalar Do Porto, Epe, Unidade De Citogenética; Unidade Multidisciplinar De Investigação Biomédica, Porto, Portugal; [2]Faculdade De Medicina Da Universidade De Coimbra, Laboratório De Citogenética E Genómica, Coimbra, Portugal; [3]Centro De Genética Médica Doutor Jacinto Magalhães, Centro Hospitalar Do Porto, Epe, Serviço De Genética Médica, Porto, Portugal; [4]Centro De Genética Médica Doutor Jacinto Magalhães, Centro Hospitalar Do Porto, Epe, Serviço De Genética Médica; Unidade Multidisciplinar De Investigação Biomédica, Porto, Portugal; [5]Centro De Genética Médica Doutor Jacinto Magalhães, Centro Hospitalar Do Porto, Epe, Unidade De Citogenética, Porto, Portugal
**Correspondence:** Cristina Candeias
*Molecular Cytogenetics* 2017, **10(Suppl 1):**1.P25

Phelan McDermid or 22q13.3 deletion syndrome (MIM: #606232) is a rare genetic disorder characterized by global developmental delay, cognitive deficits and behaviour in the autism spectrum. The mental retardation and developmental delay of these patients are mainly caused by deletions of the SHANK3 gene.
Conventional chromosomal analysis is recommended for evaluation of congenital anomalies and developmental delay but it may not be suitable for detecting microdeletions. Therefore, complementary molecular cytogenetics techniques such as fluorescence in situ hybridization (FISH), Multiplex ligation-dependent probe amplification (MLPA) or array comparative genomic hybridization (arrayCGH) may be useful methods to detect these microdeletions.
The authors present a 7-year-old girl with the clinical information of learning disabilities and behaviour disturbances. MLPA using Salsa® MLPA® Kits P036-E2, P70-B3, P245-B2 and P343-C2 (MRC, Holland, The Netherlands) were performed. These studies revealed a deletion in 22q13.3, involving the RABL2B gene and partial SHANK3 gene. Both parents had normal results in all these studies. ArrayCGH was requested in order to clarify these results. An interstitial deletion in 22q13.33, with an initial breakpoint in intron 9 of SHANK3 and a final breakpoint in an intergenic region, approximately 70 Kb, was observed.
Phelan McDermid is a complex and very heterogeneous syndrome. Although still considered rare, with the advance of genetic tests, namely MLPA and arrayCGH, it is likely that more cases will be identified. Karyotyping should be performed to exclude the presence of structural chromosomal anomalies, such as ring chromosome 22 and translocations involving this chromosome. Parents should always be tested in order to achieve the best genetic counselling for these families.
**Consent to publish:** The authors confirm that written informed consent was received by the patient for publication.

### 1.P26

### Cryptic copy number variations in a cohort of men with non obstructive azoospermia by custom designed high density array CGH

Aurélie Mouka [1], Loïc Drevillon [2], Manon Benabides [3], Vincent Izard [4], Didier Goidin [5], Lucie Tosca [1], Gérard Tachdjian[1]
D'histologie, Embryologie Et Cytogénétique, Clamart, France; [2]Ap-hp / Hôpitaux Universitaires Paris-sud, Hôpital Antoine Béclère, Service D'histologie, Embryologie Et Cytogénétique, Clamart, France; [3]Université De Lorraine, Campus Aiguillettes, Vandœuvre-lès-nancy, France; [4]Ap-hp, Hôpitaux Universitaires Paris-sud, Hôpital Antoine Béclère, Service De Gynécologie-obstétrique Et Médecine De La Reproduction, Clamart, France; [5]Agilent Technologies France, Parc Technopolis – Za Courtaboeuf, Les Ulis Cedex, France
**Correspondence:** Aurélie Mouka
*Molecular Cytogenetics* 2017, **10(Suppl 1):**1.P26

Male infertility accounts for half of couples with reproductive failure. In about 30% of them the etiology of spermatogenetic failure remains unexplained. Genetic routine investigations for severe male infertility include standard karyotype and AZF regions analysis. However, the impact of DNA copy number variations (CNVs) detected by array-based comparative genomic hybridization (array-CGH) is poorly documented. The aim of the present study was to test a custom-designed array-CGH in a cohort of patients with non-obstructive azoospermia (NOA) in order to screen DNA unbalances associated with spermatogenesis failure.
We report the use of a high resolution Agilent 400 K custom designed array-CGH to analyze a cohort of 50 men with NOA. Standard karyotype and AZF microdeletion analyses were normal. Custom array-CGH targeted a wide panel of 450 infertility-related genes. These genes are well known to be associated with male infertility or are strong candidate as published in mice. We enriched the microarray with oligonucleotide probes positioned in exons, introns as well as 5' and 3' regulated regions. Unenriched regions of the genome were covered by backbone probes to maintain a pangenomic analyze. We identified CNVs of interest that can be correlated to reproductive function within the cohort. It provided a relevant number of candidate genes like APOB or ZFPM2 which could represent novel targets for future research investigations. In addition, it also improved chromosome Y screening as we were able to detect in one patient a large microdeletion of 2 Mb in the AZFc region that was not identified by molecular genetic diagnosis.
We conclude that the use of a customized array-CGH would be suited for use in routine diagnosis and thus genetic counseling of male infertility issues and would provide new insight into the genetic basis of infertility.

### 1.P27

### A patient with a ring chromosome 14 syndrome involving adjacent 14q32.33 microduplication and microdeletion a case report

Pille Tammur [1], Klari Noormets [2], Piret Ilisson [1], Kati Kuuse [1], Sander Pajusalu [1], Maria Yakoreva [1], Tiia Reimand [1]
[1]United Laboratories, Tartu University Hospital, Department of Clinical Genetics, Tartu, Estonia; [2]Tartu University Hospital, Estonia, Children's Clinic, Tartu, Estonia
**Correspondence:** Pille Tammur
*Molecular Cytogenetics* 2017, **10(Suppl 1):**1.P27

The ring chromosome 14 is a rare cytogenetic aberration causing the r(14) syndrome which is characterized by early-onset epilepsy, developmental delay, microcephaly, and dysmorphic facial features.
Here we report on a 16-month-old boy who presented at 11 months of age with epileptic encephalopathy like clinical features: epileptic status, very frequent intractable seizures and progressive psychomotor impairment. He was born from uneventful pregnancy and delivery, but had feeding difficulties and failure to thrive in infancy. A

microcephaly, speech delay and some minor dysmorphic features were also noticed.
To investigate monogenic causes for epilepsy (presenting as epileptic encephalopathy) a next generation sequencing (NGS) of 4813 genes associated with monogenic disorders was performed using TruSight One panel (Illumina). No disease causing point mutations were detected, but the read-depth analysis of NGS alignments indicated a possible distal duplication of chromosome 14 (chr14:104,174,847-qter). To clarify the finding chromosomal microarray analysis was performed (HumanCytoSNP-12, Illumina) and two adjacent copy number variants were detected in chromosome 14: a 1.7 Mb duplication and 351 kb terminal deletion, both in the band 14q32.33. At this point r(14) was suspected, and later confirmed by routine GTG-banding. As the parents did not carry the chromosomal aberration the patient's karyotype was concluded as 46,XY,r(14)(p12q32)dn.
In conclusion, this case illustrates the necessity for integrating molecular and cytogenetic diagnostics to clarify the genetic aetiology of genetic disorders. Large structural chromosomal aberrations should be continuously included into differential diagnosis of epilepsy syndromes.
**Consent to publish:** The authors confirm that written informed consent was received by the patient for publication.

### 1.P28

### 16p11.2 Deletion Syndrome in Prenatal Diagnosis – a rare case

Manuela Mota Freitas [1], Cristina Candeias [1], Sílvia Pires [2], Natália Oliva-Teles [1], Purificação Tavares [3], Maria Do Céu Rodrigues [4], Maria Da Luz Fonseca E Silva [2]
[1]Centro De Genética Médica Doutor Jacinto Magalhães, Centro Hospitalar Do Porto, Epe, Unidade De Citogenética; Unidade Multidisciplinar De Investigação Biomédica, Porto, Portugal; [2]Centro De Genética Médica Doutor Jacinto Magalhães, Centro Hospitalar Do Porto, Epe, Unidade De Citogenética, Porto, Portugal; [3]Centro De Genética Clínica, Laboratório De Citogenética, Porto, Portugal; [4]Centro Materno Infantil Do Norte, Centro Hospitalar Do Porto, Epe, Consulta De Diagnóstico Pré-natal, Porto, Portugal
**Correspondence:** Manuela Mota Freitas
*Molecular Cytogenetics* 2017, **10(Suppl 1):**1.P28

A microdeletion in 16p11.2 represents a rare syndrome and an even rarer occurrence in prenatal diagnosis. In individuals presenting with delayed language development and abnormal speech articulation, learning difficulties/intellectual disability, social impairments with or without a diagnosis of autism spectrum disorder (ASD), macrocephaly and vertebral anomalies, this syndrome should always be considered.
The authors describe a case with a deletion of the short arm of chromosome 16, in 16p11.2, detected at prenatal diagnosis. A woman was referred for prenatal diagnosis at 21 weeks of gestation due to fetal malformations, several hemivertebras and single umbilical artery. Chromosome analysis was performed on GTL banded metaphases obtained from cultured amniocytes, with a resolution level of 400 bands. Molecular studies included array CGH and Multiplex ligation-dependent probe amplification (MLPA), using Salsa® MLPA® Kit P343 (MRC, Holland, The Netherlands).
The karyotype revealed an apparently normal female karyotype. Array CGH identified a 4,630 Mbp deletion on chromosome 16p11.2, which includes 138 genes. The pregnancy was terminated and the chromosome 16 microdeletion was confirmed in fetal skin by MLPA. Since parental studies were normal, this anomaly was considered "de novo".
The clinical features of this fetus will be compared with previously reported cases with the same microdeletion. The authors enhance the importance of multidisciplinary team discussions in prenatal diagnosis cases and thorough genomic analysis, particularly when fetal malformations are detected during echography evaluations.

### 1.P29

### RPS6KA3 duplication in a male child with severe intellectual disability

Sílvia Serafim [1], Bárbara Marques [1], Cristina Ferreira [1], Filomena Brito [1], Marisa Silva [1], Laurentino Simão [1], Cristina Alves [1], Sónia Pedro [1], Joaquim Sá [2], Hildeberto Correia [1]
[1]Instituto Nacional De Saúde Doutor Ricardo Jorge, I.p., Departamento De Genética Humana, Lisboa, Portugal; [2]Centro Hospitalar Do Algarve, Consulta De Aconselhamento Genético, Faro, Portugal
**Correspondence:** Sílvia Serafim
*Molecular Cytogenetics* 2017, **10(Suppl 1):**1.P29

Rare inherited and de novo copy number variations (CNVs) are the cause of a variety of genetic disorders with intellectual disability (ID). Chromosomal microarray analysis (CMA) has been a rapid method to identify both large and small pathogenic genomic imbalances causing those disorders.
The identification and classification of a CNV as pathogenic is not always easy to establish. For deletions or loss of function of specific genes, the likelihood of being causal is higher, and for duplications or overexpression for the same genes, the classification is harder.
Here we present a male child with severe ID and a family history with a female sibling presenting mild ID. Affymetrix Cytoscan HD CMA identified a gain of 530 Kb on Xp22.12 (chrX: 20016145_20546410 [GRCh37])) encompassing EIF1AX and RPS6KA3 genes. Inheritance was not yet possible to assess.
Mutations in the RPS6KA3 gene causes Coffin-Lowry syndrome, a mental retardation syndrome with dysmorphic facial features and skeletal anomalies. X-linked mental retardation-19 is a nonsyndromic form of mild to moderate ID also caused by mutations in RPS6KA3. Carrier females may be mildly affected. This shows that phenotypic variability may be expected for the loss of function of this gene.
Recently both familial and isolated cases have been reported with a duplication of the entire RPS6KA3 gene associated with mild to moderate ID. These few cases suggest that increased dosage of RPS6KA3 may be the cause for ID in these patients and gives additional information on genotype-phenotype correlation of imbalances regarding this gene.
The collection of more cases with duplication of RPS6KA3, as the one described here, will help to establish a better classification for these CNVs, as well as help to better predict the phenotypic consequences and facilitate subsequent genetic counseling in the families.

### 1.P30

### The Enigma of X Inactivation In Small Ring X Chromosomes phenotype In a XIST positive Case of a mos 46,X,r(X)/45,X

Fernanda Paula Oliveira [1], Maria Do Céu Ribeiro [1], Manuela Mota Freitas [2], Natália Oliva-Teles [2], Sílvia Álvares [3], Maria Da Luz Fonseca E Silva [1]
[1]Centro De Genética Médica Doutor Jacinto Magalhães, Centro Hospitalar Do Porto, Epe, Unidade De Citogenética, Porto, Portugal; [2]Centro De Genética Médica Doutor Jacinto Magalhães, Centro Hospitalar Do Porto, Epe; Unidade Multidisciplinar De Investigação Biomédica, Unidade De Citogenética, Porto, Portugal; [3]Centro Materno-infantil Do Norte, Centro Hospitalar Do Porto, Epe; Unidade Multidisciplinar De Investigação Biomédica, Departamento Da Mulher E Da Criança, Porto, Portugal
**Correspondence:** Fernanda Paula Oliveira
*Molecular Cytogenetics* 2017, **10(Suppl 1):**1.P30

Mosaic females with a 46,X,r(X)/45,X chromosomal constitution comprise about 6-7% of cases with Turner Syndrome. A more severe phenotype is often present in individuals with small X ring chromosomes and is usually due to functional disomy. Failure of these X chromosomes to undergo inactivation is either due to lack of XIST gene or lack of its expression. The authors describe a case of a XIST-positive small ring X chromosome and the implications on the phenotype.

A girl aged 12 months with dysmorphic features, motor delay, growth failure and cardiomyopathy was referred for cytogenetic studies. The karyotype was obtained from peripheral blood lymphocyte cultures using standard techniques and high resolution GTL banding. Fluorescence in situ hybridization studies (FISH) were carried out with a specific probe for the centromeric region of the X chromosome (DXZ1 probe, Cytocell, UK). FISH with a specific probe for the XIST gene (Oncor) was also performed.

Cytogenetic analysis revealed mosaicism with two cell lines: one with a small marker chromosome 46,X,r(X), and another cell line with 45,X. The small marker was identified by FISH as a ring X chromosome and the presence of the XIST gene was also established. Final karyotype of the proband: mos 46,X,r(X)[38]/45,X[12].ish r(x)(DXZ1+,XIST+).

X inactivation in females with an abnormal X chromosome usually results in a Turner Syndrome variant phenotype. However, even small X ring chromosomes comprising the XIST gene may fail to undergo inactivation. The mechanism remains unclear. Failure in X dosage compensation results in both X chromosomes being active and consequently leading to a more or less severe phenotype.

The clinical description of this patient is in agreement with similar cases in the literature, however due to the patient's young age, mental impairment is still difficult to ascertain. Appropriate genetic counseling has been offered to both parents and it will also be available to the patient when she will reach reproductive age.

**Consent to publish:** The authors confirm that written informed consent was received by the patient for publication.

### 1.P31

### Double isochromosome Xq in mosaic Turner syndrome

Maja Ficko [1], Vanda Matuc [1], PrimoŽ Kotnik [2], Tinka Hovnik [1]
[1]Children´s Hospital, Umc, Unit of Special Laboratory Diagnostics, Ljubljana, Slovenia; [2]Children´s Hospital, Umc, Department of Pediatrics Endocrinology, Diabetes And Metabolic Diseases, Ljubljana, Slovenia
**Correspondence:** Maja Ficko
*Molecular Cytogenetics* 2017, **10(Suppl 1):**1.P31

**Background**

Isochromosome Xq is a structurally abnormal chromosome, consisting of two copies of the long arm (missing all or most of the short arms). It is seen in 18% of patients with Turner syndrome (TS), as a single cell line or in mosaicism with a 45,X cell line. TS results from complete or partial monosomy X and occurs in ~ 1/2000 to 1/5000 female births. Short stature, gonadal dysgenesis, and primary amenorrhea are the most important features of TS. We present a 2 year old female with short stature and three cell lines with either isodicentric X, two copies of isodicentric X or monosomy X.

**Methods and results**

Postnatal cytogenetic analysis on metaphase chromosomes was performed according to standard procedures using GTG-banding. Mosaicism was confirmed with fluorescence in situ hybridization (FISH) using LSI XIST (Xq13.2) and CEP X (DXZ1) (Vysis, Abbott Molecular). Cytogenetic analysis showed the karyotype mos 46,X,idic(X)(p11.2)[242]/47,X,+idic(X)(p11.2)x2[16]/45,X[7]. The percentage of the cell lines was estimated to 91% for the first , 6% for the second and 3% for the third cell line.

**Conclusıons**

The majority of isochromosomes Xq include proximal Xp material and are actually dicentric . Our results confirmed the structural rearrangement of the X chromosome and mosaicism with three different cell lines, including one rare cell line with two copies of idic(X).

**Consent to publish:** The authors confirm that written informed consent was received by the patient for publication.

### 1.P32

### Structural chromosome aberrations in individuals with development delay in The Hospital of Lithuanian University of Health Sciences

Zivile Zemeckiene[1], Ausra Kybartiene[1], Dale Medelinskiene[1], Justina Bekampyte[2], Inga Poceviciene[1], Virginija Asmoniene[1]
[1]The Hospital of Lithuanian University of Health Sciences, Department of Genetics And Molecular Medicine, Kaunas, Lithuania; [2]The Lithuanian University of Health Sciences, Medical Academy, Kaunas, Lithuania
**Correspondence:** Zivile Zemeckiene
*Molecular Cytogenetics* 2017, **10(Suppl 1):**1.P32

**Introduction**

Development delay is defined as a significant impairment of cognitive and adaptive functions, affects about 10% of all children. Chromosomal aberrations are one of the most common known causes of development delay, detected from 9% to 36% of all cases. The aim of this study was to evaluate large structural chromosome aberrations (karyotype) in children with development delay tested in The Hospital of Lithuanian University of Health Sciences.

**Methods**

110 children with development delay of unexplained origin (age from 2 to 18 years, average age 7,24 ± 4,8 years; female = 50, male = 60), who underwent genetic counseling in The Hospital of Lithuanian University of Health Sciences Kauno Klinikos during 2013–2016, were involved in this study. Cases with specific syndromes, like Down syndrome, were excluded from the study.

Karyotype analysis from peripheral blood lymphocytes was performed for all patients by the standard laboratory protocol. Chromosome preparations were stained with trypsin-Giemsa to obtain G-banding. C-banding was performed when necessary.

**Results**

Normal karyotype was found in 108 (98%) cases and two had structural chromosome aberrations: 45,XY,t(13;14)(q10;q10) and mos46,XY,t(4;11)(p11;p11)[5]/46,XY[47]. Both patients had significant development delay. First case had no dysmorphic features; the second was dysmorphic (both cases were negative for syndromic deletions specific to DiGeorge or Smith Magenis).

**Conclusions**

The cytogenetic findings were probably coincidental to the deveolpment delay.

### 1.P33

### Report of two moroccan siblings with trisomy 3q25 due to parental pericentric inversion of chromosome 3

Yassir Selouani [1], Wafaa Jdioui [1], Ilham Ratbi [1], Zineb Imane [2], Abdelaziz Sefiani [1]
[1]Centre De Génomique Humaine, Faculté De Médecine Et De Pharmacie, Université Mohammed V, Rabat-Morocco; [2]Service De Pédiatrie 2, Hôpital D'enfants De Rabat, Rabat-Morocco
**Correspondence:** Yassir Selouani
*Molecular Cytogenetics* 2017, **10(Suppl 1):**1.P33

**Introduction**

3q2 duplication syndrome is a rare chromosomal disorder, in which the symptoms and patient history vary widely depending on the length of the duplicated segment. In most cases (75%), trisomy 3q2 is the product of unbalanced segregation of balanced inversions or translocations and/or insertion in the parents' gonads.

**Case**

Here we report two Moroccan siblings with 3q2 duplication syndrome. The first patient is an 8 year old male, presenting with dysmorphic features, a multiple malformations, growth delay and a severe mental retardation. The second patient is a 5 months old female presenting dysmorphic features, hypotonia and multiple malformations. The first patient's tests were normal, while the biochemical investigations of the second patient showed hypothyroidism, and ultrasonography showed biliary stones, polysplenia, rudimentary inferior vena cava.

**Cytogenetic analysis**

Karyotyping of both patients and their parents showed that the patients were carriers of additional material on the short arm of chromosome 3, and the father had a balanced pericentric inversion of chromosome 3 with karyotype 46,XY,inv(3)(p26q25). Fluorescent in situ hybridization on patient's chromosomes showed that the additional materiel on the short arm of chromosome 3 corresponded to the 3q telomeric region. This result confirmed trisomy 3q25 in both patients.

**Discussion**

This report helps to further delineate the 3q2 duplication syndrome. Furthermore, we describe new clinical signs never reported in association with this disorder namely hypothyroidism, biliary stones, polysplenia and rudimentary inferior vena cava.

**Consent to publish:** The authors confirm that written informed consent was received by the patient for publication.

### 1.P34

### Constitutional chromothripsis involving whole chromosome 20 in a patient with facial dysmorphic features axial hypotonia and neonatal cholestasis

Lucie Tosca [1], Loïc Drévillon [2], Romain Diot [2], Oanez Ackermann [3], Aline Receveur [2], Minh-Tuan Huynh [1], Sophie Brisset [1], Gérard Tachdjian [1]

[1]Hôpital Antoine Béclère, Hôpitaux Universitaires Paris-sud, Aphp / Université Paris-sud, Unité De Cytogénétique, Service D'histologie, Embryologie Et Cytogénétique, Clamart, France; [2]Hôpital Antoine Béclère, Hôpitaux Universitaires Paris-sud, Aphp, Unité De Cytogénétique, Service D'histologie, Embryologie Et Cytogénétique, Clamart, France; [3]Hôpital Bicêtre, Hôpitaux Universitaires Paris-sud, Aphp Service D'hépatologie Et Transplantation Hépatique Pédiatriques, Le Kremlin Bicêtre, France

**Correspondence:** Lucie Tosca

*Molecular Cytogenetics* 2017, **10(Suppl 1):**1.P34

Chromothripsis is a newly described biological phenomenon characterized by massive chromosomal rearrangement. It involves shattering of one or few chromosome segments followed by a random reassembly of the fragments generated and this rearrangement occurs during one unique cellular event. Chromothripsis was well documented in tumors but was also recently observed in congenital disorders. Nevertheless, constitutive observations are extremely rare. At our knowledge, there is no description of constitutional chromothripsis affecting chromosome 20.

We report the case of a 3 month-old female patient. The family history was unremarkable. She displayed evident facial dysmorphic features with bilateral epicanthus, hypertelorism, long philtrum and low-set ears associated with axial hypotonia, cerebral ventricular dilatation, hepatosplenomegaly, cholestasis and hepatic cytolysis. The Allagile syndrome was suspected.

Cytogenetic analyses were performed from lymphocytes. Agilent 180 K Array-CGH showed 6 copy number variations on chromosome 20: terminal deletion 20p13 (2.29 Mb), deletion 20p12.1 (2.97 Mb), duplication 20q11.21q11.23 (5.4 Mb), deletion 20q13.12q13.13 (3.09 Mb), deletion 20q13.2 (1.7 Mb) and terminal duplication 20q13.33 (1.4 Mb). A total of 136 OMIM referenced genes were deleted or duplicated. The rearrangement contained 10 breakpoints localized on a single chromosome and log2 ratio plot suggested a cellular mosaicism estimated at 80%. The Alagille locus was not unbalanced (JAG1 gene, 20p12). The successive deletions and duplications were not clustered on a chromosomal region but spread over the entire chromosome 20. Standard karyotype revealed a cytogenetically visible rearrangement of one chromosome 20. The chromosomal mechanism was assayed by FISH analysis.

Genotype-phenotype correlation of this first case of constitutional chromothripsis affecting chromosome 20 is discussed. In the light of this new spectrum of chromosomal rearrangements, this report outlines the main features of these catastrophic events and underlines the absolute necessity to combine conventional and molecular cytogenetic tools.

### 1.P35

### Clinical and gonadal features of 45,X/46,XY a case report

Valerica Belengeanu [1], Cristina Popescu [1], Alina Belengeanu [2], Dana Cristina Bratu [3]

[1]Vasile Goldis Western University of Arad, Department of Genetics, Arad, Romania; [2]Victor Babes University of Medicine Timisoara, Department of Cell And Molecular Biology, Timisoara, Romania; [3]Faculty of Dentistry, Victor Babes University of Medicine And Pharmacy Timisoara, Orthodontics Department, Timisoara, Romania

**Correspondence:** Valerica Belengeanu

*Molecular Cytogenetics* 2017, **10(Suppl 1):**1.P35

Karyotype 45,X/46,XY mosaicism is a rare sex chromosome disorder of sex development and is considered to be a phenotypically very heterogeneous con¬dition. There is no obvious correlation between the phenotypic appearance and the respective cell line counts on routine peripheral blood karyotyping. Disorders of sex development (DSD)are associated with a broad spectrum of clinical phenotypes which may affect growth, hormonal balance, gonadal development, and histology.

**Case Report**

The patient was a 12-year-old boy with abnormal genitalia, short stature and obesity.

The patient's height was 132.5 cm (−2,16 SD), sitting height was 78.2 cm (−2.52 SD), his weight was 56.3 Kg and his BMI was 30.1 (+3.64 SD). In his physical examination bilateral cryptorchidism, and gynecomastia were observed.The hypospadiac penis measured 3 cm, 0.5 cm apparently. Laboratory evaluation showed low levels of insulin-like growth factor 1 and growth hormone. Radiographic examination of the skeleton demonstrated a bone age corresponding to 12.6 years 6 months and diffuse osteopenia.

Cytogenetic analysis demonstrated the presence of two cell populations, 42% with 45,X and 58% with 46,XY. FISH analysis confirmed cytogenetic abnormality. Laparoscopy showed a small testicles. The testicular biopsies found of variable degrees of bilateral dysgenetic testes. Because of the prevalence of tumors, especially gonadoblastomas and dysgerminomas, in these disorders of sex development, karyotyping is important for early recognition of such patients and their appropriate management. We suggest that boys with unexplained short stature should be screened with chromosomal analysis. The management of each patient should be individualized according to detailed anatomical and histological assessment.

**Consent to publish:** The authors confirm that written informed consent was received by the patient for publication.

### 1.P36

### Estimating the risk of unbalanced offspring in autosomal reciprocal translocations

Nadezda Shilova, Tatiana Tsvetkova, Valentina Antonenko

Federal State Budgetary Institution "research Centre For Medical Genetics", Laboratory of Cytogenetics, Moscow, Russia

**Correspondence:** Nadezda Shilova

*Molecular Cytogenetics* 2017, **10(Suppl 1):**1.P36

Autosomal reciprocal translocations (ART) are associated with reproductive failure in some carriers; for others, there may be a risk of offspring with multiple malformations and mental retardation due to malsegregation at meiosis resulting in gametes with chromosome imbalance (CI).To estimate the factors associated with the risk of a viable child with CI in 49 carriers of ART, binary logistic regression method (R-statistics) was used. The offspring of a healthy carrier is the statistical unit. Dichotomic (binary) variable was the status of the progeny:1 if the unbalanced child was viable, 0 – otherwise

(spontaneous abortion, fetal death or a child with a normal phenotype). The independent variables (predictors) were selected following characteristics of translocation affecting meiotic behavior of quadrivalent and zygote viability: R 1 - female sex of the carrier; R 2 - the translocated segments (TS) are enriched by G-positive bands; R 3 - chromosome 9 is involved in translocation; R 4 - acrocentric chromosomes (13–15, 21 and 22) are involved in the translocation; R 5 – severe asymmetric quadrivalent (the ratio of the longest and the shortest TS and the ratio of the longest and the shortest centric segments, calculated separately, were both 2 or more); R 6 – terminal breakpoints, when at least one of the two TS /the arms involved in translocation ratio <0,2 (model 1, Akaike information criterion (AIC):63,365). Model 2, predicting risk of a viable child with CI from variable R 6 (terminal breakpoints), was obtained after two steps (Wald's test: 2,675, p = 0,007; AIC: 59,088; ANOVA: $\chi 2 = 8.1743$, p = 0,004 for null model/model 2, $\chi 2 = 5.7228$, p = 0.3341 for model 1/model 2; OR: 6,2045; 95% CI: 1,63-23,63).Thus, lowest AIC value and achieved signification level suggest that terminal breakpoints are independently associated with the risk of a viable child with CI.

### 1.P37

### Karyotyping and array CGH Do we still need both

Anette Kildal Eek, Camilla Novy, Anne Signe Bø, Anne Gyrid Helland, Geir J. Braathen
Section of Medical Genetics, Department of Laboratory Medicine, Skien, Norway
**Correspondence:** Anette Kildal Eek
*Molecular Cytogenetics* 2017, **10(Suppl 1):**1.P37

Array-based analysis (aCGH) has gradually replaced traditional karyotyping as the primary diagnostic tool for identifying unbalanced chromosome abnormalities. Compared to karyotyping, aCGH improves the detection of small deletions, duplications and break points, but is limited in its inability to detect balanced rearrangements. Still there are reasons to keep both techniques available in a diagnostic genetics laboratory. We present three cases where the combined use of aCGH and karyotyping made it possible to understand the full picture of the aberration.
Case 1: Patient with one normal cell line and one cell line carrying a supernumerary marker chromosome identified as a ring 11.
Case 2: Patient with a supernumerary marker chromosome identified as an isodicentric chromosome 15.
Case 3: Patient with one cell line with an isodicentric Y chromosome and one cell line with two isodicentric Y chromosomes.

### 1.P38

### Count of "dark telomere" on the track of a biomarker for lithium treatment in bipolar disorder

Paola Caria [1], Daniela Virginia Frau [1], Tinuccia Dettori [1], Alessio Squassina [2], Claudia Pisanu [2], Caterina Chillotti [3], Maria Del Zompo [2], Roberta Vanni[1]
[1]Unit of Biology And Genetics, Biomedical Sciences, Monserrato, Italy; [2]Section of Neuroscience And Clinical Pharmacology, Biomedical Sciences, Monserrato, Italy; [3]Hospital of Cagliari, Unit of Clinical Pharmacology, Cagliari, Italy
**Correspondence:** Tinuccia Dettori
*Molecular Cytogenetics* 2017, **10(Suppl 1):**1.P38

Bipolar disorder (BD) is a severe psychiatric illness affecting approximately 1%-2% of individuals worldwide. BD is characterized by alternating manic and depressive episodes. Lithium is one of the most effective long-term treatments for BD, but 30%–40% of patients, fail to respond, and there is currently no method to identify the good lithium responders before initiation of treatment (Oedegaard et al., 2016). In a recent study, we demonstrated by quantitative PCR (qPCR), that leukocyte telomere length (LTL) of lithium responders (LiRs) and non-responders (non-LiRs) was positively correlated with years of lithium therapy. However, we fail to find significant differences between LiRs and non-LiRs LTL. We also reported quantitative fluorescence in situ hybridization (qFISH), measuring changes in telomere fluorescence intensity, in a subset of 8 patients (4 LiRs and 4 non-LiRs). Results by qPCR and qFISH were significantly correlated, but still, we found no statistically significant differences in LiRs and non-LiRs (Squassina et al., 2016). Since evidence suggests that the number of short telomeres rather than LTL determines telomere dysfunction (Elvashagen et al., 2011), we focused on the presence/absence of fluorescence chromosome ends to test if the mere enumeration of signals could significantly distinguish LiRs from non-LiRs. For this purpose, we analyzed 20 metaphases from 8 patients and 7 age-matched healthy controls, for positive and negative fluorescent telomeres. The results indicated that the number of negative (dark) telomeres was significantly higher in non-LiRS than LiRs (p = 0.041). In contrast, we observe no statistically significant difference between BD patients and healthy subjects (p = 0.36). Verification and validation of our preliminary results will prove if a simple count of "dark telomeres" may provide a valid biomarker distinguishing bipolar patients responding to lithium treatment.

**Funding**
RAS and Fondazione Banco Sardegna.

### 1.P39

### Terminal duplication of chromosome region 19q13.31q13.43 report of an infant with VACTERL association biliary atresia and global developmental delay

Martin Kehrer [1], Andreas Dufke [1], Sylke Singer [1], Thomas Liehr [2], Ute Grasshoff [1], Katja Buechner [3], Karin Schaeferhoff [1]
[1]University of Tuebingen, Institute of Medical Genetics And Applied Genomics, Tuebingen, Germany; [2]Friedrich Schiller University, Institute of Human Genetics, Jena University Hospital, Jena, Germany; [3]University Children's Hospital, Department of Neonatology, Tuebingen, Germany
**Correspondence:** Martin Kehrer
*Molecular Cytogenetics* 2017, **10(Suppl 1):**1.P39

Duplications of chromosome 19q are apparently very rare. Among the few patients with duplication of 19q described in the literature, only seven have a breakpoint defined by molecular karyotyping. The 19q duplication results in a variable phenotype, including dysmorphisms, multiple congenital anomalies and intellectual disability.
We report on a male infant with global developmental delay, microcephaly, ocular coloboma (iris, retina, choroid), extrahepatic biliary atresia, and various components of VACTERL association (butterfly vertebrae, anal atresia, atrial septal defect, clubfoot), in whom a de novo terminal 14.1 Mb duplication of 19q13.31q13.43 was detected by SNP array analysis. While initial chromosome banding analysis performed by Giemsa banding at the 500 band level showed normal results, fluorescent in situ hybridization (FISH) revealed that the duplicated region was attached to the short arm of chromosome 15. Karyotype was defined as: 46,XY,der(15)t(15;19)(p12;q13.31)dn.ish der(15)t(15;19)(p12;q13.31)(D19S238E+,MD54).arr[GRCH37] 19q13.31q13.43(44899917_58956816)x3 dn.
Most of the cases described with a duplication of distal 19q are associated with imbalances of other chromosome regions. There are only few reports of pure partial duplication of 19q resulting from translocations involving the short arm of acrocentric chromosomes. This is the first description of a patient with a pure 19q duplication due to a de novo unbalanced translocation between 19q and 15p. Furthermore, it is the largest reported 19q duplication with a breakpoint defined by molecular karyotyping. We review the literature and compare the phenotype of patients with similar chromosomal rearrangements.

### 1.P40

### Phenotypic spectrum associated with copy – number variation of chromosome 16p11.2 p12.2 locus

Iren Haltrich [1], Henriett Piko [2], Mariann Tihanyi [3], Zsuzsa Toth [1], Artur Beke [4], Veronika Karcagi [2], György Fekete[1]
[1]Semmelweis University, 2nd Department of Pediatrics, Budapest, Hungary; [2]National Institute of Environmental Health, Dept. of Molecular Genetics And Diagnostics, Budapest, Hungary; [3]Zala County Hospital, Department of Genetics, Zalaegerszeg, Hungary; [4]Semmelweis University, 1st Department of Obstetrics And Gynecology, Budapest, Hungary
**Correspondence:** Iren Haltrich
*Molecular Cytogenetics* 2017, **10(Suppl 1):**1.P40

We investigated 45 children with various signs of dysmorphism, neurodevelopmental disorder and/or intellectual disability by array comparative genomic hybridization using 1.4 M probes per subarray or 180 K oligo-array (assembly GRCh37/hg19) to identify submicroscopic CNVs. We discovered 16p11.2-p12.2 CNVs in 10 cases (7 duplications and 3 deletions). On the bases of breakpoints we could distinguish 3 groups of patients. 1) In the 16p12 region a ~527 Kb duplication in a 4 year old girl and an 173 Kb deletion in a 10 year old boy with a distal and a proximal breakpoint located ~21.8 Mb from the telomere. They have similar dysmorphic facial features, and developmental delay manifested particularly in speech. The boy carried an additional 2.4 Mb 15q11 duplication and had a severe ASD.

2) In the 16p11.2 region with 28.8 Mb proximal and distal breakpoints, respectively, we identified two small deletion cases with completely different phenotypes. A 3 year old boy with a smaller, 105.5 Kb deletion presented muscular hypotonia, moderate intellectual disability and delayed speech. A 3 year old girl with a 212.7 Kb deletion that included the SH2B1 gene, presented early onset, severe obesity (pc > 97), strabism and genu varum.

3) A 575 Kb duplication with the same breakpoints (29.6-30.1 Mb) was identified in two brothers and their mother. The brothers had ASD, but their mother was healthy.

In the case of a 12 year old boy an 1.84 Mb deletion was identified with unique 31.9 and 33.8 Mb breakpoints. He was hyperactive with short attention span and concentration problems, but did not have autism. Based on our data 16p11-p12 rearrangements predispose to neuropsychiatric problems without any distinctive phenotypic manifestations.

### 1.P41

### Delineation of the critical region for proximal deletion of chromosome 12q

Maria Sobol, Ann-Charlotte Thuresson
Uppsala University Hospital, Clinical Genetics, Uppsala, Sweden
**Correspondence:** Maria Sobol
*Molecular Cytogenetics* 2017, **10(Suppl 1):**1.P41

Proximal deletion of the long arm of chromosome 12 is a rare chromosomal abnormality described in about 20 patients. Known deletions span region 12q11-12q13 with genes YAF2 and/or AMIGO2 suggested as the candidate genes for the key phenotypic features such as growth and psychomotor retardation (Miyake et al., 2004; Gimelli et al., 2011).

Here we present a child with 3.1 Mb interstitial deletion of the long arm of chromosome 12 (arr[hg19] 12q12q13.11(43889138_47011108)x1) detected with SNP-array platform (Thermofisher). The patient was born after an unremarkable pregnancy at 39 weeks of gestation. Birth weight 3942 g and length 47 cm, Apgar 9-10-10. No dysmorphic features were observed. At age 14 months a tendency to growth and developmental delay was noticed. At 3 years of age the patient shows a clearly delayed motor and language development, accompanied by generalized muscular hypotonia. Also remarkable is growth retardation with hight 84.5 cm (−3 SD) and weight 12.2 kg (−2 SD). The clinical observations of our patient overlap with major common findings for published cases such as growth and developmental retardation.

The deletion observed in our patient spans 12 protein coding genes. However genes YAF2 and AMIGO2 are not involved. Hence our observation calls for reconsideration of the critical region and/or candidate genes for 12q proximal deletion associated with growth and developmental delay.

### 1.P42

### Two cases of de novo unbalanced X autosomal translocations

Zhanna Markova, Marina Minzhenkova, Tatiana Tsvetkova, Nadezda Shilova
Federal Budgetary State Institution "research Centre For Medical Genetics", Laboratory of Cytogenetics, Moscow, Russia
**Correspondence:** Zhanna Markova
*Molecular Cytogenetics* 2017, **10(Suppl 1):**1.P42

Translocations involving an X chromosome and an autosome are rare and are associated with a variable phenotype. Most unbalanced X-autosomal translocations result in multiple abnormalities; a smaller proportion cause gonadal dysgenesis without other anomalies or mental retardation. We present clinical, cytogenetic and molecular cytogenetic findings of two patients with de novo unbalanced X-autosomal translocations.

Case 1: Proband, 12-year-old girl, with only insignificant stigmata of Turner syndrome. Cytogenetic analysis (GTG-banding) of cultured peripheral blood lymphocytes revealed an abnormal karyotype with a der(X). Additional FISH analysis with WCP18, CEP18, subtelomere X and 18 DNA-probes, mBAND X and mBAND18 allowed us to interpret proband's karyotype as 46,X,der(X)t(X;18)(p22.3;p11.32)dn, resulting in a deletion of Xp22.3 → Xpter, and a duplication of of 18p11.32 → 18qter.

Case 2: Proband, 2-year-old girl, with mental retardation, delayed motor development, speech delay, dysmorphic features (including high forehead, hypertelorism, broad nasal bridge, low-set malformed ears, polydactyly of the left hand). Cytogenetic analysis (GTG-banding) of cultured peripheral blood lymphocytes revealed an abnormal karyotype with an additional material of unknown origin on a q-arm of chromosome X. M-FISH showed the material of chromosome 12 and mBAND 12 allowed us to identify the region 12q23 → 12qter. Thus, probands karyotype was interpreted as 46,X,der(X)t(X;12)(q26;q23)dn.

We present two cases of de novo unbalanced X-autosomal translocations with different phenotypic features which may be explained by the pattern of inactivation of the derivate chromosome X. We can assume that in case 1 der(X)t(X;18) is inactivated. Transcriptional silencing spreads into the autosomal chromatin on the der(X), thus reducing the phenotypic effect of the duplication of the segment of 18, and leading simply to a phenotype of partial Turrner syndrome. In case 2, the autosomal segment of the der(X) is probably functionally active and the proband's phenotype is due to the duplication of 12q24 → 12qter.

**Consent to publish:** The authors confirm that written informed consent was received by the patient for publication.

### 1.P43

### An evaluation and a systematization of the co occurrence of autosome and sex chromosome abnormalities

Vyacheslav Chernykh [1], Liubov Kurilo [1], Tatiana Tsvetkova [2], Zhanna Markova [2], Nadezhda Shilova [2], Oksana Ryzhkova [3], Alena Chukhrova [3], Aleksandr Polyakov [3]
[1]Research Centre For Medical Genetics, Genetics of Reproductive Disorders, Moscow, Russia; [2]Research Centre For Medical Genetics, Cytogenetics, Moscow, Russia; [3]Research Centre For Medical Genetics, Dna Diagnosis, Moscow, Russia
**Correspondence:** Vyacheslav Chernykh
*Molecular Cytogenetics* 2017, **10(Suppl 1):**1.P43

The aim of our study was to evaluate and systematize a co-occurrence of gonosomal and autosomal abnormalities.

We analyzed literature data and own results of about 10,000 cytogenetic and molecular chromosome analyses. All of these combined aberrations were classified into three groups: 'numerical + numerical', 'numerical + structural', 'structural + structural'. The first one includes double/triple aneuploidies: autosome trisomy (13, 18, 21) and disomy Y, trisomy X, Klinefelter (KS) or Turner syndrome (TS). It is the result of non-disjunction in maternal meiosis or a combination of maternal and paternal meiosis errors and/or post-zygotic non-disjunction. We found mosaic 47,XY,+21/48,XXY,+21 in 0.4% KS patients, and one prenatally diagnosed a 48,XYY,+13 karyotype. The second group consists of combinations of numerical and structural aberrations. Y-inversions are rarely associated with Klinefelter and Down syndromes. We examined one family (DS proband, healthy father, grandfather) with Y-inversion, and revealed that additional chromosome 21 was maternal in origin. We found reciprocal translocations in 0.5% KS patients, Robertsonian translocations in 1.4% SRY + XX-males and in 0.5% TS patients, including one chimera – 45,X/46,XY,+13,der(13;14)(q10;q10). The third group presents co-occurrence of two or more structural aberrations of autosome(s) and sex chromosome(s). We detected Yq11.23 (AZFc) microdeletions in 21% of men with balanced autosome rearrangements (inversions, Robertsonian and reciprocal translocations).
The occurrence of two or more chromosome aberrations is accidental, rarely it is the result of interchomosomal effect. More research is needed to evaluate the interrelationship of autosomal and gonosomal aberrations/CNVs.

### 1.P44

### A child with 8q24.13-8q24.3 duplication and 13q33.1-13q34 deletion inherited from a mother with balanced translocation

Krzysztof Bernatowicz [1], Wioleta Bonda [2], Studniak Ewa [2], Ryłów Małgorzata [2], Pietrzyk Aleksandra [1], Aniruddh Kashyap [1], Stanisław Zajączek [1]
[1]Department of Genetics And Pathology, Pomeranian Medical University, Szczecin, Poland; [2]Cytogenetic Unit, Pomeranian Medical University, Szczecin, Poland
**Correspondence:** Krzysztof Bernatowicz
*Molecular Cytogenetics* 2017, **10(Suppl 1):**1.P44

We report a 2 years old boy with microcephaly, severe mental and motor retardation, congenital heart defects, genitourinary malformation and facial dysmorphic features. Chromosome analysis showed 46,XY with suspicion of a 13q aberration. aCGH revealed a terminal 8q24.13-8q24.3 duplication and a 13q33.1-13q34 deletion, which was confirmed by FISH. BACs-On-Beads with Prenatal BoBs assay excluded aneuploidy particularly in the region responsible for Langer-Giedion syndrome, among others. The aberration turned out to be an unbalanced translocation derived from a maternal balanced translocation t(8;13)(q24;q33), which was confirmed in a standard karyotype of the phenotypically normal mother. This case has been compared to other patients with partial imbalances of 8q and 13q reported in the literature.
**Consent to publish:** The author declares that written informed consent was obtained for both a patient and his mother for publication of this case report.

### 1.P45

### Partial Trisomy 18 From a marker chromosome to a rare chromosomal disorder

Sumeyra Oguz [1], Naz Guleray [1], Ozlem Dogan [2], Pelin Ozlem Simsek Kiper [2], Gulen Eda Utine [2], Koray Boduroglu [2], Mehmet Alikasifoglu [1]
[1]Hacettepe University Faculty of Medicine, Department of Medical Genetics, Ankara, Turkey; [2]Hacettepe University Faculty of Medicine, Department of Pediatrics, Division of Pediatric Genetics, Ankara, Turkey
**Correspondence:** Sumeyra Oguz
*Molecular Cytogenetics* 2017, **10(Suppl 1):**1.P45

The trisomy 18 syndrome, also known as Edwards syndrome, has a live birth prevalance ranging from 1/3600 to 1/10000. The main clinical findings consist of growth retardation, characteristic facial features, heart malformations, typical finding of clenched hands with overriding fingers and intellectual disability. With respect to the etiology this syndrome can result from full, mosaic or partial trisomy 18. Partial trisomy 18 represents only 2% of all reported cases and the phenotype is highly variable depending on the duplicated regions and their extent. We here report on a fourteen-month-old infant presenting with dysmorphic facial features, growth retardation, aortic coarctation, renal genesis, unilateral ear anomaly and hearing loss. The patient's birth weight and developmental screening test were normal. The typical findings of the trisomy 18 syndrome consisting of prenatal growth deficiency, characteristic clenched hands with overriding fingers and nail hypoplasia were not present. Karyotype analysis revealed a marker chromosome in a regular form (47,XY,+mar). Microarray analysis using the Agilent SurePrint G3 CGH 8x60K showed a duplication of 6.9 Mb (chr18:18542074–25525771, hg19) from 18q11.1 to 18q12.1. Duplicated region consisted of a total of 26 OMIM genes none of which had been associated with a specific clinical finding so far. Several studies have attempted to find a genotype-phenotype correlation for duplications of different portions of chromosome 18 to identify a possible critical region. However, a single critical region for the trisomy 18 syndrome phenotype is unlikely to exist. With the widespread use of microarray technology, a better characterization of the critical regions in partial duplications is possible. Therefore clinical reporting of patients with partial duplications of chromosome 18 is strongly encouraged to expand the attempts of genotype-phenotype correlation and better address issues about patient management and genetic counseling.
**Consent to publish:** The authors confirm that written informed consent was received by the patient for publication.

### 1.P46

### Mosaic der(18)t(6;18)(p25.3;q21.31)

Vera Lima [1], Renata D`Oliveira [2], Joel Pinto [1], Manuela Rodrigues [3], Carla Ramalho [4], Sofia Dória [1]
[1]Faculty of Medicine And Instituto De Investigação E Inovação Em Saúde, University of Porto, Genetics Unit, Department of Pathology, Porto, Portugal; [2]Centro Hospitalar São João, Porto, Medical Genetics, Porto, Portugal; [3]Centro Hospitalar São João, Porto, Neonatal Intensive Care Unit, Porto, Portugal; [4]Centro Hospitalar São João And Faculty of Medicine, Porto And Instituto De Investigação E Inovação Em Saúde, University of Porto, Department of Obstetrics And Gynecology, Porto, Portugal
**Correspondence:** Sofia Dória
*Molecular Cytogenetics* 2017, **10(Suppl 1):**1.P46

#### Introduction

Microdeletion and microduplication syndromes are well-known causes of developmental delay and/or malformations. These abnormalities can now be detected relatively easily using array-CGH but they are more difficult to identify and interpret in cases of mosaicism. The presence of a mosaic whole-chromosome aneuploidy is more frequent than mosaicism for structural abnormalities, though several different types of these have been identified, including balanced and unbalanced rearrangements.

#### Case Report

A two month-old boy was born to non-consanguineous parents with multiple congenital abnormalities (complete cleft-palate, hypospadias, right cryptorchidism, craniofacial dysmorphisms, bilateral overlapping 2/3/4 toes, bilateral optic nerve dysplasia). Using ultrasound prenatal bradycardia were observed in first trimester and supraventricular extrasystole and overlapping toes in the second trimester of pregnancy; also aortic coarctation was suspected but not confirmed (neither prenatal nor postnatal). Apart from IUGR at 36 weeks no other major abnormalities were found. Brain MRI (at 1 month-old) unveiled a possible cortical migration defect (fronto-parietal polymycrogyria-like).

**Methods**

Array-CGH was performed using the Agilent 4x180K microarrays and cytogenomics 4.0.2.21 software. Karyotype and FISH analysis were performed using standards methods. Parental karyotypes have been requested.

Results: array-CGH revealed a 11,4 Mb duplication and a 21,9 Mb deletion in the terminal regions of the chromosomes 6 and 18, respectively. Mean log ratios of 0,36 (dup6pter) and −0,413 (del18qter) suggested mosaicism. Karyotype showed 46,XY,der(18)t(6;18)(p25.3;q21.31) in 15 from 30 metaphases analysed. FISH confirmed the presence of the derivative 18 in 103 from 200 cells analysed.

**Conclusions**

Although mosaic structural rearrangements are more frequently post-zygotic events, parental karyotype should also be investigated. In this patient, a genotype–phenotype correlation could be easy to establish, because of the well-known 18q deletion syndrome. Nevertheless, the presence in a mosaic state could mitigate the phenotype. Additionally, duplication of 6pter could also contribute to the phenotype.

**Consent to publish:** The authors confirm that written informed consent was received by the patient for publication.

### 1.P47

### Detection of a de novo mosaic MECP2 mutation in a patient with Rett syndrome phenotype

Angelos Alexandrou [1], Ludmila Kousoulidou [1], Ioannis Papaevripidou [1], Ioanna Alexandrou [1], Athina Theodosiou [1], Paola Evangelidou [1], Violetta Christophidou-Anastasiadou [2], Carolina Sismani [1]

[1]The Cyprus Institute of Neurology And Genetics, Cytogenetics And Genomics, Nicosia, Cyprus; [2]The Cyprus Institute of Neurology And Genetics And Archbishop Makarios Iii Hospital, Clinical Genetics, Nicosia, Cyprus

**Correspondence:** Paola Evangelidou

*Molecular Cytogenetics* 2017, **10(Suppl 1):**1.P47

Rett syndrome is a progressive neurodevelopmental disorder accounting for a large portion of intellectual disability (ID) in females. It is most frequently caused by mutations or deletions in the MECP2 gene that codes for the methyl-CPG-binding protein 2 and is located on Xq28. Here we present an interesting case of a novel mosaic MECP2 point mutation found in a patient with a typical Rett phenotype.

The patient is a female with autistic features, psychomotor delay, speech regression and stereotypic hand movements, initially referred for copy number screening to investigate Angelman or Rett syndromes. The results of MLPA analysis were negative and subsequent Sanger sequencing of MECP2 revealed a mosaic C→T nonsense mutation at position 139 of exon 3. A second round of Sanger sequencing with region-specific primers confirmed the presence and the mosaic state of the mutation. The same primers were used to test the biological parents, who were found negative. Restriction enzyme analysis gave normal results for both parents and was consistent with a mosaic mutation in the patient. In order to estimate the level of mosaicism, Next-Generation Sequencing was performed, showing approximately 25% abnormal cells.

This is the first known female mosaic MECP2 mutation carrier, who is exhibiting the full spectrum of the Rett syndrome phenotype. The specific mutation was not previously detected in other patients.

Further studies including a buccal swap are currently ongoing and are expected to provide insights into tissue specificity, thereby allowing for a better understanding of the patient's phenotype and relevant mechanisms.

### 1.P48

### Further MLPA subtelomeric tests in patients with 22q11DS phenotype

Yulia Kozlova [1], Larisa Akulenko [1], Valentina Antonenko [2], Natalia Kotlukova [3], Nadezda Shilova [2]

[1] Moscow State University of Medicine And Dentistry, Department of Medical Genetics, Moscow, Russia; [2]Federal State Budgetary Institution "research Centre For Medical Genetics", Laboratory of Cytogenetics, Moscow, Russia; [3]Moscow Municipal Clinical Hospital №67, Perinatal Cardiological Centre, Moscow, Russia

**Correspondence:** Yulia Kozlova

*Molecular Cytogenetics* 2017, **10(Suppl 1):**1.P48

In this study we reevaluated a group of patients with a distinctive phenotype previously tested for the common 22q11.2 deletion and major chromosomal aberrations using standard cytogenetic, FISH and MLPA methods. In order to find a genetic cause for typical heart defects combined with facial dysmorphisms MLPA test with SALSA P070 Subtelomere Probe Kit (MRC-Holland) was used on 71 patients negative for the 22q11.2 deletion. We found one patient with a common arterial trunk to have a 5q deletion and two patients with a double outlet right ventricle having 8p and 4q subtelomeric deletions. All patients were several months old and had had dysmorphic facial features. In two cases the probands' parents were not available for testing, leaving the question of polymorphisms unclear. We previously described the patient with 4q deletion as having an unbalanced translocation of parental origin, 46,XY,der(4)t(4;8)(q35;q22)mat. This case was included in the test group in order to understand the ability of MLPA to reveal subtelomeric duplications as well as deletions. In spite of double testing, the case of the proband with the translocation did not exactly show the result for the 8q duplication. Apparently this method lacks the efficiency for the detection of a duplication.

Thus, we can conclude that MLPA can still be considered as reliable and cost-effective primary test for the diagnosis of microdeletion syndromes, including subtelomeric ones, even if there is a risk that a duplication may be overlooked.

### 1.P49

### Interstitial deletion 4q21.21q21.23 in a boy with severe developmental delay hypotonia macrocephaly and dysmorphic features due to a cryptic paracentric inversion inv(4)(q21.21q21.23) in the mother

Sylke Singer [1], Karin Schaeferhoff [1], Martin Kehrer [1], Angelika Rieß [1], Andreas Dufke [1], Martin Schoening [2], Thomas Liehr [3], Ulrike A. Mau-Holzmann [1]

[1]Institute of Medical Genetics And Applied Genomics, University of Tuebingen, Tuebingen, Germany; [2]University Children's Hospital, University of Tuebingen, Tuebingen, Germany; [3]Institute of Human Genetics, University of Jena, Jena, Germany

**Correspondence:** Sylke Singer

*Molecular Cytogenetics* 2017, **10(Suppl 1):**1.P49

We report on a boy with severe hypotonia, global developmental delay, macrocephaly and further facial features. SNP array analysis was performed at the age of ten months and revealed an interstitial deletion in the long arm of a chromosome 4, with breakpoints in 4q21.21 and 4q21.23 and 6,15 Mb in size.

Conventional chromosome analysis of the parents showed normal karyotypes in both. In additional FISH experiments, the mother was identified as the carrier of a paracentric inversion inv(4)(q21.21q21.23). This inversion is not visible at the 550 band level.

There are a number of patients with interstitial deletions 4q described in the literature. Most of them are de novo in origin.

Interstitial deletions as the result of an unequal crossing over event in the carrier of a paracentric inversion are very rare in occurence. There are several reports, but none of them concerning chromosome 4.

The mechanism of formation will be discussed. In addition, clinical data will be presented and compared to other patients carrying this aberration.

**Consent to publish:** The authors confirm that written informed consent was received by the patient for publication.

### 1.P50

### Pure deletion and pure duplication of 19p13.2p13.12 in two family members beware of insertion

Marija Volk, Karin Writzl, Luca Lovrečić, Borut Peterlin
Umc Ljubljana, Clinical Institute of Medical Genetics, Ljubljana, Slovenia
**Correspondence:** Marija Volk
*Molecular Cytogenetics* 2017, **10(Suppl 1):**1.P50

We present a familial case of an intrachromosomal insertion of chromosome 19. The proband was a second child of non-consanguineous parents. A prenatal cytogenetic analysis was performed due to the positive screen for nuchal translucency and showed a normal male karyotype. At birth, he was small for gestation, had generalized muscle hypotonia and dysmorphic features. Because of perinatal phenotype aCGH was requested, which showed a heterozygous deletion of 19p13.2p13.12, 2.1 Mb in size, associated with features of Malan syndrome, while parental aCGH studies were normal. Family history revealed that a maternal uncle had mild intellectual disability and short stature. This prompted an aCGH analysis, which revealed a heterozygous duplication of the same 19p13.2p13.12 region. Deletion and duplication of 19p13.2p13.12 in two relatives alerted us to search for genomic rearrangement of chromosome 19. FISH analyses of the three generation family members confirmed that an intrachromosomal insertion ( (ins(19)(q13p13.2p13.12)) was present in proband's mother and grandmother. Labour intensive and time consuming karyotyping and customized FISH probes were utilized to recognize previously unrecognized intrachromosomal insertion. We anticipate that whole genome sequencing will play a crucial role in resolving complex structural genomic rearrangement cases in the future. To conclude, we described an intrachromosomal insertion that gave rise torecombinant chromosomes 19 in two relatives, one with a deletion and the other with a duplication of the inserted segment 19p13.2p13.12. Although, most interstitial deletions and duplication arise de novo, one should always think of the possibility of parental insertion. The detection of balanced parental chromosomal rearrangements is crucial in establishing familial recurrence risk.

**Consent to publish:** The authors confirm that written informed consent was received by the patient for publication.

### 1.P51

### Overlapping Langer Giedion Syndrome and Cornelia de Lange Syndrome type 4 an atypical case of microdeletion 8q23.3q24.12

Morgane Plutino, Fabienne Giuliano, Manal Dayem-Quere, Véronique Duboc, Houda Karmous-Benailly
Teaching Hospital, Department of Medical Genetic, Nice, France
**Correspondence:** Morgane Plutino
*Molecular Cytogenetics* 2017, **10(Suppl 1):**1.P51

Trichorhinophalangeal type II (TRPSII) or Langer-Giedion Syndrome (LGS) combines intellectual disability (ID), short stature, microcephaly, distinctive facial features and exostoses. TRPSII is a contiguous gene deletion syndrome usually encompassing both *TRPS1* and *EXT1* genes on chromosome 8q24.1-q24.13. Deardorff et al., (2012) identified a causative association between deletions and mutations in the *RAD21* gene, also localized on the 8q24.11 locus, and Cornelia de Lange Syndrome type 4 (CdLS-4). TRPS type I, LGS/TRPSII and CdLS-4 affected patients share many facial features. Deletions involving *RAD21* and not *TRPS1* result in typical dysmorphisms including thick eyebrows, long and flat philtrum, and thin upper lip vermilion. These facial hallmarks might be partially resulting from *RAD21* deletion. In general, individuals with heterozygous intragenic mutations and deletions of *RAD21* gene have clinical features reminiscent of those seen in CdLS such as short stature, synophrys, micrognathia, brachydactyly, mild radioulnar differences, vertebral anomalies, and mild cognitive involvement. We report here the case of a girl carrying a 1,7 Mb microdeletion encompassing *EXT1* and *RAD21* but not *TRPS1* with an atypical phenotype. She developed post-natal growth retardation, mild neurodevelopmental delay, one fibular exostosis, Solitary Median Maxillary Central Incisore (SMMCI) and pyriform aperture stenosis but no distinctive facial phenotype of LGS or CdLS-4. We will discuss the role of this interstitial microdeletion in this phenotype overlapping 2 clinical entities. Our observations suggest that the phenotypic consequences of *RAD21* disruption might be underestimated in TRPSII and CdLS patients.

### 1.P52

### Array CGH in patients with intellectual disability developmental delay our experience

Angeles Pérez-Granero [1], Angeles Pérez-Granero [2], Angeles Pérez-Granero [3], Maria Antonia Grimalt [4], Elena Miravet [4], Esther Cordoba [4], Begoña De Azua [5], Jordi Rosell [1], Jordi Rosell [2], Jordi Rosell [3]
[1]Hospital Son Espases, Genetica, Palma, Spain; [2]Institut De Investigacions Sanitàries De Palma (idispa), Genetica, Palma, Spain; [3]Centro De Investigación Biomédica En Red De Enfermedades Raras (ciberer), Genetica, Madrid, Spain; [4]Hospital Son Espases, Pediatria, Palma, Spain; [5]Hospital Son Llatzer, Pediatria, Palma, Spain
**Correspondence:** Angeles Pérez-Granero
*Molecular Cytogenetics* 2017, **10(Suppl 1):**1.P52

**ıntroductıon**

Genomic microarrays has been assumed to be the first genetic test offered to detect genomic imbalances in patients with unexplained intellectual disability / developmental delay, autism spectrum disorders and multiple congenital anomalies.

In this study we report the frequency of imbalances in a cohort of 641 patients with the clinical diagnosis of intellectual disability/developmental delay and accompanying dysmorphic features and/or congenital malformations.

**Methods**

We have examined samples from affected individuals by targeted array comparative genomic hybridization using a Cytochip ISCATM 8x60 v2.0 Illumina, over a 3-year period, between 2013 and 2016. When parental samples were available, they were included in the analysis.

**Results**

Almost all the patients had at least one copy number variant (CNV) independently of its clinical significance. Most of them classified as benign (63,5%). In 87 patients had a pathogenic change that explained the phenotype, and 23 cases had a potentially pathogenic change, total 17,2%. 147 patients had CNVs of uncertain significance.

**Dıscussıon**

The interpretation of the results is a great challenge due to the detection of a large amount of CNVs found in patients and healthy individuals.

Our studies provide more insights into the benefits derived by using chromosomal microarray analysis and demonstrate the usefulness of array CGH as a first-tier test in patients with intellectual disability and developmental delay.

### 1.P53

### A new case of 17p13.3 microduplication syndrome class II

Lavinia Caba [1], Dumitru D. Brănisteanu [2], Mihaela Grămescu [1], Roxana Popescu [1], Violeta Martiniuc [3], Monica Zeleniuc [4], Georgeta Cardos [4], Monica Pânzaru [1], Lăcrămioara Butnariu [1], Setalia Popa [1], Livia Matei [2], Eusebiu Vlad Gorduza [1]
[1]University of Medicine And Pharmacy "grigore T. Popa", Medical Genetics Department, Iasi-Romania; [2] University of Medicine And Pharmacy "grigore T. Popa", Department of Endocrinology, Iasi-Romania; [3]"cuza-voda" Obstetrics And Gynecology Hospital, Medical Genetics Department, Iasi, Romania; [4]Personal Genetics Laboratory, Medical Genetics Department, Bucharest, Romania
**Correspondence:** Lavinia Caba
*Molecular Cytogenetics* 2017, **10(Suppl 1):**1.P53

**Introduction**
The 17p13.3 microduplication syndrome is characterized by variable psychomotor delay and dysmorphia. Microduplications of 17p13.3 could be divided in class I duplications (that involve YWHAE, and are associated with autistic features, speech and motor delay, subtle dysmorphia and a tendency to postnatal overgrowth) and class II duplications (that involve PAFAH1B1 (+/− CRK and YWHAE) and are associated with developmental and psychomotor delay and hypotonia). Individuals with duplication that imply only PAFAH1B1 show microcephaly and severe growth restriction, but not particular dysmorphia.
**Methods**
We report a 9 years boy with short stature, developmental delay, subtle craniofacial dysmorphia (long face, upslanting palpebral fissures, broad nasal bridge, long philtrum, right clinodactily). Boy's mother presents intellectual disability. The proband was born at term with 1900 g (small for gestational age). Magnetic resonance imaging: Rathke pouch type I.
**Results**
We made chromosomal analysis (using cultures of lymphocytes and GTG banding) – normal result 46,XY ; MLPA using P245-B1 Microdeletion Syndromes-1 – abnormal result with a duplication of PAFAH1B1 from 17p13.3. We applied array-CGH (Sure Print G3 ISCA V2 CGH 8x60K Array Kit (Agilent Technologies), NimbleGen MS 200 Microarray Scanner and NimbleGen MS 200 Software v1.1), and the result confirm a 894 kb microduplication in 17p13.3 region in association with a 33 kb microdeletion in 16p13.3 region, a 461 kb microdeletion in 17q25.3 region and a 208 kb microdeletion in 22q11.23 - q12.1 region. Parents were not screened (adopted child).
**Conclusions**
The clinical signs in 17p13.3 microduplication syndrome are not specific and it needs array-CGH for diagnosis and to establish the genomic coordinates of the duplicated region.
This study was supported by founding of PN-II-PT-PCCA-2013-4-133 Program of UEFISCDI (National Romanian organism of research).
**Consent to publish:** The authors confirm that written informed consent was received by the patient for publication.

### 1.P54
### Correlation between cytogenetic and molecular genetic analysis in infertile males with azoospermia
Oguz Cilingir, Beyhan Durak Aras, Sara Khadem Ansari, Sabri Aynaci, Konul Haziyeva, Serap Arslan, Ebru Erzurumluoglu, Sevilhan Artan, Muhsin Ozdemir
Eskisehir Osmangazi University, Faculty of Medicine, Department of Medical Genetics, Eskisehir, Turkey
**Correspondence:** Oguz Cilingir
*Molecular Cytogenetics* 2017, **10(Suppl 1):**1.P54

Infertility is major social and health problem affecting up to 15% of couples of reproductive age. Male infertility accounts for about one half of infertility cases, among them 50-60% of the cases are azoospermia. The incidence of chromosomal abnormalities is especially high in azoospermia cases, varying from 13% to 24%. Not only chromosome anomalies, but also deletion of the Y chromosome region containing Azoospermia Factor (AZF) is frequently seen as a genetic cause of male infertility. We aimed to evaluate the type and frequency of chromosomal abnormalities and Y chromosome microdeletions in a total of 389 infertile men with azoospermia referred to our clinics.
Conventional karyotyping were performed on peripheral blood lymphocytes, using GTG banding. FISH analysis was used in the samples with suspicious translocations and for the detection of the mosaicism. Molecular diagnosis of Yq microdeletions were performed in 337 cases with normal karyotypes by the multiplex PCRs using 14 STS markers in the AZF regions on the Y-chromosome.
The overall incidence of chromosomal abnormality was 13.4% (52/389) with the most frequent one being Klinefelter Syndrome in 42 (10.8%) patients. Out of the 52 patients with chromosome aberrations; sex chromosome anomalies were observed in 46 cases (88.5%) including Klinefelter Syndrome, 47,XYY Syndrome, 45,X/46,XY mosaicism, 45,X/46,X,der(Y) mosaicism and 46,X,t(Y;2). Autosomal chromosomal anomalies detected in 6 cases (11.5%) and the 3 of them were structural aberrations of chromosome 12. Furthermore, incidence of Yq microdeletions was 2.97% and AZF(c + d) was the most frequent one. These results highlight the importance of chromosomal analysis in azoospermia cases. The microdeletions in AZF regions have shown to be pathologically involved in azoospermia. Therefore, genetic screening would add useful information for genetic counselling in azoospermia patients prior to starting assisted reproductive treatments.

### 1.P55
### Two new cases of ICF syndrome with no specific facial dysmorphism and due to a p.Gly583Ser homozygous mutation in DNMT3B in a consanguineous family from Gambia
Carmen Mediano [1], Anna Cueto[1], Roger Colobran [2], Andrea Martin-Nalda [3], Leonor Espi [1], Lourdes Trobo [1], Alberto Plaja [1], Elena Garcia-Arumí [1], Eduardo Tizzano [1]
[1]Hospital Universitari Vall D'hebron, Àrea De Genètica Clínica I Molecular, Barcelona, Spain; [2]Hospital Universitari Vall D'hebron, Servei D'immunologia, Barcelona, Spain; [3]Hospital Universitari Vall D'hebron, Patologia Infecciosa I Immunodeficiècies Pediàtriques, Barcelona, Spain
**Correspondence:** Carmen Mediano
*Molecular Cytogenetics* 2017, **10(Suppl 1):**1.P55

The Inmunodeficiency, Centromeric region instability and Facial anomalies syndrome (ICF) is a rare autosomal recessive disease characterized by recurrent infections caused by hypogammaglobulinemia and characteristic rearrangements in the vicinity of the centromeres (the juxtacentromeric heterochromatin) of chromosomes 1, 16 and 9. The majority of the 70 ICF cases reported to date involve mutations in the DNA methyltransferase gene DNMT3B. Dysregulation of this gene contributes to immunodeficiency and to hypomethylation of certain DNA regions resulting in particular chromosome rearrangements found in these patients.
We describe two siblings (brother and sister) diagnosed as ICF born of a consanguineous couple from Gambia. The boy was referred to our hospital due to growth delay, recurrent pulmonary infections, several episodes of sepsis and hypogammaglobulinemia. At the age of 7, his sister was born with intra and extrauterine growth delay;for this reason, a karyotype was requested in another hospital and showed the characteristic ICF chromosome rearrangements involving pericentromeric regions of chromosomes 1 and 16. A subsequent karyotype performed in the brother showed similar results. Both patients present no memory B cells and facial dysmorphism was not specific including broad flat nasal bridge and sloping forehead.
Molecular analysis in both siblings revealed a homozygous mutation in DNMT3B consisting in a nucleotide change in exon 16 (c.1747G > A) (p.Gly583Ser) predicted as pathogenic. Interestingly, this mutation was also recently found (by Sterlin et al., J.Clin Immunol, 2016 36,149-159) in another two patients from Gambia suggesting the possibility of a founder effect. These two new ICF cases highlight the possibility of misdiagnosis of this syndrome due to lack of facial clinical features and delay in performing karyotype analysis.
**Consent to publish:** The authors confirm that written informed consent was received by the patient for publication.

### 1.P56
### De novo 7q31 deletion involving FOXP2 gene associated with speech disability
Ebru Erzurumluoglu, Muhsin Ozdemir, Sinem Kocagil, Oguz Cilingir, Efsun Tosumoglu, Beyhan Durak Aras, Sevilhan Artan
Eskisehir Osmangazi University, Faculty of Medicine, Department of Medical Genetics, Eskisehir, Turkey
**Correspondence:** Sevilhan Artan
*Molecular Cytogenetics* 2017, **10(Suppl 1):**1.P56

Mutations in Forkhead box protein P2 (FOXP2), are known to cause an autosomal dominant condition characterized by developmental dyspraxia with profound speech and language deficits. Interrupting mutations in the gene sequence are sufficient for the development of phenotype and haploinsufficiency is the likely etiology. We report a 24-year-old male who was referred to the department for mild speech and learning disability. The patient had seizures until the age of three. Physical examination revealed some dysmorphic features including downslanting palpebral fissures, macrotia, high palate and bilateral single transverse palmar creases. His gross motor skills were normal but he had mild mental retardation; speech disability started in childhood. Conventional cytogenetic analysis revealed 46,XY,del(7)(q31). The karyotypes of the healthy, second degree consanguineous parents were normal. Array Comparative Genomic Hybridization (aCGH) analysis allowed fine mapping of the breakpoints of the deletion and identification of the deleted genes and confirmed a 18 Mb deletion at 7q31.1-q31.33 (108278159–126460673) region where 11 OMIM-disease-causing genes are located, including FOXP2 gene, as well. Although the 7q31 locus was known as Autism Susceptibility locus9 (AUTS9), he didn't receive autism spectrum disorder (ASD) diagnosis. Even though autism, severe speech disorders and dysmorphic phenotypic features have already been reported in the cases with such a large deletions, the clinical features of our presented case were accordant with childhood apraxia of speech(CAS). SNP array revealed a paternal FOXP2 deletion. Although the FOXP2 gene has been defined as a maternally imprinted and therefore paternally expressed gene, more recently, it has been suggested that parent-specific-transmission effects are coincidental. We conclude that there is still a lack of consensus on the phenotypic effects of FOXP2 disruptions. Therefore, further analyses in larger CAS series should be carried out to understand the pathophysiology of CAS and its haploinsufficient background.
**Consent to publish:** The authors confirm that written informed consent was received by the patient for publication.

### 1.P57

### ArrayCGH for the diagnosis of a ring X Turner syndrome variant

Viorica Elena Radoi [1], Oana Istrate [2], Marinela Malageanu [2], Sandra Grigore [2], Gratiela Chelu [2], Cristina Dragomir [2], Bianca Basangiu [2], Roxana Bohiltea [3], Laurentiu-Camil Bohiltea [4], Radu-Ioan Ursu [1]
[1]Synevo Romania; University O of f Medicine And Pharmacy, Medical Genetics, Bucharest, Romania; [2]Synevo Romania, Medical Genetics, Bucharest, Romania; [3]University of Medicine And Pharmacy, Obstetrics Gynecology, Bucharest, Romania; [4]University of Medicine And Pharmacy, Medical Genetics, Bucharest, Romania
**Correspondence:** Viorica Elena Radoi
*Molecular Cytogenetics* 2017, **10(Suppl 1):**1.P57

**Background**
Turner syndrome (TS, 45,X) is one of the most frequent chromosomal aberrations, with an incidence of 1/2500-3000 in newborn girls.
It is estimated than about half (50%) of the patients with TS present an apparently mosaic karyotype, the cell line other than the 45,X being either a rearranged X chromosome, a Y chromosomal variant, or other rearrangements.
**Methods and Results**
We report a 3 year-old girl who was referred to our medical genetics department for a slow growth rate and a Turner-like facial appearance.
A whole blood karyotype was performed. The result revealed a mos 45,X[43]/46,X,r(X)(p11.?q28)[28] formula.
Array-CGH (aCGH - Comparative Genomic Hybridization) analysis using the Agilent SurePrint G3 v2, 8x60 platform was performed to identify the breakpoints on the ring X and to evaluate the lost fragment size.
The aCGH results reported terminal deletion of both arms of the ring X chromosome (Xp11 and Xq28, respectively), with breakpoints between 38,011,254 and 153,242,719.
**Discussions**
Fertility options might exist in this case in the future.
A reproductive risk for the proband is a recombination event between the normal and the ring X chromosomes during meiosis, which may lead either to a non-disjunction or to a structurally unbalanced chromosomal anomaly.
A classic and molecular (aCGH) karyotype have been recommended for the mother of the patient. Pelvic ultrasound examination to identify possible existing gonadal tissue has also been indicated for the patient.
**Conclusion**
The typical TS clinical symptomatology, although milder (due to the presence of the second X chromosome), is a direct effect of the ring structure and subsequent DNA loss. Mosaicism is credited for much of the phenotypical variability, including the severity of ovarian dysfunction.
**Consent to publish:** The authors confirm that written informed consent was received by the patient for publication.

### 1.P58

### Sperm DNA fragmentation and chromosomal aneuploidy in men with unexplained infertility

Beyhan Durak Aras [1], Ayca Tanriverdi [1], Efsun Tosumoglu [1], Iyimser Ure [2], Muhsin Ozdemir [1], Oguz Cilingir [1], Sevilhan Artan [1]
[1]Eskisehir Osmangazi University, Faculty of Medicine, Department of Medical Genetics, Eskisehir, Turkey; [2]Eskisehir Osmangazi University, Faculty of Medicine, Department of Urology, Eskisehir, Turkey
**Correspondence:** Beyhan Durak Aras
*Molecular Cytogenetics* 2017, **10(Suppl 1):**1.P58

Infertility is a reproductive health problem that is observed in approximately 15% of couples around the world. Although half of the male infertility etiology is unknown, the other constitutes acquired and congenital defects. The parameters of the conventional sperm do not reliably reflect the male fertility potential. The defects in chromatin and DNA structure are important parameters in sperm quality and it is well known that these parameters could be indicative of male subfertility. Therefore, determination of sperm DNA fragmentation index (DFI) by using different assays has a great value in the assesment of the fertility potential of spermatozoa. The Sperm Chromatin Dispersion (SCD) test is an assay for sperm DNA fragmentation in semen. The present study was aimed to evaluate sperm DNA fragmentation and chromosomal aneuploidy in 50 infertile non-normozoospermic cases, in comparison with 50 normozoospermic control men of proven fertility. Semen samples from normozoospermic and non-normozoospermic infertile cases were processed for DNA fragmentation analysis by the SCD test: Big halo: no DNA fragmentation, small or no halos: nuclei with fragmented DNA. Fluorescent In Situ Hybridization (FISH) analysis was performed to identify aneuploidy for chromosome 13, 16, 18, 21, X and Y on both groups. The results showed a significantly higher DNA fragmentation rate (small/no halo rate) in sperm of infertile group (45%) compared to control group (24%) ($p < 0.001$).On the contrary, big halo rates were 70% and 96% in infertile and control groups, respectively($p < 0.001$). The autosomal and sex-chromosomal aneuploidy rates for the infertile patients were higher than for fertile individuals in the control group ($p < 0.05$). Our results also supported the utility of sperm DNA fragmentation tests in the clinical management of the unexplained infertile males.

### 1.P59

### A case of a non mosaic trisomy 22 in a 7 months old girl

Radu-Ioan Ursu [1], Sandra Grigore [2], Marinela Malageanu [2], Oana Istrate [2], Gratiela Chelu [2], Cristina Dragomir [2], Bianca Basangiu [2], Roxana Bohiltea [3], Camil Laurentiu Bohiltea [3], Viorica Elena Radoi [1]
[1]Synevo Romania; University of Medicine and Pharmacy, Medical Genetics, Bucharest, Romania; [2]Synevo Romania, Medical Genetics, Bucharest, Romania; [3]University of Medicine And Pharmacy, Medical Genetics, Bucharest, Romania
**Correspondence:** Radu-Ioan Ursu
*Molecular Cytogenetics* 2017, **10(Suppl 1):**1.P59

**Background**
The most frequently detected numerical chromosomal anomaly in spontaneous abortions, apart from trisomy 16, is trisomy 22. Due to its numerous severe phenotypical effects however, survival beyond the first gestational trimester is uncommon and full trisomy 22 is extremely rarely seen in living babies.
**Methods and case study**
We report a female patient aged 7 months old who was referred to our medical genetics department with the clinical suspicion of Down syndrome.
The patient had multiple clinical features, including severe growth retardation, muscular hypotonia, facial dysmorphism (depressed nasal bridge, flat nose, high arched palate, dysplastic ears, hypertelorism, epicantus, up-slanted palpebral fissures) and imperforate anus.
A classical whole blood karyotype was performed and revealed a 47,XX,+22 karyotype.
Array-CGH (aCGH - Comparative Genomic Hybridization) analysis using the Agilent SurePrint G3 v2, 8x60 platform was performed to identify the origin of the additional genetic material and confirmed the full trisomy 22 diagnosis.
There is a very small number of postnatal cases reported with non-mosaic trisomy 22, of which just a few showed longer survival (the mean age at death is only 4 days). Our patient reached the age of 7 months, but is in intensive care at the moment with severe respiratory insuffiency and has a poor prognosis.
**Conclusion**
The clinical findings in this case confirm other reports in the literature showing that children with non-mosaic trisomy 22 may survive birth and beyond.
**Consent to publish:** The authors confirm that written informed consent was received by the patient for publication.

### 1.P60

### An interstitial deletion at 8q22.3 q24.11 associated with the Tricho Rhino Phalangeal Syndrome (TRPS) type I

Sevilhan Artan, Oguz Cilingir, Ebru Erzurumluoglu, Hasan Bas, Beyhan Durak Aras, Sabri Aynaci, Muhsin Ozdemir
Eskisehir Osmangazi University, Faculty of Medicine, Department of Medical Genetics, Eskisehir, Turkey
**Correspondence:** Hasan Bas
*Molecular Cytogenetics* 2017, **10(Suppl 1):**1.P60

The Tricho–Rhino–Phalangeal Syndromes (TRPS) are rare autosomal dominant genetic disorders characterized by craniofacial dysmorphism and bone deformities. They are subclassified into three different types (TRPSI, TRPSII, also known as Langer Giedion syndrome (LGS), and TRPSIII) depending on either gross deletions or loss of function mutations of the TRPS1 gene, located on 8q23.3 and EXT1 gene on 8q24.11. We report a female patient who was referred to our clinic due to developmental delay and dysmorphic facial features. Our 4 years old female patient was the second child of healthy unrelated parents. She had a healthy brother and no family history of TRPS1. She was born at 41 weeks of gestation after an uneventful pregnancy but observed in incubator for 3 days for breathing problems. Her developmental milestones was mildly behind her peers on infancy. She was examined by pediatric psychiatrists for possible attention-deficit/hyperactivity disorder. Physical examination revealed a weight of 11 kg (<3rd percentile), a height of 93 cm (10th percentile) and a head circumference of 46 cm (<3rd percentile). She had a long face, macrotia, sparse hair, thin eyebrows and prominent long philtrum. Cytogenetic and array-Comparative Genomic Hybridization (aCGH) analyses revealed that the patient was a carrier of an interstitial deletion at 8q22.3q24.11 (105352043–118391406) of 13 Mb. Parental karyotypes were normal. The deletion included TRPS1 gene on 8q23.3 but not EXT1 gene on 8q24.11. The clinical features and genetic analyses of the presented case were coherent with TRPS type I. She had no multipl exostoses and no deletion detected on EXT1 gene that are specific for TRPS type II. We concluded that long term follow-up and examination of psychomotor development of the presented case are necessary to evaluate the role of deleted genes.
**Consent to publish:** The authors confirm that written informed consent was received by the patient for publication.

### 1.P61

### Rare cytogenetic anomalies in 2q37 microdeletion syndrome

Monica Cristina Panzaru, Roxana Popescu, Mihaela Gramescu, Lacramioara Butnariu, Irina Resmerita, Cristina Rusu, Eusebiu Vlad Gorduza
University of Medicine And Pharmacy, Medical Genetics, Iasi, Romania
**Correspondence:** Monica Cristina Panzaru
*Molecular Cytogenetics* 2017, **10(Suppl 1):**1.P61

2q37 microdeletion syndrome is characterized by short stature, obesity, characteristic facial appearance, brachymetaphalangy of digits 3–5, joint hypermobility/dislocation and scoliosis, hypotonia, mild-moderate intellectual disability and autism disorders. Phenotypic variability is observed. Chromosome analysis confirms the diagnosis in 80%-85% of patients. In 15%-20% of patients the reduced size of the deleted fragment can only be detected using deletion analysis. 2q37 microdeletion can result, in some cases, from chromosomal rearrangements involving 2q37 region (e.g., inversion, ring or reciprocal translocation). No case of mosaicism has been documented to date. Deletion size does not correlate well with phenotype.
We present two patients with 2q37 deletion and rare associated chromosomal abnormalities. One had a deletion due to a mosaic translocation and the other patient had a del/dup rearrangement.
Case1- female, 4 years old. Clinical features – facial dysmorphism: frontal bossing, fine highly arched eyebrows, deep set eyes, hypoplastic alae nasi, prominent columella, teeth anomalies, dysplastic ears; brachymetaphalangy; ventricular septal defect; umbilical and inguinal hernia; joint hypermobility; mild developmental delay. Karyotype: 46,XX,1qh+ /46,XX,1qh+,t(2;9) (q37.3;q12) [30/8]. MLPA: 46,XX.mlpa 2qsubtel x1.
Case 2 – female, 5 years old. Clinical features – facial dysmorphism: arched eyebrows, upper lids ptosis, broad nasal bridge, tented upper lip, cleft palate, dysplastic ears; single transverse palmar crease; atrial septal defect; moderate developmental delay; autism. Karyotype: 46,XX, add(2). MLPA: 46,XX.mlpa 2qsubtel x1,15qsubtelx3.
Deletion size will be discussed. The phenotypes of our patients will be compared with other reports in the literature with 2q37 microdeletion.
Our cases illustrate the genetic heterogeneity of cytogenetic abnormalities in 2q37 microdeletion syndrome.
**Consent to publish:** The authors confirm that written informed consent was received by the patient for publication.

### 1.P62

### A de novo ins(21;13) and two interstitial deletions in 13q in a boy with multple congenital anomalies

Ozden Altiok Clark [1], Gokhan Ozan Cetin [1], Banu Nur [2], Asli Toylu [1], Sibel Karauzum [3], Ercan Mihci [2]
[1]Akdeniz University Medical Faculty, Medical Genetics, Antalya, Turkey;
[2]Akdeniz University Medical Faculty, Pediatric Genetics, Antalya, Turkey;
[3]Akdeniz University Medical Faculty, Medical Biology And Genetics, Antalya, Turkey
**Correspondence:** Ozden Altiok Clark
*Molecular Cytogenetics* 2017, **10(Suppl 1):**1.P62

In this report, we present a 13-year-old boy with chronic renal failure and mental-motor retardation. By the age of five, this patient had already undergone several surgeries: an anal atresia operation at 15 days old, an anorectoplasty at 13 months, and a vesicoureteral reflux operation at 5 years of age. Developmental delay, a dysmorphic apperance, and strabismus were also observed. Additionally, a skeletal survey showed mild thoracal scoliosis, thoracal kyphosis, and lumbar lordosis.
Chromosome analysis of the patient showed an exceptional interchromosomal insertion of a segment of the long arm of 13q into the short arm of 21. His karyotype was 46,XY,ins(21;13)(p13?;q32q14.1).

Molecular karyotyping revealed two interstitial deletions in chromosome 13: 5.7 Mb at 101,012,528-106,764,677 and 4.5 Mb at 108,133,173-112,622,85. His final molecular karyotype was found to be (arr[hg19] 13q32.3q33.2(101,012,528-106,764,677)x1, 13q33.3q34 (108,133,173-112,622,853)x1).
These deleted regions encompass the 47 RefSeq genes and include genes, such as COL4A1 and COL4A2, which may impact renal development. These deleted regions did not, however, include the EFNB2 gene, which was a candidate gene for anorectal atresia.
We will compare and discuss the phenotypic similarities between our patient and those with VATER/VACTERL-like syndrome.
**Consent to publish:** The authors confirm that written informed consent was received by the patient for publication.

### 1.P63

### Duplication deletion disorder – rare chromosome imbalances

Roxana Popescu [1], Adriana Sireteanu [2], Monica Panzaru [1], Lacramioara Butnariu [1], Elena Braha [1], Lavinia Caba [1], Setalia Popa [1], Mihaela Gramescu [1], Elena Graur [1], Eusebiu Vlad Gorduza [1], Cristina Rusu [1]
[1] "grigore T. Popa" University of Medicine And Pharmacy, Department of Medical Genetics, Iasi-Romania; [2]Regional Institute of Oncology, Laboratory of Molecular Biology, Iasi-Romania
**Correspondence:** Roxana Popescu
*Molecular Cytogenetics* 2017, **10(Suppl 1):**1.P63

Subtelomeric rearrangements are a major cause of idiopathic intellectual disability (ID). The combined use of MLPA kits has a relatively high detection rate in ID patients. We report 5 cases with rare subtelomeric rearrangements resulting in deletion/duplication involving the same chromosome. MLPA was performed using P070/P036 and Telomere Follow-up. The unbalanced rearrangements were confirmed and completely characterized by aCGH. The mechanisms will be discussed.
Case 1: female, 8 year old. Clinical findings: dysmorphic face, obesity, hypotonia, moderate ID, speech delay. Karyotype: 46,XX,add(5)(p15.1); MLPA P358: deletion in 5p15.33 > 1.9 Mb and duplication 5p15.31-5p15.2 > 7.1 Mb. Diagnosis: Inverted duplication deletion 5p.
Case 2: male, 6 months old; family history: two early miscarriages. Clinical findings: growth failure, microcephaly, dysmorphic face, hypotonia. Echocardiography: ASD, PDA. Karyotype: 46,XY,add(2)(q37); MLPA P264: deletion in 2q37.3 > 1.77 Mb and duplication 2q37.3 > 2.6 Mb. Diagnosis: Inverted duplication deletion of 2q.
Case 3: female, 1.5 year old. Clinical findings: turricephaly, dysmorphic face, pectus excavatum, hypotonia, severe motor delay; MRI: corpus callosum agenesis. Karyotype: 46,XX,add(8)(p23.1); MLPA P208: deletion in 8p23.3-p23.1 > 6.4 Mb; Array: 6.8 Mb deletion 8p23.3-p23.1 and 31.3 Mb duplication 8p23.1-p11.1. Diagnosis: Inverted duplication deletion 8p.
Case 4: female, 4 year old; premature birth. Clinical findings: dwarfism, dysmorphic face, small hands, severe ID. Echocardiography: ASD, PDA, mitral and tricuspid regurgitation. Karyotype: 46,XX,add(3)(p25); MLPA P208: deletion 3p26.3 > 1.1 Mb; P264: duplication 3q29 > 3.9 Mb. Diagnosis: deletion 3p and duplication 3q. For orientation two color FISH is required.
Case 5: male, 2 year old. Clinical findings: asymmetric and dysmorphic face, hypotonia, speech delay; Echocardiography: ASD, mitral and tricuspid regurgitation. Karyotype: 46,XY; MLPA P249: deletion 18p11.3 > 3.5 Mb; P320: duplication 18q22.3-q23 > 5.2 Mb; Diagnosis: deletion 18p and duplication 18q. For orientation - two color FISH.
Conclusion: MLPA is useful to detect aberrant chromosomes derived from inverted duplication deletion.
**Consent to publish:** The authors confirm that written informed consent was received by the patient for publication.

### 1.P64

### Rare complex gonosomal and autosomal mosaicism in a female patient with severe developmental delay

Uwe Heinrich [1], Monika Cohen [2], Imma Rost [3]
[1]Mvz Martinsried, Cytogenetics, Martinsried, Germany; [2]Kbo Children's Centre, Clinical Genetics, Munich, Germany; [3]Mvz Martinsried, Clinical Genetics, Martinsried, Germany
**Correspondence:** Uwe Heinrich
*Molecular Cytogenetics* 2017, **10(Suppl 1):**1.P64

We present the results of the chromosomal, FISH, and microarray analyses of an 8 years old female patient born to non consanguineous parents. Phenotypically, she had severe developmental delay, muscular hypotonia, hyperactivity, hypertelorism, outer ear dysplasia, and glaucoma. At the age of 11 month, a first chromosome analysis was performed showing three different cell lines: cell line 1 with a chromosome 19 having additional material at the tip of the short arm; cell line 2 with the aberrant chromosome 19 and a putative ring chromosome X; and cell line 3 with the aberrant chromosome 19 and a second, aberrant X chromosome carrying a putative interstitial duplication Xp. A second chromosome analysis in combination with FISH analyses seven years later could prove the aberrant chromosome 19 as a derivative chromosome resulting from a translocation (X;19)(p21.1;p13.3). The ring X could be confirmed, but the presumptive dup(Xp) could not be confirmed. Instead, array cgh analysis could prove this structurally aberrant X chromosome to be a derivative X chromosome resulting from a translocation (X;1)(p21.1;q32.2). The breakpoints of the r(X) could be determined as p11.22 and q22.1. Altogether the patient showed the following imbalances: cell line 1 with a deletion Xp21.1- > qter; cell line 2 with a deletion Xq22.1- > qter; cell line 3 with a deletion 1q32.2- > qter. This case again demonstrates that only the combination of different techniques such as chromosome, FISH, as well as array cgh analysis is capable of elucidating the complete picture of such complex aberrations.
**Consent to publish:** The authors confirm that written informed consent was received by the patient for publication.

### 1.P65

### Clonal chromosomal abnormalities in ataxia telangiectasia

Vivi Miriam Srivastava [1], Gautham Arunachal [2], Pujari Ganesh Pandurang [1], Yuvarani S [1], Sumita Danda [2]
[1]Christian Medical College, Cytogenetics, Vellore, India; [2]Christian Medical College, Clinical Genetics, Vellore, India
**Correspondence:** Vivi Miriam Srivastava
*Molecular Cytogenetics* 2017, **10(Suppl 1):**1.P65

We present the clonal cytogenetic abnormalities seen in the lymphocytes of 12 patients with clinical features of ataxia telangiectasia (AT) seen at the Christian Medical College, Vellore, India from 2005–2016. Metaphases obtained by culturing peripheral blood for 72 hours with phytohemagglutinin stimulation following standard protocols were analysed using an automated karyotyping system. At least 20 G-banded metaphases were studied from each case. If a non-clonal abnormality was seen, upto 50 or more metaphases were studied to establish clonality. Our patients, eight of whom were males, ranged in age from 4–13 years. All 12 had clonal abnormalities of chromosome 7 and /or chromosome 14. The inversion (7)(p14q35), t(7;14)(q35;q11.2) and the t(7;14)(p14;q11.2) were the most commonly seen clonal abnormalities, being present in 50%, 40% and 25% respectively. The other clonal abnormalities seen were the t(7;7)(p14;q35), the t(14;14)(q11.2;q32) and the inv(14)(q11.2q32). These abnormalities were also present non-clonally in some patients. Several unusual clonal abnormalities were present in addition to the above, involving chromosomes 2, 16 and 22, apart from chromosomes 7 and 14. These were t(7;14)(q22 ~ q31;q22), t(7;14)(q35;p11.2), r(2)(p25q37), t(2;16)(q13;p13.3), t(2;22)(q13;q13) and add(16)(p13.3). The breakpoints of most of these unusual abnormalities did not involve regions in which T cell receptor subunits are located. At the time of testing, none of our patients had evidence of a neoplasm, but one child's brother had died of leukemia. Information about consanguinity was available for six patients, three of whom had consanguineous parents.

### 1.P66

### Characterization of five new patients with AUTS2 deletions detected by aCGH

Sónia Custódio, Ana Sousa, Rosário Silveira-Santos, Sílvia Serafim, Oana Moldovan, Juliette Dupont, Patrícia Dias, Ana Berta Sousa
Hospital De Santa Maria/centro Hospitalar Lisboa Norte, Centro Académico De Medicina De Lisboa, Serviço De Genética Médica, Departamento De Pediatria, Lisboa, Portugal
**Correspondence:** Sónia Custódio
*Molecular Cytogenetics* 2017, **10(Suppl 1):**1.P66

The use of high resolution microarray analysis as a first tier approach for the etiological investigation of patients with neurodevelopmental disorders has uncovered several genes associated with these clinical entities. Among them AUTS2, known to have a regulatory function in brain development. Copy number variants involving this gene have allowed the delineation of AUTS2 syndrome characterized by intellectual disability (ID), feeding difficulties, microcephaly, minor dysmorphic features and a specific behavioral phenotype.

Here we report five patients, referred to our medical genetics department for ID, all carrying deletions involving the AUTS2 gene, detected by aCGH (CGX-HD 180 k, PerkinElmer). The deleted regions range from 174 kb to 1.59 Mb in size. Three deletions are intragenic involving one or several exons, and the other two deletions are located at the extremities of the gene, one at the 5´ end spanning the promoter region and the other at the 3´end encompassing also two downstream genes. Inheritance was possible to assess in only three of the patients, and the deletion occurred de novo in all three.

All patients presented with dysmorphism and ID with speech delay. Behavioral problems and attention deficit hyperactivity disorder were also common findings, while autism spectrum disorder was present in only one patient.

The diversity of cases reported here with deletions scattered all across AUTS2 contributes to genotype/phenotype correlations and further delineation of the AUTS2 syndrome.

### 1.P67

### A report of mosaic Turner syndrome with a mild Kabuki like phenotype

Kanay Yararbas [1], Ceyhan Sayar [2], Sait Tumer [2], Cemre Tavukcu [2], Nihal Oncu [2], Derya Ozciger [2], Cumhur Ekmekci [2], Aysegul Kuskucu [3], Yasemin Alanay [1]
[1]Acibadem University Medical School, Medical Genetics, Istanbul, Turkey; [2]Acibadem Healthcare Group, Genetic Diagnosis Center, Istanbul, Turkey; [3]Yeditepe University Medical School, Medical Genetics, Istanbul, Turkey
**Correspondence:** Aysegul Kuskucu
*Molecular Cytogenetics* 2017, **10(Suppl 1):**1.P67

We present a 5 year old girl who was born at term by sectio from a second pregnancy of non-consanquineous parents. Her birth weight was 2300 g, and length 43 cm. The patient showed a mild phenotype of Kabuki syndrome. Both parents were healthy. Her karyotype was 45,X/46,X,r(X). FISH analysis verified that the ring was derived from X and included XIST, which is important for the evaluation of the phenotype.

We will present the array characteristics of the marker chromosome and further evaluate the clinical phenotype of the patient.

### 1.P68

### Clinical cytogenetic and molecular study of Fanconi anemia in Polish population

Anna Repczynska [1], Katarzyna Julga [1], Marta Ewa Bryskiewicz [2], Agata Pastorczak [3], Katarzyna Babol-Pokora [3], Jolanta Skalska-Sadowska [4], Mariusz Wysocki [5], Agnieszka Zaucha-Prazmo [6], Katarzyna Drabko [6], Artur Bossowski [7], Beata Zalewska-Szewczyk [8], Bozena Dembowska-Baginska [9], Wojciech Mlynarski [3], Stanislaw Zajaczek [10], Olga Haus [1]
[1]Collegium Medicum In Bydgoszcz, Nicolaus Copernicus University In Torun, Department of Clinical Genetics, Faculty of Medicine, Bydgoszcz, Poland; [2]Independent Public Clinical Hospital No. 2 of The Pomeranian Medical University In Szczecin, Cytogenetics Unit, Department of Laboratory Diagnostics, Szczecin, Poland; [3]Medical University of Lodz, Laboratory of Immunopathology And Genetics, Department of Pediatrics, Oncology, Hematology And Diabetology, Lodz, Poland; [4]Medical University In Poznan, Department of Oncology, Hematology And Pediatric Transplantology, Poznan, Poland; [5]Collegium Medicum In Bydgoszcz, Nicolaus Copernicus University In Torun, Department of Pediatric Hematology And Oncology, Faculty of Medicine, Bydgoszcz, Poland; [6]Medical University of Lublin, Department of Pediatric Haematology, Oncology And Transplantology, Lublin, Poland; [7]Medical University of Bialystok, Department of Pediatrics, Endocrinology, Diabetology With Cardiology Division,, Bialystok, Poland; [8]Medical University of Lodz,, Department of Pediatrics, Oncology, Hematology And Diabetology, Lodz, Poland; [9]The Childrens' Health Institute, Department of Oncology, Warsaw, Poland; [10]Independent Public Clinical Hospital No. 2 of The Pomeranian Medical University, Cytogenetics Unit, Department of Laboratory Diagnostics, Szczecin, Poland
**Correspondence:** Anna Repczynska
*Molecular Cytogenetics* 2017, **10(Suppl 1):**1.P68

#### Background

Fanconi anemia (FA) is a rare heterogenous genetic syndrome which clinical suspicion is mostly based on growth retardation and congenital defects associated with lifethreatening bone marrow failure. However, clinical manifestation are highly variable and some symptoms may overlap with those observed in other syndromes, making a reliable diagnosis based on clinical features impossible. Analysis of chromosome fragility induced by DNA interstrand crosslinking agents (ICL), such as diepoxybutane (DEB) or mitomycin C (MMC) is still the 'gold standard' test for the diagnosis of FA.

#### Methods

Clinical and hematological data from 94 patients with suspicion of FA were obtained from genetic counsel or hematology clinics. Cytogenetic studies were carried out on peripheral blood lymphocytes from cultures supplemented with MMC (50nM, 150nM and 300nM) or DEB (0,1 μg/ml). Chromosome instability data were analyzed and calculated (number of metaphases with breakage, mean chromosome breakage number per aberrant metaphase, and tri-, tetra- and multi- radials frequency). Multiplex Ligation-dependent Probe Amplification and Next-Generation Sequencing were applied to confirm positive results of chromosomal breakage test. Clonogenic MMC survival test of fibroblasts was used to detect mosaics. All cytogenetic tests were also done in healthy parents of ill children. The study was conducted for over 6 years (2011–2017).

#### Results

Chromosome fragility was analyzed in all 94 patients. The MMC test was positive in 6 patients and DEB test in 2 patients. In 13 patients the result of MMC test was equivocal. According to results of chromosomal breakage test, the patients were divided into three subgroups: sure FA (8 patients), possible mosaic FA (13 patients) and no FA (73 patients). In four FA patients mutation analysis was also done, confirming the cytogenetic diagnosis. The mutations were detected in genes: FANCA, FANCD1/BRCA2, FANCD2 and FANCI.

### 1.P69

### Rare genomic imbalances in patients with neurodevelopmental disorders and complex phenotypes

Sorina Mihaela Papuc [1], Andreea-Cristina Tutulan-Cunita [1], Magdalena Budisteanu [1], Ioana Minciu [2], Dana Barca [2], Bogdan Budisteanu [3], Raluca Colesniuc [1], Ioana Borcan [1], Catrinel Iliescu [2], Carmen Burloiu [2], Dana Craiu [2], Aurora Arghir [1]
[1]"victor Babes" National Institute of Pathology, Medical Genetics Laboratory, Bucharest, Romania; [2]Prof. Dr. Alex. Obregia" Clinical Hospital of Psychiatry, Department of Pediatric Neurology, Bucharest, Romania; [3]Prof. Dr. Alex. Obregia" Clinical Hospital of Psychiatry, Department of Pediatric Psychiatry, Bucharest, Romania
**Correspondence:** Sorina Mihaela Papuc
*Molecular Cytogenetics* 2017, **10(Suppl 1):**1.P69

Structural variation is playing a major etiological role in neurodevelopmental disorders (NDDs), and numerous studies have demonstrated the relevance of both recurrent and rare genomic regions. We report on the copy number variants (CNVs), not yet associated with distinct clinical entities, identified by array-CGH in a cohort of patients with NDDs and complex phenotypes.
From a larger study focused on genetic testing by array-CGH (Agilent Technologies) in a pediatric population with NDDs, we selected 60 patients with intellectual disability (ID), epilepsy, autism, dysmorphic features and/or malformations.
Pathological genomic imbalances were detected in 23 out of 60 patients (38%). Besides CNVs associated with distinct clinical entities (15 patients, 65%), rare CNVs were identified in 8 patients (35%) with the following genomic distribution: chromosome 3 – two patients, chromosome 9 – two patients, chromosomes 1, 11, 12, 13, and 16 – one case each. The rare CNVs ranged between 0.97 to 11.5 Mb (median of 4.78 Mb) with 4 deletions (9p13.1p13.3, 9q34.11q34.12, 11q13.5q14.1, 16q24.1q22.3) and 5 duplications (1q21.3q22, 3p26.3, 3p26.3p26.2, 12q24.31q24.33, 13q11q13.3). Some of these rare CNVs (e.g. 9p13.1p13.3 and 16q24.1q22.3) partially overlap syndromic regions suggesting the possibility of distinct clinical disorders. Others, have just recently been reported in association with NDDs, such as 3p26.3 duplication covering CHL1.
The data illustrates the utility of array-CGH to bring new information regarding rare CNVs contribution to NDDs, thus paving the way for defining new clinical entities.

**Acknowledgments**
This poster was realized within Core Programme, supported by ANCSI, Projects no PN 09.33.02.03 and PN 16.22.05.01.

### 1.P70

### A novel frameshift mutation in the NLRP7 gene in a Turkish patient with molar pregnancy and recurrent miscarriage

H. Betül Gerik Çelebi, Esra Çolak, F.sirri Çam
Manisa Celal Bayar University Faculty of Medicine, Medical Genetics, Manisa, Turkey
**Correspondence:** H. Betül Gerik Çelebi
*Molecular Cytogenetics* 2017, **10(Suppl 1):**1.P70

**Introduction**
Recurrent pregnancy loss is the consecutive loss of 3 or more pregnancies. Pregnancy loss occurs in approximately 15% of all pregnancies, and recurrent loss in approximately 1%, depending on various factors such as hereditary thrombophiles, autoimmune factors, endocrine factors, anatomic factors, infections and genetic causes. However, in most of the cases, no etiologic cause can be found. We present a married couple with recurrent pregnancy loss and a history of molar pregnancy.

**Methods**
Chromosome analysis was performed from peripheral blood samples according to the appropriate protocol. Twenty metaphases stained with the giemsa trypsin banding technique were evaluated according to the 2016 International System for Human Cytogenetic Nomenclature (ISCN). A thrombophilia panel was studied and NLRP7 gene sequence analysis, including all coding exons and exon-intron boundaries was performed.

**Results**
Chromosome analysis showed a normal constitutional karyotype (46, XY and 46, XX) in the couple. There was no genetic change that would predispose to hereditary thrombophilia. Sequence analysis revealed a homozygous mutation in the NLRP7 gene NM_206828.3: c.1557_1557delA; (p.His520Thrfs*46) in the female, but not in the male.

**Conclusion**
In this study a novel NLRP7 mutation is reported. According to In silico analysis softwares, this mutation causes a premature stop codon and therefore it could be the cause of the molar pregnancy. Recurrent hydatidiform mole, 1(HYDM1, OMIM#231090) is an autosomal recessive inherited disorder characterized by trophoblastic hyperplasia, hydropic placental villi and no embryo. HYDM1 is due to mutations in the NOD-like receptor proteins (NLRP7) gene mapped to 19q13.42 and plays a role in several inflammatory pathways. Repeated hydatidiform mole should be supported by molecular studies.

### 1.P71

### Variable expressivity of the TPTPS SD4 phenotype in a family with segregation of LMBR1 duplication from a mosaic father to his daughter

Claudia Cesario [1], Cristina Gorgone [2], Barbara Torres [1], Marina Goldoni [1], Teresa Mattina [2], Laura Bernardini[1]
[1]Irccs "casa Sollievo Della Sofferenza", Mendel Laboratory, San Giovanni Rotondo, Italy; [2]University of Catania, Medical Genetics, Department of Pediatrics, Catania, Italy
**Correspondence:** Laura Bernardini
*Molecular Cytogenetics* 2017, **10(Suppl 1):**1.P71

The genomic region ZRS is a limb-specific cis-regulator of the gene SHH (OMIM: *600725) localized in intron 5 of the LMBR1 gene (OMIM: *605522), about 800 kb from SHH. Duplications of this region are associated with Syndactyly type IV (SD4), characterized by complete cutaneous syndactyly of all fingers often accompanied with polydactyly, and TriPhalangeal Thumb–Polysyndactyly Syndrome (TPTPS) consisting of duplicated triphalangeal thumbs, syndactylous fingers 3–5, and normal index fingers. SD4 and TPTPS are transmitted in an autosomal dominant manner with variable expression and represent a phenotypic continuum. We report a family with two affected individuals, father and daughter: the daughter showed a typical TPTPS, while the father had cutaneous syndactyly of 4–5 fingers on the right hand and syndactyly of 3–5 fingers on the left. SNP-array analysis of the daughter detected a microduplication of 268 kb in the region 7q36.3, overlapping with other cases already described in the literature and associated with SD4 and TPTPS. Real-Time PCR analysis on the trio suggested a de novo origin of the microduplication. However, the presence of limb anomaly in the father suggested a genetic cause segregating in the family. Therefore, SNP-array and a locus-specific FISH analysis, both able to detect diluted mosaicism, were extended to the parents, demonstrating that the father was mosaic for the same microduplication (15%), justifying his milder phenotype, not completely overlapping with the typical SD4/TPTPS spectrum. This family highlights that phenotypic variability could be due to mosaicism for a genetic defect. The detection of mosaicism is not only useful to better understand the genotype-phenotype correlation of limb anomalies, but also to give a correct recurrence risk to the family members.

### 1.P72

### Three new cases of interstitial microdeletion 4p16.3 contributing to a genotype phenotype correlation of WHS critical region of deletion

Laura Bernardini [1], Maria Cristina Digilio [2], Francesca Clementina Radio [2], Fabio Acquaviva [3], Cristina Gorgone [4], Diana Postorivo [5], Barbara Torres [1], Viola Alesi [2], Anna Maria Nardone [5], Teresa Mattina [4], Gioacchino Scarano [3], Antonio Novelli [2], Bruno Dallapiccola [2]
[1]Casa Sollievo Della Sofferenza Foundation, Mendel Laboratory, San Giovanni Rotondo (fg), Italy; [2]Bambino Gesù Children's Hospital, Medical Genetics Unit, Rome, Italy; [3]A.o.r.n, Gaetano Rummo, U.o.s.d. Medical Genetics, Benevento, Italy; [4]Catania University, Medical Genetics, Department of Pediatrics, Catania, Italy; [5]Policlinico Tor Vergata, U.o.c. Medical Genetics Laboratory, Rome, Italy
**Correspondence:** Laura Bernardini
*Molecular Cytogenetics* 2017, **10(Suppl 1):**1.P72

Wolf–Hirschhorn syndrome (WHS) is a well-known contiguous gene syndrome due to 4p16.3 deletion. The phenotype is characterized by typical facial features ('Greek warrior helmet'), intellectual disability, pre- and postnatal growth retardation, seizures and additional findings such as skeletal anomalies, congenital heart defects, midline defects, ocular and renal anomalies. These malformations are more frequent in the patients with larger deletions, involving a large portion of 4p16 chromosome region.
In the last several years, the characterization of patients with microarray platforms allowed to elucidate the role of some genes mapping to 4p16. WHSC1 is responsible for different WHS clinical features, whereas LETM1 is the candidate gene for seizures.
We report on three new patients with small interstitial deletions mapping to 4p16.3: two (P1 and P2) with deletions very similar for localization and extent and both presenting with mild intellectual disability, growth retardation, microcephaly and dysmorphic features, the other (P3) with a deletion just partially overlapping to the others and showing intellectual disability, growth retardation, microcephaly, partial atrioventricular canal and the typical WHS facial features. All deletions involved WHSC1 gene, but in Patient 3 the deletion also encompassed the more proximal genes NELFA, POLN and miRNA943. Interestingly, among the three affected children herein reported, this patient presented with a more severe malformative phenotype including a congenital heart defect.
These three new cases of interstitial deletions 4p16.3 highlight that WHSC1 is not sufficient per se to generate the full clinical spectrum, in particular the facial gestalt, of WHS. Moreover, it confirms that a more proximal region of deletion, mapping approximately to 2 Mb from the subtelomeric region is causative of congenital malformations, such as heart defects, shown by WHS patients with larger deletions.

### 1.P73

### A novel insertion from chromosome 18 to chromosome 15 with a 183Kb 18q deletion

Rosário Pinto Leite [1], Marta Souto [1], Pedro Botelho [1], Fernanda Pereira [2], Bárbara Marques [3], Hildeberto Correia [3], Osvaldo Moutinho [4], Márcia Martins [5]
[1]Centro Hospitalar De Trás-Os-Montes E Alto Douro, Cytogenetic Laboratory, Vila Real, Portugal; [2]Centro Hospitalar Do Nordeste, Pediatric Service, Mirandela, Portugal; [3]Instituto Nacional Doutor Ricardo Jorge, Cytogenetic Unit, Human Genetic Department, Lisboa, Portugal; [4]Centro Hospitalar De Trás-Os-Montes E Alto Douro, Gynecology / Obstetrics Service, Vila Real, Portugal; [5]Centro Hospitalar De Trás-Os-Montes E Alto Douro, Genetic Consultation, Vila Real, Portugal
**Correspondence:** Rosário Pinto Leite
*Molecular Cytogenetics* 2017, **10(Suppl 1):**1.P73

Chromosome 18q- syndrome is a rare chromosomal disorder with an incidence of 1 in 40 000 live births. The phenotype is highly variable, depending on the amount of deleted genetic material, and is characterized by mental retardation, developmental delay, hypotonia, seizures, obesity, abnormal behavior, short stature and craniofacial dysmorphisms.
We report a patient with mental retardation, dysmorphic features, hypotonia, growth retardation, severe expressive speech delay and Duane syndrome, with an insertion of 18q in 15q causing a 183Kb deletion in18q.
Cytogenetic and SNP array analysis showed a female karyotype presenting a de novo rare chromosome rearrangement: an insertion of the 18q21q23 on the 15q22 region, with deletion 18q12.3 (chr18:42,484,980-42,667,966, [GRCh37]), involving only the MIR4319 and SETBP1 genes.
There are few reports of 18q12.3 deletion associated with mild dysmorphic features, mental retardation and impairment of expressive language. To our knowledge this is the smallest deletion described, involving two genes: SETBP1 and a microRNA (MIR4319). SETBP1 gene is associated to expressive speech delay. The authors present a literature review of 18q12.3 deletion.

### 1.P74

### An apparently balanced complex translocation involving chromosomes 1 8 and 9 in a 3 year old infant

Rosa Lobo Valentín [1], América De León Rodríguez [1], María Talavera Yagüez [2], Patricia De La Fuente Alonso [1], Álvaro Sánchez [3], Carles Garrido Fusté [4], Esther Geán [4], Albert Torrents [4], Eva Domínguez Bernal [5], Celia Reig Del Moral [5], Guadalupe Ruiz [1]
[1]Río Hortega University Hospital, Clinical Analysis Service, Cytogenetics Unit, Valladolid, Spain; [2]Ramón Y Cajal University Hospital, Medical Genetics Department, Madrid, Spain; [3]General Hospital, Anatomical Pathology Service, Segovia, Spain; [4]Reference Laboratory, Genetics Division, Barcelona, Spain; [5]General Hospital, Pediatrics Service, Segovia, Spain
**Correspondence:** Rosa Lobo Valentín
*Molecular Cytogenetics* 2017, **10(Suppl 1):**1.P74

A 3-year-old male infant, was referred to our Cytogenetics Unit due to his short stature and phenotypic features. The child was born after a spontaneus conception and a pregnancy with no sign of abnormalities (low risk prenatal screening test and no ultrasonographic alterations), so no prenatal genetic study was carried out.
The karyotype in peripheral blood (PB) lymphocytes showed chromosome abnormalities in many regions, including in chromosomes 1,8 and 9, in a three-way balanced translocation. This finding was confirmed by the multiple FISH (M-FISH) technique.
Investigations by array-based comparative genomic hybridization (array-CGH) revealed a 6.7 Mb interstitial deletion on the short arm of chromosome 8 in the region 8p11.22 - 8p12. This de novo interstitial deletion in 8p involves 37 genes. There is no evidence of this constitutional deletion being previously described in the literature.
The karyotypes of the parents and the brother were normal. The couple was recomended to attend a genetic counselling appointment and to undergo prenatal diagnosis testing for future pregnancies, despite the low risk of recurrence.
This type of chromosomal rearrangements, involving 3 breakpoints, is a very rare occurrence in the population. Either the interstitial loss of 8p arm alone or such interstitial loss plus the triple translocation are likely to be responsible for the child's pathology.
This case has shown once again the necessary contribution of each cytogenetic technique to a comprehensive approach of any disease.
Through this work, the mechanisms that lead to the generation, the selection and the stabilisation of such complex karyotypes —within cells and human tissues— are discussed.

**Consent to publish:** The authors confirm that written informed consent was received by the patient for publication.

### 1.P75

### Moaicism detection by conventional cytogenetic techniques

América De León Rodríguez [1], Rosa Lobo Valentín [1], María Talavera Yagüez [2], Patricia De La Fuente Alonso [1], Gonzalo Quesada Segura [3], Guadalupe Ruiz Martín [4]
[1]Río Hortega University Hospital, Cytogenetics Unit, Valladolid, Spain; [2]Ramón Y Cajal University Hospital, Medical Genetics Service, Madrid, Spain; [3]Río Hortega University Hospital, Obstetrics And Gynaecology Service, Valladolid, Spain; [4]Río Hortega University Hospital, Clinical Analysis Service, Valladolid, Spain
**Correspondence:** América De León Rodríguez
*Molecular Cytogenetics* 2017, **10(Suppl 1):**1.P75

During the years 2015 and 2016, our Cytogenetics Unit detected 30 cases of mosaicism, 7 of them in prenatal samples and the remaining 23 in peripheral blood.
We will discuss the technical limitations of detecting mosaicism in different tissues, the impact of detection and non-detection, implications of detecting mosaicism prenatally and after birth, and the issues concerning genetic counselling

### 1.P76

### Two siblings with alternate unbalanced recombinants derived from a paternal inv(6)(p24q27): clinical and genetic characterization

Ascensión Vera-Carbonell[1], Lidia Rodríguez[2], Juan Antonio Bafalliu-Vidal[1], María Juliana Ballesta-Martínez[2], Gloria Soler-Sánchez[1], Vanesa López-González[2], María Jose Sánchez-Soler[2], Juana Abellán-Moreno[1], Ana Teresa Serrano-Antón[2], Isabel López-Expósito[1], Encarna Guillen-Navarro[2]
[1]Hospital C.U. Virgen de la Arrixaca Sección Citogenética. Centro de Bioquímica y Genética Clínica. Murcia-Spain; [2]Hospital C.U. Virgen de la Arrixaca Sección Genética Médica. Servicio de Pediatría. Murcia-Spain
*Molecular Cytogenetics* 2017, **10(Suppl 1):**1.P76

Carriers of chromosome 6 pericentric inversion (p24q27) have an increased risk of chromosomally unbalanced offspring due to the size of the chromosome and the inverted segment. Cross-overs within the inversion during the gametogenesis of heterozygote carriers produce two recombinant products which are compatible with survival to term.
We report on the clinical, cytogenetic, and molecular analysis of two siblings with discordant phenotypes who were found to each have a different recombinant 6 chromosome arising from a paternal inv(6)(p24q27).
Conventional cytogenetic studies in the Patient 1 detected a rec(6)del(6p)inv(6)(p24q27). She was born with hydrocephaly secondary to Dandy-Walker malformation prenatally detected, cerebellar vermix and corpus callosum agenesis. OFC at birth was 39 cm (>99th centile), clinical phenotype with exophthalmos, micrognatia, protruding tongue, hypotonia, generalized hyperreflexia and proximally impantede thumbs. She died at 1-month of age due to respiratory distress. Years later, a rec(6)dup(6p)inv(6)(p24q27) was postnatally found in the step-brother (Patient 2), which was cytogenetically undetectable and identified by oligo array-CGH (60 K). Array data defined the unbalanced segments, a duplication of ~9.3 Mb (6p24.3-pter) and a deletion of ~3.6 Mb (6q27-qter). He presented with less severe brain anomalies, with partial hypoplasia of corpus callosum and mild colpocephaly (supratentorial ventriculomegaly of posterior predominance). He was born at term, with normal growth parameters. Clinically he presented with mild language delay, divergent strabismus and bilateral criptorquidism.
Since that Patient 1 was identified as having the “opposite” chromosomal imbalance of Patient 2, the phenotypic characteristics associated with these genotypes are compared in this family. In addition, we compare similar genetic findings and phenotype in other patients reported in the literature, and discuss about the role of FOXC1 and other genes associated with the central nervous system malformations in these patients.
**Consent to publish:** The authors confirm that written informed consent was received by the patient for publication.

### 1.P77

### SRY-negative 46,XX male with ovotesticular disorder of sex development and cardiac defects due to a 3-Mb deletion in 15q

Gianna Carvalheira[1], Mariana Moysés-Oliveira[1], Renata Ueta[2], Magnus Dias da Silva[3], Maria Isabel Melaragno[1]
[1]UNIFESP Morphology and Genetics, Sao Paulo, Brazil; [2]UNIFESP Morfology and Genetics, Sao Paulo, Brazil; [3]UNIFESP Medicine, Sao Paulo, Brazil
*Molecular Cytogenetics* 2017, **10(Suppl 1):**1.P77

The physiopathology of disorders of sex development (DSD) has not been completely elucidated. Testicular tissue development can occur in 46,XX patients who usually present heterogeneous male external genitalia with various degrees of genital ambiguity and azoospermia. We report on a 19-year-old male born with perineal hypospadias, undescended gonads, pectus excavatum, and aortic coarctation with a 46,XX karyotype. Orchiectomy of right gonad was performed during infancy and his cardiac defect was surgically corrected at 11 months. At first clinical evaluation, he was at Tanner 4 without androgen reposition need. No Müllerian structures were evidenced by ultrasound or tomography. Reviewed right gonadal biopsy confirmed ovotesticular DSD 46,XX. Molecular assays showed SRY negative, no mutations in RSPO1 and SOX9; and no copy number variation in WNT4, NR5A1 and SOX9 by MLPA analysis. SNP-array technique showed a 3-Mb deletion and the result was arr[hg19]15q26.2(95127653_98146649) × 1,15q26.2q26.3(98263048_98618586) × 1. In silico analysis showed that the 15q26.2 deletion is a gene-poor region, containing four coding genes, one microRNA and three pseudogenes. The microRNA-1469 is highly expressed in testis with no predicted mRNA targets associated to DSD. One of the gene (NR2F2) is highly expressed in ovary, uterus, vagina and artery-coronary. Although this 3-Mb region is gene-poor, it seems to be a critical regulatory one, containing more than 15,000 transcript factors binding sites (TFBS), with around 300 predicted binding sites to SRY and more than 100 to SOX5 and SOX9. In addition to, this deletion seems to interfere in the three-dimensional cis interaction between the FES and IGF1R genes, as well as in the cis interaction between the NR2F2 and IGF1R genes. Therefore, this 3-Mb 15q26.2 region probably has a regulatory role being important for sexual and cardiac development.

### 1.P78

### Patient with de novo 4q31.21 deletion and 9q34.2 duplication. A case report

Matias Perez Sanchez[1], Susana Garcia Linares[1], Amanda Gonzalez Ramirez[2], Adelardo Mora Guijosa[1], Margarita Martinez Atienza[1]
[1]Hospital Virgen de las Nieves Genetics, Granada, Spain; [2]Instituto Biosanitario de Granada FIBAO, Granada, Spain
*Molecular Cytogenetics* 2017, **10(Suppl 1):**1.P78

Structural aberrations of chromosomes are associated with various syndromes, although there are a lot of chomosomal gains and losses that are not yet associated with a specific syndrome. Most of the cases are the novo, few cases are due to inherited unbalanced rearrangements.
We present a thirteen months-old boy with microcephaly, trigonocephaly, plagiocephaly and speech and motor delay. He was born at 36 weeks of gestation in a twin pregnancy obtained by in vitro fertilization. The parents were healthy. His twin brother had no detectable pathology.
Karyotype in leucocytes was normal (46,XY). Comparative Genomic Hybridization (CGH-Array) with the Nimblegen CGX Cytogenetic Microarrays platform, supplied by PerkinElmer, was performed for the patient, parents and brother.

The CGH-Array was normal in both parents and brother, but the patient presented a deletion of 3,04 Mb in 4q31.21 and a duplication of 212,03 Kb in 9q34.2 (arr[hg19] 4q31.21(143,272,775-146,310,106)x1,9q34.2(137,103,263-137,315,288)x3 Abnormal Male). Both duplication and deletion are “de novo” and not previously described. The 4q31.21 deletion includes 14 genes (11 OMIM). The 9q34.2 duplication includes the RXRA OMIM gene, without triplo-sensitivity previously described.
The pathologic potential of chromosome aberrations detected “de novo” can be difficult to determine, and it is necesary to have the phenotype of the patient in mind as well as the genes involved in the deletion/duplication. In our case we believe that both detected chromosomal alterations, alone or in conjunction, are responsible for the patient's phenotype.
**Consent to publish:** The authors confirm that written informed consent was received by the patient for publication.

### 1.P79
**A rare case report of Langer-Giedion syndrome/ Trichorhinophalangeal syndrome (TRPS) type II and Cornelia de Lange syndrome 4**
Nikoletta Selenti, Maria Tzetis, Maria Braoudaki, Eirini Tsoytsoy, Sofia Kitsioy-Tzeli, Helen Fryssira
[1]Athens University, School Of Medicine Department Of Medical Genetics Athens-Greece
**Correspondence:** Nikoletta Selenti
*Molecular Cytogenetics* 2017, **10(Suppl 1):**1.P79

This abstract is not included here as it has already been published.

### Tumour Cytogenetics

### 2.P1
**WAGR region deletions size and position do mean**
Rena Zinchenko [1], Tatyana Vasilyeva [2], Anna Voskresenskaya [3], Olga Khlebnikova [2], Nadezhda Pozdeyeva [3], Yuliya Kozlova [4], Nadezhda.V. Shilova [4], Andrey Marakhonov [5]
[1]Federal State Budgetary Institution «research Center For Medical Genetics»,pirogov Russian National Research Medical University, Laboratory of Genetic Epidemiology, Moscow, Russia; [2]Federal State Budgetary Institution «research Center For Medical Genetics», Laboratory of Genetic Epidemiology, Moscow, Russia; [3]Cheboksary Branch of S. Fyodorov Eye Microsurgery Federal State Institution, Laboratory of Genetic Epidemiology, Cheboksary, Russia; [4]Federal State Budgetary Institution «research Center For Medical Genetics», Laboratory of Cytogenetic, Moscow, Russia; [5]Federal State Budgetary Institution «research Center For Medical Genetics», Moscow Institute of Physics And Technology, Laboratory of Genetic Epidemiology, Moscow, Russia
**Correspondence:** Rena Zinchenko
*Molecular Cytogenetics* 2017, **10(Suppl 1):**2.P1

**Introduction**
WAGR syndrome (OMIM#194072) is caused by deletions of 11p13 encompassing the PAX6 and WT1 genes. Almost all cases are sporadic. Four WAGR classical clinical signs rare present in complete combination. The 1.6 Mb region is supposed to be critical for all classical WAGR criteria, though clinical diagnosis is based on identified 11p13 deletion, aniridia in combination with at least one other feature.
**Aims**
Study of association between clinical criteria of WAGR syndrome and 11p13 deletion length and position.
**Methods**
MLPA-, FISH.
**Results**
14 Russian patients with WAGR region deletions, 4 males and 10 females from 2 months to 40 years old, were studied. 12 cases were sporadic while 1 was familial. Unilateral nephroblastoma developed in 6 patients within 2 first years of life, 4 adult patients had no tumor, 4 kids under 3 years old had not developed it (probably yet). 3 out of 6 patients with WAGR syndrome and the largest deletions (>7.5 Mb) showed unilateral nephroblastoma and severe neurological deficit combined with mental retardation. The rest of the patients with congenital aniridia or WAGR syndrome associated with narrower deletions demonstrated milder cognitive and neurologic status. Unilateral nephroblastoma developed in 1 out of 5 patients carrying the narrowest deletions with the same right breakpoint at the WT1 level (0.7–2.2 Mb). 4 patients under 3 years old are still in the high oncological risk group despite the lack of tumor, as they possess gross deletions of critical region. They require rigorous monitoring.
**Conclusion**
Risk for Wilms’ tumor development in patients with 11p13 deletion in a cohort of Russian patients is 43% – 71%. Prognosis for the Wilms' tumor development and cognitive deficit worsens with deletion region increase towards centromere from WT1 locus.
The research was partially supported by grants RFFR 17-04-00288; 15-04-01859, RSF 17-15-01051.

### 2.P2
**The importance of conventional cytogenetic analysis in monitoring the progression of haematological malignancies**
Li Eng Loo [1], Lai Ching Lau [1], Alvin Soon Tiong Lim [1], Sim Leng Tien [2]
[1]Singapore General Hospital, Molecular Pathology, Singapore, Singapore; [2]Singapore General Hospital, Molecular Patholgy, Singapore, Singapore
**Correspondence:** Li Eng Loo
*Molecular Cytogenetics* 2017, **10(Suppl 1):**2.P2

**Aims**
To highlight the importance of cytogenetic investigations in detecting disease progression after cancer treatment.
**Methods**
Four leukemic patients were treated but subsequently developed new haematological malignancies. A variable number of tandem repeat test was run to correlate the degree of transplant engraftment for patients who did a bone marrow transplant (BMT).
**Results**
Case 1: A female patient in 2012 diagnosed with Acute Lymphoblastic Leukaemia (ALL) had a t(9;22)(q34;q11.2) rearrangement. She underwent a BMT in 2013 and remained in remission until 2015. Despite VNTR showing 100% donor engraftment, cytogenetics revealed that her bone marrow (BM) had undergone transformation to Myeloproliferative Disorder.
Case 2: A female patient with ALL in 1995 underwent a BMT and was in remission with 100% donor cells. In 2015, a t(12;18) rearrangement detected in her bone marrow. This was not seen previously, suggesting either disease progression or donor cell constitutional rearrangement. Donor karyotype was recommended to rule out constitutional rearrangement but was not forthcoming.
Case 3: A female patient with Acute Myeloid Leukaemia in 1995 had a complex karyotype. She had a BMT in 1997 and was in remission until a relapse in 2007. A second BMT in 2009 showed 100% donor engraftment. In 2014, cytogenetics detected a completely different karyotype, suggesting malignant transformation.
Case 4: A male patient admitted in 2013 for Severe Aplastic Anaemia and suspected MDS had a complex karyotype. He had a sex-mismatched BMT in 2014. His BM never reached 100% engraftment as the karyotype showed persistence of the malignant clone and normal donor cells. Three months later, cytogenetics detected disease progression.
**Conclusion**
BMT is the only cure for haematological malignancies. Routine karyotyping is necessary to detect malignant transformation. This could alter the patient treatment. The study shows that 100% donor engraftment does not necessarily mean 100% treatment success.

### 2.P3

### Conventional cytogenetic analysis in chronic lymphocytic leukemia using two different culture procedures

Anastasia Athanasiadou [1], Maria Gkaitatzi [1], George Papaioannou [1], Aliki Xochelli [2], Olga Asteriou [1], Zografia Lazarou [1], Niki Stavroyianni [1], Vasiliki Douka [1], Achilleas Anagnostopoulos [1]
[1]G.papanikolaou Hospital, Haematology, Thessaloniki, Greece; [2]Certh, Institute of Applied Biosciences, Thessaloniki, Greece
**Correspondence:** Anastasia Athanasiadou
*Molecular Cytogenetics* 2017, **10(Suppl 1):**2.P3

Cytogenetic analysis is not routinely performed in chronic lymphocytic leukemia (CLL), but this may likely change, in view of recent reports highlighting complex karyotype (CK) as a poor predictive marker independently of the presence of the deletion 17p. We investigated whether two different culture procedures can improve the number of metaphases and the detection rate of chromosomal abnormalities in CLL. A total of 674 samples were studied from patients with CLL (310 of 466 patients with available data were at diagnosis, 39/310 needed treatment). Parallel cultures of MNC were set up with the addition of either phytohemagglutinin and Interleukin 2 (PHA/IL-2) or an immunostimulatory CpG-oligonucleotide DSP30 and IL-2 (CpG/IL-2). Overall, 639/674 (94.8%) samples had evaluable metaphases. Significantly increased metaphases were observed in PHA/IL-2 632/674 (93.8%) versus in CpG/IL-2 560/674(83%) ($p < 0.0001$). Clonal abnormalities were identified in 290 cases (45.38%). Abnormal karyotypes were observed more frequently in cultures with CpG/IL-2 (242/639, 37.9%) than these with PHA/IL-2 (192/639, 30%) $p = 0.003$. In 145/639 samples (22.7%), the aberrant clone was detected in both cultures. Clonal aberrations were detected in only one culture, either in CpG/IL-2 or PHA/IL-2 in 97 (15.18%) and 47 (7.3%) samples, respectively. Trisomy 12 was the most common abnormality 95/639 cases (13.7%); del(13q) was detected in 31/639 cases (4.5%); del(11)(q23) in 44/639 (6.9%) and del(17p)/-17 in 40/639 (5.8%) cases. CK revealed in 55/639 (8.6%) cases: in 24 of these cases, the CK was detected in both cultures and in the remainder only in one culture, 20 in CpG/IL-2 and 11 in PHA/IL-2($p = 0.29$). In conclusion, CpG/IL-2 stimulation increases the detection rate of chromosomal abnormalities in CLL compared with the PHA/IL-2 technique. The combination of both cultures can provide more comprehensive genetic information for CLL.

### 2.P4

### Expression studies in the bone marrow of patients with Shwachman Diamond Syndrome (SDS) and deletion of the long arm of chromosome 20 encompassing the EIF6 gene relation with benign prognosis

Roberto Valli [1], Marco Fabbri [2], Annalisa Frattini [3], Lucia Nacci [4], Gabriele Babini [5], Jacopo Morini [5], Francesco Pasquali [1], Emanuela Maserati [1]
[1]University of Insubria, Medicine And Surgery Department, Varese, Italy; [2]European Insitute of Oncology, Unit of Haematopathology, Milano, Italy; [3]National Council of Research, Irgb, Milano, Italy; [4]University of Pavia, Department of Molecular Medicine, Pavia, Italy; [5]University of Pavia, Physics Department, Pavia, Italy
**Correspondence:** Roberto Valli
*Molecular Cytogenetics* 2017, **10(Suppl 1):**2.P4

Shwachman-Diamond Syndrome (SDS) is a rare autosomal recessive disorder with peculiar clinical signs and a high risk of developing Myelodysplastic Syndrome (MDS) and/or Acute Myeloid Leukaemia (AML). SDS is caused by mutation of SBDS gene in >90% of patients. The most frequent chromosomal anomalies observed in the bone marrow (BM) of SDS patients are an isochromosome i(7)(q10) and an interstitial deletion of chromosome 20, del(20)(q), and they are generally considered benign prognostic signs. EIF6 gene is located on the long arm of chromosome 20 and produces a partner in SBDS pathways. We ascertained by array CGH (aCGH) that in 11/11 patients carrying the del(20)(q), the gene EIF6 is invariably lost and we speculated that its haploinsufficiency is related to a better "fitness" of the clone carrying the deletion.
To confirm our hypothesis, we collected BM RNA from 3 patients carrying the del(20)(q) at various clonal percentages, and 2 patients carrying other different chromosome anomalies. We performed quantitative real-time PCR for the study of the expression of EIF6 gene and we also performed an array-based whole transcriptome analysis in order to focus on possible differences between the patients carrying the del(20)(q) and the others.
Hypoexpression of EIF6 was demonstrated. Whole transcriptome analysis showed in patients with the del(20)(q) an overall expression pattern of BM more similar to the normal controls (in line with the clonal percentages) than patients that do not carry the del(20)(q). This preliminary study involved only few patients, but we conclude that EIF6 loss due to the del(20)(q) clonal deletion really represents a "rescue" mechanism for the clone carrying the anomaly.

### 2.P5

### Molecular cytogenetic detection of the ASXL1 gene alterations in patients with 20q deletion

Jana Brezinova [1], Iveta Sarova [1], Sarka Ransdorfova [1], Karla Svobodova [2], Halka Lhotska [2], Silvia Izakova [2], Lenka Pavlistova [2], Zuzana Zemanova [2], Jana Markova [3], Jaroslav Cermak [4], Anna Jonasova [5], Kyra Michalova [2]
[1]Institut of Hematology And Blood Transfusion, Department of Cytogenetics, Prague, Czech Republic; [2]Institute of Medical Biochemistry And Laboratory Diagnostics, General University Hospital And First Faculty of Medicine, Charles University, Center of Oncocytogenetics, Prague, Czech Republic; [3]Institut of Hematology And Blood Transfusion, Department of Molecular Genetics, Prague, Czech Republic; [4]Institut of Hematology And Blood Transfusion, Clinical Department, Prague, Czech Republic; [5]General University Hospital And First Faculty of Medicine, Charles University, 1st Medical Department, Prague, Czech Republic
**Correspondence:** Jana Brezinova
*Molecular Cytogenetics* 2017, **10(Suppl 1):**2.P5

Alterations of the AXSL1 gene which maps to chromosome sub-band 20q11.21 play an important role in the pathogenesis of hematologic malignancies. In contrast to deletion 20q - del(20q) - recurrent mutations in this gene, most frequently located within the exon 12, are generally associated with poor prognosis. The aim of our study was to determine the frequency of ASXL1 gene alterations in bone marrow cells of patients with isolated del(20q) and to characterize the breakpoints with molecular cytogenetic techniques. Fluorescence in situ hybridizations (FISH) with locus specific probes for 20q12 and 20q13 regions (Kreatech, MetaSystems) confirmed the cytogenetically observed deletions of 20q in a cohort of 34 patients (25 male, 9 female, median age 68 years) with hematologic diseases (32 x myeloid, 2 x lymphoid). Metaphase FISH mapping with set of 4 bacterial artificial chromosome (BAC) probes (BlueGnome) distributed in 20q11.21 and 20q13.2 and ASXL1 BA Probe Set (Empire Genomics) were used for determination of the breakpoints. According to the FISH results, three groups of patients were established: in 11 patients (32%) the proximal breakpoint in the ASXL1 gene was found, in 10 patients (30%) this gene was deleted, in 13 patients the ASXL1 gene remained unchanged with the proximal breakpoint below this gene. Array comparative genomic hybridization (Illumina, Agilent) performed on DNA samples of bone marrow cells of patients with ASXL1 breakpoint confirmed the high heterogeneity of the breakpoints with deletion of the exon 12 in all studied cases. In conclusion, the ASXL1 gene was altered in 21 out of 34 patients (62%). The association of ASXL1 alterations with the worse prognostic risk was proved in several studies and relations with clinical data should be studied further. Supported by MHCR project 00023736, RVO-VFN64165, GACR P302/12/G157, ProgresQ28.

### 2.P6

### Follow up cytogenetic studies on a chronic myeloid leukemia case developing into acute myeloid leukemia after treatment with imatinib

Wilma Kroes [1], Elke Den Ouden [1], Mieke Baasten [1], Corine Buitenhuis [1], Jean-Louis Kerkhoffs [2], Peter Von Dem Borne [3]
[1]Leiden University Medical Center, Clinical Genetics, Leiden, The Netherlands; [2]Haga Hospital, Hematology, The Hague, The Netherlands; [3]Leiden University Medical Center, Hematology, Leiden, The Netherlands
**Correspondence:** Wilma Kroes
*Molecular Cytogenetics* 2017, **10(Suppl 1):**2.P6

In 2006 a 34-year-old male presented with leukocytosis. Karyotyping of bone marrow revealed t(9;22)(q34;q11.2) in all metaphases, FISH was positive for BCR/ABL1 and he was diagnosed having CML. Treatment with imatinib followed and after one year no t(9;22) positive metaphases were found . At that time FISH showed 2.5% positivity for BCR/ABL1. An unexpected finding was monosomy 7 in 75% of metaphases. There were no clinical signs of MDS or AML. Treatment with imatinib was continued and after three years monosomy 7 was detected in 100% of metaphases and by FISH in 90% of interphase nuclei. FISH for BCR/ABL1 was negative. Cytology of bone marrow indicated AML and induction therapy was given. Subsequent. karyotyping of bone marrow showed the presence of 3 cell lines: 45,XY,-7[3]/47,XY,+Y[5],46,XY[22]. The patient received a double cord blood transplant in 2012. After 17 months of remission the patient relapsed. Monosomy 7 was present again, BCR/ABL1 was not detectable with FISH. After chemotherapy the patient received an allogeneic stem cell transplant **.
The patient had a second relapse of the AML in 2016. At this time cytogenetic investigation showed 3 related cell lines again and clonal evolution: 45,XY,t(1;7)(p13;p13),del(6)(q13),-7[11]/45,XY,del(6)(q13),-7,del(12)(p11.2)[12]/45,XY,del(6)(q13),-7,t(17;22)(q2?2;p11.2)[7].
Chemotherapy was started, but the patient died due to acute liver failure.
Conclusion: patients who present with monosomy 7 in Ph negative cells during imatinib treatment have a risk of developing AML. For this reason these patients need to be checked at regular intervals for cytogenetic or clinical sign of MDS or AML.
**See also
Cytogenetic studies on a chronic myeloid leukemia case developing into acute myeloid leukemia after treatment with imatinib.", Chromosome Research 2015 23:1 SUPPL. 1 (S99)
**Consent to publish:** The authors confirm that written informed consent was received by the patient for publication.

### 2.P7

### TET2 deletion in MDS patients

Helena Podgornik [1], Maja Kambič [2], Hermina Čibej [1], Matjaž Sever[1]
[1]Umc Ljubljana, Department of Haematology, Ljubljana, Slovenia;
[2]University of Ljubljana, Faculty of Pharmacy, Ljubljana, Slovenia
**Correspondence:** Helena Podgornik
*Molecular Cytogenetics* 2017, **10(Suppl 1):**2.P7

Tumor suppressor TET2 gene is located on 4q24. It codes for TET2 protein, an α-ketoglutarate/Fe(II)-dependent dioxygenase which regulates DNA demethylation. TET2 is one of the most frequently mutated genes in myelodysplastic syndromes (MDS). Nonsense, missense mutations, and small insertions/deletions lead to the loss of protein function and consequently to increased hematopoietic stem cell self-renewal and myeloid transformation. 6–26% of MDS patients exhibit the loss of TET2 function.
In 60 MDS patients del(4)(q24) was determined by FISH using commercially available SureFISH probe (Agilent). For DNA probe a specificity of 100% and a cut-off value of 5,7% was determined on normal samples.
Del(4)(q24) was detected in 8,3% (5/60) of samples. IPSS-R cytogenetic group was determined in all patients while IPSS-R risk group was determined in 55 patients only due to lack of data on cytopenias and bone marrow blast count. We observed no significant correlation between the gender and age distribution and the TET2 deletion. The distribution of patients regarding IPSS-R cytogenetic groups was similar to the general distribution in Slovenian MDS patients with 53,3% of the patients with a normal karyotype. TET2 deletion was found only in patients with chromosomal abnormalities previously confirmed by GTG banding. While TET2 deletion was significantly more frequent ($p = 0.042$) in cytogenetically poor groups, no association between the presence of deletion and IPSS-R risk group was found. The most interesting case was a patient with apparently balanced translocation involving 4q24 (46,XX,t(4;12)(q24;q14)[5]/46,XX[16]) which turned out to be unbalanced with confirmed TET2 deletion in 15,5% of cells.
We found that TET2 deletions are significantly more frequent in MDS patients with chromosomal aberrations with poor prognostic significance. FISH can be used not only to validate molecular results but also to detect TET2 deletions in cases with 4q rearrangements.

### 2.P8

### KMT2A rearrangement through translocation inversion of chromosomes 10 and 11

Ana Doplihar Kebe, Hermina Čibej, Karmen Dežman, Helena Podgornik
Umc Ljubljana, Department of Haematology, Ljubljana, Slovenia
**Correspondence:** Ana Doplihar Kebe
*Molecular Cytogenetics* 2017, **10(Suppl 1):**2.P8

Rearrangements involving the KMT2A (MLL) gene located at 11q23 are between the most common cytogenetic aberrations found in acute leukemia (AL) generally and particularly in M4/M5 acute myeloid leukemia (AML). The heterogeneity of MLL-rearranged AL is reflected by the identification of more than 50 different fusion partners of this gene. Non-random chromosomal rearrangements involving chromosomes 10 and 11 were identified, but with the type of rearrangements and breakpoints being variable. A significant proportion of these 10;11 abnormalities was shown to result in KMT2A-MLLT10 fusion. Here we present a case of 18 years old AML M5 male patient with a complex KMT2A rearrangement involving chromosomes 10 and 11.
FISH analysis (LSI MLL, Abbot) confirmed KMT2A rearrangement in 47% of cells. GTG banding revealed rearranged chromosomes 10 and 11 in all analyzed cells. On the bases of pattern obtained by WCP probes (WC10 and WC11, Leica), we assumed that reciprocal translocation between long arms of chromosomes 10 and 11 (t(10;11)(q22;q23)), followed by pericentric inversion of rearranged chromosome 10 with breakage in KMT2A of translocated 11 resulted in KMT2A- MLLT10 fusion gene. It is known that besides direct translocation this fusion can be also a consequence of translocation/inversion mechanism. Fusion of both genes was soon excluded by molecular genetics as well as by FISH (SureFISH MLLT10, Agilent Technologies). Metaphase FISH showed that 3'KMT2A was located on 10q11.1 as a consequence of paracentric inversion of the translocated chromosome 10.
The assigned chromosomal region 10q11 has some candidate oncogenes, but none of them known as KMT2A fusion partner and even more - none of them is involved in AML carcinogenesis. Although KMT2A rearrangements are known to be of poor prognostic significance, very early relapse in the patient encourages us to further explore the genetic background of the described rearrangement.

### 2.P9

### Spectrum of Standard and Rare Cytogenetic Abnormalities in 392 Indian AML Patients

Mayur Parihar [1], Manish Kumar Singh [2], Arun Sr [2], Neeraj Arora [3], Deepak Mishra [3], Sourabh Bhave [4], Jeevan Kumar [4], Vivek Radhakrishnan [4], Reena Nair [4], Arpita Bhattacharya [5], Shekhar Krishnan [5], Anirban Das [5], Vaskar Saha [5], Mammen Chandy [6]
[1]Tata Medical Center, Cytogenetics And Lab Haematology, Kolkata, India; [2]Tata Medical Center, Cytogenetics, Kolkata, India; [3]Tata Medical Center, Lab Haematology, Kolkata, India; [4]Tata Medical Center, Clinical Haematology, Kolkata, India; [5]Tata Medical Center, Peadiatric Onocology, Kolkata, India; [6]Ata Medical Center, Clinical Haematology, Kolkata, India
**Correspondence:** Mayur Parihar
*Molecular Cytogenetics* 2017, **10(Suppl 1):**2.P9

**Introduction**

There is little data available on cytogenetic profile of AML patients from India. Using a uniform cytogenetic testing strategy we describe cytogenetic profile of 392 (non APML) AML patients seen in a single centre from 2011–2016.

**Methods**

Karyotyping was performed on all the AML patients. FISH for specific probes were done on selective patients based on the analysis of morphology, imunophenotyping and karyotype results.

**Results**

Of the 392 AML patients, 83 were pediatric (≤18 yrs) and 309 adults with a median age of 36 years. Successful karyotype analysis was possible in 387 (98.2%) patients with 64.1% (248) showing abnormal karyotypes. Favourable risk cytogenetic profiles were more frequent in paediatric patients (31.4% vs 15.9% in adults). Intermediate risk cytogenetics was seen in 45.8% paediatric and 61.1% adult patients and adverse risk profile was seen in 22.8% paediatric and 23% adults. The t(8;21) accounted for 24.5% of abnormal karyotypes (n = 61). Inversion 16 was seen in 5.2% (n = 13),MLL rearrangement in 7.6% (19),3q abnormality 6.4% (n = 16),t(6;9) in 2% (n = 5) of abnormal karyotypes. Monosomy 7/del7q and monosomy 5 /del5q were seen in 31 patients and 22 patients of which 11 and 18 were complex respectively. Complex karyotypes were seen in 40 patients(16.1%) and monosomal in 7 (2.8%). Ph positive AML was seen in 2 patients. Hyperdiploidy was seen in 8 patients. Amplification of the MLL gene was seen in 3 patients. Rare translocations included t(2;11)(q37;q23),t(10;11)(p12;q23),t(6;11)(q27;q23),t(8;16)(p11.2;p13.3) and jumping translocations.

**Conclusions**

We report a lower frequency of normal karyotype AML and a higher frequency of t(8;21) compared to literature. Selective FISH analysis based on the clinicopathological features in the presence of karyotyping is a cost effective strategy to identify the spectrum of cytogenetic abnormalities in AML including rare abnormalities.

### 2.P10

### MDS with t(1;7)(p36;p12) A Case Report and Literature Review

Heather Williams, Loretta Brown
King's College Hospital/viapath, Cytogenetics, London, UK
**Correspondence:** Heather Williams (Williams.Heather.E@gmail.com)
*Molecular Cytogenetics* 2017, **10(Suppl 1):**2.P10

The PR/SET domain 16 (PRDM16) gene is located on chromosome 1 (1p36) and encodes a zinc finger transcription factor. PRDM16 has high sequence similarity to MDS1 and EVI1 complex locus (MECOM), and MECOM rearrangements are recurrent findings in myeloid malignancies. Haematological diseases with the t(1;3)(p36;q21) translocation have overlapping characteristics of the MECOM AML and MDS subgroup, a distinct entity in the World Health Organization (WHO) classification. Other chromosomal aberrations involving 1p36 may overlap similarly, and characterizing the distinct clinical presentation may have prognostic and therapeutic implications. Cases of AML and MDS with recurrent PRDM16 associated translocations without involvement of chromosome 3 include t(1;2)(p36;q22), t(1;3)(p36;q21), t(1;6)(p36;q15) and t(1;7)(p36;p12) as documented in the clinical literature. Herein we present the unique case of an MDS patient with a t(1;7)(p36;p12) evaluated for PRDM16 rearrangement.

### 2.P11

### Characterıstıcs of varıant translocatıons among 1324 Greek patıents wıth Chronıc Myelogenous Leukemıa

C Sambani [1], G Bardi [2], Kn Manola [1], G Papaioannou [3], E Gourgouveli [2], P Diamantopoulou [1], D Iakovaki [2], M Gaitatzi [3], M Kalomoiraki [1], A Anagnostopoulos [3], A Athanasiadou [3]
[1]Ncsr Demokritos, Laboratory of Health Physics, Radiobiology & Cytogenetics, Athens, Greece; [2]Bioanalytica-genotype Sa, Cytogenetics, Athens, Greece; [3]G. Papanicolaou Hospital, Hematology Department & Hct Unit, Thessaloniki, Greece
**Correspondence:** C Sambani
*Molecular Cytogenetics* 2017, **10(Suppl 1):**2.P11

Chronic Myelogenous Leukemia (CML) is defined by the Philadelphia chromosome (Ph) as a result of t(9;22)(q34;q11.2) and the generation of BCR-ABL1 fusion gene. Variant translocations are found in 3-10% of patients with newly diagnosed CML. They can be present either in a simple form, involving 22q11 and one additional breakpoint, or in a complex form, involving 22q11, 9q34 and at least one additional breakpoint. In variant translocations, the distribution of breakpoints exhibits a nonrandom pattern. Although the clinical and hematologic features of these cases are not distinct from those seen in standard t(9;22), however, the prognostic role of each individual variant translocation and its additional chromosomal abnormalities has not been elucidated yet.

The aim of this multicenter study is to determine the type and the frequency of variant Ph translocations and their additional chromosomal abnormalities in a large Greek cohort of CML patients.

The study included 1324 newly diagnosed CML patients. Among them, 60 (40 males, 20 females) exhibited a variant translocation in their karyotype (4,5%). A simple variant was found in 11 and a complex in 49. The most frequent variants were: t(2;9;22)(p13;q34;q11) (n = 3), t(6;9;22)(p21;q34;q11) (n = 3) and t(9;11;22)(q34;q13;q11) (n = 3). Additional chromosomal abnormalities were observed in 11 out of 60 patients (18,3%): +8, −Y, −7, −3, +Ph, i(17)(q10), del(5)(q12q33), add(21)(q22), t(6;14)(q23:q32), add(9)(q34), t(6;16)(p12;q22-24), del(6)(q21), t(1;12)(p32;q24). Twenty one out of 60 variant translocations have not been reported previously and ten of them exhibited involvement of new breakpoints: 3q11, 2q21, 5q14, 5q15, 5q21-22, 6q15, 11p11, 11q25, 12q13. Conventional cytogenetic analysis is the only appropriate methodology for the identification of variant translocations in CML. Reviewing of cytogenetic data during each patient's course of the disease is expected to point out the role of certain variants and additional abnormalities in prognosis.

### 2.P12

### Higher frequency of dicentric chromosomes in complex karyotypes of bone marrow cells of patients with acute myeloid leukemia (AML)

Iveta Sarova [1], Jana Brezinova [2], Sarka Ransdorfova [2], Zuzana Zemanova [1], Karla Svobodova [1], Silvia Izakova [1], Lenka Pavlistova [1], Cyril Salek [3], Kyra Michalova [1]
[1]Institute of Medical Biochemistry And Laboratory Diagnostics, General University Hospital, Center of Oncocytogenetics, Prague, Czech Republic; [2]Institute of Hematology And Blood Transfusion, Cytogenetic Department, Prague, Czech Republic [3]Institute of Hematology And Blood Transfusion, Clinical Department, Prague, Czech Republic
**Correspondence:** Iveta Sarova
*Molecular Cytogenetics* 2017, **10(Suppl 1):**2.P12

Complex karyotypes (CK) with dicentric chromosome (DC) often contain more than one DC. It is usually missed and considered as monosomy when a conventional or mFISH analyses are used. To prove it we revised monosomies in CK in bone marrow cells of 72 out of 607 adult patients with AML using FISH with centromeric and/or multicolor multi-centromeric probes.

DCs were detected in 24 patients already at the diagnosis. CK of these patients were revised using centromeric FISH as non-monosomy (n = 3); DC and monosomy (n = 12); DC, monosomy and new DC (n = 7) and DC and monosomy within polyploidy (n = 2). In other 48 patients, DC was newly identified in 27 of them. CK were found to be non-monosomy (n = 4); monosomy and new DC (n = 22) and monosomy with new DC within polyploidy (n = 1). Karyotypes of remaining 21 of 48 patients were assessed as CK without monosomy (n = 2); with confirmed monosomy (n = 17) and with monosomy within polyploidy (n = 2). 51 total (often chromosomes 7 and 18) and 54 partial (without centromere, often chromosomes 17 and 15) monosomies were found. DCs were often formed by chromosomes 17 and 20.

Frequency of DCs is higher than it was expected and FISH analyses were essential for their detection. We proved hidden DCs in 34 cases and confirmed monosomies in 63 out of 72 patients. The most frequently involved was chromosome 17 in both partial monosomies and DCs. In some cases, it was apparent that derivative monocentric chromosomes were primary dicentrics. DCs can induce chromosomal aberrations, new DCs, undergo variety of stabilization changes and can be also important step for development of unbalanced translocations associated with monosomy.

Supported by MHCR project 00023736, RVO-VFN64165, GACR-P302/12/G157, ProgresQ28.

### 2.P13

### Aberrations of chromosome 7 in myeloid malignancies

Sarka Ransdorfova [1], Jana Brezinova [1], Iveta Sarova [1], Zuzana Zemanova [2], Karla Svobodova [2], Halka Lhotska [2], Silvia Izakova [2], Lenka Pavlistova [2], Anna Jonasova [3], Jaroslav Cermak [4], Kyra Michalova [2]

[1]Institute of Hematology And Blood Transfusion, Cytogenetic Department, Prague, Czech Republic; [2]Center of Oncocytogenetics, Institute of Medical Biochemistry And Laboratory Diagnostics, General University Hospital And First Faculty of Medicine, Cytogenetic Department, Prague, Czech Republic; [3]1st Medical Department, General University Hospital And First Faculty of Medicine, Charles University, Clinical Department, Prague, Czech Republic; [4]Institute of Hematology And Blood Transfusion, Clinical Department, Prague, Czech Republic

**Correspondence:** Sarka Ransdorfova

*Molecular Cytogenetics* 2017, **10(Suppl 1):**2.P13

Complete or partial loss of chromosome 7 is a recurrent chromosomal aberration frequently found in myeloid disorders such as myelodysplastic syndromes (MDS) and acute myeloid leukemia (AML). This finding is an important prognostic indicator associated with poor response to the treatment and faster disease progression.

During the years 2006–2016, we examined 1463 adults with AML (n = 615) and MDS (n = 848). In bone marrow cells of 151 patients monosomy 7, deletion of 7q/7p or other structural rearrangements of chromosome 7 were found. Karyotypes were analyzed using conventional and molecular cytogenetic techniques: FISH (Abbott, Kreatech), mFISH/mBAND (MetaSystems) and array CGH/SNP (Illumina, Agilent). In 143 patients (95%) chromosome 7 aberration was proved in combination with other changes or as a part of complex karyotype, in 8 patients (5%) monosomy 7 was a sole aberration. The most frequently altered regions were identified in bands 7q22, 7q31 and 7q33-7q35. Ring chromosome 7 was found in 9 cases (in one patient a sole aberration). Dicentric chromosome 7 was demonstrated in 27 (19%) patients. Translocations of chromosome 7 often involved partner chromosomes 17 (n = 8; 5,5%), 2 (n = 6; 4%) and 11 (n = 6; 4%). In two cases clonal evolution was found: in the first patient three separate clones were detected with monosomy 7, r(7)(p11.2q21) and del(7)(q21q36). In the second patient, only del(7)(q11q31) was found initially, in the next sample after 5 months two clones with del(7)(q11q31) and with monosomy 7 were proved, and in the last sampling after 2 months only monosomy 7 was observed. These findings correspond to the theory of multi-step process, when deletion 7q occurs in the early stage of the disease as the first step of the clonal evolution, which later on leads to the complete monosomy 7.

Supported by MHCR project 00023736, RVO-VFN64165, GACR P302/12/G157, ProgresQ28.

### 2.P14

### Fluorescence in situ Hybridization as a Tool in the Diagnosis of Soft Tissue Sarcomas

Jana Zmolikova [1], Irena Urbanovska [2], Magdalena Uvirova [1], Sylva Pitronova [1], Romana Skalikova [1], Jarmila Simova [3], David Konvalinka [3], Dusan Ziak [4], Jana Dvorackova [5]

[1]Cgb Laboratory Inc., Laboratory of Cytogenetics, Ostrava-Czech Republic; [2]University of Ostrava, Faculty of Medicine, Ostrava-Czech Republic; [3]Cgb Laboratory Inc., Laboratory of Molecular Genetics, Ostrava-Czech Republic; [4]Cgb Laboratory Inc., Laboratory of Pathology, Ostrava-Czech Republic; [5]Ostrava University Hospital, Institute of Pathology, Ostrava-Czech Republic

**Correspondence:** Jana Zmolikova

*Molecular Cytogenetics* 2017, **10(Suppl 1):**2.P14

Soft tissue sarcomas (STS) are rare solid cancers of mesenchymal cell origin accounting for <1% of adult cancers and they represent histologically and molecularly heterogeneous group of tumors. The diagnostics of STS is difficult because of heterogeneity and low incidence, but it is necessary to diagnose the highly malignant sarcomas early and accurately. Specific genetic findings as translocations of SS18, COL1A/PDGFB, EWSR1, DDIT3 genes have been found in 30% STS, and they can be detected by fluorescence in situ hybridization (FISH).

Between January 2014 and January 2017, in our laboratory, 17 formalin-fixed, paraffin-embedded (FFPE) tissues were examined by FISH using probes: Kreatech ON SYT (18q11) Break, ON EWSR1 (22q12) Break, ON CHOP (12q13) Break, ZytoVysion SPEC COL1A1/PDGFB Dual color.

A total of 17 cases were evaluated, namely a Ewing sarcoma (7 cases), Synovial sarcoma (8 cases), Liposarcoma (1) and Dermatofibrosarcoma (1). Rearrangements of investigated genes were identified in 11 samples (64,7%): rearrangement of SS18 in 3 cases of synovial sarcoma, rearrangement of EWSR1 in 4 cases of Ewing sarcoma, rearrangement of genes COL1A1/PDGFB in one case of dermatofibrosarcoma and rearrangement of gene DDIT3 in one case of liposarcoma. In 2 cases of suspect Ewing sarcoma with rearrangement of EWSR1 the final histopathological diagnosis were olfactory neuroblastoma and extraskeletal myxoid chondrosarcoma; this genetic change in both diagnosis has been described in literature before. 6 FISH negative cases (35,3%) were reported as stromal endometrial carcinoma, malignant peripheral nerve sheath tumor, leiomyosarcoma, adenocarcinoma, sarcomatoid carcinoma.

Rearrangements of investigated genes have been identified by FISH in 64,7% cases, the findings were corresponding with the final diagnosis and with literature.

### 2.P15

### Is the right time for aCGH analysis to replace FISH for CLL investigation

Valentina Achille [1], Anna Venci [1], Luciana Di Schiena [1], Carmelo Carlo-Stella [2], Daniela Bettio [1]

[1]Humanitas Clinical And Research Center, Cytogenetics And Medical Genetics Laboratory, Rozzano, Milan, Italy; [2]Humanitas Clinical And Research Center, Humanitas Cancer Center And Humanitas University, Rozzano, Milan, Italy

**Correspondence:** Daniela Bettio

*Molecular Cytogenetics* 2017, **10(Suppl 1):**2.P15

Chronic lymphocytic leukemia (CLL) exhibits a highly heterogeneous clinical course, where genomic aberrations with known clinical relevance are mainly gains and losses, rather than balanced rearrangements. This observation justifies the application of a-CGH technology in the clinical practice, in particular using a-CGH + SNPs (SurePrint G3 Cancer CGH + SNPs 4x180K platform, Agilent) that allows the identification of loss of heterozygosity (LOH) and uniparental disomy (UPD), events that play a critical role in haematological diseases.

So far we analyzed 50 selected patients: 26 using classical cytogenetic, FISH and a-CGH + SNPs and 24 with classical cytogenetics along with a-CGH + SNPs.

The most recurrent anomalies identified by the array platform, with proved or possible prognostic significance, included losses in 2p11.2(4%), 3p26.3-p25.3(4%), 6q14.2-q22.3(8%), 9p21.3(8%), 10q22.2-q26.3(12%), 11q22.1-q23.3(16%), 14q24.2-q32.33(6%), 15q14-q15.1(6%), 17p13.3-p12(12%), gains in 2p25.3p14(4%), and 8q12.3-q24.3(4%) and trisomy 12(16%). Moreover, two distinct subtypes of 13q14 deletions were identified: type I exclusive of RB1(21%) and type II inclusive of RB1(18%) characterized by a worse clinical outcome.

LOH was observed at: 1q21.2q44(2%), 5q23.3(8%), 6q13q14.1(2%), 7p21.1p15.3(2%), 8q11.21q11.23(2%), 9q33.1q33.3(2%) and 12q13.3q14.1(4%). UPD was detected for the whole chromosome 13(2%), 17q(2%), and 20q(2%).

In one case UPD was present in 18q in association with a duplication of qter region. In conclusion, combined a-CGH + SNPs analysis improved the characterization of genomic aberrations, allowing also the detection of clonal heterogeneity and low-grade mosaicism up to 10%. The flow chart we applied since 2015 that includes conventional cytogenetics and a-CGH + SNPs replacing FISH, turned to be a valid approach both in term of diagnostic/prognostic efficacy and saving time procedure.

### 2.P16

### Characterisation of chromosomal aberrations in mouse pancreatic cancer using multiplex FISH (M FISH)

Ruby Banerjee [1], Beiyuan Fu [1], Sebastian Muller [2], Roland Rad [2], Fengtang Yang [1]
[1]The Wellcome Trust Sanger Institute, Molecular Cytogenetics, Cambridge, UK; [2]Klinikum Rechts Der Isar Der Tu Munchen, Klinik Und Poliklinik Fur Innere Medizin Ii, Munich, Germany
**Correspondence:** Ruby Banerjee
*Molecular Cytogenetics* 2017, **10(Suppl 1):**2.P16

Cancer can be caused by mutations that activate oncogenes and/or inactivate tumour suppressor genes. In addition to such mutations, chromosome aberrations are a hallmark of cancer cells. Karyotyping by multiplex-FISH (M-FISH), a single cell assay, not only allows a genome-wide screen for gross chromosome rearrangements, but can also detect aberrations that are present in a subset of cells, thus making it an important tool in cancer research. In this study, we karyotyped 39 mouse primary pancreatic tumours using M-FISH. These tumours were induced by known mutating oncogenes and/or tumour suppressor genes in order to recapitulate the development of human pancreatic cancer, and were divided into three cohorts according to the gene(s) targeted.

From each tumour, 10 random metaphases were karyotyped using M-FISH and DAPI-banding. Apart from delineating the structural and numerical rearrangements in each tumour, M-FISH karyotyping revealed a variable level of karyotype heterogeneity and instability. More interestingly, we observed apparently characteristic rearrangements for each cohort, involving the chromosomes carrying the targeted mutations.

Kras and Cdkn2a cohort: 18 tumours were induced by mutations in the Kras oncogene (chromosome 6) and the Cdkn2a tumour suppressor gene (chromosome 4). Eleven tumours showed gains or rearrangement of chromosome 6, eleven showed deletion, loss or rearrangement involving chromosome 4, and seven showed aberrations involving both chromosomes.

Nfkb2 cohort: 17 tumours were induced by PIK kinase mutation (Nfkb2, an oncogene on chromosome 19), and 16 of them showed gains of chromosome 19.

In the third cohort, induced by Kras mutation and by knocking out tumour suppressor genes, three epithelial and mesenchymal tumours from the same mouse carried a common translocation involving chromosomes 9 and 11, indicating a common origin. This study is ongoing.

In conclusion, M-FISH karyotyping is a robust tool for the characterisation of genomic rearrangements in cancers. The karyotype heterogeneity data will enable a better interpretation of genomic data generated by comparative genomic hybridisation and next-generation sequencing, which generally use pools of cells.

### 2.P17

### FISH panel and prognosıs with AML's trisomy 8

Esra Çolak, H. Betül Gerik-Çelebi, F. Sirri Çam
Manisa Celal Bayar University Faculty of Medicine, Medical Genetics, Manisa, Turkey
**Correspondence:** Esra Çolak
*Molecular Cytogenetics* 2017, **10(Suppl 1):**2.P17

**Introduction**

Classical conventional cytogenetics and FISH (Fluorescent In Situ Hybridization) analysis provide great support for clinical diagnosis in the identification of leukemia diagnosis, prognosis and treatment options. In acute myeloid leukemia (AML), chromosomal arrangements that are 50-70% non-clonal are observed and more than 50 structural chromosome anomalies are described. Cytogenetic translocations determined according to the WHO criteria in de novo AML; t(8; 21)(q22; q22), t(15;17)(q22; q11-12), inv16 (p13q22), 11q23. It was aimed to diagnose the case with AML indications, to determine the prognosis and to assist with the treatment.

**Methods**

In interphase fish scanning; t(15;17)(q22; q11-12), inv16(p13q22), 11q23, TP53 (17P), del (5q), −7 del (7q) and del (20q) target regions were analyzed. Chromosome screening and examination were also performed by classical cytogenetic method.

**Results**

Approximately 35% of the cells examined were determined the deletion in p53 gene region and, detachment has been detected, suggesting translocation in the MLL (11q23) probe. In addition, two fusion signals were detected in 80% of patients suggesting translocation. Again, in the same cells, it was seen that there was one more signal from both chromosomes 8 and 21. In bone marrow culture studies, a small number of metaphases were obtained with the reason of the difficulty of the metaphase and found to be 46, XY in 3 areas and 46, XY, −1, der (1) +8 in 3 areas.

**Discussion**

MLL, rearrangement is observed in 85% of newly born B-ALL and is a poor prognosis for ALL. De novo AML is present in 3% of the cases and 10% in the secondary AML, reducing treatment success. t (8; 21) is observed in 10% of all AMLs mainly M2 AML. Recurrence after treatment is frequent. Trisomy 8 is a common finding, especially in myeloid leukemia. There are publications on the impact of imprinting on the clinic. t(9; 11), t (11; 19) and t (6; 11) of other chromosomal abnormalities may be associated with trisomy 8 at 5%. Trisomy 8 is rated as moderate and poor prognosis. No prognostic evidence has been reported with trisomy 21. By conventional chromosome analysis, trisomy 8 was detected and correlated. As a result, the classical cytogenetic method is extremely important since the FISH scan will probe known regions only.

### 2.P18

### Gene nuclear positioning in leukaemia with chromosome 7 rearrangements

Denise Ragusa [1], Concetta Federico [2], Claudia Giovanna Leotta [2], Francesca Bruno [2], Anna Maria Longo [2], Dominika Dercz [1], Salvatore Saccone [2], Sabrina Tosi [1]
[1]Brunel University London, Division of Biosciences, College of Health And Life Sciences, Institute of Environment, Health And Societies, Uxbridge, UK; [2]University of Catania, Department of Biological, Geological And Environmental Sciences, Catania, Italy
**Correspondence:** Denise Ragusa
*Molecular Cytogenetics* 2017, **10(Suppl 1):**2.P18

The arrangement of the genome within the cell nucleus is organised to consent optimal gene expression. Overall, transcriptionally inactive regions are mostly found towards the nuclear periphery, and active regions in the nuclear interior. We previously showed that the radial distribution of human chromosome 7 in the nucleus of lymphocytes depends on the chromosomal band gene density, with gene rich regions protruding towards the nuclear interior and gene poor regions remaining at the periphery. Our hypothesis is that chromosomal rearrangements such as translocations cause a disturbance in the distribution of chromatin in the nucleus, bringing regions of the genome closer together or further apart, depending on the chromosomal bands affected by the rearrangements. Disturbance to genome organisation, and thus gene expression, is a relevant factor in the development of disease. In this study, we focused on the characterisation of chromosome 7 abnormalities and the radial positioning of specific genes, in particular HLXB9, mapping at 7q36, in acute myeloid leukaemia (AML) patient samples and in the AML derived cell line GDM-1. The latter harbours a t(6;7)(q23;q36) with breakpoint distal to HLXB9. We used dual-colour fluorescence in situ hybridisation (FISH) using probe combinations to discriminate the normal chromosome 7 from the der(7). In GDM-1, the HLXB9 on the der(7) mapped at the nuclear periphery, whereas the non-translocated allele was in the nuclear interior, considered the transcriptionally active compartment. Moreover, a combination of dual colour FISH and in situ reverse transcription showed that HLXB9 transcripts emerged from the allele repositioned towards the nuclear interior. This rather unusual finding indicates that regulation of gene expression in cancer cells carrying chromosomal translocations might be more complex than previously thought. Further studies will elucidate the impact of gene repositioning and HLXB9 expression in AML diagnosis and prognosis.

### 2.P19

### MYC contaınıng double mınute chromosomes orıgın structure and ımpact upon transcrıptome ın Acute Myeloıd Leukemıa

L'abbate Alberto [1], Tolomeo Doron [1], Turchiano Antonella [1], Pafundi Mariella [1], Daniele Giulia [1], Lonoce Angelo [1], D'addabbo Pietro [1], Carella Massimo [2], Palumbo Orazio [2], Merla Giuseppe [2], Augello Bartolomeo [2], Dolnik Anna [3], Muhlematter Dominique [4], Van Roy Nadine [5], Haferlach Claudia [6], Bullinger Lars [7], Ingrid Cifola [8], Marco Severgnini [8], Gianluca De Bellis [8], Storlazzi Clelia Tiziana [1]
[1]University of Bari, Department of Biology, Bari, Italy; [2]Irccs 'casa Sollievo Della Sofferenza', Laboratory of Oncology, San Giovanni Rotondo, Italy; [3]University Hospital of Ulm, 3department of Internal Medicine Iii, Ulm, Germany; [4]Chuv, Centre Hospitalier Universitaire Vaudois, Lausanne, Switzerland; [5]Ghent University, 5center For Medical Genetics, Ghent, Belgium; [6]Mll Munich Leukemia Laboratory, Citogenetics Laboratory, Munich, Germany; [7]University Hospital of Ulm, Department of Internal Medicine Iii, Ulm, Germany; [8]Institute For Biomedical Technologies (itb), Cnr, Segrate, Italy
**Correspondence:** Storlazzi Clelia Tiziana
*Molecular Cytogenetics* 2017, **10(Suppl 1):**2.P19

Double minute chromosomes (dmin) are rare in Acute Myeloid Leukemia (AML) and their genesis, as well as their impact on the tumor transcriptome, are still poorly understood. Recently, the emergence of dmin was explained by chromothripsis; however, our results did not support this possibility in tumor cell lines with MYC-dmin. In addition, MYC is not overexpressed when amplified in leukemia, suggesting a nonlinear correlation between amplification and overexpression. By integrating DNAseq, SNP array, RNAseq, FISH and PCR, we reconstructed the internal structure of head-to-tail amplicons in 23 leukemia cases with MYC-dmin. For the majority of them (19/23), we could identify an "ancestral" amplicon as the most prevalent amplified structure. Furthermore, amplicons displayed variable levels of molecular heterogeneity. Our analyses excluded chromothripsis as the driving force underlying amplicon genesis in our samples. Furthermore, we detected neocentromeres emerging at ring chromosomes that harboured tandemly amplified sequences. Interestingly, dmin were accompanied by a number of novel fusion transcripts involving 8q24 amplified genes (mainly PVT1), validated by RT-PCR and Sanger sequencing. Several of these chimeras were recurrent among samples and showed multiple transcript isoforms, although not being supported by genomic rearrangements. Interestingly, RNA-seq analysis of 20 AML cases with normal karyotype, obtained at The Cancer Genome Atlas, and of two remission samples of dmin cases, failed to identify any of the validated chimeras. Furthermore, we detected a significant over-expression of a PVT1 circular RNA (circPVT1), described as a molecular sponge for miRNA with tumor-suppressor activity, in cases with 8q24 amplifications versus cases with normal karyotype. Our results open new scenarios on the genesis and transcriptional impact of 8q24 amplifications in AML, suggesting that the role of post-transcriptional chimeras and circular RNAs accompanying amplifications needs further clarifications.

### 2.P20

### From the establishment of primary culture cell lines of oral squamous cell carcinoma to the identification of complex rearrangements

Isabel M Carreira [1], Ilda P Ribeiro [1], Alexandra Mascarenhas [2], Susana I Ferreira [2], Joana Rodrigues [2], Vanessa Marques [2], Claúdia Pais [2], Francisco Marques [3], Joana B Melo [1]
[1]Faculty of Medicine, University of Coimbra, Coimbra, Portugal, Cytogenetics And Genomics Laboratory; Cimago – Center of Investigation On Environment, Genetics And Oncobiology, Coimbra, Portugal; [2]Faculty of Medicine, University of Coimbra, Coimbra, Portugal, Cytogenetics And Genomics Laboratory, Coimbra, Portugal; [3]Faculty of Medicine, University of Coimbra, Coimbra, Portugal, Department of Dentistry, Coimbra, Portugal
**Correspondence:** Isabel M Carreira
*Molecular Cytogenetics* 2017, **10(Suppl 1):**2.P20

**Introduction**

Oral squamous cell carcinoma (OSCC) is frequently diagnosed in advanced stage, presenting a poor 5-year survival rate. Cell lines derived from tumors are extensively used as cancer models in research increasing the understanding of cancer biology and enabling the prediction of patient response to drugs. Nevertheless, only a small number of primary tumor cell lines have been successfully developed, since human carcinomas exhibit a wildly growth in the body, but in culture they are particularly difficult to establish and grow. In this study we aimed to establish and perform a detailed characterization of numerical and structural aberrations in OSCC primary cell cultures.

**Methods**

Primary cell cultures were established from three surgically resected OSCC samples (tongue and retromolar trigone tumors, stage II and IV). The (cyto)genomic characterization was assessed by karyotype and array comparative genomic hybridization (aCGH) using sex-matched controls.

**Results and Discussion**

These primary cell cultures are near-triploid. Chromosome analysis revealed complex karyotypes with several numerical and structural alterations, involving almost all chromosomes. Isochromoses are present in these cultures, including i(5)(q10), i(9)(q10), i(1)(q10) and i(8)(q10), which are common aberrations of the carcinogenesis process. Genomic imbalances were detected by aCGH, such as in chromosomes 3, 5, 8, 9, 18 and X, being the most of these aberrations in agreement with cytogenetic results. Some of these alterations were also observed in commercial OSCC cell lines previously characterized in our laboratory. The identification of complex rearrangements in primary cultures is useful to establish the OSCC genetic profile, contributing for the identification of diagnosis and prognosis biomarkers. Additionally, the concordance between primary cultures and commercial cell lines shows the utility of these cells in further studies, enriching the resources available for oral cancer research.

### 2.P21

### Acute myelomonocytic leukemia with complex karyotype

Blanka Godza [1], Dóra Isaszegi [1], Szabolcs Modok [2], Dóra Földeák [2], András Kozma [3], Emma Ádám [3], Zsuzsanna László [1], Emese Horváth [1], Zita Borbényi [2], Márta Széll [1]
[1]University of Szeged, Department of Medical Genetics, Szeged, Hungary; [2]University of Szeged, Second Department of Internal Medicine And Cardiology Center, Szeged, Hungary; [3]St. Istvan And St. Laszlo Hospital, Department of Hematology And Stem Cell Transplantation, Budapest, Hungary
**Correspondence:** Emese Horváth
*Molecular Cytogenetics* 2017, **10(Suppl 1):**2.P21

Cytogenetic findings are among the most important prognostic factors in acute myeloid leukemia. Deletion of the long arm of chromosome 5 is a common abnormality. The association of 5q loss with complex karyotype, occurring only in 13% of the patients older than 55 is less frequent. 5q deletions are most commonly accompanied by abnormalities affecting chromosmes 7, 20, 13, 12, 17, 18, and 3.
Our aim was the analysis of genotype-phenotype correlation in a case of AML with complex karyotype.
Chromosome analysis performed by G-banding and extended fluorescence in situ hybridization (FISH) tecniques were used to determine the cytogenetic abberrations.
Chromosome banding and extended FISH analysis revealed an 5q deletion and a complex karyotype harbouring ≥5 abberrations. However along the aforementioned chromosome 3 and 7, chromosomes 11, 22 and 6 were also affected and the following karyotype could be described:
44,XY,-3,del(5)(q13q33),del(6)(q13),der(7)del(7)(p10)-dic(7;11)(p10;q10),-11,i(22)(q10),der(22)t(3;22)(q26;p11)add(3)(?)
These complex karyotypes are classified as adverse genetic risk. The patient received 7 + 3 induction therapy with idarubicin. Control biopsy showed 20% of residual disease. Due to the poor prognosis and the residual disease, allogenic transplantation was an option and search for a donor for transplantation was initiated.
The patient reached complete remission after a treatment with FLAG-IDA. During this period consolidation therapy was given. An appropriate donor wasn't available therefore the treatment continued with 2 cycles of intermedier Ara-C. After 4 months a relapse occured and low-dose Ara-C was given again as palliative treatment. Despite the therepeutic efforts the patient deceased because of sepsis, kidney failure and respiratory distress.
In accordance with other studies, we found that complex karyotype is associated with unfavorable prognosis and rapid disease progression. This case further emphasizes the importance of identifying therapeutic targets for these subset of patientes. For this accurate cytogenetic and molecular characterization is indispensable.

### 2.P22

### Chromosomal aberrations in lymphocytes of patients with newly diagnosed solid tumors and patients undergoing long term chemotherapy detected by FISH

Petra Musilova [1], Hana Sebestova [1], Miluse Vozdova [1], Dita Kadlcikova [1], Marketa Petrovova [2], Marek Svoboda [2], Jiri Rubes [1]
[1]Central European Institute of Technology - Veterinary Research Institute, Department of Genetics And Reproduction, Brno, Czech Republic; [2]Masaryk Memorial Cancer Institute, Clinic of Comprehensive Cancer Care, Brno, Czech Republic
**Correspondence:** Petra Musilova
*Molecular Cytogenetics* 2017, **10(Suppl 1):**2.P22

Chromosomal aberrations are recognized as a valuable biomarker of genotoxic exposure and cancer susceptibility. Increased frequency of chromosomal aberrations indicates an increased risk of cancer. Phytohaemagglutinin-stimulated whole blood lymphocytes were cultured in complete RPMI1640 medium at 37 °C for 72 h. Colcemid was added two hours before the end of cultivation. The cells were harvested and dropped on microscopic slides according to a standard procedure. The slides were denatured in 0.07 M NaOH and hybridized with fluorescently labeled painting probes for chromosomes 1 (red), 2 (green) and 4 (green). 1000 metaphase cells from each sample were scored under a fluorescent microscope and aberrant cells were captured and classified according to the Protocol for Aberration Identification and Nomenclature. Genomic frequencies of stable chromosomal exchanges were calculated. The non-parametric Mann–Whitney U test was used for statistical analysis.
We investigated 53 healthy control individuals recruited through the outpatient clinic of preventive oncology who have not suffered from cancer and live and work in a non-hazardous environment (average age 48.2 ± 11.0; group A), 128 patients with newly diagnosed cancer (average age 59.7 ± 11.9; group B) and 10 patients with metastatic disease who have been receiving chemotherapy for at least 3 years (average age 58.9 ± 10.4; group C). Genomic frequencies of translocations per 1000 cells (FG) in groups A, B and C were 22.9 ± 16.2, 33.6 ± 21.5 and 347.5 ± 288.7, respectively. Differences in FG between groups A and B can be probably ascribed to higher age in group B. We detected highly significant correlation of FG with age (R = 0.36 in group A, R = 0.40 in group B). We detected significantly higher FG in group C and high variability among individuals.
Supported by Ministry of Health of the Czech Republic, grant no. 15-33968A

### 2.P23

### Cytogenetic characterization of 133 acute lymphoblastic leukemia cases

Yasmine El Ayeb [1], Hend Chaker [1], Wiem Ayed [1], Helmi Guermani [1], Nabila Abidli [1], Imen Chemkhi [1], Yosr Ben Abdennebi [2], Manel Bchir [2], Hend Ben Neji [2], Balkiss Meddeb [3], Samia Menif [4], Ahlem Amouri [1]
[1]Institut Pasteur Tunis, Cytogenetics, Tunis, Tunisia; [2]Aziza Othmana Hospital, Hematology, Tunis, Tunisia; [3]Aziza Othmana, Hematology, Tunis, Tunisia; [4]Institut Pasteur Tunis, Hematology, Tunis, Tunisia
**Correspondence:** Yasmine El Ayeb
*Molecular Cytogenetics* 2017, **10(Suppl 1):**2.P23

Acute lymphoblastic leukemia is a disseminated malignancy of B- or T lymphoblasts imposing a rapid diagnostic process to support an optimal therapy. The cytogenetic and Molecular hematology laboratories of Institute Pasteur of Tunis drain all Tunis city ALL cases. The purpose of this study was to determine the frequencies of chromosomal abnormalities among ALL Tunisian patients and to report the rare detected cytogenetic alterations. Our cohort included 133 consecutive patients, who presented with ALL during 2016, from January to December. Chromosomal abnormalities were evaluated by R-banding karyotypes and RT-PCR. RT-PCR determination was carried out for BCR-ABL1, KMLT2A-AFF1 (MLL-AF4) rearrangements. Among analyzed cases, 56 were de novo ALL. The current study includes infants leukemias (5.3%), chidren leukemias (44.3%) and adult leukemia (48.9%). Concerning de novo ALL, 89.3% were B-ALL and 10.71% were T-ALL .Conventional karyotype and RT-PCR results were highly concordant (96% of cases). B-ALL cases showed, t(9;22)(q34;q11) in 16%, with recurrently additional cytogenetic abnormalities, KMT2A rearrangement in 2% of cases, hyperdiploidy in 8% of cases and complex karyotypes in 14% of cases . For T-ALL , two rarely described translocations were noticed, t(11;14)(p15;q11)and t(11;14)(p13;q11). These rearrangements involve LMO1 (11p15), LMO2(11p13) and TCRD/A ( 14q11). Their prognostic significance is not well defined because of their low frequency. The follow up of one of these cases does reveal no cytogenetic remission after first induction therapy. One case of T-ALL have shown t(6;14)(q24;q23). This translocation has never been reported to our best knowledge. Finally, as far as we know, this is the first series of cytogenetic findings in patients with ALL reported in Tunisia.

### 2.P24

### Cytogenetic analysis in 101 Tunisian patients with de novo acute myeloid leukemia

Hend Chaker[1], Yasmine El Ayeb[1], Wiem Ayed[1], Helmi Guermani[1], Nabila Abidli[1], Imen Chemkhi[1], Yosr Ben Abdennebi[2], Manel Bchir[2], Hend Ben Néji [2], Balkis Meddeb [2], Samia Menif [3], Ahlem Amouri [1]
[1]Institut Pasteur, Cytogenetics, Tunis, Tunisia; [2]Aziza Othmana Hospital, Hematology, Tunis, Tunisia; [3]Institut Pasteur, Molecular Hematology, Tunis, Tunisia
**Correspondence:** Hend Chaker
*Molecular Cytogenetics* 2017, **10(Suppl 1):**2.P24

Cytogenetic alterations represent interesting biomarkers for diagnosis, prognosis assessment and treatment adjustment of acute leukemia. We studied the cytogenetic and the molecular findings in 101 consecutive Tunisian patients who presented with de novo acute myeloid leukemia (AML), during 2016, from January to December. Analysis was achieved in Cytogenetic and Molecular Hematology Laboratories of Institute Pasteur of Tunis, draining most Tunis city AML cases for diagnosis and follow up. The study included pediatric and adult leukemia. Chromosomal abnormalities and genes rearrangement were detected by R-banding karyotype and RT-PCR, carried out for PML-RARA, RUNX1-RUNX1T1 (AML1-ETO) and CBFb-MYH11 transcripts. 51% of patients had cytogenetic abnormalities in their karyotypes, 36% had normal karyotype and 13% of patients had no adequate metaphases for karyotype analysis. Based on karyotypes findings, analyzed AML were categorized into four cytogenetic risk groups. The first is favorable cytogenetic risk group (21% of cases), includes patients presenting t(15 ;17)(q24 ;q21), t(8 ;21)(q22 ;q22) and inv(16)(p13q22) found respectively, in 10%, 5% and 6% of cases. The second is intermediate cytogenetic risk group (49% of cases), includes patients presenting normal karyotype, trisomy 8, −Y. The third is unfavorable cytogenetic risk group (21% of cases), includes patients presenting del(5q)/-5, −7/del(7q), 11q23 translocations/trisomy 11, t(9;22)(q34;q11), del(9q) and complex karyotype. Remaining eight patients (9% of cases) had miscellaneous clonal aberrations considered to have unknown prognostic significance because of their low frequency in AML. We have compared the incidence of specific clonal abnormalities in our series, to the results of cooperative studies including higher number of patients. In this study, we aimed to determine the frequencies and subtypes of chromosomal abnormalities among AML patients in the Tunisian population, and to report the rare detected cytogenetic alterations in this series of patients.

### 2.P25

### Identification of a constitutional t(1;19)(q23;p13) translocation in an Extranodal Natural Killer T cell Lmyphoma patient

Sibel Berker Karauzum [1], Ramazan Erdem [2], Ozden Altiok Clark [3], Ayse Esra Manguoglu [1], Turgay Ulas [2], Bahar Akkaya [4], Levent Undar [2]
[1]Medical Faculty of Akdeniz University, Medical Biology And Genetics, Antalya, Turkey; [2]Medical Faculty of Akdeniz University, Hematology, Antalya, Turkey; [3]Medical Faculty of Akdeniz University, Medical Genetics, Antalya, Turkey; [4]Medical Faculty of Akdeniz University, Pathology, Antalya, Turkey
**Correspondence:** Sibel Berker Karauzum
*Molecular Cytogenetics* 2017, **10(Suppl 1):**2.P25

An E2A-PBX1 fusion gene produced from a t(1;19)(q23;p13) translocation is commonly observed in childhood pre-B acute lymphoblastic leukemia. The t(1;19) translocation is detectable in neonatal Guthrie cards in 10% of pediatric ALL cases. Although chromosomal abnormalities have been reported in limited extranodal natural killer (NK)/T-cell lymhoma cases, with the deletion of 6q22-25 being the most frequently seen, recurrent translocations, especially the constitutional t(1;19)(q23;p13) translocation, have not yet been described in NK/T-cell lymphoma in the literature. Here, we present the cytogenetic characterization of a 46-year-old Turkish female who was clinically diagnosed with extranodal NK/T-cell lymphoma. Using cytogenetic analysis and FISH on the patient's bone marrow, peripheral blood, and skin biopsy, it was found that the sole constitutional chromosome abnormality was the t(1;19) translocation. While the E2A- PBX1 fusion gene was detected in both of the bone marrow and the peripheral blood samples, it wasn't detected in the skin biopsy by using RT-PCR technique. In conclusion, we believe that having the constitutional t(1;19) translocation may predispose to the development of NK/T-cell lymhpoma with the presence of secondary events.
**Consent to publish:** The authors confirm that written informed consent was received by the patient for publication.

### 2.P26

### HLXB9 expression is independent from its nuclear gene positioning in breast cancer cells

Temitayo Owoka [1], Concetta Federico [2], Helen Foster [1], Anna Maria Longo [2], Salvatore Saccone [2], Joanna M Bridger [1], Amanda Harvey [1], Sabrina Tosi [1]
[1]Brunel University London, Department of Life Science, Uxbridge, UK; [2]University of Catania, Dipartimento Di Scienze Biologiche, Geologiche E Ambientali, Catania, Italy
**Correspondence:** Sabrina Tosi
*Molecular Cytogenetics* 2017, **10(Suppl 1):**2.P26

The location of genes and chromosomes in the interphase nucleus of healthy human cells is non-random, therefore understanding the mechanisms that regulate nuclear genome organization is an emerging field of interest. In disease states such as cancer, changes in the location of genes from their normal position in the cell nuclei have been observed and can be linked with abnormal gene expression. HLXB9 is a homeobox gene mapping at 7q36 that is expressed during foetal development and is important in the genesis of organs from the ectoderm and endodermal germ layer. Overexpression of HLXB9 has been reported in various cancers including hepatocarcinoma, insulinomas and colorectal cancer. Our previous work showed that overexpression of HLXB9 corresponds to a mis-location of this gene to the inner part of the nucleus in a specific subset of childhood leukaemia.
In our current study, we performed Real Time Quantitative PCR, indirect immunofluorescence and fluorescence in situ hybridisation in three breast cancer derived cell lines and as controls we used the MCF10a cell line and a number of human mammary epithelial cells (HMECs) samples. All these samples were investigated for both HXLB9 expression levels and nuclear localisation. In non-cancer cells, HLXB9 resides typically at the nuclear periphery. Our studies show that HLXB9 is downregulated in BC cell lines compared to the controls although the HLXB9 gene is localised in the inner part of the nucleus in the cancer cells. These findings suggest that HLXB9 expression in breast cancer might be regulated by other factors and is not dependent on nuclear positioning. Further studies aimed to investigate the structure of the nuclear envelop in breast cancer cells might shed some light into the mechanisms of nuclear gene positioning and expression in these diseased cells.

### 2.P27

### Neuroblastoma by Fluorescence in situ Hybridization Experience of a Single Center in Singapore

Sharon Ai Chien Ling [1], Derrick Wen Quan Lian [1], Kenneth Tou En Chang [1], Joyce Ching Mei Lam [2], Bhattacharyya Rajat [2], Min Hwee Yong [1]
[1]Kk Women's And Children's Hospital, Department of Pathology & Laboratory Medicine, Singapore, Singapore; [2]Kk Women's And Children's Hospital, Haem/onc Services, Singapore, Singapore
**Correspondence:** Sharon Ai Chien Ling
*Molecular Cytogenetics* 2017, **10(Suppl 1):**2.P27

**Introduction**
Neuroblastomas are one of the most common solid tumours in children. Molecular cytogenetic studies are of paramount importance in the prognosis and treatment stratification of patients with this tumour, and are recommended by The International Neuroblastoma Risk Group Staging System (INRGSS). In particular, tumour cell ploidy, MYCN amplification, deletion of chromosome 1p and 11q, and gain of chromosome 17q portend an unfavorable prognosis. In this study, we report 16-years' of experience at the Cytogenetics Laboratory, KK Women's and Children's Hospital (KKH), Singapore.
**Methods**
Interphase fluorescence in situ hybridization (FISH) studies of neuroblastoma specimens from 2001 to 2016 were reviewed. These studies were performed on tumour imprints, formalin-fixed paraffin-embedded tissues (FFPE), fresh frozen tissues and bone marrow specimens, using locus-specific (MYCN, 1p and 11q or 17q) commercial fish probes.
**Results**
Our work summarises the results of 404 neuroblastoma samples, of which 32 were primary tumours and the other 372 were post-treatment tumours. A total of 166 neuroblastoma tumours (18 primary and 148 post-treatment tumours) exhibited one or more of the three genetics parameters being investigated. Less than 20% of these 166 tumours showed two or more of the three genetics parameters investigated. Of the 18 primary tumours, 14 correlated with unfavorable histology.
**Conclusion**
Our data suggests that MYCN amplification, 1p and/or 11q deletion are reliable independent parameters correlating with an unfavourable histology and poor prognosis of patients for primary tumour. Furthermore, our data also suggests that detection of these genetic parameters via FISH is also useful in follow up of post-treatment neuroblastomas.

### 2.P28

### 1p36 Abnormalities In Hematological Malignancies

R. Dilhan Kuru [1], Ayşe Çirakoğlu [1], Şükriye Yilmaz [1], Yelda Tarkan-Argüden [1], Şeniz Öngören [2], M.Cem Ar [2], Ayhan Deviren [1], Zafer Başlar [2], Teoman Soysal [2], Seniha Hacihanefioğlu [1]
[1]Cerrahpaşa Faculty of Medicine, Istanbul University, Medical Biology, Istanbul, Turkey; [2]Cerrahpaşa Faculty of Medicine, Istanbul University, Internal Medicine, Division of Hematology, Istanbul, Turkey
**Correspondence:** R. Dilhan Kuru
*Molecular Cytogenetics* 2017, **10(Suppl 1):**2.P28

Structural aberrations of chromosome 1p36 involving in tranlocations, inversions, deletions and duplications are very common in most hematologic malignancies. They are observed in myeloproliferative disorders (MPD), myelodysplastic syndrome (MDS) and multiple myeloma (MM). Also, 1q36 abnormalities are rare in other myeloid malignancies, acute lymphoblastic leukemia (ALL), chronic lymphocytic leukemia (CLL), and non-Hodgkin's lymphoma.
We present deletions and rearrangements of breakpoint 1p36 in 18 patients with hematologic malignancies in this study.
Rearrangements occur via translocations with diffrent chromosomes, additions and inversions. Our cases consist of 3 MDS, 2 AML, 2MM, 6 CML, 2 Lymphoma, 1 B-ALL, 1 ITP and 1 Aplastic anemia patients. We have shown t(1;3)(p36;q21) at 3 patients (1AML M2 and 2 CML). The t(1;3)(p36;q21) leads to fusion of PRDM16 (containing 16 gene) at 1p36 with RPN1 at 3q21. AML and MDS with t(1;3)(p36;q21) were first described by Moir et al. (1984). Translocations of PRDM16 lead to its overexpression, and those patients have a poor prognosis. Also 7 patients (1MDS, 2 MM, 2 CML, 1 AML and 1 Aplastic anemia) have translocation 1q36 with other chromosomes or rearrangements. One ITP patient has der(1)t(1;1)(p36;q21) and another CML patient has der(1)t(1;1)(p36;q12). 1p36 region consists of genes which are important in oncogenesis. der(1)t(1;1)(p36;q11-q32) are reported in literature for both myeloid and lymphoid neoplasias.
23 candidate tumor suppressor genes have been mapped in the 1p36 region, and it is the smallest region of deletion (SRD). Terminal 1p36 deletions are frequent in both myeloid and lymphoid neoplasias. We have also demostrated several terminal deletions from 1p11 to 1p36 at five patients, that are 2 MDS, 2 Lymphoma, and 1 B-ALL.

### 2.P29

### The Use of Multitarget FISH on Formalin Fixed Parafin Embedded Tissue in Bladder Cancer

Ayşe Çirakoğlu [1], R. Dilhan Kuru [1], Sultan Rana Ramazanoğlu [2], Süleyman Ataus [3], Bülent Alici [3], Ayhan Deviren [1], Seniha Hacihanefioğlu [1]
[1]Cerrahpaşa Faculty of Medicine, Istanbul University, Dept. of Medical Biology, Istanbul-Turkey; [2]Kanuni Sultan Süleyman Training And Research Hospital, Dept. of Pathology, Istanbul-Turkey; [3]Forte Urology, Urology, Istanbul-Turkey
**Correspondence:** Ayşe Çirakoğlu
*Molecular Cytogenetics* 2017, **10(Suppl 1):**2.P29

Bladder cancer is one of the common cancers in the world. The majority (70%) of bladder cancers (urothelial carcinoma) are superficial including papillary pTa (40%), pT1 (30%) and carcinoma in situ (Cis) (2-5%), at the time of diagnosis.
Genetic studies of bladder cancers observed that chromosomal changes are one of the important factors that act in tumorigenesis and behaviour of the tumor. Polysomies of chromosomes 3, 7, and 17 and deletions of p16 gene located at 9p21 are used as biomarkers in diagnosis of bladder tumors. Loss of both alleles of p16 has been shown that occur at early stages whereas polysomies of chromosomes 3, 7, and 17 are associated with progression of the tumor. A multicolor fluorescence in situ hybridization (FISH) assay, UroVysionTM, was developed to detect these abnormalities in urine specimens for diagnosis of bladder cancer. It can also be applied to parafin-embedded tissues.
In this study, we applied UroVysionTM on archival paraffin-embedded sections from 21 patients with superficial grade T1 tumors. All cases were FISH-positive. In 14 cases, polysomy of at least two chromosomes, in similar frequencies for all combinations, with biallelic deletion of p16 were observed. Six cases had only biallelic deletion of p16 and one case had polysomy of at least two chromosomes.

### 2.P30

### Analysis of NFκB1 gen ins del polymorphisms using PfLMI restriction enzyme on breast cancer

Onur Eroglu [1], Esin Guvenir Celik [2], Hacer Kaya [2], Merve Celen [2], Ceylan Demirci [2]
[1]Bilecik Seyh Edebali University, Biotechnology Application And Research Center, Bilecik, Turkey; [2]Bilecik Seyh Edebali University, Molecular Biology And Genetics, Bilecik,Turkey
**Correspondence:** Onur Eroglu
*Molecular Cytogenetics* 2017, **10(Suppl 1):**2.P30

Nuclear factor kapa B(NF-kB) is a transcription factor protein family and an important role in tumor development and aggressiveness by enhancing tumor angiogenesis, antiapoptosis and proliferation. The NFKB1 gene located at chromosome 4q24. The polymorphism is a 4-basepair insertion/deletion located 94 basepairs upstream of the gene (−94ins/del ATTG). Researchers indicated that the functional −94 ins/del ATTG polymorphism in the NFKB1 gene may be associated with cancer risk. We aimed to investigate the NFKB1 -94ins/del polymorphism using PfLMI restriction enzyme on breast cancer patients by Restriction Fragment Length Polymorphism (RFLP).
We analysed primary tumor core biopsies from 80 high-risk primary breast cancer patients (tumors ≥2 cm and/or lenfatic metastase and/or distant metastases and/or under 40 years) DNA was isolated after deparaffinization stage from the tissues of 80 patients, which belong breast cancer patients. After amplification the products digested at spesific sites by using PfLMI restriction enzyme. DNA fragmentation were analyzed using electrophoresed on 2% agorose gels.
We observed that 13.63% of patients are homozygous del/del (240, 45 bp) and 86.37% of patients are heterozygous ins/del (281, 240, 45 bp). Homozygous variants were cleaved by PfLMI restriction enzyme into two fragments of 240 and 45 bp. On the other hand, heterozygoutes showed all three bands, 281, 240 and 45 bp.
In this study we investigate the relationship between NFκB1-94 promotor site and risk of breast cancer. These results showed that NFκB1 promotor-94 ins/del ATTG gene polymorphism is related with breast cancer progression.

### 2.P31

### Concomitant KMT2A (MLL) GIGYF2 and TRA MYC gene fusions in an infant B cell precursor acute lymphoblastic leukemia (BCP ALL)

Sabrina Haslinger [1], Andrea Inthal [1], Karin Nebral [1], Margit Koenig [1], Astrid Mecklenbraeuker [2], Rolf Marschalek [3], Claus Meyer [3], Renate Panzer-Grümayer [2], Oskar A. Haas [1]
[1]Ccri, Children's Cancer Research Institute, Cytogenetics And Molecular Cytogenetics, Vienna, Austria; [2]Ccri, Children's Cancer Research Institute, Biology of Leukemia, Vienna, Austria; [3]Institute of Pharmaceutical Biology, Goethe University, Diagnostic Center of Acute Leukemia, Frankfurt/main, Germany
**Correspondence:** Sabrina Haslinger
*Molecular Cytogenetics* 2017, **10(Suppl 1):**2.P31

With 135 different and 94 already molecularly characterized rearrangements, the transcriptional co-activator KMT2A (MLL) is the most promiscuous gene that is involved in various types of acute leukemias. We present the case of a 20 month old boy with BCP-ALL and two translocations, a t(2;11)(q37;q23) and a t(8;14)(q24;q11) which relate to a novel KMT2A-GIGYF2 and a rare TRA-MYC gene fusion. FISH analyses with KMT2A, TRA and MYC specific dual color split-apart specific FISH probes, revealed the involvement of the former in the t(2;11) and the latter two in the t(8;14). Although copy number abnormalities (CNA) are rather uncommon in cases with KMT2A fusions, array analysis nevertheless uncovered a 1.1 Mb intron 17 deletion of GIGYF2 on chromosome 2 and another 38 kb exons 9–36 deletion of KMT2A on chromosome 11. The combination of these deletions was therefore not only highly suggestive of an underlying KMT2A-GIGYF2 gene fusion but their location also eased its direct confirmation with RT-PCR on the RNA and with LDI-PCR on the DNA level. Although the multitude of CNAs that accompany the physiological rearrangement of the TRA region prevented us to obtain a similar rewarding information in case of the t(8;14)(q24;q11), we found additional intragenic PAX5 and homozygous CDKN2A/B deletions as well as a copy-neutral loss of heterozygosity of 9p24.3q21.3. The Drosophila GIGYF2 (GRB10 interacting GYF) protein ortholog is a modulator of autophagy that controls neuron and muscle homeostasis. It is involved in siRNA-mediated post-transcriptional silencing by selectively blocking the process of translation of distinct capped mRNAs. TRA-MYC fusions are encountered in 0.5 to 1.3% of primarily BCP ALL in children and young adults with a male preponderance and in the context of Down syndrome and a t(9;22).

### 2.P32

### Cytogenetical aberration determines the therapeutic strategy in adult acute lymphoblastic leukemia

Arpad Szomor [1], Renata Csalodi [1], Agnes Nagy [1], Hussain Alizadeh [1], Zsuzsanna Szijarto [2], Bela Kajtar [3]
[1]University Pecs, First Department of Medicine, Pecs, Hungary;
[2]University Pecs, Department of Ophthalmology, Pecs, Hungary;
[3]University Pecs, Institute of Pathology, Pecs, Hungary
**Correspondence:** Arpad Szomor
*Molecular Cytogenetics* 2017, **10(Suppl 1):**2.P32

Several recurrent conventional cytogenetic abnormalities have been detected in acute lymphoblastic leukemia. The greatest impact on patient's management has been the finding that the cytogenetic result is an independent prognostic indicator. Certain karyotypes are associated with a very poor outcome.
The aim of our study was to determine the cytogenetic profiles of a series of 11 adulthood acute lymphoblastic leukemia. Patients treated at our clinic in the last 4 years were studied by standard cytogenetic analysis or fluorescent in situ hibridization. Mean age of three female and 8 male patients was 37 years (18–68), 8 B-cell and 3 T-cell cases were included of our series. We evaluated karyotype results of bone marrow specimens and/or peripheral blood. The frequency of cytogenetic abnormalities, including numerical and/or structural changes, was 72%. We observed 11q23 involvement: t(4;11)(q21;q23) in 2 cases, translocation of Philadelphia chromosome: t(9;22)(q34;q11) was identified also in 2 cases. Complex cytogenetic aberrations were detected in 4 cases. Following the report of the unfavorable cytogenetic results 6 of 11 patients were organized to recieve allogeneic hemopoietic stem cell transplantation. Therapeutic results without allogeneic stem cell transplantation are disappointing in these cases.

### 2.P33

### Conventional cytogenetics findings t(12;21)(p13;q22) translocation and 9p21deletion in pediatric ALL patients

Esra Manguoglu [1], Sibel Berker-Karaüzüm [1], Hakan Akkurt [1], Koray Yalçin [2], Alphan Küpesiz [2]
[1]Akdeniz University, Faculty of Medicine, Medical Biology And Genetics, Antalya, Turkey; [2]Akdeniz University, Faculty of Medicine, Pediatric Hematology, Antalya, Turkey
**Correspondence:** Esra Manguoglu
*Molecular Cytogenetics* 2017, **10(Suppl 1):**2.P33

Acute lymphoblastic leukemia (ALL) is one of the most common childhood malignancy. t(12;21)(p13;q22)/ ETV6-RUNX1 (TEL-AML1) and 9p abnormalities are among the major chromosome aberrations in pediatric and adult acute lymphoblastic leukemia. A total of 21 pediatric ALL patients were included in this study. Conventional cytogenetics analysis and FISH analysis for t(12;21)(p13;q22) translocation and 9p21 aberrations using dual color FISH probes were performed. Conventional cytogenetics analyses revealed hyperdiploidy in three patients and a complex karyotype in six patients. Ten patients had either heterozygous or homozygous deletion of 9p21(p16). On the other hand two of the five patients with the t(12,21)(p13;q22) translocation were also positive for 9p deletion and two patient had trisomy 21 in addition to t(12,21)(p13;q22) translocation. In addition, the deletion of 12p was detected in six patients while either duplication or amplification of 12p and 21q were observed in three patents without t(12;21) translocation. These and other cytogenetics results are evaluated with regard to clinical findings.

### 2.P34

### Rare translocatıon t(3;12)(q26;q21) and mecom rearrangement ın a patıent wıth myelodysplasıa

Gloria Cantamessa [1], Donatella Fantasia [2], Rita Di Gianfilippo [2], Matteo Cancelli [1], Artemio Di Tecco [2], Paolo Guanciali Franchi [1], Stefano Pulini [3], Prassede Salutari [3], Giuseppe Calabrese [1]
[1]Chieti University, Dept. of Medical, Oral And Biotech Sciences, C hieti-Italy; [2]Ausl Pescara, Oncohaematological Genetics, Pescara-Italy; [3]Ausl Pescara, Dept. of Hematology, Pescara-Italy
**Correspondence:** Gloria Cantamessa
*Molecular Cytogenetics* 2017, **10(Suppl 1):**2.P34

**Introduction**
Overexpression of EVI1, due to rearrangements of the locus MECOM in 3q26, has been described in 10% of acute myeloid leukemia (AML) and of myelodysplastic syndromes (MDS) as a marker of poor prognosis.
**Case report**
In March 2016 a 62-year-old female patient presented with hepatosplenomegaly, anemia, reduced platelet count and leukocytosis (Hb 9.5 g/dl; PLT 85000/mm3; WBC 16690/mm3; PMN 50%, Lymph 18%, Mono 22%, Blasts 10%). The patient was diagnosed as a severe MDS-EB2 (WHO 2016). Flow cytometry of bone marrow biopsy showed 15% of CD13+, CD33+, CD34+, CD117+ myeloid blasts. The patient received 5-Azacitidine chemotherapy and is currently in remission.
**Methods**
Cytogenetic analysis of bone marrow aspirate showed a homogeneous karyotype 46,XX,t(3;12)(q26;q21) [20]. The constitutional karyotype was normal 46,XX [17]. FISH analysis with three-color EVI1 probe (Metasystems, Germany) showed the translocation of 5′MECOM on chromosome 12q. Using a panel of BAC clones (Technogenetics, Italy) by FISH analysis, we mapped the breakpoint site within a region 2.6 Mb in size at 12q21.31.
**Discussion**
Despite yet uncharacterized, chromosome 12q21 is critical for chronic idiopathic myelofibrosis (CIMF), and the t(3;12)(q26;q21) has been described only in two patients, with AML secondary to treatment (Poppe et al., 2006), and CIMF JAK2-negative (Mešanović et al., 2014), respectively. In both cases EVI1 partner genes remained unknown. Our ongoing analysis has narrowed the breakpoint region in an interval of 2.6 Mb at 12q21.31, in view to enlighten 12q21 role in the pathogenesis of MDS/CIMF.
**Consent to publish:** The authors confirm that written informed consent was received by the patient for publication.

### 2.P35

### Allele specific mutation analysis revealed by chromosome specific sequencing; implications for chromosome instability during cancer genome evolution

Fumio Kasai [1], Jorge Pereira [2], Noriko Hirayama [1], Arihiro Kohara [1], Malcolm Ferguson-Smith [2]
[1]National Institutes of Biomedical Innovation, Jcrb Cell Bank, Ibaraki, Japan; [2]University of Cambridge, Department of Veterinary Medicine, Cambridge, UK
**Correspondence:** Fumio Kasai
*Molecular Cytogenetics* 2017, **10(Suppl 1):**2.P35

Tumor cells often show abnormal karyotypes, indicating the occurrence of chromosome rearrangements and the accumulation during clonal evolution. This causes difficulties in the analysis of mutation profiles when multiple variants are detected. Chromosome sorting by flow cytometry produces flow karyotypes that enable the isolation of abnormal chromosomes and the generation of chromosome-specific DNA. In this study, approximately 2000 chromosomes of a derivative chromosome t(9;14) and its homologous normal chromosomes 9 and 14 from an endometrial cancer cell line were used for the preparation of genomic DNA fragment libraries. Chromosome-specific sequencing identified the breakpoint junction in the der(9) at 9p24.3 and 14q13.1 and revealed the formation of a fusion gene. Different from single cell analysis, comparisons of mutation profiles between normal and abnormal alleles permits their precise mapping. This reveals additional variants that have occurred not in the abnormal allele but in an apparently normal allele, implying that chromosomes can be stable through fusions. Expression patterns have been investigated based on comparisons between the allele-specific variant profiles and the RNA sequencing. We demonstrate that allele-specific mutation profiles can be determined precisely by chromosome sequencing and show how chromosome rearrangements can influence chromosome instability.

### 2.P36

### Rare recurrent chromosomal abnormalities in CLL detected by conventional cytogenetics stimulated by CpG ODN & IL 2 and FISH

Martina Stoklasova [1], Bronislava Drevojankova [1], Alena Burdova [1], Tamara Sablaturova [1], Aneta Strnkova [1], Lucie Truhlikova [1], Zuzana Lencesova [1], Hana Kucharova [1], Yvona Brychtova [2], Anna Panovska [2], Karla Plevova [2], Alexandra Oltova [2], Jiri Mayer [3], Michael Doubek [3], Sarka Pospisilova [3], Natalija Rytikova [4], Dagmar Adamova [5], Irena Zygulova [5], Vladimira Heinzova [5], Jana Zuchnicka [6], Juraj Duras [6], Cecilia Bodzasova [6], Jana Laska [7], Marie Urbankova [8], Zuzana Jehlikova [8], Premysl Slezak [8], Jolanta Macurova [9], Daniel Janek [9], Elen Bogoczova [10], Drahomira Klodova [11], Zuzana Zetkova [11], Marek Wrobel [11], Martin Brejcha [11]
[1]Agel Research And Training Institute – Novy Jicin Branch, Department of Medical Genetics – Laboratory of Cytogenetics, Laboratories Agel, Novy Jicin, Czech Republic; [2]University Hospital And Faculty of Medicine, Masaryk University, Department of Internal Medicine – Hematology And Oncology, Brno, Czech Republic; [3]Central European Institute of Technology, Masaryk University, Department of Internal Medicine – Hematology And Oncology, University Hospital And Faculty of Medicine, Masaryk University, Brno, Czech Republic; [4]Hospital Krnov, Department of Hematology, Krnov, Czech Republic; [5]Silesian Hospital In Opava, Department of Hematology, Opava, Czech Republic; [6]University Hospital Ostrava, Institute of Clinical Hematology, Ostrava, Czech Republic; [7]Hospital Cesky Tesin, Department of Hematology, Cesky Tesin, Czech Republic; [8]Hospital Sumperk, Department of Hematology, Sumperk, Czech Republic; [9] Hospital Karvina-raj, Department of Hematology, Karvina, Czech Republic; [10] Hospital Vitkovice, Department of Hematology, Ostrava, Czech Republic; [11]Hospital Novy Jicin, Department of Hematology, Novy Jicin, Czech Republic
**Correspondence:** Martina Stoklasova
*Molecular Cytogenetics* 2017, **10(Suppl 1):**2.P36

Traditional chromosomal aberrations /i.e. del(11q), +12, del(13q), del(17p)/ occupy an important position in chronic lymphocytic leukaemia (CLL) as significant indenpendent prognostic factors.
FISH and conventional chromosome banding (CBA) stimulated by CpG oligonucleotides (CpG-ODN) and interleukin-2 (IL-2) are standard methods for capturing chromosomal abnormalities. We focused on the incidence of rare recurrent chromosomal aberrations (RCAs) that can provide new prognostic information in CLL.
Metaphase cytogenetic techniques and molecular-cytogenetic analyses were performed in 849 CLL-patients from 2008 to 2016 (319 females, 530 males, median age at the time of analysis: 66 years). CBA was performed on peripheral blood or bone marrow samples cultivated in medium with stimulants. I-FISH was performed on unstimulated cells for detection of del(11q), +12, del(13q), del(17p). RCAs were confirmed by I-FISH, multicolor FISH or multicolor banding.
A total of 778/849 (91,6%) cases were succesfully stimulated for metaphase analysis. Chromosomal aberrations were detected in 714/849 (84,1%) of patients by combining both methods.
RCAs were found in 214 cases (214/778, 27,5%). Most frequent RCAs were 3,7% gain of 2p, 3,7% del(6q), 3,5% rearrangement of IGH (translocations partners: 2, 6, 8, 9, 11, 12, 13, 15, 16, 18, 19), 2,6% del(14q), 1,7% gain of 8q (MYC gene), 1,2% +18, 1,2% +19 and other less known RCAs were also identified.
Cytogenetic results are highly relevant in defining the prognosis of CLL patients. I-FISH as gold standard for detection of important chromosomal aberrations and CBA after stimulation with cocktail of

CpG-ODN and IL-2 are efficient tools in routine analysis of chromosomal aberrations. The importance of RCAs in CLL will be discussed in the poster.

### 2.P37

### Genomic abnormalities in acute myeloid leukemia with normal karyotype

Aurora Arghir [1], Sorina Mihaela Papuc [1], Andreea-Cristina Tutulan-Cunita [1], Raluca Colesniuc [1], Ioana Borcan [1], Silvana Angelescu [2], Mihaela Andreescu [3], Diana Cisleanu [4], Ana-Maria Vladareanu [4], Horia Bumbea [4]
[1]Victor Babes National Institute of Pathology, Medical Genetics Laboratory, Bucharest, Romania; [2]Coltea Clinical Hospital, Hematology Department, Bucharest, Romania; [3]Colentina Clinical Hospital, Hematology Department, Bucharest, Romania; [4]Emergency University Clinical Hospital, Hematology Department, Bucharest, Romania
**Correspondence:** Aurora Arghir
*Molecular Cytogenetics* 2017, **10(Suppl 1):**2.P37

Acute myeloid leukemia (AML) is a heterogeneous group of disorders characterized by excessive proliferation of myeloid progenitor cells. Although cytogenetic lesions are described in ~ 50% of de novo AMLs, the rest of the patients need further testing in order to obtain genetic data that informs the diagnosis, prognosis and response to treatment.
We report on the results of microarray investigation of genomic variants in 12 patients with AML and normal cytogenetics (AML-NC). Genomic profiling was performed on 4x180K CGH + SNP platforms (Agilent Technologies) using genomic DNA extracted from bone marrow samples obtained at diagnosis, prior to any therapy. DNA extracted from buccal swab samples was hybridized on microarrays for 4 patients. Targeted NGS testing with Ion AmpliSeq™ AML Research Panel is ongoing for the patients tested by microarray.
Several copy number anomalies (CNA) and copy-neutral loss of heterozygosity (CN-LOH) were observed in our patient group. Three patients presented CN-LOH larger than 10 Mb affecting chromosomes 6 and 13: a patient with a 47 Mb CN-LOH on 6p25.3-p12.3, a patient with a 50 Mb CN-LOH on 13q14.2-q32.1 and a patient with co-occurring CN-LOH on 6p25.3-p21.2 (37 Mb) and 13q12.11-13q34 (94 Mb). Both 6p and 13q regions are recurrently affected by CN-LOH in AML and considered to contribute to leukemogenesis by doubling an oncogenic event. Among CNAs, we report a 12p deletion including CDKN1B gene previously reported as haploinsufficient in AML.
Our study proves the value of genomic investigation of hidden anomalies, highlighting the complexity of the molecular profiles in AML-NC. The ultimate goal of these genomic investigations is a better understanding of cancer biology and improvement of patient care.

**Acknowledgments**
This poster was realized within Core Programme, supported by ANCSI, Project no 16.22.01.01.

### 2.P38

### A retrospective evaluation of 512 results of UroVysion test applications

Tadeusz Kałużewski [1], Lucyna Morzuch [1], Adam Jędrzejczyk [2], Piotr Marks [2], Marek Różniecki [3], Bogdan Kałużewski [1]
[1]Genos, Laboratory of Medical Genetics, Zapolice, Poland; [2]The Jp Ii Voividship Hospital In Belchatow, Department of Urology, Belchatow, Poland; [3]Voividship Hospital In Zduńska Wola, Non-public Department of Urology Rozniecki And Partners, Zduńska Wola, Poland
**Correspondence:** Bogdan Kałużewski
*Molecular Cytogenetics* 2017, **10(Suppl 1):**2.P38

In the process of carcinogenesis, there is a time point of cellular genome destabilisation, a frequent symptom of which is chromosomal aneuploidy of chromosomes 3, 7 and 17 and a loss of locus 9p21 (UroVysion Test). In the course of 5 years, we carried out 512 UroVysion tests; in 68%, the patients were referred for diagnostics of urinary bladder cancer, in 8.4% for therapeutic effect follow up, in 2% it was dysuria, in 2.7% haematuria, in 0.5% chronic, recurrent cystitis, other indications - 1.4%, no precise indications - 13.1%. Diagnostic criteria: 25 morphologically abnormal cells were evaluated. A result was regarded positive when more than 10 cells demonstrated a loss of 9p21 fragment (the presence of one or the loss of both signals) or if more than 4 cells showed polysomy of two chromosomes (among chromosomes 3,7,17) or if more than 10 cells indicated polysomy for one of the above-mentioned chromosomes. Positive results were obtained in 56% of the cases. In the group with negative results, our attention was drawn to a statistically higher percent of rearrangements in 9p21 region, compared to changes in chromosomes 3, 7, 17. That observation did confirm the known fact that the change in question is the earliest one in the process of carcinogenesis. What was a surprising new observation was that the change was found in the group with negative results.

### 2.P39

### Derivative (16)t(1;16)(q11;q11.1) in a case of Fanconi anemia with myelodysplastic syndrome and review of the literatüre

George Papaioannou, Maria Gkaitatzi, Michael Iskas, Chrysa Lalayanni, Riant Saloum, Aliki Tsompanakou, Zografia Lazarou, Olga Asteriou, Achilleas Anagnostopoulos, Anastasia Athanasiadou
G.Papanikolaou Hospital Hematology Thessaloniki, Greece
*Molecular Cytogenetics* 2017, **10(Suppl 1):**2.P39

Fanconi anemia (FA) is an autosomal recessive genetic disorder. FA patients have physical abnormalities, organ defects, and an increased risk of certain cancers. As there is a DNA repair defect, patients are more likely to develop bone marrow (BM) failure, myelodysplastic syndromes (MDS) and acute myeloid leukemia (AML). The cytogenetic clonal patterns in FA-related AML differs from this one in de novo AML. Presence of +1q, −7, +3q, 7q−, +13q, or 20q − are more frequent in a FA patient with AML. Unbalanced t(1;16), e.g. trisomy 1q and monosomy 16q, is a rare non-random cytogenetic abnormality associated with myelodysplastic syndrome. Duplication of the long arm of chromosome 1 has been mostly described as a secondary event in MDS, and has been preliminarily associated to cytogenetic and/or clinical progression toward AML. We present a 13 year-old girl with FA diagnosed with MDS in July 2013. Chromosome analysis on BM aspirate revealed:46,XX,add(1)(p34.3),add(6)(p22),der(16)t(1:16)(q11:q11)[3]/47,sl,+8[12]/46,sl,add(1)(p34.3)[1]/46,sld,del(12)(p11p13)[3]/46,XX[5]. The derivative chromosome 16, was described on the basis of fluorescent in situ hybridization result. The patient received treatment with anti-thymocyte globulin and then cyclosporine (CsA) for six months . She was progressed to AML in February 2014 and she was treated with Idarubicin hydrochloride and Cytarabine without response and then Mitoxantrone and Cytarabine. The patient died cause of bacterial sepsis in April 2014. The literature review revealed that der(16)t(1:16)(q11:q11) was found in three MDS cases evolving towards AML. As a sole anomaly in one MDS patient and with additional trisomy 8 in the two others. Because of the rarity of this cytogenetic abnormality, additional cases need to be studied before its clinical impact is fully appreciated.

### 2.P40

### The case study of agressive type of Mantle cell lymphoma characterized by hallmark t(11;14) and the most frequent secondary genetic events - deletion of TP53 and monoalellic and biallelic deletion of CDKN2A genes

Lucie Truhlikova[1], Martina Stoklasova[1], Bronislava Drevojankova[1], Libor Kudelka[2], Martin Brejcha[3], Andrea Ondrejkova[4]
[1]AGEL Laboratories Laboratory of Medical Genetic Novy Jicin, Czech Republic; [2]AGEL Laboratories Bioptic and Cytological Laboratory Novy Jicin, Czech Republic; [3]The Hospital of Novy Jicin Haemathology Novy Jicin, Czech Republic; [4]AGEL Laboratories Laboratory of Imunology Novy Jicin, Czech Republic
*Molecular Cytogenetics* 2017, **10(Suppl 1):**2.P40

Mantle cell lymphoma (MCL) is agressive type of lymphoma. The rearrangement of genes IGH and CCND1 is a crucial diagnostic marker to distinguished by the other lymphomas. Additional genetic aberrations occur in many cases and help assess thein prognostic importance.
Our case is 77 years old woman with suspected MCL. The classical cytogenetic analysis presents two patological clones with complex karyotype and common translocation t(11;14). FISH analysis detected bialellic and monoalellic deletion of CDKN2A, deletion of TP53 and gain of BCL6 and MYC. CDNK2A and MYC aberrations are associated with a high MCL international prognostic index (MIPI). CDKN2A/TP53 losses correlated with an unfavourable outcome. The examination was done from bone marrow and material from lymph node. Histologic biopsy identifies difuse growing lymphoma with nodular bearing. The cytology examination finds lymphoid elements with irregularly nucleolus of centrocytoid morphology. The clonal population of B-lymphocytes in peripheral blood and bone marrow is demonstrated by immunophenotyping.
MIPI score 7,2 of patient is associeted with high-risk prognosis with median survival 29 months. The combination of chemotherapeutic treatment R-CHOP/ R-ARAC and Rituximab was brought.
The patient died for central nervous system attack after 4 months.

### 2.P41
### Report on two rare Fanconi Anemia patients

Nikoletta Selenti [1], Antonis Kattamis [2], Konstantina Kosma [1], Stavroyla Kostaridoy [3], Sofia Kitsioy [1], Ariadni Mavroy [1]
[1]Athens University School of Medicine Department of Medical Genetics Athens-Greece; [2]Athens University School of Medicine Department of Pediatrics Athens-Greece; [3]Athens University, School of Medicin Pentelis General Children's Hospital Athens-Greece
**Correspondence:** Nikoletta Selenti
*Molecular Cytogenetics* 2017, **10(Suppl 1):**2.P41

**Background**
Fanconi Anemia (FA) is a rare genetic disorder that is mainly inherited in an autosomal recessive pattern and is rarely X-linked. Clinical features of FA patients include various congenital abnormalities, the classical features, however, may be absent in 25-40% of patients.
We report on two female patients, aged 1 and 4 years old referred to the Department of Medical Genetics of Athens University for genetic investigation due to clinical suspicion of FA.
**Methods**
Cytogenetic analysis
For clastogen-induced chromosome damage, lymphocyte cultures were set up with the addition of Mitomycin (MMC). The sample was considered as FA-positive if the percentage of breaks and radial formations detected were 7–10 times higher compared to an aged-matched control.
Molecular analysis
Molecular analysis was performed for FANCA by MLPA and Sanger sequencing for FANCA, FANCC, FANCE, FANCF and FANCG.
**Results**
**Cytogenetic analysis**
Induced breaks and radial formations were noted in 96% of analyzed metaphases in both patients.
**Molecular analysis**
The first patient was carrier of c.3521G > A,(p.W1174*) in exon 36 and c.3788_3790delTCT,(p.Phe1263del) in exon 38 of FANCA (FA-A subtype).
In the second patient a homozygous substitution, c.1313 T > C p.(Leu438Cys) in exon 7 of FANCE was revealed (FA-E subtype).
**Conclusion**
The c.3521G > A,(p.W1174*) in exon 36 of FANCA is a stop codon mutation, and is reported for the first time in the literature. Two out of five family members tested were heterozygotes.
In the FA-E patient, the c.1313 T > C p.(Leu438Cys) substitution in exon 7 of FANCE is also reported for the first time.The patient does not present any congenital abnormalities, and although she hasn't been transplanted, two years after the diagnosis, no typical hematological manifestation is apparent. Although both parents are Greeks, are not related and come from different areas of the country, they are carriers of the same rare substitution.

### Prenatal Diagnosis

### 3.P1
### Prenatal diagnosis of fetal aneuploidy and de novo copy number variation by isolating extravillus trophoblasts and nucleated fetal red blood cells from maternal circulation a proof of principle study using the automated Cell RevealTM platform based on nanostructure and microfluidics

Ming Chen [1], Chung-Er Huang [2], Frank Mau-Chung Chang [3]
[1]Changhua Christian Hospital, Department of Genomic Medicine, Changhua, Taiwan; [2] National Chiao-tung University, International College of Semiconductor Technology, Hsinchu, Taiwan; [3]University of California Los Angeles, Department of Electrical Engineering, Los Angeles, CA, USA
**Correspondence:** Ming Chen
*Molecular Cytogenetics* 2017, **10(Suppl 1):**3.P1

Non-invasive prenatal testing/screening (NIPT/NIPS) based on cell-free fetal DNA (cffDNA) in maternal circulation has been widely accepted worldwide since 2011, however, the limitations of cffDNA-based NIPT/NIPS, such as maternal malignancy and fetoplacental mosaicism, preclude its fully replacement of invasive prenatal diagnosis using amniocentesis and chorionic villus sampling. Despite the repertoire of NIPT/NIPS had been recently expanded into microdeletion/ microduplication disorders by a few commercialized service providers, the consensus of the academic communities still regards the scope of cff-DNA based NIPT/NIPS is limited to fetal aneuploidy involving common trisomies and monosomies. On the other hand, cell-based noninvasive prenatal diagnosis had been the aim of another group of researchers with the booming of new technologies originally developed to isolate and enrich the circulating tumor cells (CTCs). Here we present a nanostructure, microfluidics based novel automated system Cell RevealTM to demonstrate the feasibility of capturing fetal cells including both the extravillus cytotrophoblasts (EVT) and the nucleated fetal red blood cells (fnRBC), followed by cytogenetic analyses using fluorescence in situ hybridization (FISH), array comparative genomic hybridization (aCGH), and next generation sequencing (NGS), to detect fetal aneuploidy and de novo copy number variation (CNV). We also performed paralleled cffDNA-based NIPT/NIPS and confirmed the results with those obtained by invasive procedures.

### 3.P2
### Maternally inherited Xp;Yq translocation in male fetus

Piret Ilisson, Maria Keernik, Riin Klade, Kati Kuuse, Mari Sitska
United Laboratories, Tartu University Hospital, Department of Clinical Genetics, Tartu, Estonia
**Correspondence:** Piret Ilisson
*Molecular Cytogenetics* 2017, **10(Suppl 1):**3.P2

Xp;Yq translocations are relatively rare events and may be a challenge in genetic counseling. Female carriers of Xp;Yq translocation may be phenotypically normal or have short stature. In males, phenotypic consequences depend on the genes deleted in derivative X chromosome.
We report a prenatal case of Xp;Yq translocation in a male fetus. A 31-year old woman underwent amniocentesis at 15th week of pregnancy due to positive maternal serum screening test.

Cytogenetic analysis of the fetus showed male karyotype with additional material attached to Xp. Chromosomal microarray analysis (CMA) was performed to discover possible imbalances. It showed a 8.5 Mb homozygous terminal deletion of Xp from breakpoint Xp22.31 and an at least 12.5 Mb heterozygous duplication of Yq from region Yq11.221. The deleted region of Xp harbours 39 genes, including SHOX, ARSE, NLGN4 and STS. The loss of these genes may result in a contiguous gene syndrome with short stature, chondrodysplasia, mental retardation and ichthyosis in male individuals. The duplicated region of Yq is not expected to cause phenotypic manifestations. The karyotype of the fetus is 46,Y,der(X)t(X;Y)(p22.3;q11.2).
Cytogenetic investigation of the parents showed the same derivative X chromosome in the mother. The father´s karyotype was normal. So the mother of the fetus is a carrier of the Xp;Yq translocation. Her karyotype is 46,X,der(X)t(X;Y)(p22.3;q11.2). She is a phenotypically normal woman, but had a period of infertility and irregular menarche. She has no other children. Cytogenetic analysis of her parents gave normal results. So, she has a de novo translocation.
After genetic counseling the family decided to continue the pregnancy. A precise clinical examination and the follow-up of the baby will be offered to the family after birth.
**Consent to publish:** The authors confirm that written informed consent was received by the patient for publication.

### 3.P3
### The complete cytogenetic analysis of chorionic villi is a valuable tool to study early spontaneous miscarriages

Anna Soler, Ester Margarit, Yolanda Viedma, M. Angeles Villar, Antoni Borrell, Virginia Borobio, Aurora Sánchez
[1]Hospital Clínic, Servei De Bioquímica I Genètica Molecular, Barcelona, Spain; [2]Hospital Clínic, Bcnatal, Barcelona, Spain
**Correspondence:** Anna Soler
*Molecular Cytogenetics* 2017, **10(Suppl 1):**3.P3

Chromosomal abnormalities are the most frequent cause of early pregnancy losses. Thus, cytogenetic analysis of spontaneous miscarriages is essential to establish their etiology and to assess patients about risks of recurrence in future pregnancies. Conventional tissue culturing of products of conception collected after evacuation is laborious and impaired by bacterial contamination, and at risk of diagnostic error due to overgrowth of maternal cells present in the specimen. Recent studies using microarrays, which don't require cell culture, have proved to be useful to detect chromosomal abnormalities in these samples, but they also present limitations. We analyze the cytogenetic results obtained in a large series of first trimester miscarriages, using a diagnostic approach with a high success rate and no maternal contamination, and underline some advantageous aspects when comparing with the molecular studies. A total of 1119 chorionic villi samples were obtained before evacuation and karyotypes were performed after short-term culture (STC). In 603 samples a long-term culture (LTC) was also performed. Cytogenetic result could be achieved in 1011 samples (90.3%). An abnormal karyotype was detected in 70.3% of the samples. Single autosomal trisomy was the most frequent abnormality (64.6% of the abnormal cases), followed by triploidy (13.1%) and monosomy X (10.4%). Chromosome rearrangements were found in 5.2%, combined abnormalities in 8.9%, and placental mosaicism in 3.5% of the cases with STC and LTC performed. The 3.1% of the samples showed an unexpected chromosome rearrangement. Our study offers reliable information on the incidence and types of chromosome abnormalities and placental mosaicism in miscarriages. Compared to microarray-based studies, our series gives a higher rate of chromosome abnormalities causative of pregnancy loss, including poliploidies and structural abnormalities which can increase the recurrence risk.

### 3.P4
### Microdeletions and microduplication of uncertain significance found on the X chromosome in prenatal samples

Helena Peková, Radka Mansfeldová, Marie Trková, Věra Bečvářová, Jiří Horáček
Gennet S.r.o., Cytogenetic Laboratory, Prague, Czech Republic
**Correspondence:** Helena Peková
*Molecular Cytogenetics* 2017, **10(Suppl 1):**3.P4

Chromosomal microarray is increasingly used in prenatal diagnosis. However, higher resolution can bring problems when interpreting some copy number variants. Using SNP array and array CGH, more than 750 amniotic fluid and chorionic villi samples were investigated. In total, 15 cases of microduplication/microdeletion and 7 cases of variants of uncertain significance on the X chromosome were found.
Using databases, publications and familial genealogy, pathogenicity was not definitely proven in these cases. In one case, the pregnancy was terminated due to pathologic ultrasound findings, the remaining 6 pregnancies have continued.

### 3.P5
### Mosaicism for unbalanced chromosome 18 rearrangements in first trimester prenatal diagnosis

Ester Margarit, Aurora Sánchez, M. José Rubio, M. Angeles Vallecillos, Anna Soler
Hospital Clínic, Servei De Bioquímica I Genètica Molecular, Barcelona, Spain
**Correspondence:** Ester Margarit
*Molecular Cytogenetics* 2017, **10(Suppl 1):**3.P5

Confined placental mosaicism for a chromosome abnormality is detected in 1-2% of pregnancies undergoing first trimester prenatal diagnosis in chorionic villi samples (CVS), mostly aneuploidies. Mosaicism for structural rearrangements is a rarer event that complicates interpretation. Among these cases, those involving chromosome 18 are frequently found in CVS analysis. Subsequent fetal studies are needed to discriminate between a confined placental abnormality and a chromosome rearrangement involving fetal tissues, therefore with potential phenotype consequences. Here we report five cases with different fetal chromosome 18 structural abnormalities in mosaic, detected at CVS (short and long term culture) by karyotyping and FISH analysis. These rearrangements involved: 18p deletion, isochromosomes for 18p and for 18q, addition to 18p, and a ring chromosome 18. Additional fetal investigations using ultrasound scan, QF-PCR, FISH and/or chromosome analysis from amniotic fluid contributed to differentiate a confined placental mosaicism from a true fetal mosaic and allowed a correct prenatal diagnosis. Among five cases with chromosome 18 rearrangements, one of them underwent directly gestation termination, since fetal malformations were detected by ultrasound scan (case 1); in four cases, an amniocentesis was performed for additional fetal cytogenetic analysis. In one of this (case 2), karyotype was normal in 100 fetal amniocytes, therefore the rearrangement was considered confined to the placenta and a normal baby was born. In the three remaining gestations, however, the cytogenetic analysis performed in amniotic fluid cells confirmed an abnormal fetal karyotype: in cases 3 and 4, only one cell line with 18p deletion was found, while in case 5, the abnormal cell line found in CVS with an extra i(18p) was in mosaic with a normal cell line. Possible cytogenetic mechanisms for the different rearrangements and clinical consequences are discussed.

### 3.P6
### Clinical Experience with a Single Nucleotide Polymorphism Based Non Invasive Prenatal Test for Five Clinically Significant Microdeletions

Peter Benn [1], Sushma Iyengar [2], Zackary Demko [2], Kimberly Martin [2]
[1]University of Connecticut Health Center, Genetics And Genome Sciences, Farmington, CT, USA; [2]Natera, Inc., –, San Carlos, CA, USA
**Correspondence:** Peter Benn
*Molecular Cytogenetics* 2017, **10(Suppl 1):**3.P6

Single-nucleotide polymorphism (SNP)-based noninvasive prenatal testing (NIPT) can be used to screen for a subset of sub-chromosomal deletions (<10 Mb) associated with severe clinical phenotypes. Over a one-year period of test referrals to Natera, Inc. (San Carlos, CA), 74,938 tests were performed for fetal 22q11.2 deletion syndrome (22qdels) and 39,678 for 1p36, cri-du-chat, Prader-Willi, and Angelman microdeletion syndromes. The screen-positive rate (SPR) for 22qdels was 0.38%. Based on follow-up information on 54% of these cases, the false-positive rate (FPR) was 0.33% and the positive predictive value (PPV) was 15.7%. Six maternal 22qdels were identified. Similarly, for the other four microdeletion syndromes combined, the SPR was 0.59%, there was a 58% positive test follow-up information rate, 0.56% FPR, and 5.3% PPV, with zero maternal deletions. A minimal estimate for the prevalence was approximately 1/1,255 for 22qdels and 1/1,464 for 1p36, cri-du-chat, and Angelman syndromes combined. A protocol improvement was prospectively evaluated. This included reflex re-sequencing of positive call cases at a higher depth of read, which increased the PPV for 22qdels to 44.2% and lowered the FPR to 0.07%. For the other microdeletion syndromes combined, the PPV increased to 31.8%, and the FPR was reduced to 0.07%. Given that the original study was carried out on a high-risk cohort, modelling was performed to estimate PPVs for average-risk populations. The results show that the PPVs would remain similarly high.

The results of this study demonstrate that these microdeletions are relatively common in the test referral population, SNP-based screening is effective and the performance is improved with high-depth resequencing. Invasive testing with microarray analysis is indicated for all women with positive microdeletion screening test results.

### 3.P7

### Detection of cryptic chromosomal abnormalities and fetoplacental discrepancies after cytogenetic study of both placental layers

Aurora Sánchez [1], Ester Margarit [1], Virginia Borobio [2], Emma Peruga [1], Marc Papiol [1], Anna Soler [1]

[1]Hospital Clínic, Servei De Bioquímica I Genètica Molecular, Barcelona, Spain; [2]Hospital Clínic, Bcnatal, Barcelona, Spain

**Correspondence:** Aurora Sánchez

*Molecular Cytogenetics* 2017, **10(Suppl 1):**3.P7

The study of chorionic villi samplings (CVS) by conventional cytogenetics reveals chromosomal abnormalities in 12% of cases, and in 1-2% of pregnancies mosaicism is observed. In order to increase the diagnostic yield, new molecular techniques that offer higher resolution have been developed, such as chromosomal microarray-based analysis (CMA). Similar to cytogenetically visible chromosomal abnormalities, cryptic chromosomal abnormalities (CCA) may also be presented as confined placental mosaics, leading to misinterpretations. The purpose of the present work was to establish the frequency of CCA and confined placental mosaicism of CCA in CVS, and to evaluate the reliability of the strategy used.

We performed CMA in CVS of 90 pregnancies with normal karyotype or a balanced familial rearrangement, in both trophoblast and mesenchyme. Thirty-three percent of them belonged to the pathological group as they were referred for ultrasound abnormalities, and the remainder 67% was the control group referred mostly due to abnormal first trimester screening.

The overall frequency of reportable non-mosaic CCA was 7,8%, but only a 6,7% with phenotypic consequences, and in 1% of samples a CCA was only found in trophoblast. Most of the CCA were diagnosed in the control group.

Although the cohort presented is relatively small, it seems that the rate of CCA is higher than other previous prenatal diagnosis reports. Placental mosaicisms of CCA present a frequency similar to that of cytogenetically visible chromosomal abnormalities during the first trimester of pregnancy. However, CCA do not seem to be generally associated with ultrasound abnormalities in the first trimester of gestation. The strategy used is reliable for the detection of placental mosaicisms of CCA.

Partial results from the case study have been previously presented (Eur J Hum Genet 2015, Vol 23 Supp 1, 55)

### 3.P8

### Cystic hygroma in fetuses with normal and aneuploid karyotypes

Iryna Gordienko, Tetiana Nikitchina, Oksana Vashchenko, Olena Tarapurova, Andriy Velychko

Si «institute of Pediatrics, Obstetrics And Gynecology Nams of Ukraine», Department of Fetal Medicine, Kyiv, Ukraine

**Correspondence:** Oksana Vashchenko

*Molecular Cytogenetics* 2017, **10(Suppl 1):**3.P8

**Introduction**

Cystic hygroma is a vascular-lymphatic malformation and can occur either as an isolated finding or as a part of syndrome (chromosomal abnormality; Noonan, Smith–Lemli–Opitz, Escobar, Fanconi pancytopenia syndromes). The incidence of cystic hygroma is about 1:1000–1:6000 births.

Objective: The analysis of the ultrasonographic and karyotyping data of fetuses with cystic hygroma.

**Methods**

A complete fetal sonographic examination was performed, followed by fetal karyotyping. For cytogenetic analysis of biopsy samples a direct method of processing the villi was used and for fetal blood a halfmicromethod.

**Results**

A total of 48 (0,7%) fetuses with cystic hygroma were identified in 6919 high-risk pregnancies referred for fetal karyotyping. Cystic hygroma was diagnosed in 50% of the cases at 12–14 weeks of gestation.

A karyotype abnormality occurred in 29 (60,4%) of fetuses, including trisomy 21 (20,7%), monosomy X (62,1%), trisomy 18 (10,3%), and other (6,9%). Despite well-known association between maternal age and frequency of aneuploidy, there were only 4 (13,8%) pregnant women over 35 years among diagnosed aneuploid cases. The proportion of pregnant women under 25 years was 2 times higher than in other age groups among euploid fetuses with cystic hygroma.

Besides hygroma, associated congenital anomalies were observed in 12 (63,2%) of 19 fetuses with normal karyotype. Among them the most frequently diagnosed were cardiac abnormalities – 10 (83,3%) cases.

**Conclusion**

Cystic hygroma is associated with high rates of karyotype abnormality, major congenital malformations. Monosomy X was the most common form (62,1%) among aneuploid cases of cystic hygroma (n = 29). Only 7 (14,6%) fetuses were euploid and without ultrasonographic detectable structural anomalies.

### 3.P9

### Transfer of aneuploid embryos following preimplantation genetic diagnosis the added value of a haplotyping based genome wide approach

Cindy Melotte [1], Eftychia Dimitriadou [1], Sophie Debrock [2], Koen Devriendt [1], Thomy De Ravel [1], Eric Legius [1], Karen Peeraer [2], Joris Robert Vermeesch [1]

[1]University Hospital Leuven, Center For Human Genetics, Leuven, Belgium; [2]University Hospital Leuven, Leuven University Fertility Center, Leuven, Belgium

**Correspondence:** Cindy Melotte

*Molecular Cytogenetics* 2017, **10(Suppl 1):**3.P9

**Introduction**

Embryo selection for monogenic diseases has been mainly performed using targeted disease-specific assays. Recently we have developed haplarithmisis, which is based on genomic haplotype reconstruction of cell(s) biopsied from embryos. This provides information not only about the inheritance of Mendelian disease alleles, but also about numerical and structural chromosome anomalies and haplotypes genome-wide. Reflections on how to use this information in the diagnostic laboratory are lacking.

**Methods**

Here we present the clinical outcome of 164 embryo transfers following PGD for monogenic and/or chromosomal disorders using haplarithmisis. Given that (1) our main aim is selection against the genetic disorder that occurs in the family and (2) embryo selection is performed on day 3 biopsied material, we opted for a relaxed aneuploidy testing model, according to which only embryos carrying viable trisomies and trisomies of meiotic origin are excluded from transfer. Embryos were approved for transfer based on (1) the haplotype in the region of interest, (2) genome-wide copy number profile and (3) embryo morphology at the blastocyst stage.

**Results**

From January 2015 until December 2016, 164 embryos had been transferred in 152 single and 6 double embryo transfers. This led to 44 singleton and 2 twin pregnancies which gives a pregnancy rate of 30% per embryo transfer. Interestingly, 38 embryos with one or more aneuploidies had been included in the transfers, 6 of which gave rise to a clinical pregnancy and resulted in the birth of 4 healthy babies and 2 ongoing pregnancies.

**Conclusions**

Haplarithmisis allows the distinction of meiotic and mitotic trisomies enabling the use of embryos that would have otherwise been discarded as inappropriate for embryo transfer. With regard to pregnancy outcome, our data show an improved pregnancy rate per embryo transfer in case of chromosomally normal embryos (33% versus 15.8%). Importantly, a remarkable 15.8% of the aneuploid embryos seem to be leading to normal pregnancies and life births, stressing the value of correct interpretation of detected aneuploidies on day 3. Both findings underline the added value of genome-wide based haplotyping used in preimplantation genetic diagnosis.

### 3.P10

### Rare Autosomal Trisomies detected through NIPT a relevant secondary incidental finding

Nicolas Chatron [1], Marie Luino [2], Sylvie Vono [2], Carine Abel [3], Elisabeth Ollagnon [3], Marie Noelle Bonnet Dupeyron [4], Claire Bardel [5], Marie Pierre Cordier [6], Renaud Touraine [7], Axel Fichez [3], Cyril Huissod [3], Océane Lablanche [8], Jérôme Massardier [8], Eudeline Alix [2], Audrey Labalme [2], Marianne Till [2], Damien Sanlaville [1], Caroline Schluth-Bolard [1]
[1]Hospices Civils De Lyon And Crnl Inserm U1028 Cnrs Umr5292 Ucbl1, Laboratoire De Cytogénétique Constitutionnelle And Gendev Team, Lyon, France; [2]Hospices Civils De Lyon, Laboratoire De Cytogénétique Constitutionnelle, Lyon, France; [3]Hospices Civils De Lyon, Centre Pluridisciplinaire De Diagnostic Prénatal, Hôpital De La Croix Rousse, Lyon, France; [4]Centre Hospitalier De Valence, Service De Génétique, Valence, France Hospices Civils De Lyon And Santé, Université Claude Bernard Lyon 1, Service De Biostatistique; [5]Bioinformatique And Umr Cnrs 5558, Lbbe, Équipe Biostatistique, Lyon, France; [6]Hospices Civils De Lyon, Service De Génétique Médicale, Lyon, France; [7]Chu De Saint-Etienne, Service De Génétique Médicale, Saint-Etienne, France; [8]Hospices Civils De Lyon, Centre Pluridisciplinaire De Diagnostic Prénatal, Hôpital Femme Mère Enfant, Lyon, France
**Correspondence:** Nicolas Chatron
*Molecular Cytogenetics* 2017, **10(Suppl 1):**3.P10

After the discovery of circulating cell free fetal DNA, non-invasive prenatal testing (NIPT) for Down syndrome was permitted by massively parallel sequencing technologies with low-pass whole genome sequencing. Since 2015, we have been performing NIPT using a pangenomic strategy with a semiconductor sequencer (H+ Consortium) and WISECONDOR for data analysis.

After 1000 tests, we have identified 20 trisomies 21, 1 trisomy 18 all confirmed after invasive testing, no trisomy 13 and 6 other autosomal aneuploidies. A trisomy 15 was suspected for a 45 years-old patient. Amniotic fluid karyotype was normal but molecular testing demonstrated maternal uniparental heterodisomy for chromosome 15 (Prader-Willi syndrome) leading to termination of the pregnancy while ultrasound scans were normal. 4 patients had a NIPT profile suspicious of trisomy 16. Patients were informed considering potential consequences on fetal growth during the last trimester and complications leading to intrauterine fetal death (ACLF Guidelines). None of them had an invasive test but two pregnancies are still ongoing. The two others were complicated by intrauterine growth delay for the first one and preeclampsia for the second one with respective births at 34 and 27 weeks of gestation. Postnatal karyotype was normal in both cases with mosaic placental trisomy 16 demonstrated for the only placenta sample available. Finally, a trisomy 8 was suspected for a 42 years old patient who refused invasive testing considering normal ultrasound scans and was later lost to follow-up. With 6 cases over 1000 tests, we are close to the attended 0.7% all-chromosome confined placental aneuploidy probability based on chorionic villus sampling data. Ideally, all observations should be confirmed postnatally on a placental sample. Herein we stress the interest of a pangenomic strategy and the role of cytogeneticists facing situations experienced with chorionic villus sampling.

### 3.P11 Microarray

### Incidental X Linked Findings A female fetus with a gain in the DMD gene

Bárbara Marques [1], Silvia Serafim [1], Sónia Pedro [1], Cristina Ferreira [1], Laurentino Simão [1], Cristina Alves [1], Mónica Viegas [1], Marisa Silva [1], Filomena Brito [1], Marta Amorim [2], Joaquim Correia [3], Hildeberto Correia [1]
[1]Instituto Nacional De Saúde Doutor Ricardo Jorge, I.p., Departamento De Genética Humana, Lisboa, Portugal; [2]Hospital D. Estefânia, Centro Hospitalar Lisboa Central, Serviço De Genética Médica, Lisboa, Portugal; [3]Maternidade Dr. Alfredo Da Costa, Centro Hospitalar Lisboa Central, Centro De Diagnóstico Pré-natal, Lisboa, Portugal
**Correspondence:** Bárbara Marques
*Molecular Cytogenetics* 2017, **10(Suppl 1):**3.P11 Microarray

In prenatal diagnosis, chromosomal microarray analysis (CMA) has not yet fully replaced conventional cytogenetic but has rapidly become the recommended genetic test in pregnancies with ultrasound abnormalities. This methodology allows the identification of pathogenic small copy number variation (CNVs) in 5-10% of pregnancies with ultrasound abnormalities and a normal karyotype, increasing the diagnostic yield. However, this increased resolution can also result in the detection of incidental findings.

Here we report a fetus referred for prenatal diagnosis due to skeletal dysplasia. Affymetrix Cytoscan HD chromosome microarray analysis was performed and a 204 kb gain was detected at Xp21.1 region (chrX: 31993622_32191110 [GRCh37]) in a female fetus, encompassing the intron 44 of the DMD gene, for the largest gene transcript. Nevertheless, if we considered the smaller transcripts it encompasses exon 1. The gain was maternally inherited.

The DMD gene is involved on Becker muscular dystrophy, Cardiomyopathy, dilated, 3B and Duchenne muscular dystrophy. Intron 44 is a preferential breakpoint in about 30% of all DMD deletions, being the DMD transcript NM_004006.2 responsible for dystrophin expression in the skeletal muscle.

The FGFR3 gene sequencing revealed the presence of the c.1118A > G, p.Y373C mutation associated to Thanatophoric Dysplasia, type 1 (TD1) justifying the ultrasound abnormalities.

With this case, we reinforce that the discovery of CNVs in prenatal CMA goes beyond the correlation with the CNV and the ultrasound abnormalities. Incidental findings can also have a larger impact to the family clinical managing, even if not for the ongoing pregnancy for the reproductive future of the couple.

### 3.P12

### 19p13.12 Microdeletion in a fetus with Facial Dysmorphism Renal Hypotrophy and Placental Anomalies

Minh-Tuan Huynh [1], Lucie Tosca [1], François Petit [2], Jelena Martinovic [3], Virginie Benoit [4], Frédéric Parisot [2], Aline Receveur [4], Loïc Drévillon [4], Gérard Tachdjian [1], Sophie Brisset [1]
[1]Hôpital Antoine Béclère, Hôpitaux Universitaire Paris-sud / Université Paris-sud, Unité De Cytogénétique, Service D'histologie, Embryologie Et Cytogénétique, Clamart, France; [2]Ap-hp Hôpital Antoine Béclère, Hôpitaux Universitaire Paris-sud, Laboratoire De Génétique Moléculaire, Clamart, France; [3]Ap-hp Hôpital Antoine Béclère, Hôpitaux Universitaire Paris-sud, Unité De Fœtopathologie, Clamart, France; [4]Ap-hp Hôpital Antoine Béclère, Hôpitaux Universitaire Paris-sud, Unité De Cytogénétique, Service D'histologie, Embryologie Et Cytogénétique, Clamart, France
**Correspondence:** Minh-Tuan Huynh
*Molecular Cytogenetics* 2017, **10(Suppl 1):**3.P12

Microdeletion 19p13.12 has been rarely reported. Only five post-natal cases with intellectual disability, dysmorphism and overlapping deletions involving the 19p13.12 chromosome region have been documented. Two critical intervals were previously defined: one of 700 kb for branchial arch defects and another interval of 350 kb for hypertrichosis, synophrys, protruding front teeth. We describe the first prenatal case, a fetal death in utero at 39 weeks of gestation presenting dysmorphic features, cupped ears and pre-auricular skin tag, bilateral renal hypotrophy and placental pathology with chorionic villous ischemia. 180 K array-CGH analysis identified a 745 kb microdeletion in 19p13.12. FISH analyses with probes RP11-79P23, RP11-81 K14 and a control 9p telomeric probe showed that the deletion occurred de novo. The maternal inheritance of the deletion was demonstrated by molecular studies.
Included within the 745 kb microdeletion in 19p13.12 are at least 6 functionally relevant genes: SYDE1, NOTCH3, BRD4, AKAP8, AKAP8L, WIZ, encompassing both previously reported critical intervals. SYDE1, primarily expressed in placental trophoblasts, is an important candidate gene for placental abnormalities observed in this case. Syde1 −/− KO mice placenta exhibit placental defects associated with intrauterine growth restriction. NOTCH3 is strongly expressed in renal blood vessels, the glomerular tuft, cytotrophoblasts and placental endothelial cells. NOTCH3 (MIM *600276), gene mutation in CADASIL syndrome with ischemic strokes and white matter lesions, is another candidate gene for renal hypotrophy and placental ischemia in this fetus. BRD4, AKAP8, AKAP8L, WIZ highly expressed in branchial arches are candidate genes for ear malformations in the fetus. We report a new interstitial deletion at 19p13.12 and also refine more precisely a critical interval for renal hypotrophy, branchial arch defects, facial dysmorphism and placental pathology.

### 3.P13

### Twin IVF pregnancy with a balanced de novo translocation (5;17) in one fetus a case report

Gerly Kilusk [1], Triin Mölter-Väär [1], Piret Ilisson [1], Konstantin Ridnõi [2], Riina Zordania [1]
[1]Tartu University Hospital, Department of Clinical Genetics, United Laboratories, Tallinn, Estonia; [2]East-Tallinn Central Hospital, Women's Clinic, Tallinn, Estonia
**Correspondence:** Gerly Kilusk
*Molecular Cytogenetics* 2017, **10(Suppl 1):**3.P13

We report a case of twin IVF pregnancy with a balanced de-novo translocation between chromosomes 5 and 17 in one fetus. The couple underwent several cycles of IVF treatments and the last one (used method was ICSI) resulted in a dichorionic diamniotic pregnancy. Extensive ultrasound investigation of the twins at 13 weeks of pregnancy revealed a microretrognathia in one fetus. The other had no visible abnormalities (both fetuses had NT = 2,0 mm). Subsequently, the chorionic villus sampling was performed to the first fetus. Cytogenetic studies by G-banding revealed a karyotype 46,XX,t(5;17)(q22;q23). Parental karyotypes were normal.
Chromosomal microarray analysis (CMA) was performed to exclude imbalances. No alterations were detected. In case of a balanced translocation that is ascertained by chromosomal microarray analysis, the predicted outcome of the pregnancy can be expected as normal. Pregnancy is going on and a follow-up of the newborns was proposed.
Translocations, when detected prenatally, remain a challenge for the geneticists. Molecular approaches often give additional beneficial information in the counseling process.

### 3.P14

### The Karyotype did not die

Celine Dupont [1], Jonathan Levy [1], Jonathan Rosenblatt [2], Severine Drunat [3], Sandra Bhattacharjee [1], Aurelie Marc [1], Marie-Adeline Thaly [1], Cecile Le Long [1], Kathleen Guiraud [1], Berethe Diarra [1], Eva Pipiras [4], Brigitte Benzacken [4], Anne-Claude Tabet [5]
[1]Hôpital Robert Debré (ap-hp), Chu Paris, Unité De Cytogénétique, Département De Génétique, Paris, France; [2]Hôpital Robert Debré (ap-hp), Chu Paris, Service De Gynécologie Obstétrique, Paris, France; [3]Hôpital Robert Debré (ap-hp), Chu Paris, Unité De Biologie Moléculaire Et Biochimie, Département De Génétique, Paris, France; [4]Hôpital Jean Verdier,université Paris 13, Sorbonne Paris Cité, Umr 1141, Unité De Cytogénétique, Bondy, France; [5]A)hôpital Robert Debré (ap-hp), Chu Paris-b) Institut Pasteur, A)unité De Cytogénétique,département De Génétique-b) Human Genetics And Cognitive Functions, Paris, France
**Correspondence:** Celine Dupont
*Molecular Cytogenetics* 2017, **10(Suppl 1):**3.P14

In recent years, it has been positively demonstrated that molecular methods represent an improved diagnostic tool for prenatal detection of chromosome abnormalities.
We report two prenatal cases where molecular diagnosis failed while conventional cytogenetic leads to the diagnosis in these fetuses.
The first patient was addressed at 26 WG to our prenatal center because of a congenital heart defect observed on ultrasonography. Amniocentesis and fetal blood sampling were performed. Prenatal BoBs on amniotic fluid did not find any aneuploïdy. Three days later, metaphases from fetal blood showed 46 chromosomes with one dicentric chromosome 21 in all the metaphases. Rapidly, amniotic fluid culture showed also the presence of this dicentric in all metaphases resulting in fetal trisomy 21. Surprisingly, FISH on uncultured amniotic fluid revealed the existence of two cellular populations in equivalent proportion: one with the dicentric (trisomy 21) and one with only one chromosome 21 (monosomy 21). Prenatal BoBs is an assay based on quantitative dosage. In this case, the co-existence between the two populations compensate for chromosome 21 dosage making Trisomy 21 invisible to the molecular diagnose.
The second pregnant patient was referred because of ventriculomegaly and abnormal corpus callosum in one twin. Amniocentesis was performed. Prenatal BoBs and CGH-array did not reveal any abnormality but karyotype analysis showed a fetal apparently balanced de novo translocation:46,XX,t(16;17)(p13;p13.3). Fetal brain RMI revealed lissencephaly. Interruption of 17p13.3 region spanning LIS1/PAFAH1B1 gene by the translocation breakpoint was demonstrated by FISH and explained the Miller-Dieker brain phenotype of this fetus.
These two cases illustrate the interest and contribution of morphological cytogenetic methods, such as FISH, associated with these purely quantitative molecular techniques. However, the molecular techniques alone can lead to a false negative diagnosis at first, thus very difficult for parents once the diagnosis is established.

### 3.P15

### Androgenetic biparental chimerism in prenatal diagnosis a case report

Mc Pinto [1], Lm Pires [1], A Mascarenhas [1], A Jardim [1], M Val [1], P Paiva [1], Jb Melo [2], Im Carreira [2]
[1]Faculty of Medicine, University of Coimbra, Cytogenetics And Genomics Laboratory, Coimbra, Portugal; [2]Faculty of Medicine, University of Coimbra, Cytogenetics And Genomics Laboratory; Cimago – Center of Investigation On Environment, Genetics And Oncobiology, Coimbra, Portugal
**Correspondence:** Mc Pinto
*Molecular Cytogenetics* 2017, **10(Suppl 1):**3.P15

Chimerism occurs when two or more stable and genetically distinct cell lines, originated independently from one another, are present in an individual. Sex-chromosome discordant chimeras are rare in humans, with only a few cases reported and investigated at molecular level.
We report a prenatal diagnosis performed at 16 weeks because of hyperechogenic bowel, intrauterine growth restriction (IUGR) and an abnormal appearing placenta. The karyotype of amniocytes revealed two different cell lines: one 46,XX and another 46,XY. The fetus was female and maternal contamination was discarded. Combination of the cytogenetic result and molecular analysis with polymorphic DNA markers was compatible with the presence of two cell lines: one biparental and another androgenetic. These results were confirmed in skin fibroblasts after termination of pregnancy. The molecular study was crucial to differentiate between a chimera and a mosaic and also to propose a mechanism for its origin.
Phenotypic expression in sex-discordant chimeras is highly variable, depending on the distribution of the two cell lines in the gonads, and may range from normal to various degrees of ambiguous genitalia. The presence of an androgenetic cell line in these cases is frequently associated with placental mesenchymal dysplasia. The associated fetus may be normal, but IUGR and features common to Beckwith-Wiedemann syndrome (BWS) may occur.
Prenatal genetic counseling is difficult in cases of chimerism, since it is not possible to predict the phenotype at term. The possibility of sexual ambiguity, infertility and the possibility of developing gonadoblastoma should be discussed. Chimeras may be phenotypically normal males/ females and since there are a limited number of cases studied, some authors assume chimeras may be under-diagnosed. Chimerism is an interesting biological issue with the genotype-phenotype correlation still far from being defined.

### 3.P16

### Prenatal Diagnosis of two rare inv(22) different at molecular level

Jb Melo [1], Mc Pinto [2], A Jardim [2], N Lavoura [2], Lm Pires [2], P Paiva [2], F Ramos [3], L Simões [2], Im Carreira [1]
[1]Faculty of Medicine, University of Coimbra, Cytogenetics And Genomics Laboratory; Cimago – Center of Investigation On Environment, Genetics And Oncobiology, Coimbra, Portugal; [2]Faculty of Medicine, University of Coimbra, Cytogenetics And Genomics Laboratory, Coimbra, Portugal; [3]Pediatric Hospital, Coimbra Hospital And Universitary Centre Epe, Department of Medical Genetics, Coimbra, Portugal
**Correspondence:** Jb Melo
*Molecular Cytogenetics* 2017, **10(Suppl 1):**3.P16

Inversions are frequently innocuous. Nevertheless, rarely, it may induce a submicroscopic alteration, with a locus or a small number of loci being disrupted or removed.
Excluding those involving the heterochromatic regions and others considered harmless variants, inversions are uncommon, with a frequency range from about 0,12% to 0,7% (pericentric) and 0,1% to 0,5% (paracentric).
We report two cytogenetically identical pericentric inversions on chromosome 22(p13q11.21), prenatally detected. To guarantee the integrity of proximal region on chromosome 22, including the critical region for DiGeorge syndrome, MLPA (P250) was performed. Family 1 showed a normal result but in Family 2 a small deletion in 22q11.21 of about 600Kb was found. This deletion, as well as the pericentric inversion was inherited from the father.
Inversions of chromosome 22 are rare with only 8 cases reported. All involve a larger segment, with the breakpoint in 22q in a more distal position than it is in our two cases.
The deletion identified in Family 2 is an atypical deletion in the distal part of the "22q11.2 microdeletion syndrome" critical region and shows a great inter- and intrafamilial phenotypic variability, with some patients reported in Decipher database with deletions that overlap the one we report.
The inv(22) seems to be a rare event. However, this may be due to an under diagnosis considering the difficulty in identifying the recombinant chromosome by conventional cytogenetic. The accurate characterization of these alterations requires the use of molecular techniques.
The two inv(22) we report were inherited from apparently normal parents. Even in those situations the integrity of the 22q11.2 region must be guaranteed, since it contains a cluster of LCR that mediates non-allelic homologous recombination that results in deletions/ duplications in the region.

### 3.P17

### Three incidental findings of non invasive prenatal screening by whole genome analysis

Angelique Kooper [1], Esther Sikkel [2], Gwendolyn Woldringh [1], Dominique Smeets [1]
[1]Radboudumc, Human Genetics, Nijmegen, The Netherlands; [2]Radboudumc, Obstetrics And Gynaecology, Nijmegen, The Netherlands
**Correspondence:** Angelique Kooper
*Molecular Cytogenetics* 2017, **10(Suppl 1):**3.P17

Non-invasive prenatal testing (NIPT) approaches, based on whole genome sequencing of circulating free DNA (cfDNA) has become more and more standard prenatal care. A whole genome analysis enables not only the detection of fetal chromosomal imbalances of chromosomes 13, 18 and 21 but (partial) aneuploidies of all other chromosomes can be detected as well. Because fetal cfDNA originates from the apoptosis of placental trophoblast cells, there is also a chance of a positive screening result attributable to fetoplacental mosaicism. Moreover, an abnormal result from NIPT may also be caused by a vanishing twin or even from malignant cells of an occult maternal malignancy. We present three incidental findings detected in our diagnostic setting during the Dutch NIPT study TRIDENT-1 (Trial by Dutch laboratories for Evaluation of Non-Invasive Prenatal Testing): a double aneuploidy (A), a segmental duplication (B) and an unbalanced translocation (C) and demonstrate additional pre- and postnatal follow-up testing elucidating the origin of the finding.
Case A: cfDNA screening showed a combination of trisomy 15 and 21. Case B: cfDNA screening showed a duplication of 9p. Case C: in the first trimester cfDNA screening was done (elsewhere) indicative for a structural duplication of 7p and a small deletion of 13q.
From April 2017, in the Netherlands cfDNA screening is implemented as first screening test for all pregnant women (national TRIDENT-2 study) and parents will have the option to choose between targeted (chromosome 13, 18, and 21 only) or whole genome analysis. Based on the experience of analysis of chorionic villus samples and the results of NIPT in TRIDENT-1 thusfar, we expect that more incidental findings will be detected. Because of their possible clinical relevance and for pregnancy management, extensive laboratory testing and clinical follow-up is needed to clarify these findings.

### 3.P18

### Can we give up Karyotyping for prenatal diagnosis and use CMA only A debate

Esther Manor [1], Shirly Amar [2], Livnat Mazor [2], Paulina Kachko [2]
[1]Ben Gurion University of The Negev, Shraga Segal Department of Microbiology, Immunology And Genetics, Beer-Sheva, Israel; [2]Sorika University of The Negev, Human Genetics, Beer-Sheva, Israel
**Correspondence:** Esther Manor
*Molecular Cytogenetics* 2017, **10(Suppl 1):**3.P18

Chromosomal Microarray Analysis (CMA) is becoming more useful in prenatal diagnosis(PND) than karyotyping in the last years. Some laboratories exclusively use CMA for PND. Karyotyping is still needed for couples experiencing multiple abortions and for oncology cases.
Here we present 3 examples which emphasise the CMA limitations.
Example No. 1: a case showed about 18 mega-bases deletion of chromosome 9p24.3p22.1, and about 22 mega-bases duplication of chromosome 12p13.33p12.1. We assumed that the fetus inherited an unbalanced translocation. Karyotyping of the parents revealed that the mother is carrying a complex translocation 46,XX,t(5;12;9)(p15.1;p12;p22).
Example No. 2: a case with de-novo balanced translocation t(3;10)(p14.1;p13), with normal CMA result. Missing the translocation causes misleading counselling.
Example No. 3: misleading counselling in 2 cases where we missed low level mosaicism (20-30%) of 45,X.
In summary, although CMA is considered an advanced and potent technique in PND, yet it still has some limitations, as it still lacks data regarding many unknown deletions and duplications which are defined as: variants of unknown significance (VOUS) or no other specified (NOS). Also, while we know exactly where the deletions are located we cannot know definitely, where the duplications are located. It also misses balanced translocation and low level mosaicism as shown here. This decreases our ability to give more precise counseling. It is suggested to keep doing CMA routinely, without giving up totally karyotyping in PND.

### 3.P19

### Non invasive prenatal testing for aneuploidies implementation in Estonia

Olga Žilina [1], Martin Sauk [1], Lauris Kaplinski [1], Eva-Liina Ustav [2], Maire Peters [3], Andres Salumets [3], Ants Kurg [1]
[1]University of Tartu, Institute of Molecular And Cell Biology, Tartu, Estonia; [2]Tartu University Hospital, Women's Clinic, Tartu, Estonia; [3]Competence Centre On Health Technologies, Competence Centre On Health Technologies, Tartu, Estonia
**Correspondence:** Olga Žilina
*Molecular Cytogenetics* 2017, **10(Suppl 1):**3.P19

Conventional prenatal screening for fetal aneuploidies relies on biochemical and sonographic measurements in the first and second trimesters followed by invasive confirmatory test for women who are deemed to be at high risk. Noninvasive prenatal testing (NIPT) is an expanding clinical application in which circulating cell-free DNA (cfDNA) in the pregnant woman's blood is analysed, preferably by next-generation sequencing (NGS), to detect potential fetal chromosomal aneuploidies.
Our aim is to introduce NIPT in clinical practice in Estonia.
We collected blood samples from pregnant women with high-risk of fetal aneuploidies who undergo an invasive aneuploidy test (Group I; n = 203) and blood samples from pregnant women with no indications for an invasive procedure (Group II; n = 174). Massive parallel single-end sequencing of cfDNA was performed on the NextSeq500 platform (Illumina, Inc). Data analysis were performed using an in-house developed analysis algorithm in which time- and resource-consuming sequencing read mapping is replaced by composing and counting k-mer lists.
To date, 343 samples were analysed. Five trisomies 21, five trisomies 18, and one trisomy 13 were detected among Group I pregnancies, which was in concordance with the invasive test results. No aneuploidies were found among Group II pregnancies. The samples collection and analysis is ongoing to estimate the sensitivity and specificity of the method before it can be offered as a screening test.
In general, it was demonstrated that NIPT provides greater sensitivity and specificity compared to traditional aneuploidy screening programs while reducing the number of invasive procedures. This makes NIPT an attractive alternative to the current possibilities in prenatal diagnostics.

### 3.P20

### The Role of Mosaicism in Discordant cf DNA Testing Results

Seher Basaran [1], Birsen Karaman [1], Melike Kirgiz Aytan [2], Tahir Dehgan [2], Ibrahim Halil Kalelioglu [3], Recep Has [3], Atil Yuksel [3]
[1]Istanbul Medical Faculty, Medical Genetics, Istanbul, Turkey; [2]Center For Genetic Diagnosis, Premed, Istanbul, Turkey; [3]Istanbul Medical Faculty, Obstetrics And Gynecology, Istanbul, Turkey
**Correspondence:** Seher Basaran
*Molecular Cytogenetics* 2017, **10(Suppl 1):**3.P20

cf-DNA testing is the most sensitive and specific noninvasive prenatal screening test for trisomy 21 (T21). However, the test performance for other common aneuploidies is lower as compared to T21. The false positive and negative results of this test are associated with technical limitations and biological factors. The mosaicism either in the fetus or placenta is the most important biological factor of these discordant results.
Here, we report the cytogenetic follow up studies of eight cases with discordant cf-DNA results. Two cases were positive for monosomy X (MX) by cf-DNA test, but 45,X/46,XX mosaicism has been found in amniotic fluid cells. Follow-up studies in term placentas exhibited a third cell line (47,XXX) in both cases.
A mosaic trisomy 21 has been detected in amniotic fluid cells of a case with normal cf-DNA test result but abnormal ultrasound findings. Following the termination of the pregnancy, the placental studies showed that the rates of trisomic cells were different in 4 regions.
A monozygotic diamniotic twin pregnancy with negative cf-DNA test has been karyotyped due to the pathological ultrasound findings in one of the twin pairs. Amniocentesis revealed trisomy 13 (T13) in affected fetus, while twin pair was normal. Term placenta work-up revealed mosaicism.
Cytogenetic work-up in term placentas of four unconfirmed cases (two positive for MX, one case for T21 and one for T13) revealed normal results.
All together, placental studies showed that the mosaicism was the causing factor in 50% of discordant cf-DNA testing results.
Term placentas in all discordant cf-DNA cases and mosaic fetal karyotypes should be investigated cytogenetically to understand the underlying biological mechanism of the discordant results and to determine the role of mosaicism, which is necessary for the genetic counseling.

### 3.P21

### Familial translocation 21 22 follow up

Marinela Vasilevska [1], Elizabeta Ivanovska [1], Slobodan Drakulevski [2], Gordana Dimeska [3]
[1]Clinical Hospital Acibadem Sistina, Diagnostic Laboratories, Skopje, Macedonia; [2]Clinical Hospital Acibadem Sistina, Perinatology-Department For High Risk Pregnancy, Skopje, Macedonia; [3]Faculty of Natural Sciences And Mathematics, Institute of Biology, Skopje, Macedonia
**Correspondence:** Marinela Vasilevska
*Molecular Cytogenetics* 2017, **10(Suppl 1):**3.P21

Here we present three prenatal cytogenetic diagnoses from four pregnancies in a mother carrying a balanced reciprocal translocation 46,XX,t(21;22)(q22.3;q11)pat.
First, amniocentesis was performed due to ultrasound abnormalities of CNS. Fetal karyotype was 47,XY,+der(22)t(21;22)(q22.3;q11)mat. This karyotype is the result of a 3:1 meiotic segregation in the oocyte. The karyotype results in partial duplication 22 from pter to q11 and partial duplication of the terminal segment of chromosome 21 distal to q22.3. Chorion biopsy (CVS) was performed in the second pregnancy, resulting in the karyotype 46,XX,+der(21)t(21;22)(q22.3;q11)mat,-22. This unbalanced karyotype resulted from an adjacent 2 segregation with partial duplication of chromosome 21 and partial deletion of chromosome 22. The third pregnancy ended in spontaneous abortion without cytogenetics. The fourth pregnancy followed by chorion biopsy (CVS) showed a normal karyotype. This karyotype is the result of alternate segregation in mothers gametogenesis. Array CGH was suggested in the subsequent amniocentesis, but ultrasound examinations are in normal range and a normal outcome is expected.
These results showed three different types of meiotic segregation in gametogenesis of mother carrier of familial balanced translocation involving chromosome 21 and chromosome 22.

### 3.P22

**Array analyses find 40% more chromosomal aberrations than karyotyping and increase diagnostic yield to explain foetal ultrasound abnormalities**

Beike Leegte, Katelijne Bouman, Trijnie Dijkhuizen, Ron Suijkerbuijk, Harke Alkema, Joke Verheij, Nicole Corsten-Janssen, Birgit Sikkema-Raddatz
Univeristy Medical Centre Groningen, Genetics, Groningen, The Netherlands
**Correspondence:** Beike Leegte
*Molecular Cytogenetics* 2017, **10(Suppl 1):**3.P22

Karyotyping of chorionic villi and/or amniotic fluid cells was long used to detect chromosomal abnormalities when foetal ultrasound abnormalities were seen. However, the resolution of such karyotyping techniques is low. In 2011 we therefore introduced array analysis, to increase the resolution from approximately 8 Mb to 150 kb and here show that the use of array techniques increased our diagnostic yield. Array analysis was only offered after genetic counselling and when both parents were available for interpretation of copy number variations (CNVs) and uniparental disomy (UPD).
In the last six years, 2011–2017, we analysed 852 samples, first with CGH array [Agilent 180 K] and later with an 850 K SNP array [Illumina Omni Express] including UPD analysis (for chromosomes 6, 7, 11, 14 and 15). Deletions larger than 150 kb and duplications larger than 200 kb were reported. A CNV was defined as a polymorphism when it was present in our department database (at 0.5%) and in the Database of Genomic Variants. A CNV was considered causative if it was not seen in the parents and/or the literature described similar ultrasound abnormalities. Known pathogenic CNVs that did not explain the ultrasound findings were considered as incidental findings.
No chromosomal abnormalities were seen in 789 samples (93% of total). The array results explained the ultrasound findings in 52 samples (6%), while incidental findings were seen in 11 samples (1%). Karyotyping would have missed 21 of the causative CNVs.
Thus, using arrays instead of karyotyping led to an extra 40% of causally related chromosomal abnormalities being detected.

### 3.P23

**Two crazy stories of placental mosaicism in prenatal samples**

Zuzana Dobsakova [1], Jitka Kadlecova [1], Diana Nikulenkov Grochova [1], Marketa Hiemerova [1], Monika Matyasova [1], Lucie Kupcikova [1], Jitka Sojkova [1], Radka Pirklova [1], Eva Popelinska [1], Ilga Grochova [2], Ishraq Dhaifalah [3]
[1]Cytogenetics Laboratory Brno, Cytogenetics Laboratory Brno, Brno, Czech Republic; [2]Centre of Prenatal Diagnosis, Centre of Prenatal Diagnosis, Brno, Czech Republic; [3]Centre of Fetal Medicine And Medical Genetics, Centre of Fetal Medicine And Medical Genetics, Olomouc, Czech Republic
**Correspondence:** Zuzana Dobsakova
*Molecular Cytogenetics* 2017, **10(Suppl 1):**3.P23

We are presenting case reports where we used several different techniques (QF PCR, array CGH, karyotyping, FISH and MLPA) to find “the true” in our prenatal samples.
Case 1: Noninvasive test (NIPT) for the most common aneuploidies was performed in the gravidity of 29 years old woman at 14 weeks of pregnancy with low PAPP-A but normal ultrasound (US). NIPT called chromosome 13 as “inconclusive”. At 17 weeks the pregnancy led to a miscarriage. Due to the abnormal result of NIPT we wanted to determine the status of chromosome 13 in the fetus and placenta. The result of QF PCR and standard karyotyping of the fetus was normal female. On the other hand, in placenta, QF PCR did not find aneuploidies of chromosomes 13, 18, 21 but ratios of STR markers on chromosome X were either uninformative or inconclusive. Cultivated cells from the placenta were of poor quality and contaminated in ~50% with maternal cells (confirmed by QF PCR). In native placental DNA a monosomy of chromosome X in mosaic using array CGH was detected. Indeed, deep reanalysis of contaminated cultured cells revealed several cells with monosomy X.
Case 2 is more complicated and will be clearly demonstrated on poster. Shortly, chorionic villi (CVS) were biopsied from a 24 year old woman at 13 weeks of pregnancy with abnormal US. Array CGH analysis of uncultured CVS revealed three pathogenic aberrations on chromosomes 11 and 13 but FISH (on cultured cells) showed a discrepant result. Furthermore, array CGH on cultivated CVS found different aberrations on the same chromosomes in accordance to FISH. Due to the discrepant results from cultivated/uncultivated CVS, amniotic fluid was taken at 18 weeks of pregnancy. Finally, array CGH analysis of native amniotic fluid confirmed the result of cultivated CVS.

### 3.P24

**Sequential Combined test second trimester maternal serum markers and circulating fetal cells to select women for invasive prenatal diagnosis**

Paolo Guanciali-Franchi [1], Chiara Palka [1], Elisena Morizio [1], Giulia Sabbatinelli [1], Melissa Alfonsi [2], Donatella Fantasia [3], Giammaria Sitar [1], Peter Benn [4], Giuseppe Calabrese [1]
[1]University of Chieti, Medical, Oral And Biotechnological Sciences, Chieti-Italy; [2]Ausl Chieti, Obstetrics And Pediatrics, Chieti-Italy; [3]Ausl Pescara, Hematology, Pescara-Italy; [4]University of Connecticut Health Center, Genetics And Genome Sciences, Farmington-USA
**Correspondence:** Paolo Guanciali-Franchi
*Molecular Cytogenetics* 2017, **10(Suppl 1):**3.P24

From January 1st 2013 to August 31st 2016, 24408 pregnant women received the first trimester Combined test and contingently offered second trimester maternal serum screening to identify those women who would most benefit from invasive prenatal diagnosis (IPD). The screening was based on first trimester cut-offs of ≥1:30 (IPD indicated), 1:31 to 1:899 (second trimester screening indicated) and ≤1:900 (no further action), and a second trimester cut-off of ≥1:250. From January 2014, analysis of fetal cells from peripheral maternal blood was also offered to women with positive screening results. For fetal Down syndrome, the overall detection rate was 96,6% for a false-positive rate of 2.8% resulting in an OAPR of 1:11 (equivalent to a positive predictive value of 8.1%). Additional chromosome abnormalities were also identified resulting in an OAPR for any chromosome abnormality of 1:6 (PPV 14.1%). For a sub-set of cases with positive contingent test results, FISH analysis of circulating fetal cells in maternal circulation identified 7 abnormal and 39 as normal cases with 100% specificity and 100% sensitivity. We conclude that contingent screening using conventional Combined and second trimester screening tests is effective but can potentially be considerably enhanced through the addition of fetal cell analysis.

### 3.P25

**Prenatal CNV classification and reporting survey from 60 European centres**

Katrina Rack [1], Daniela Giardino [2], Ingrid Simonic [3], Javier Suela [4], Kris Van Den Bogaert [5], Luca Lovrecic [6], Sian Morgan [7], Silvana Guerneri [8], Rosalind Hastings [1]
[1]Oxford University Hospitals NHS trust CEQAS Oxford, UK; [2]IRCCS Istituto Auxologico Italiano Laboratorio di Citogenetica Milano, Italy; [3]Addenbrooks Hospital Medical genetics laboratory Cambridge, UK; [4]NIM Genetics Clinical genetics laboratory Madrid, Spain; [5]Gasthuisberg Centre for Human genetics Leuven, Belgium; [6]University medical centre Clinical institute of medical genetics Ljubliana, Slovenia; [7]University hospital of wales Institute of medical genetics Cardiff, Wales; [8]Ca Ospedale Maggiore Policlinico Fondazione IRCCS Milano, Italy
**Correspondence:** Katrina Rack
*Molecular Cytogenetics* 2017, **10(Suppl 1):**3.P25

In 2016 the participants of the CEQAS (Cytogenomic External Quality Assessment Scheme) prenatal array EQA were invited to participate in an on-line survey to review how they would classify a series of copy number variants (CNVs).
A total of sixty out of eighty contacted laboratories participated. Nine case scenarios were prepared by the EQA assessors who agreed in advance the expected classification of the presented CNV.
The laboratories were supplied with a referral card, clinical details and the nature, size and genomic co-ordinates of the reported CNV and asked to classify each CNV according to the American College of Medical Genetics standards and guidelines for reporting copy number variants as either: pathogenic', 'likely pathogenic', 'uncertain significance', 'likely benign', and 'benign'. The expected outcome for the CNVs as assessed by the assessors was: 3x 'pathogenic', 1x 'likely pathogenic', 2x 'uncertain significance', 1x 'likely benign' and 1x 'benign'. There was a good concordance of classification for the pathogenic and benign CNVs but variation for the classification of the other categories. This survey highlights the need for established guidance for reporting CNVs consistently between laboratories in a prenatal setting. The findings of the survey will be presented.

### 3.P26
### A dicentric chromosome 18 detected with NIPT

Ann Van Den Bogaert[1], Nathalie Fieremans[1], Toon Janssen[1], Minh Tri Do[2], Kathelijn Keymolen[1]
[1]UZbrussel Medical Genetics Brussels, Belgium; [2]Brugmann Prenatal Medicine Brussels, Belgium
*Molecular Cytogenetics* 2017, **10(Suppl 1):**3.P26

A 27 year old woman performed a Non Invasive Prenatal Test (NIPT) at a gestational age of 13 weeks + 4 days. The indication for performing NIPT was the personal request of the patient since PAPP-A testing did not reveal a high risk for trisomy 13,18 or 21.
NIPT analysis, showed no trisomy 13, 18 and 21. Nevertheless, we found an indication for a duplication on chromosome 18 including the centromere. However, NIPT is unable to distinguish between maternal and fetal aberrations.
Using array CGH, a 9.5 Mb duplication of chromosome bands 18p11.22-q11.2 was detected in the blood of the mother. This duplication explains the NIPT result and encompasses 23 genes, including GATA6, MC2R, PTPN2 and AFG3L2. At first, we thought about the presence of a marker chromosome, however FISH analysis on interphase cells could not confirm this hypothesis. After conventional karyotyping and FISH on chromosomes and in combination with the array result, we detected a dicentric chromosome 18 with a 9.5 Mb duplication of chromosome bands 18p11.22-q11.2, namely 46,XX,der(18)dupl(18)p11.2q11.2. In the meanwhile, the mother was seen at the genetics clinic and she presented with mild intellectual disability and had always been in a special education program. This duplication can thus explain the phenotypic features of the mother.
After genetic counselling, the patient decided to refrain from invasive prenatal procedures and at this moment, we do not know if the fetus has inherited this duplication or not. Postnatal samples will be requested.
We report on a dicentric chromosome 18 with a 9.5 Mb duplication of chromosome bands 18p11.22-q11.2, illustrating that the diagnostic yield of NIPT analysis can reach beyond the frequent aneuploidies.
The importance of using the combination of tests for genetic diagnosis is also illustrated. A follow-up after birth is needed, including micro-array analysis in the child.

### Animal and Plant Cytogenetics

### 4.P1
### Structure and regulation of holocentric chromosomes

Andreas Houben [1], Stefan Heckmann [1], Andre Marques [2], Jankowska Maja [1], Veit Schubert [1], Jörg Fuchs [1], Wei Ma [1], Andrea Pedrosa-Harand [2], Jiri Macas [3], Pavel Neumann [3], Gerhard Wanner [4]
[1]'Leibniz Insitute of Plant Breeding and Crop Plant Research, Gatersleben, Germany; [2]Federal University of Pernambuco, Botany, Recife, Brazil; [3]Institute of Plant Molecular Biology, Biology Centre as, Biology, Olomouce, Czech Republic; [4]Biozentrum Der Ludwig-Maximillians-Universität München, Biology, München, Germany
**Correspondence:** Andreas Houben
*Molecular Cytogenetics* 2017, **10(Suppl 1):**4.P1

This abstract is not included here as it has already been published.

### 4.P2
### First attempt to use the combined method of immunofluorescence and DNA FISH to study surface antigens in bovine X and Y spermatozoa

Ruth Mikelsaar [1], Andres Salumets [1], Aavo-Valdur Mikelsaar [1], Ülle Jaakma [2], Triin Hallap [3]
[1]Competence Centre On Health Technologies, Department of Reproductive Medicine, Tartu, Estonia; [2]Estonian University of Life Sciences, Institute of Veterinary Medicine And Animal Sciences, Tartu, Estonia; [3]Estonian University of Life Sciences, Department of Reproductive Biology, Tartu, Estonia
**Correspondence:** Ruth Mikelsaar
*Molecular Cytogenetics* 2017, **10(Suppl 1):**4.P2

Combined method of immunofluorescence and FISH (immuno-FISH) is a unique method to assess relations between DNA and proteins at the single cell level. This approach has some descriptions for human and animal somatic cells, but lacking for sperm cells. Our goal was to develop a combined method for animal, namely bovine sperms, and to analyze the expression of a surface antigen transglutaminase (TGM1) on X- and Y-chromosome bearing cells.
We have modified and simplified existing protocols and to our knowledge give for the first time a method of simultaneous visualization of immunostained surface antigen on the X- or Y-chromosome bearing bovine sperms identified by FISH. The method was applied to analyze sperms of 4 Holstein bulls. Immunostaining of TGM1 was performed with the bovine specific polyclonal anti-TGM1 antibody (LSBio, Inc., USA) and FISH using DNA-probes specific for bovine X- and Y-chromosome (Empire Genomics LLC, IDLabs, NY). In total 5823 cells were visualized and images analyzed. Our results showed that the ratios between X- and Y- spermatozoa were normal, ranged 0.98-1.1 ($p > 0.05$) in different subjects. The TGM1 was clearly expressed with variable intensity on the surface both of X- and Y-sperms. It was more frequently observed on X-cells being statistically significant ($p < 0.05$) in one bull. Some individual differences were detected between the bulls.
In conclusion, the developed immuno-FISH method has enabled us to analyze on the same image every X- or Y-chromosome bearing bovine spermatozoa also for surface antigen immunoreaction. We have found that among individual bulls TGM1 was differentially expressed on X- or Y-spermatozoas being slightly more frequent on X-cells.
Supported by project ETKY 616215780004.

### 4.P3
### Molecular Cytogenetics of Owl Monkeys
Naiara Pereira De Araújo [1], Valéria Do Socorro Pereira [2], Giorgia Chiatante [3], Roscoe Stanyon [3], Marta Svartman [1]
[1]Universidade Federal De Minas Gerais, Biologia Geral, Belo Horizonte, Brazil; [2]Fundação Zoobotânica De Belo Horizonte, Seção De Mamíferos, Belo Horizonte, Brazil; [3]Universitá Degli Studi Di Firenze, Biologia, Firenze, Italy
**Correspondence:** Marta Svartman
*Molecular Cytogenetics* 2017, **10(Suppl 1):**4.P3

Molecular cytogenetic data show that owl monkeys (genus Aotus) are characterized by rapid chromosome evolution. The eleven species currently recognized have diploid numbers ranging from 2n = 46 to 58, but even species with the same diploid number can differ dramatically. We analyzed a male and female of wild captured Aotus infulatus from the Fundação Zoobotânica de Belo Horizonte, Brazil. The diploid numbers were 2n = 49 (male) and 50 (female). Chromosome painting identified 11 conserved HSA chromosomes (HSA 6, 9, 12, 13, 17, 18, 19, 20, 21, 22 and X). Other chromosomes were fragmented into two or three segments and 14 associations were found: HSA 1/3, 1/16, 2/7, 2/20, 3/21, 4/15, 5/7, 5/15 (twice), 7/11, 8/18, 10/11, 10/22, 15/14/15/14 and 16/22. The Y chromosome was translocated to AIN16 (HSA 3a). Hybridizations with BAC-clones shed light on its evolutionary history, revealing that, after the fusion, the AIN16/Y chromosome underwent a pericentromeric inversion followed by a paracentric inversion. Moreover, we found evidence of satellite DNA relicts where the chromosome Y centromere was originally located, suggesting that it was inactivated sometime after the fusion. A comparison with the chromosome painting pattern of three other species of Aotus and the putative ancestral Platyrrhini karyotype (APK) allowed us to propose an ancestral Aotus complement with 2n = 52.
Financial support: CNPq, CAPES, PRIN 2012

### 4.P4
### Here be dragons chromosomes flow sorting of Varanus komodoensis for sequencing and genome anchoring
Alessio Iannucci [1], Martina Johnson Pokorná [2], Marie Altmanová [2], Michail Rovatsos [2], Lukáš Kratochvíl [2], Ivan Rehák [3], Jorge Claudio Pereira [4], Malcolm Ferguson-Smith [4], Claudio Ciofi [1]
[1]University of Florence, Department of Biology, Florence, Italy; [2]Charles University In Prague, Faculty of Science, Prague, Czech Republic; [3]Prague Zoological Garden, Zoological Garden, Prague, Czech Republic; [4]University of Cambridge, Cambridge Resource Centre For Comparative Genomics, Cambridge, UK
**Correspondence:** Alessio Iannucci
*Molecular Cytogenetics* 2017, **10(Suppl 1):**4.P4

The study of reptilian genomes is crucial for the accurate investigation of genome evolution across amniotes. So far the genomes of only thirteen reptile species have been sequenced and published. Although draft assemblies are often all that are required to answer some important biological questions, a greater level of resolution is needed for more detailed research into evolutionary processes. This can be achieved through building high-quality chromosomal maps and anchoring genomes to chromosomes.
We report on a preliminary work aimed at identifying the chromosome sequences of the Komodo dragon Varanus komodoensis, the world's largest lizard, using an integrated approach based on flow sorting and next generation sequencing (NGS). We isolated V. komodoensis chromosomes by flow sorting, assigned each peak to a pair of chromosomes by FISH, and finally amplified chromosome-specific DNA pools for NGS sequencing.
The resultant data will provide necessary database for anchoring newly sequenced and assembled Komodo dragon genome sequences to chromosomes, helping to provide a better insight into the evolution of squamate genomes. Integration of our results with chromosome painting data will allow the comparison of genome organization among squamates and facilitate the reconstruction of the ancestral amniote karyotype. Moreover, the production of high-quality genomic information will be of great benefit to the identification of molecular markers for further understanding of the evolutionary history of wild Komodo dragon populations.

### 4.P5
### Chromosome structural variation in grape genome unlocking the genetic potential of table grape varieties towards the design of new water conservation programs in sustainable viticulture
Claudia Rita Catacchio [1], Maria Francesca Cardone [2], Carlo Bergamini [2], Rocco Perniola [2], Fiammetta Alagna [2], Silvia Rotunno [2], Pietro D'addabbo [1], Can Alkan [3], Fabio Anaclerio [1], Giorgia Chiatante [1], Annamaria Marra [1], Giuliana Giannuzzi [4], Donato Antonacci [2], Mario Ventura [1]
[1]University of Bari, Dep. of Biology, Bari, Italy; [2]Research Unit For Viticulture And Enology In Southern Italy, Crea-utv, Turi, Italy; [3]Bilkent University, Department of Computer Engineering, Ankara, Turkey; [4]University of Lausanne, Center For Integrative Genomics, Lausanne, Switzerland
**Correspondence:** Claudia Rita Catacchio
*Molecular Cytogenetics* 2017, **10(Suppl 1):**4.P5

Grapevine (Vitis vinifera L.) significant role in fruit and wine production makes it one of the most interesting crop plants existing, particularly from a genomic point of view.
We recently published the first inter-varietal atlas of structural variation (SV) and single nucleotide variants (SNVs) for the grapevine genome, obtained combining high throughput biotechnologies and bioinformatics tools (Cardone et al., 2016) . Coming from the comparison among four different grape varieties, our data disclosed genomic variations of which the effects are involved in inter-varietal, phenotypic differences.
Among these, differences in gene dosages likely playing critical roles in response to biotic and abiotic stresses, such as drought, have been showed. Due to the effects of climate change, many arid and semi-arid areas will be particularly vulnerable to the risk of a significant reduction of their water resources. Besides, table grapes vegetative growth cycle requires high water consumption; thus, cultivation of varieties with lower water requirements are needed to prevent the disappearance from some of its current areas of cultivation. We then integrated our inter-varietal atlas of structural variations and single nucleotide variants with agronomic, chemical and transcriptome analyses of table grapes exposed to different levels of water stress. Our data allowed us to identify specific genes and metabolic pathways differentially involved in the adaptation of the plant to water stress. Our work provided the assessment of the variability of drought resistance related to genotype and selection of table grape varieties having a high tolerance to such stress.
Cardone, M. F., et al. (2016). "Inter-varietal structural variation in grapevine genomes." Plant J 88(4): 648–661.

### 4.P6
### A comprehensive overview on Equus caballus segmental duplications
Nicola Lorusso [1], Claudia Rita Catacchio [1], Francesco Maria Calabrese [1], John Huddleston [2], Evan E. Eichler [2], Mario Ventura [1]
[1]University of Bari, Department of Biology, Bari, Italy; [2]University of Washington School of Medicine, Department of Genome Sciences, Seattle, WA, USA
**Correspondence:** Nicola Lorusso
*Molecular Cytogenetics* 2017, **10(Suppl 1):**4.P6

Segmental duplications (SDs), also known as low-copy repeats (LCRs), are stretches of DNA that typically share a high level of sequence similarity (≥90%) and map to two or more locations. In-tandem or interspersed SDs may contain genes and common repeats. In order to create an integrated view of SDs evolution among mammalian genomes, we here aim to identify and characterize the pattern of SDs in one of principal species within the Boreoeutheria group: Equus caballus. Our approach may help in defining breakpoints related to SDs in horse species and at the same time to refine the assembly looking at specific loci enriched in duplications. Both the whole horse genome and 50 selected BAC clones have been inspected for SDs content by using two in silico approaches: the whole-genome shotgun sequence detection (WSSD) and the whole-genome assembled comparison (WGAC). BAC clones were fully sequenced both by Illumina and PacBio platforms. Fluorescence in situ hybridization (FISH) technique allowed us to validate and characterize predicted segmentally duplicated regions. The merged output of WGAC and WSSD methods revealed that 4.56% (113.4Mbp) of the whole horse genome is made of SDs. The 82% of these in silico predictions were confirmed by cytogenetic analyses. Contrarily to what observed in human genome, horse in-tandem duplications appear to be 3 times more abundant than interspersed ones. Flanking SDs regions were also inspected for gene content using NCBI RefSeq gene database. Our results showed the existence of 26 different genes among which major histocompatibility complex (MHC) gene or gene belonging olfactory receptor family. Thanks to this reliable dual approach, high level duplicated regions, difficult to sequence and assemble, may be solved.

### 4.P7

### The chromosomal abnormalities as causes of reproductive failure in buffalo females

Ioana Nicolae [1], Adrian Bota [2], Daniela Vidmichi [1], Cosmin Sonea [3]
[1]R&d Institute For Bovine, Cytogenetics, Balotesti, Romania; [2]R&d Station For Buffalo, Reproduction, Sercaia, Romania; [3]Usamv, Fmv, Bucuresti, Romania
**Correspondence:** Ioana Nicolae
*Molecular Cytogenetics* 2017, **10(Suppl 1):**4.P7

Cytogenetic investigation is an excellent check system for the evaluation of the health and genetic control of breeding animal farms. In veterinary practice the most common requirement for a cytogenetic examination is related to fertility investigations. This work presents our observations concerning the role of chromosomal abnormalities as causes of reproductive failure in subfertile buffalo females. Considering these premises, the karyotype analysis of 36 Romanian buffaloes belonging to the R&D Station for Buffalo, Sercaia has been performed. In this study we identified 4 buffalo females with abnormal configuration of the chromosomal complement, represented by a large number of mono-and bichromatidic breakages on autosomes and heterosomes, loss of chromosome fragments and gaps. Our investigation continued through SCEs-test and for animals with many chromosomal breakages the number of sister chromatid exchanges (SCEs) was very high (11–23 SCEs/cell) compared to the normal animals. Although the carriers have had a normal phenotype, the analysis of their reproductive activity revealed a degradation of the reproductive performances characterized by repeated inseminations, lack of oestrus and loss of pregnancy. According to these results the chromosomal instability, identified in four of the 36 investigated buffalo females, demonstrated once again the role of these structural defects in the ethiology of different levels of infertility.

**Acknowledgements**
This study was supported by the Sectorial Project 5.3.3./2015

### 4.P8

### The toad headed agamas (Phrynocephalus) are champions in crossing over rate

Artem Lisachov [1], Evgeniya Solovyeva [2]
[1]Institute of Cytology And Genetics Sb Ras, Lab. of Recombinational And Segregational Analysis, Novosibirsk, Russia; [2]Moscow State University, Zoological Museum, Moscow, Russia
**Correspondence:** Artem Lisachov
*Molecular Cytogenetics* 2017, **10(Suppl 1):**4.P8

Recombination rate and crossover distribution along chromosomes vary considerably between the animal species. Several hypotheses explaining this variation were developed. However, most data on recombination patterns were obtained from mammals. The variation in recombination is strongly dependent on karyotypic diversity of the species comprising this taxon.
Squamate reptiles present a good model to study recombination due to more conservative karyotypes. Using immunolocalization of SYCP3, the protein of the axial elements of synaptonemal complex; MLH1, the mismatch-repair protein associated with the mature recombination nodules; and the centromere proteins we investigated chromosome synapsis and recombination at pachytene in two closely related species of iguanian lizards: the toad-headed agamas Phrynocephalus guttatus and Ph. alpherakii.
The karyotypes of the studied specimens consisted of 23 acrocentric bivalents: 12 pairs of macrochromosomes and 11 pairs of microchromosomes (2n = 46 FN = 46). Average SC length was equal to 223.9 ± 31.7 mkm (compared to 140–150 mkm in Anolis, another iguanian). We found that the toad-headed agamas show very high recombination rates, on average 50.1 ± 17.5 MLH1 foci per cell (compared to 21–22 in Anolis). The distribution of the crossovers along the chromosomes was almost even, not showing centromere effect and interference. This is in strict contrast with other vertebrates, which show prominent peaks of recombination near the telomeres and a decrease in recombination near the centromere.
This intense and evenly distributed recombination would lead to very low linkage disequilibrium in the population, efficient selection, and high genetic diversity. These lizards live in very harsh desert conditions. Perhaps high recombination serves to ensure efficient adaptation to their extreme habitat.

### 4.P9

### Modern methods of karyotyping in bitches and a case report of mosaicism

Dana Pusta [1], Rodica Sobolu [2], Melinda Szallai [3], Alexandra Tabaran [4], Camelia Raducu [5]
[1]Usamv Cluj-Napoca, Faculty of Veterinary Medicine, Department of Genetics, Cluj-Napoca, Romania; [2]Usamv Cluj-Napoca, Faculty of Horticulture, Department of Statistics And Mathematics, Cluj-Napoca, Romania; [3]Laboratory of Cytogenetics, 1-St Clinic of Obstetrics And Gynecology, Cluj-Napoca, Cluj-Napoca, Romania; [4]Usamv Cluj-Napoca, Faculty of Veterinary Medicine, Department of Food Sciences, Cluj-Napoca, Romania; [5]Usamv Cluj-Napoca, Faculty of Animal Husbandry, Cluj-Napoca, Romania
**Correspondence:** Dana Pusta
*Molecular Cytogenetics* 2017, **10(Suppl 1):**4.P9

The purpose of this study is to present the modern methods of karyotyping, using MetaSystem Ikaros in bitches and the presentation of a case report in a bitch with mosaicism. To fulfil this goal, classical techniques were used in order to visualise chromosomes in lymphocytes sampled form peripheral blood. The metaphases were analysed, both in classical Giemsa staining and in G-banding. The microscope which was used in the study was a motorized Zeiss Axioscop 2 with a capture camera connected to a computer and MetaSystem Ikaros software. Processing of the chromosomes is done in two stages: the first one allows us to capture the metaphase and to separate and overlap the chromosomes and the second stage assigs the chromosomes in pairs and allows their magnifying and extra- staining. Several studies were carried out on different bitches for classical routine examinations. Among these cases there was one bitch, a Golden retriever, which presented abnormal oestrus cycle and in which hormonal therapy was without any result. The cytogenetical studies led us to the diagnosis of 77, XO - 78, XX mosaicism. Utilization of MetaSystem Ikaros software is very facile compared to classical karyotyping methods and for this reason we recommend it to be used in animals with increased biological value or for diagnosis purposes in the cases where classical therapy has no effect.

### 4.P10

### Lampbrush chromosome microdissection as an approach to comprehensive investigation of heterochromatic chromosome regions

Anna Zlotina [1], Nadezda Kosyakova [2], Antonina Maslova [1], Thomas Liehr [2], Alla Krasikova [1]
[1]Saint Petersburg State University, Department of Cytology And Histology, Saint-Petersburg, Russia; [2]Jena University Hospital, Institute of Human Genetics, Jena, Germany
**Correspondence:** Anna Zlotina
*Molecular Cytogenetics* 2017, **10(Suppl 1):**4.P10

Heterochromatic chromosome regions are commonly represented by repetitive sequences of different nature, which makes them difficult for careful cytogenetic analysis. Previously, we showed that the approach of giant lampbrush chromosome (LBC) microdissection allows to explore comprehensively various chromosomal loci as small as an individual chromomere. In the present study we applied LBC microdissection to investigate heterochromatic chromosomal blocks of Japanese quail (Coturnix c. japonica), a galliform species closely related to chicken. Particularly, using microdissection technique we isolated centromeric chromomeres from macrobivalents and the material of heterochromatic short arms from submetacentric microbivalents. The distribution of these sequences in karyotype was investigated by FISH on metaphases and lampbrush chromosome spreads. The hybridization pattern indicated that in contrast to chicken machrochromosomes, which are known to possess chromosome-specific centromere sequences, quail macroautosomes share common centromere repeats. Besides, comparative FISH with dissected material of quail centromeric regions to chicken chromosomes supported our previous data on centromere repositioning events during Galliform karyotype evolution.
Heterochromatic arms of microchromosomes also proved to accumulate common DNA sequences that homogeneously constitute the whole material of the arms. Present findings together with earlier obtained data on epigenetic status and polymorphic nature of such segments allow us to believe that this specific heterochromatin is formed by some interspersed repetitive retroelements. Interestingly, the same sequences as well as the repeats from centromeric regions of macrochromosomes seem to compose the heterochromatic sex chromosome W, whose genetic nature has not been characterized yet. Finally, we investigated three-dimensional architecture of isolated repeats in quail interphase nucleus and discuss its involvement in radial genome organization during interphase.
The work was supported by a grant of the President of the Russian Federation (MK-1630.2017.4). Technical support: Resource Centers Molecular and cell technologies", "Chromas" of SPbU.

### 4.P11

### Filling in the gaps in the chicken sex W chromosome map

Alsu Saifitdinova [1], Svetlana Galkina [2], Maria Kulak [3], Elena Koshel [3], Aleksandr Dyomin [3], Raisa Chetverikova [4], Alexey Komissarov [5], Stephen J. O'brien [5], Elena Gaginskaya [3]
[1]International Centre of Reproductive Medicine, Laboratory of Assisted Reproductive Technology, Saint-petersburg, Russia; [2]Saint-petersburg State University, Department of Genetics And Biotechnology, Saint-Petersburg, Russia; [3]Saint- Petersburg State University, Department of Cytology And Histology, Saint-Petersburg, Russia; [4]Saint- Petersburg State University, Department of Vertebrate Zoology, Saint-Petersburg, Russia; [5]Saint-Petersburg State University, Theodosius Dobzhansky Centre For Genome Bioinformatics, Saint-Petersburg, Russia
**Correspondence:** Alsu Saifitdinova
*Molecular Cytogenetics* 2017, **10(Suppl 1):**4.P11

In birds, females are heterogametic sex (ZW) and males are homogametic (ZZ). The Z chromosome is very similar in size, gene density and AT-content to macrochromosomes. The chromosome W is small, gene poor and late replicating. It is detectable as a W-heterochromatic body in the interphase nucleus. In evolution, avian W chromosome, as mammalian male-specific Y-chromosome, has undergone extensive degradation and accumulated repetitive DNAs. It's estimated that the chicken W chromosome could harbour 44 Mb of repetitive DNA including at least 6 Mb of unknown repetitive sequences. The chicken W chromosome assembly has been currently found to contain 26 genes being of only about 7.08 Mb in Galgal5 of expected 50–55 Mb. Unlike other sequenced sex chromosomes chicken W does not contain genes specifically expressed in reproductive tissues.
In this work we succeed in annotation of high copy number tandem repeats from unassembled short raw reads. Tandem repeat (GGAAA)n has been identified and found to be the second most abundant in the chicken genome. Also we have shown that it forms expanded arrays on the both arms of the chicken W chromosome. In autosomes the repeating units (GGAAA)n were identified within promoter regions of some sex specific protein coding genes.
Together with previously studied EcoRI, XhoI, SspI high copied sequences our finding not only fills the gaps in the chicken sex W chromosome map but also highlights the importance of repetitive sequences and invites further exploration into their role in sex determining in birds.
Financial and technical support: RFBR #16-04-01823, Research Resource Centre "Chromas" and Theodosius Dobzhansky Centre for Genome Bioinformatics of Saint Petersburg State University.

### 4.P12

### Construction of physical maps of chromosomes in brachiaria spp. beginning a journey

Thais Nani[1], James Schnable[2], Jacob Washburn[3], Patrice Albert[3], Welison Pereira[1], James Birchler[3], Fausto Souza Sobrinho[4], Vânia Techio[1]
[1]Federal University of Lavras, Biology, Lavras-Brazil; [2]University of Nebraska Lincoln, Biological Sciences, Nebraska-USA; [3]University of Missouri, Biological Sciences, Columbia-USA; [4]Embrapa Dairy Cattle, Agronomy, Juiz De Fora-Brazil
*Molecular Cytogenetics* 2017, **10(Suppl 1):**4.P12

Brachiaria spp. (Poaceae) includes forage species distributed across tropical and subtropical regions. Cytogenetic analysis demonstrates different ploidy levels and intraspecific variability in chromosome morphology and in number of rDNA sites. Repetitive DNA sequences have been widely used in cytogenetic analysis. However, the use of low copy sequences is little explored. Analysis on the sequences that comprise the centromere in Brachiaria spp. chromosomes, as well as detailed karyotyping with localization of specific sequences in Brachiaria decumbens are lacking. The present study aimed to contribute to the construction of physical maps of chromosomes of Brachiaria brizantha, B. decumbens and B. ruziziensis. This was done using repetitive sequences from 5S and 45S rRNA, centromeric retrotransposons (CRs), and low copy genes, for which probes were developed using RNAseq and sequencing data. With detailed karyotypes of the three species, it was possible to identify the chromosomal pairs, and to observe heteromorphic pairs, as well as intra- and interspecific differences in the number and size of the ribosomal DNA sites. CRs were not identified in all chromosomes of the species studied, but low copy genes were in synteny with ribosomal gene sites, which was essential for the identification of certain chromosome pairs. In these Brachiaria species, some chromosomes with conserved segments were observed. Rearrangements involving the rDNA sites and chromosomal breaks were part of the karyotype diversification of the species studied. The use of genomic sequencing data was of fundamental importance in enabling the detailed cytogenetic analysis.
Financial support: Capes, FAPEMIG AND CNPq

### 4.P13

### The duplication events have traced the evolution of vitellogenin gene family

Maria A. Biscotti [1], Marco Barucca [1], Alberto Pallavicini [2], Marco Gerdol [2], Ettore Olmo [1], Adriana Canapa [1]
[1]Università Politecnica Delle Marche, Department of Life And Environmental Sciences, Ancona, Italy; [2]Università Di Trieste, Department of Life Sciences, Trieste, Italy
**Correspondence:** Maria A. Biscotti
*Molecular Cytogenetics* 2017, **10(Suppl 1):**4.P13

The genes of vitellogenin, from which the main egg-yolk proteins of oviparous animal species arise, are an excellent example to understand how a gene family origins and has been changing during the evolutionary history. In this study several sequences belonging to vertebrates, included those of the "fossil species" coelacanth and lungfish, have been investigated through phylogenetic and microsyntenic analyses. The results provide new insights into the evolutionary history of this gene family that was characterized by different/independent duplication events occurred in teleosts, basal sarcopterygians, amphibians, and amniotes. Moreover the sequences obtained in the coelacanth Latimeria, compared to those of other oviparous vertebrates, present conserved domains but also some sites similar to those of teleosts and others to those of tetrapods, emphasizing the intermediate characteristics of this living fossil.

### 4.P14

### The evolution of sex chromosomes in reptiles

Martina Johnson Pokorná[1,2]
[1] Faculty of Science, Charles University, Prague, Czech Republic; [2] Institute of Animal Physiology and Genetics, The Czech Academy of Sciences, Liběchov, Czech Republic
*Molecular Cytogenetics* 2017, **10(Suppl 1):**4.P14

Sex determining mechanisms in reptiles are in general remarkably variable. The sex is determined by the temperature or different external factor in species with environmental sex determination and males and females share the same genotype. On the other hand, under genotypic sex determination, males and females differ in genotype and sex is determined by the combination of sex chromosomes. There are lineages with male as well as lineages with female heterogamety among reptiles, with highly differentiated as well as almost undifferentiated sex chromosomes. In this contribution, I will present latest data showing that some groups have evolutionary very stable sex chromosomes (iguanids, lacertids, advanced snakes) while some others possess great variability in sex determining systems (geckos and acrodont lizards). We will present new information about sex chromosomes in several important species and lineages in evolutionary context, including the discovery of previously unknown sex chromosomes. Also, I will introduce a rare case of dedifferentiation of sex chromosomes in one group of geckos and discuss the possible mechanisms behind it. This contribution summarises new trends and findings regarding the evolution of sex chromosomes in a very important and interesting group of vertebrates and suggests new views on some aspects of sex chromosome evolution in general.

### 4.P15

### The importance of having iguanas Great contribution of iguanid lizards to cytogenetics and genomics

Marie Altmanová, Martina Johnson Pokorná, Michail Rovatsos, Lukáš Kratochvíl
Faculty of Science, Charles University, Department of Ecology, Prague, Czech Republic
**Correspondence:** Marie Altmanová
*Molecular Cytogenetics* 2017, **10(Suppl 1):**4.P15

Iguanas (Squamata: Pleurodonta) form one of the most diverse groups of squamate reptiles. The group includes almost all imaginable ecomorphs – species from deserts as well from rainforests, from high mountains or living on the seashore, climbing on rocks, trees or even diving in the sea. The genus Anolis with over 400 species is well-known as a model of adaptive radiation on Caribbean islands. One of the species of this genus, Anolis carolinensis, is also the first squamate reptile with a fully sequenced and well-annotated genome. Despite all these facts, the iguanas are still quite poorly studied cytogenetically. From the 60's and 70's there have been studies reporting karyotype formulas for many species; however up to now, the more detailed knowledge about genome organization and sex determination was scarce as in other groups of reptiles due to the lack of advanced molecular-cytogenetic, genetic and genomic methods. In our contribution we report current findings about karyotype evolution. We present the reconstruction of the ancestral karyotype and evaluate the rate of chromosomal rearrangements among iguana families. Also, we describe sex chromosomes and although they differ between lineages, we prove they have a common origin in the whole group. We also review evidence on (non)homology of iguana sex chromosomes with those of other amniotes. Altogether, we offer an overview of investigations about iguana cytogenetics highlighting their synapomorphies and contrastingly, the plesiomorphic characters they share with other reptiles to enlarge our knowledge and understanding of evolutionary biology, in particular the evolution of sex determination and genome organization.

### 4.P16

### Genetic analysis of canine mast cell tumors

Jan Frohlich, Halina Cernohorska, Miluse Vozdova, Svatava Kubickova, Jiri Rubes
Central European Institute of Technology - Veterinary Research Institute, Genetics And Reproduction, Brno, Czech Republic
**Correspondence:** Jan Frohlich
*Molecular Cytogenetics* 2017, **10(Suppl 1):**4.P16

Dogs (Canis familiaris) have been our companions for millennia. As our pets, they share our environment and develop similar diseases. Cancers cause as much morbidity and mortality in dogs, as they do in humans and are leading cause of death in dogs over the age of 10. One of the most frequent skin neoplasias in dogs are mast cell tumors (MCTs) which account for up to 21% of all canine skin tumors. Despite the importance of MCTs in veterinary medicine, little is known about the genetic background of this disease.
To find associations between cytogenetic abnormalities, gene mutational status, histological grade and clinical outcome, we performed the molecular analysis of c-kit gene and cytogenetic analysis by FISH using a panel of whole chromosome painting probes in fresh and FFPE samples. However, the mutations in c-kit gene were found only in 15.9% of all our examined MCTs, and FFPE samples could not be analyzed by FISH. Therefore, to shed light on the other genetic cause of this disease we used the whole exome sequencing (WES) to search for molecular aberrations in the coding part of the genome. Paired sample approach was used when tumor tissue and blood from the same animal were subjected to WES for detection of causal mutations and for elimination of non-causative genetic variants.
Identification of new MCT-associated genes will improve our understanding of the genetic background of canine MCT, provide data for accurate diagnosis and prognosis and offer a perspective of novel targeted treatment strategies.

### 4.P17

### A Cross Species Bioinformatics and FISH approach to physical mapping of Mammalian Genomes

Rebecca Jennings [1], Rebecca E O'connor [1], Marta Farré [2], Joana Damas [2], Laura Buggioti [2], Lucas Kiazim [1], Gothami Fonseka [3], Denis M Larkin [2], Darren K Griffin [1]
[1]University of Kent, School of Biosciences, Canterbury, UK; [2]Royal Veterinary College, Department of Comparative Biomedical Sciences, London, UK; [3]Cytocell Ltd, Cytocell Ltd, Cambridge, UK
**Correspondence:** Rebecca Jennings
*Molecular Cytogenetics* 2017, **10(Suppl 1):**4.P17

An essential pre-requisite to whole genome analysis is an unbroken length of contiguous DNA sequence along the length of each chromosome. Most de novo sequenced assemblies are however, highly fragmented. This fragmentation restricts their use in studies of gene mapping, trait linkage, phylogenomics and genomic organisation. In order to overcome these limitations, we recently presented a novel scaffold-to-chromosome anchoring method combining Reference-Assisted Chromosome Assembly (RACA) and fluorescence in situ hybridisation (FISH) to map the scaffolds of de novo sequenced avian genomes (Damas et al. 2016).

In order to test this method in non-avian species, we present preliminary work on a set of universal FISH probes developed using BAC clones isolated from evolutionarily conserved sequences from the cattle (Bos taurus) genome. Using the cow as the reference genome, a selection of BACs were labelled and tested on multiple mammalian species using FISH in order to refine our selection criteria, with the ultimate goal of mapping scaffolds using these probes.
Successful hybridisations were achieved on the chromosomes of all species attempted including (among others) human, pig (Sus scrofa), sheep (Ovis aries), chevrotain (Tragulus javonica). These results provide preliminary evidence that our combined FISH and bioinformatics approach developed for avian species can also be applied to the mapping of genome assemblies in other orders, allowing comparative genomics research at a higher resolution than previously possible.

### 4.P18

### Microchromosomal genome conservation revealed across multiple avian species through cross species BAC mapping

Rebecca E. O'connor [1], Joana Damas [2], Gothami Fonseka [3], Lucas Kiazim [1], Rebecca Jennings [1], Sophie Bennett [1], Sunitha Joseph [1], Denis M. Larkin [2], Darren K. Griffin [1]
[1]University of Kent, School of Biosciences, Canterbury, UK; [2]Royal Veterinary College, Department of Comparative Biomedical Sciences, London, UK; [3]Ltd, Cytocell, Cambridge, UK
**Correspondence:** Rebecca E. O'connor
*Molecular Cytogenetics* 2017, **10(Suppl 1):**4.P18

An extraordinary degree of genome conservation has previously been illustrated in the macrochromosomes of distantly related birds through chromosome painting, however the application of microchromosomal paints in this context, has been limited (due to technical difficulties associated with flow sorting of microchromosomes). To overcome these limitations, and to assess whether the degree of genome conservation identified in the macrochromosomes extends through avian genomes to the microchromosomes, we developed a cross-species BAC based approach using clones isolated from evolutionarily conserved sequences from the chicken and zebra finch genomes.
Using the chicken (Gallus gallus) genome as the reference, two distally located BACs per microchromosome were labelled and tested on over 25 avian species using fluorescence in situ hybridisation (FISH). Results were achieved successfully on all species attempted including (among others) Japanese Quail (Coturnix japonica), Blackbird (Turdus merula), Budgerigar (Melopsittacus undulatus), Pharoah Eagle Owl (Bubo ascalaphus) and Peregrine Falcon (Falco peregrinus). Interchromosomal rearrangements involving the microchromosomes were restricted to only two orders - the Falconiformes and Psittaciformes, while in all other species tested, the microchromosomes remained intact as discrete individual units. Further testing of these probes on non-avian reptile species (turtles and anole lizard) revealed a level of microchromosome conservation extending far beyond avian species.
Our study provides new insight into the nature of avian genomes and genomic stability, revealing an extraordinary degree of previously undetected genome conservation throughout the avian phylogenetic class and beyond.

### 4.P19

### Cytogenetic studies of the genus Raphicerus (Bovidae)

Terence J. Robinson [1], Halina Cernohorska [2], Svatava Kubickova [2], Jan Frohlich [2], Petra Musilova [2], Jiri Rubes [2]
[1]University of Stellenbosch, Department of Botany And Zoology, Stellenbosch, South Africa; [2]Veterinary Research Institute, Department of Genetics And Reproduction, Brno, Czech Republic
**Correspondence:** Halina Cernohorska
*Molecular Cytogenetics* 2017, **10(Suppl 1):**4.P19

Raphicerus (Antilopini, Bovidae) is a group of small-sized African antelope comprising three species, R. sharpei, R. campestris and R. melanotis. In spite of marked phenotypic differences, they are noteworthy for possessing identical karyotypes (2n = 30) based on FISH and conventional banding analyses. Identical Robertsonian translocations predominate in shaping of their autosomal genomes. Unlike autosomes, however, the morphology of the X chromosomes differ among species. The differences in morphology are largely attributable to the amplification of large heterochromatin blocks that, importantly, comprise different repetitive DNA sequences. The DNA blocks were isolated by laser microdissection, cloned, sequenced and the data examined from a phylogenetic perspective. We reflect on the accumulation of the repeats in the different positions of the X chromosomes, and postulate how these could affect the species' evolution.

### 4.P20

### Multiple hybridization devices for subtelomeric regions of pigs and cattle reveal hitherto undetected translocations

Rebecca E. O'Connor [1], Gothami Fonseka [2], Lucas G. Kiazim [1], Rebecca L. Jennings [1], Richard Frodsham [2], Alan Archibald [3], Martin Lawrie [2], Grant A. Walling [4], Darren K. Griffin [1]
[1]University of Kent, School of Biosciences, Canterbury, UK; [2]Cytocell, Ltd, Cambridge, UK; [3]University of Edinburgh, The Roslin Institute, Midlothian, UK; [4]Jsr, Genetics, North Humberside, UK
**Correspondence:** Lucas G. Kiazim
*Molecular Cytogenetics* 2017, **10(Suppl 1):**4.P20

Reciprocal chromosomal translocations have been shown to affect fertility in most agricultural species, leading to reduced litter sizes in pigs and lower birth rates in cattle. With an increasing emphasis in modern farming on the use of a small population of males for artificial insemination, the potential economic and environmental costs of using hypoprolific boars and bulls impact on global food production strategies. Traditionally, translocation screening has been performed using standard karyotype analysis, however this approach relies on a significant level of expertise and is limited in its ability to identify cryptic translocations. In cattle, the complex karyotype (2n = 60) usually restricts analysis to Robertsonian translocations alone. To address this problem in pigs and cattle, we developed novel screening tools using subtelomeric probes and multi-target fluorescence in situ hybridisation strageies based on Cytocell MultiProbe devices.
Probes were designed using BACs from the most subtelomeric region of each chromosome in pigs and cattle and were directly labelled with FITC or Texas Red. Probes were subsequently applied to a 'Multiprobe' device, enabling simultaneous detection of each chromosome on a single slide, therefore significantly shortening analysis time. Initial experiments led to the discovery of a series of incorrectly mapped regions in the porcine genome assembly, highlighting the importance of accurate physical mapping of newly sequenced genomes. After screening of around 400 samples, multiple porcine translocations have been identified using this approach, including previously cryptic translocations undetectable at the karyotype level. The cattle device is still in the developmental stage but has the potential to detect previously untractable reciprocal translocatins.

### 4.P21

### Novel approaches for the physical, chromosomal mapping of avian genomes doubles the number of assemblies

Rebecca O'Connor [1], Damas Joana [2], Farre Marta [2], Martell Henry [1], Kiazim Lucas [1], Rebecca Jennings [1], Anjali Mandawala [3], Sunitha Joseph [1], Slack Eden [2], Allanson Emily [2], Fowler Katie [3], Denis Larkin [2], Darren Griffin [1]
[1]University of Kent, Biosciences, Canterbury, UK; [2]Royal Veterinary College, Comparative Biomedical Sciences, London, UK; [3]Canterbury Christ Church University, Human And Life Sciences, Canterbury, UK
**Correspondence:** Darren Griffin
*Molecular Cytogenetics* 2017, **10(Suppl 1):**4.P21

The ultimate aim of a genome assembly is to create a contiguous length of sequence from p- to q- terminus of each chromosome. Most assemblies are however, somewhat fragmented, and this impedes their use in investigations involving e.g. genomic organization and mapping, trait linkage and phylogenomics. In order to overcome these limitations, we present a novel scaffold-to-chromosome anchoring method combining Reference-Assisted Chromosome Assembly (RACA) and fluorescence *in* situ hybridisation (FISH) to position scaffolds from assemblies with N50 > 1-2 Mb on chromosomes.
Recnetly we presented an approach whrre scaffolds generated from *de novo* sequenced genomes were ordered and orientated using RACA against a reference and outgroup genome into 'predicted chromosome fragments' (PCFs). PCFs were verified using PCR prior to mapping with FISH. A universal set of FISH probes has now been developed through selection of conserved regions were then previously used to map PCFs of the peregrine falcon (*Falco peregrinus*) and rock pigeon (*Columba livia*) genomes. We previously reported we were able to physically map 87% of the peregrine and 84% of the pigeon genome, improving the N50 of both seven-fold, as well as identify a series of intra and interchromosomal rearrangements. Here we report the mapping of three additional genomes mapped using this method and more sophisticated multi-plex FISH strategies, namely the ostrich (*Struthio camelus*), the saker falcon (*Falco cherrug*) and the budgerigar (*Melopsittacus undulatus*). This illustrates the universal application of this method to avian genomes and doubles the number of chromosomally mapped genomes.
The approach presented here, that involves a combination of zoo-FISH and bioinformatics approach permits comparative genomic research at a higher resolution than previously described and opens up, new avenues of investigation into genome karyotype evolution and the role of Evolutionary Breakpoint Regions (EBRs) and Homologous Synteny Blocks (HSBs).

### 4.P22

### First cytogenetical characteristics of Cobitis simplicispina Hanko 1925 (Teleostei Cobitidae)

Sevgi Unal [1], Muradiye Karasu Ayata [2], Muhammet Gaffaroglu[3]
[1]Gazi University, Biology, Ankara, Turkey; [2]Ahi Evran University, Biology, Kirsehir, Turkey; [3]Ahi Evran University, Molecular Biology And Genetics, Kirsehir, Turkey
**Correspondence:** Sevgi Unal
*Molecular Cytogenetics* 2017, **10(Suppl 1):**4.P22

The aim of the present study is investigate cytogenetical properties, constitutive heterochromatin (CH) and nucleolar organizer regions (NORs) of the endemic Turkish loach species Cobitis simplicispina Hanko, 1925 (Teleostei, Cobitidae) from Küfi Creek, Denizli, Turkey. Specimens were transported alive to the laboratory. Metaphase chromosomes were prepared from kidney cells according to the "air drying technique". From each specimen, at least 10 chromosome slides were prepared. C-banding technique and Ag-NOR staining were performed to the slides. Chromosomes were investigated with a Leica DM 3000 microscope and photographs of good metaphases were taken with AKAS software. For determining diploid chromosome number 100 metaphase plaques were analyzed. The diploid chromosome number of C. simplicispina was found as 2n = 50. The karyotype was consisted of eight pairs of metacentric, eight pairs of submetacentric and nine pairs of subtelo-acrocentric chromosomes. Fundamental arm number was calculated as 82. CHs were identified on the centromeric region of most chromosomes. Totally 64 silver nitrate stained metaphases were counted. 90.63% frequently of these metaphases have two NORs on the largest metasentric chromosomes and 9.37% frequently metaphases have four NORs. In conclusion the obtained results present the first cytogenetic features of C. simplicispina.

### 4.P23

### Chromosomal analysis of Anatolian loach Cobitis phrygica Battalgazi 1944 (Teleostei Cobitidae)

Muradiye Karasu Ayata [1], Sevgi Unal [2], Muhammet Gaffaroglu [3]
[1]Ahi Evran University, Biology, Kirsehir, Turkey; [2]Gazi University, Biology, Ankara, Turkey; [3]Ahi Evran University, Molecular Biology And Genetics, Kirsehir, Turkey
**Correspondence:** Muhammet Gaffaroglu
*Molecular Cytogenetics* 2017, **10(Suppl 1):**4.P23

A chromosomal study of endemic loach Cobitis phrygica Battalgazi, 1944 was conducted by means of Giemsa staining, C-banding and silver staining procedures. The specimens were collected from Salda Lake, Burdur, Turkey. They were transported alive to the laboratory and kept in well-aerated aquaria until analysis. Chromosome preparations were obtained from the head kidney by using the "air drying technique". On the average of 12 slides were prepared from each specimen. Also, C-banding technique was used for determining constitutive heterochromatin regions and silver staining was used for determining nucleolar organizer regions (NORs). All preparations were scanned with a Leica DM 3000 microscope. Photographs of good metaphase plaques were taken with AKAS software. At least 100 metaphase plaques were counted to determine the diploid chromosome number (2n). 2n of C. phrygica was determined as 50 and karyotype was consisted of four pairs of metacentric, four pairs of submetacentric and 17 pairs of subtelo-acrocentric chromosomes. Fundamental arm number was calculated as 66. Heteromorphic sex chromosomes were not detected. Constitutive heterochromatin regions were found on the centromeres of almost all chromosomes. NORs were observed on the terminal regions of one pair of metasentric chromosomes. This study may contribute to cytogenetics of Anatolian Cobitis species.
This work was supported by the Ahi Evran University Scientific Research Projects Coordination Unit. Project Number:FEF.E2.17.019

### 4.P24

### Karyotypic changes in response to heat shock

Tatiana Grinchuk [1], Mariia Shilina [1], Nikolay Nikolsky [2]
[1]Institute of Cytology of The Russian Academy of Science, Group of Genetic Mechanisms of Cell Differentiation And Malignization, St-Petersburg, Russia; [2]Institute of Cytology of The Russian Academy of Science, Department of Intracellular Signalling And Transport, St-Petersburg, Russia
**Correspondence:** Tatiana Grinchuk
*Molecular Cytogenetics* 2017, **10(Suppl 1):**4.P24

Previously, we showed that long-term cultivation of Chinese hamster fibroblasts CHLV-79 RJK at elevated temperature resulted in the selection of a thermoresistant line with genetic changes at the level of karyotype. These cells had chromosomes with homogeneously staining regions (HSRs) and new markers resulted from chromosome breaks near centromeres.
In this work we studied the karyotype of CHLV-79 RJK progeny survived after heat shock (HS) (40 ° C, 1 hour). Cells were karyotyped by G- banding on the 4th passage after HS. These cells have high level of karyotypic instability. About 90% of metaphase plates were defective. These abnormalities were manifested mostly as variable chromosome number and breakages of chromosomes. Damage on the chromatid level, involvement one or two sister chromatids, were revealed in some metaphase plates. The chromosomes 2, Z1, Z3 and Z4 were involved in breakages more frequently than other chromosomes.
Interestingly, chromosome 2, Z3 and Z4 were involved in the formation of marker chromosomes in the thermoresistant line. The karyotype in control cells was stable – about 95% population.
In conclusion, changes in the structure of CHLV-79 RJK karyotype in response to HS are genetically programmed and important tool for adaptive changes in the genetic structure. The work was supported by Russian Science Foundation (project 14-50-00068).

### 4.P25

### Interspersed telomeric sequences (TTAGGG)n distribution in Primates and Tupaia minor (Scandentia)

Sofia Mazzoleni [1], Odessa Schillaci [2], Sara Vlah [2], Francesca Dumas [2]
[1]Prague University, Department of Ecology, Faculty of Science, Prague, Czech Republic; [2]Palermo University, Department of Scienze E Tecnologie Biologiche Chimiche E Farmaceutiche, Palermo, Italy
**Correspondence:** Francesca Dumas
*Molecular Cytogenetics* 2017, **10(Suppl 1):**4.P25

Over recent years a growing number of studies suggest that the (TTAGGG)n repeats, characterizing telomeres of Vertebrates, can be detected not just at the traditional position of chromosomal ends, but at interspersed sites as well. Cytogenetic and molecular studies indicate these interspersed sequences, known as Interstitial Telomeric Sequences (ITSs), as important elements affecting genome plasticity and evolution. Even if ITSs role is still not fully understood they have been associated to (1) chromosomal rearrangements; (2) mechanism of genome reorganization, such as double DNA strand break repair, involving retrotransposons; (3) process of recombination and amplification of the terminal end of chromosomes; (4) mechanism of centromere/telomere interchanges. In this context, to elucidate ITSs role and distribution, we mapped through Fluorescence In Situ Hybridization (FISH) the telomeric PNA (TTAGGG)n probe on 16 species representative of each major primates groups and Tupaia minor (Scandentia). Our PNA mapping results, as expected, showed hybridization signals at telomeric ends of chromosomes in all samples and consistently at several centromerical and interstitial position in many analyzed species. The distribution of ITS in primates species have been analyzed in a phylogenetic perspective using Tupaia minor as outgroup. In particular in Tupaia the probe hybridization revealed many bright ITSs on at least eleven chromosome pairs, both bi-armed and acrocentric; our comparative analysis shows probe signals, both in correspondence to fusion points of human syntenic associations, and on other chromosomes uniformly composed of a single human synteny. These data compared with that of literature in Primates let us to support the hypothesis about a correlation between ITSs and rearrangements, thus indicating their possible role in genome organization and evolution.

### 4.P26

### The Japanese quaıl genome a cytogenetıc revısıon

Maria Kulak [1], Svetlana Galkina [2], Alsu Saifitdinova [3], Denis Larkin [4], Joana Damas [4], Marta Farre [4], Darren Griffin [5], Rebecca O'connor [5], Alexey Komissarov [6], Elena Gaginskaya [1]
[1]Saint Petersburg State University, Department of Cytology And Histology, Saint Petersburg, Russia; [2]Saint Petersburg State University, Department of Genetics And Biotechnology, Saint Petersburg, Russia; [3]International Centre of Reproductive Medicine, Laboratory of Assisted Reproductive Technologies, Saint Petersburg, Russia; [4]Royal Veterinary College, University of London, Department of Comparative Biomedical Sciences, London, England; [5]University of Kent, School of Biosciences, Kent, England; [6]Saint Petersburg State University, Theodosius Dobzhansky Center For Genome Bioinformatics, Saint Petersburg, Russia
**Correspondence:** Maria Kulak
*Molecular Cytogenetics* 2017, **10(Suppl 1):**4.P26

Low quality of the existing quail genome assembly is an important limiting factor for using the Japanese quail as a developmental biology animal model and applying genomic selection to quail breeding. Japanese quail Coturnix coturnix japonica has a relatively small genome (≈1.41 Gb) packed into 39 chromosome pairs. Its karyotype is very similar to the chicken one. It is accepted that quail and chicken chromosomes are orthological, a few intrachromosomal rearrangements were described. The microchromosome morphology is different in these two species. Quail microchromosomes are mainly submetacentric having heterochromatic short arms while in chicken they are acrocentric. In 2016, the quail genome assembly was produced on the chicken genome assembly galGal3 as a reference. Currently, it includes 32 (of 38 + Z and W) linkage groups. However, the quail assembly contains a number of sequence gaps because of repetitive coding and non-coding DNA elements. In this work, we have identified new highly repeated tandem sequences within unassembled quail short raw reads and mapped them to Japanese quail genome. To verify the quail draft genome assembly we performed systematic ZOO-FISH experiments on Japanese quail chromosomes with chicken BAC clone probes from CHORI-261 chicken BAC library. BAC clones were selected according to their chromosomal positions and assigned for specific sequence markers and high cross-species hybridization efficiency. All BACs used were positioned in the quail genome assembly. In some cases we have found discrepancies between positions of the markers in the quail genome assembly and physical maps, caused by the newly identified interspecific rearrangements.
Support: "Chromas" Research Resource Center (Saint-Petersburg State University), RFBR #16-04-01823.

### 4.P27

### Evaluation of chromosome microrearrangements of a horse applying array CGH

Katarzyna Kowalska [1], Klaudia Pawlina [1], Wojciech Witarski [2], Magdalena Wojtaszek [1], Monika Bugno-Poniewierska [1]
[1]National Research Institute of Animal Production, Genomics And Animal Molecular Biology, Cracow, Poland; [2]National Research Institute of Animal Productionnational Research Institute of Animal Production, Genomics And Animal Molecular Biology, Cracow, Poland
**Correspondence:** Katarzyna Kowalska
*Molecular Cytogenetics* 2017, **10(Suppl 1):**4.P27

A selection of research techniques and quality of obtained metaphase plates plays a crucial role in diagnostic cytogenetics. The dynamic development of high-resolution cytogenetic techniques such as array CGH allows increasing the accuracy of diagnosis of chromosomal disorders and the early elimination of breeding individuals carrying aberrations. Therefore, the aim of the study is a broad range analysis of chromosome abnormalities, including unbalanced microrearrangements, with the use of a highly precise technique of array comparative genomic hybridization (aCGH), in individuals affected by reproductive and/or development disorders.
One of the examined animals was 19 -year old mare, which has overall female body conformation and oestrus, however, has not had a foal. A karyotype analysis showed that of 333 metaphases,7 had the normal karyotype 64, XX while 326 had 65, XXX. In the next step, we carried out DNA labeling using SureTag DNA Labeling Kit (Agilent). The labelled DNA was then subjected to 40 h hybridization onto 2x400K custom equine CGH microarrays (Agilent). The washed microarrays were scanned with SureScan G2565CA Scanner (Agilent). The obtained raw data were analysed using Agilent Genomic Workbench (7.0).
The comprehensive analysis using aCGH technique revealed 426 aberrations, including 377 amplifications and 49 deletions, in the genome of the investigated horse. As many as 3 deletions and 374 amplifications were identified on chromosome X. The largest number of aberrations was detected on ECA1 and 12 and they mainly embraced genes connected with MHC II - DRB and DQA. The obtained results allowed characterising genes and pathways which are altered in the genome of the investigated mare.
Financed: BIOSTRATEG2/297267/14/NCBR/2016.

### 4.P28

### Screening of the Polish young horse population for detection of sex chromosomes abnormalities preliminary results

Monika Bugno-Poniewierska, Magda Wojtaszek, Katarzyna Kowalska, Wojciech Witarski
National Research Institute of Animal Production, Genomics And Animal Molecular Biology, Cracow, Poland
**Correspondence:** Monika Bugno-Poniewierska
*Molecular Cytogenetics* 2017, **10(Suppl 1):**4.P28

Abnormalities of chromosome number or morphology often lead to embryonic or perinatal death, subfertility or infertility in horses. Aberrations can be either inherited from parents or can appear de novo. In the case of mares, a long lasting adolescent period causes their withdrawal from the breeding program after a second non-pregnant season, which leads to significant economic losses associated with maintaining mares with breeding difficulties for an average of 5 years. Concerning measurable economic losses caused by the carrier state of chromosomes disorders, cytogenetic diagnostics are of great importance for animal husbandry.
Research conducted in 2003–2007 showed that in a population of 500 young horses, sex chromosome aberrations are at the level of about 2% while concerning aberrations occurring only in mares value rises to 3.7%. The cytogenetic investigation was conducted based on classic staining methods (CBG, GTG).
The development of molecular methods increases the accuracy of diagnosis. In this study, we validate the results of cytogenetic examination using the fluorescence in situ hybridization (FISH) technique with molecular probes specific to the sex chromosomes, examined on 500 young horses (up to 2 years) that belong five breeds.
Until now, we collected blood samples from 244 animals including 93 Hucul, 88 Arabian, 20 Sztumski and Sokolski draft horses, 13 Polish Konik and 10 Malopolska breeds; from which lymphocyte cultures were established and cultivated. Thirty animals were karyotyped by molecular cytogenetic analyses – FISH. Each karyotype analysis consisted of the examination of 300 metaphase plates which precludes any aberration in the form of a mosaic at the 1% level. The preliminary karyotyping results of 30 horses show normal karyotype in all investigated animals: 64, XX in mares and 64, XY in stallions. This study is to be continued to enlarge to the maximum examined the population of horses in Poland.
Financed: BIOSTRATEG2/297267/14/NCBR/2016.

### 4.P29

**Leveraging molecular cytogenetics in biopharm cell line development**

Remben Talaban, Shahla Salehi, Shawal Spencer, Daniele Perna, Gary Finka, Mark Uden
Glaxosmithkline, Biopharm Process Research, Stevenage, UK
**Correspondence:** Remben Talaban
*Molecular Cytogenetics* 2017, **10(Suppl 1):**4.P29

Chinese hamster ovary (CHO) cells are a widely used mammalian expression platform in the industrial production of therapeutic recombinant proteins. The choice of CHO cells as the biopharmaceutical industry's workhorse is largely due to its high adaptability to various culture conditions, ease of genetic manipulation, ability to produce vast amount of recombinant products and proven safety record. Cell line development (CLD) for expression of biopharm assets is a lengthy process with significant time and resource dedicated to the identification of top performing lines from a myriad of less suitable phenotypes. Efforts are underway to improve cell line performance and streamline the identification of top performers through rational design of our biopharm platform. Here, we describe the use of fluorescence in situ hybridisation (FISH) to compare and contrast two in-house targeted integration approaches to CHO CLD.
Firstly, we have developed a Rapid-FISH screen which has been successfully embedded as an integral step of the CLD process for cell lines generated using our in-house Chromos Artificial Chromosome (AC) Expression technology. The application of Rapid-FISH at early stage CLD allows us to rapidly screen clones and determine those with high level, on-target AC integration for subsequent progression, ensuring Chromos technology is dictating cell line performance. Secondly, high-sensitivity FISH has been used to confirm successful targeting of genes of interest into pre-defined CHO genomic loci using genome engineering and expression cassette flipping technologies; indeed we have successfully confirmed both mono- and bi-allelic genome targeting and have established integration profiles for a number of our CHO cell lines using FISH.
The use of molecular cytogenetics has afforded us significant comprehension of various cytogenetic phenomena underpinning the performance of our biopharm cell lines and continues to play an important role in the ever-changing CLD landscape.

### 4.P30

**Discrimination of the C genome and two closely related forms of the A genome in endosperm of an Avena magna x A. longiglumis amphiploid**

Paulina Tomaszewska [1], Romuald Kosina [2]
[1]Institute of Experimental Biology, University of Wroclaw, Department of Plant Developmental Biology, Wroclaw, Poland; [2]Institute of Environmental Biology, Plant Speciation Group, Wroclaw, Poland
**Correspondence:** Paulina Tomaszewska
*Molecular Cytogenetics* 2017, **10(Suppl 1):**4.P30

The free nuclear endosperm of an Avena magna x A. longiglumis amphiploid (2n = 6x = 42) and their parental species was investigated by the classical and molecular cytogenetic methods. The number of chromosomes in endosperm was established. Endosperms of oat amhiploid and species are the mosaics of cells with different ploidy levels. The expected triploid number of chromosomes in endosperm was observed sporadically. The genomic compositions of endosperm nuclei has been established for Avena magna x A. longiglumis, tetraploid A. magna and diploid A. longiglumis. The in situ hybridization technique with the use of total genomic DNA probes derived from A. nuda and A. eriantha allowed to discriminate the C genome as well as two closely related forms of the A genome in endosperm of an A. magna x A. longiglumis amphiploid. The number of translocations between genomes has been determined. The results show that the gain of genome segments in the endosperm nuclei is genome-specific. Double fluorescence in situ hybridization was used to determine the chromosomal localization of 5S and 18S-5.8S-25S rRNA genes in endosperm. The number of rDNA sites on chromosomes of amphiploid corresponds to the total number of signals in their parental species. The number of signals after combined DAPI + PI staining corresponds to the number of 25S rDNA signals detected by fluorescence in situ hybridization. The maximum number of nucleoli observed in endosperm nuclei reflects the number of 25S rDNA sites on chromosomes.

### Accreditation and Quality Control

### 5.P1

**Quality control CHECK YOUR CULTURES! Karyotyping identifies genetic instability**

Ulrike A. Mau-Holzmann [1], Maria Joana Osorio [2], Niklas Schwarz [3], Benjamin Schmid [4]
[1]University of Tuebingen, Institute of Medical Genetics And Applied Genomics, Tuebingen, Germany; [2]University of Copenhagen, Center for Basic and Translational Neuroscience, Hoersholm, Denmark; [3]University of Tuebingen, Hertie Institute, Tuebingen, Germany; [4]Technological University of Denmark, Bioneer A/s, Hoersholm, Denmark
**Correspondence:** Ulrike A. Mau-Holzmann
*Molecular Cytogenetics* 2017, **10(Suppl 1):**5.P1

Many useful guides and guidelines exist for those working with cell cultures to be aware of contamination with microorganisms, phenotypic and genetic instability or even cell-line misidentification. A lot of these problems are avoidable with the necessary foresight. Though, simple rules are frequently ignored. With depressing regularity retraction or modification of these data is seen.
Among the problems that continue to affect cell culture, genetic instability is an important mosaic stone. Depending on the cell type (senescent or immortal cell lines, human or not human, iPSC, cancer cell line) the chromosomal content can be euploid, aneuploid (abnormal chromosome content) and heteroploid (variable chromosome content within the population). Aneuploidy, heteroploidy or even special structural aberrations can be typical for specific cell lines. The latter can be very useful for authentication. Descendents of normal lines or iPSC lines usually are euploid. Anyway, pluripotent stem cell lines (i.e. iPSCs), which are usually clonal in origin, are well known to be susceptible to developing chromosomal changes. Therefore, they need to be periodically karyotyped.
The primary purpose of this poster is to increase awareness for genetic instability to those new in the field and those engaged in teaching and instruction. Results of conventional karyotyping (which has the advantage to detect balanced structural aberrations compared to array technologies) of different cell types and cell lines (mainly iPSC) will be presented.

### 5.P2

**A New Automated Microwell Assay That Makes FISH Simple Reproducible and Cost Effective**

Eric Crawford [1], Mingya Liu [1], Cynthia Brooks [1], David Wright [2], James Stranchfield [3]
[1]Genetics Associates, Inc, Cytogenetics, Nashville, TN, USA; [2]Art Robbins Instruments, Engineering, Sunnyvale, CA, USA; [3]Scigene, Engineering, Sunnyvale, CA, USA
**Correspondence:** Eric Crawford
*Molecular Cytogenetics* 2017, **10(Suppl 1):**5.P2

FISH assays are among the most widely used diagnostic tests for human cancers. The combination of the high cost of DNA probes used in a labor intensive, complex workflow and shrinking cost reimbursements is causing mounting economic strain on cytogenetics laboratories.
We describe a simple, slide based system for FISH with streamlined workflow that reduces up to 10 fold the probe cost per test and decreases up to 75% hands-on technologist time. The methodology uses an eight-well, microvolume slide for performing up to eight independent FISH assays in batches of up to 12 slides (MicrofISH Assay Slides; Sunnyvale, CA). Automation of the front part of the workflow is carried out using a high-speed liquid dispensing robot that performs both cell dropping and probe addition (Art Robbins Instruments, Sunnyvale, CA) onto the microvolume slides. After application of a single coverslip (rubber cement not required), slides are briefly heated, incubated and automatically washed using a calibratible thermocycler, hybridization incubator and washing robot (CytoBrite System, MicrofISH Oven and Little Dipper Processor; SciGene, Sunnyvale, CA).
The MicrofISH system provides several important advantages over existing methods. Assays can be now be performed using 1ul of sample and probe per assay preserving precious samples and dramatically reducing probe cost per test. Automation of slide dropping, probe application and slide washing allows reduction of technologist time and potential human error. Automated inventory control, movement log, and generation of patient specific scoresheets yields additional quality control points. Eliminating of a coverslip sealant removes a time consuming and tedious step in the workflow and reduces repetitive motion.
We will present the clinical validation of this system along with optimization studies of several steps in the workflow that has provided consistent high quality clinical results on over 20,000 patient samples.

### 5.P3

**Preclinical florescent in situ hybridization (FISH) validation studies**

Aysegul Kuskucu, Sezin Gurkan
Yeditepe University School of Medicine, Medical Genetics, Istanbul, Turkey
**Correspondence:** Aysegul Kuskucu
*Molecular Cytogenetics* 2017, **10(Suppl 1):**5.P3

Despite the widely usage of Fluorescence in situ hybridization (FISH) probes for clinical diagnosis, number of commercially available and FDA approved assays are restricted. Custom designed or home-brewed FISH assays and probes meet these essential requests. Both probes, FDA approved or home-brewed, need validation and/or verification studies before clinical usage and reporting. However the needs of these kind quality control issue, there is no consensus guideline or protocol for these tests. Additionally, FISH assays can be complicated for validation and verification studies. Metaphase FISH analyses are qualitative tests, and the other hand, interphase FISH analyses are semiquantitative tests and therefore validation studies must be designed differently. Generally validation studies are classified as qualitative, semiquantitative, and quantitative. Like analyses methods and assays, validation procedures could vary between laboratories.
In this study we would like to share our interphase and metaphase FISH validation methods which includes assessment of a probe's technical specifications, establishment of its standard operational procedure (SOP), determination of its clinical sensitivity and specificity, development of its cutoff, confirmation of its applicability to a specific clinical setting, testing of samples with or without the abnormalities that the probe is meant to detect, staff training, and report building.

### Molecular Mechanisms of Chromosome Rearrangements

### 6.P1

**Enhancer swapping dual loss and gains of enhancers by balanced translocations and inversions between highly conserved regulatory domains**

Mana M Mehrjouy [1], Mads Bak [1], Darío G. Lupiáñez [2], Verena Heinrich [3], Joris Vermeesch [4], Albert Schinzel [5], Bitten Schönewolf-Greulich [6], Iben Bache [1], Stefan Mundlos [2], Niels Tommerup [1]
[1]University of Copenhagen, Department of Cellular And Molecular Medicine, Copenhagen-Denmark; [2]Max Planck Institute For Molecular Genetics, Rg Development & Disease, Berlin-Germany; [3]Max Planck Institute For Molecular Genetics, Department of Computational Molecular Biology, Berlin-Germany; [4]University of Leuven, Department of Human Genetics, Leuven-Belgium; [5]University of Zurich, Institute of Medical Genetics, Schlieren-Switzerland; [6]Copenhagen University Hospital, Rigshospitalet, Centre For Rett Syndrome, Kennedycentre, Department of Clinical Genetics, Glostrup-Denmark
**Correspondence:** Mana M Mehrjouy
*Molecular Cytogenetics* 2017, **10(Suppl 1):**6.P1

**Introduction**
Balanced chromosomal translocations/inversions (BCR) may cause disease by long range position effects (LRPE) due to rearrangements of regulatory landscapes. One consequence of this may be enhancer adoption, where the ectopic action of an enhancer dysregulates another gene. We have defined >400 Topological Domains (TAD) and TAD clusters enriched for evolutionary conserved non-exonic elements (CNEs), as high-risk candidates for LRPE. These CNE-TADs might also be high-risk regions for enhancer adoption and potentially even more complex regulatory disturbances.
**Methods**
We compared published cases of enhancer adoption with CNE-TADs, and looked for the double involvement of CNE-TADs in published and unpublished affected and healthy carriers of two-way BCRs. We exemplify the deleterious nature of this by a 46,XX,t(2;13)(p25;q32)dn associated with severe intellectual disability and microcephaly, where we performed mate-pair sequencing and 4C-Seq analysis.
**Results**
Ten/eleven cases with proven enhancer adoption involved either one or two CNE-TADs. Furthermore, 13/283 affected BCR carriers (4.6%) had breakpoints involving two CNE-TADs, vs. 0/116 healthy BCR carriers ($p = 0.024$). The t(2;13)-breakpoints were located within the regulatory domains of SOX11 on chr2, and SOX21 on chr13. 4C-seq analysis of t(2;13)-fibroblasts showed ectopic interactions between the SOX11 promoter and the translocated SOX21-domain segment, and between the SOX21 promoter and the translocated SOX11-domain segment.

**Conclusion**

CNE-TADs are high-risk domains for enhancer adoption events, and dual exchanges between CNE-TADs are high-risk mutations. We term this enhancer swapping, due to the simultaneous removal of CNEs and gain of ectopic CNEs in both domains, with potentially unpredictable phenotypic consequences.

### 6.P2

### Centromere destiny in dicentric chromosomes New insights from the evolution of human chromosome 2 ancestral centromeric region

Giorgia Chiatante [1], Giuliana Giannuzzi [2], Francesco Maria Calabrese [3], Evan E. Eichler [4], Ventura Mario [3]
[1]University of Florence, Department of Biology, Florence, Italy; [2]University of Lausanne, Center For Integrative Genomics, Lausanne, Switzerland; [3]University of Bari 'aldo Moro', Department of Biology, Bari, Italy; [4]University of Washington, Department of Genome Sciences, Seattle, WA, USA
**Correspondence:** Giorgia Chiatante
*Molecular Cytogenetics* 2017, **10(Suppl 1):**6.P2

Dicentric chromosomes are products of genomic rearrangements that place two centromeres on the same chromosome. Due to the presence of two centromeres, dicentric chromosomes are inherently unstable. Instability is overcome by epigenetically inactivating and/or deleting one of the two centromeres resulting in functionally monocentric chromosomes that then segregate normally during cell division. Our current understanding of dicentric chromosome formation, behavior and fate was largely inferred from observational studies in plants and humans. Other clues come from artificially de novo dicentrics produced in yeast and human cells. We investigated the most recent product of a chromosome fusion event fixed in the human lineage, human chromosome 2 which produced a difference in chromosome number between humans (2n = 46) and great apes (2n = 48). Chromosome 2 stability was insured by the suppression of one centromere. Using molecular cytogenetics, and comparative sequencing data, we deeply characterize the relicts of the chromosome 2q ancestral centromere and its flanking regions. We developed two possible centromere inactivation hypotheses to explain the evolutionarily stabilization of human chromosome 2 and gained insight into the ancestral organization that can be easily applied to the centromeres of all acrocentric chromosomes. Our analyses traced the evolutionary history of rDNA and satellite III sequences among great apes, suggesting a novel hypothesis for the preferential inactivation of IIq and other human centromeres. Our results strongly favor centromere excision through a one-step process.

### 6.P3

### De novo deletions and duplications in spermatozoa from fathers of children affected by genomic disorders: cis or trans mediated

Laia Vergés, Yaiza Garcia, Òscar Molina, Francesca Vidal, Joan Blanco
Universitat Autònoma de Barcelona Genetics of Male Fertility Group, Unitat de Biologia Cel · lular Bellaterra (Cerdanyola del Vallès), Spain
**Correspondence:** Joan Blanco
*Molecular Cytogenetics* 2017, **10(Suppl 1):**6.P3

Genomic disorders are human diseases caused by chromosomal rearrangements of unstable regions flanked by Low Copy Repeats (LCRs). LCRs act as substrates for Non-Allelic Homologous Recombination (NAHR) leading to structural aberrations. Preliminary data suggested that some fathers of children affected by genomic disorders showed an increased susceptibility to NAHR that could be either restricted to one specific region or generalized to several regions.

The aim of this study was to analyze the incidence of de novo sperm deletions and duplications in fathers of children affected by Williams-Beuren syndrome (WBS) (n = 6; 7q11.23 deletion), Prader-Willi syndrome (PWS) (n = 17; 15q11-q13 deletion) and 22q11.2 deletion syndrome (22qS); (n = 6; 22q11.2 deletion) of proven paternal origin. The final endpoint was to check whether NAHR mechanisms are restricted to the region involved in the syndrome (cis effect), or associated to a general instability of LCR-unstable regions (trans effect).

Semen samples were processed by FISH using a customized combination of probes. All samples were analyzed for the three regions, compiling retrospective and new acquired data. The frequencies of deletions and duplications were individually compared with an internal control dataset.

Significant increases of sperm anomalies were observed in 13 out of 29 fathers. Of them, eight PWS and two 22qS individuals showed increases restricted to the critical region of the corresponding syndrome. In one of the PWS fathers, anomalies affected the 15q11-q13 and 7q11.2 regions, while in one PWS and one WBS individuals the three analyzed regions were affected.

Recurrent NAHR could be mediated by cis and trans effects, thus suggesting differences in the mechanism that originate anomalies during spermatogenesis. The observation of trans events should be considered of worse prognosis for the recurrence of genomic disorders.

Financial support: CF-180034 (UAB, Spain), SGR2014-524 2 (Generalitat de Catalunya, Spain), SAF2016-77165 (Gobierno de España, Spain)

### 6.P4

### Genomic mobility Fortuitous observation of a private DKC1 retrocopy

Nicolas Chatron [1], Patrick Edery [1], Audrey Labalme [2], Thomas Simonet [3], Pierre-Antoine Rollat-Farnier [2], Claire Bardel [4], Gaetan Lesca [1], Damien Sanlaville [1], Caroline Schluth-Bolard [1]
[1]Hospices Civils De Lyon And Crnl Inserm U1028 Cnrs Umr5292, Ucbl1, Service De Génétique And Gendev Team, Bron, France; [2]Hospices Civils De Lyon, Service De Génétique, Lyon, France; [3]Hospices Civils De Lyon And Institut Neuromyogène Cnrs Umr 5310 - Inserm U1217 - Université Claude Bernard Lyon 1, Centre De Biotechnologie Cellulaire And Nerve-muscle Interactions Team, Lyon, France; [4]Hospices Civils De Lyon And Univ Lyon, Université Lyon 1, Cnrs, Service De Biostatistique And Laboratoire De Biométrie Et Biologie Evolutive Umr5558, Equipe Biotatistique-santé, Lyon, France
**Correspondence:** Nicolas Chatron
*Molecular Cytogenetics* 2017, **10(Suppl 1):**6.P4

Forty five percent of the human genome is composed of retroposed elements consecutive to cis or trans activity of the L1 machinery. Retrocopies of human genes have been mainly studied in an evolutionary context but patient-focused descriptions are very rare. Herein we describe sequencing results for a 450-gene panel in a 10 years old female patient presenting with mild intellectual disability, obesity and dysmorphic features. After sonication and library preparation using SureSelectXT capture (Agilent), 2x75 paired-end sequencing was performed on a NextSeq500 (Illumina). While no clearly pathogenic SNV was identified, depth of coverage analysis using DeCovA revealed a duplication of all 15 exons of DKC1 (Xq28). Careful sequence alignment observation revealed a surprising pattern for paired-end sequencing with a clear shift between exonic depth of sequencing and immediate flanking nucleotides. Moreover, many pairs were encompassing consecutive exons from the 5'UTR region to the 3'UTR region. Subsequent visualization of soft-clipped sequences evoked the presence of a processed retrotranscribed inverted copy of DKC1 inserted within the second intron of IQCH on the long arm of chromosome 15. Fragment junction and cDNA amplifications confirmed these findings and showed paternal inheritance. Inheritance from an unaffected parent added to testis-restricted IQCH expression profile and absence of in silico gene fusion makes this fortuitous observation of an inserted processed transcript likely benign. However, this DKC1 aberrant copy is absent from public databases (Retrofinder UCSC) and doesn't harbour any single nucleotide variation compared to X-copies of DKC1 suggesting a relatively recent evolutionary event. These retrocopies of human genes will likely be more frequently observed with the spread of whole genome sequencing motivating data analysis with a cytogenetic point of view, which provides careful attention to potential mechanisms leading to pathogenicity including aberrant expression, gene disruption, gain of function, gene fusion and siRNA.

### Next Generation Sequencing in Cytogenetics

### 7.P1

### NGS mapped breakpoints in balanced chromosomal rearrangements including the first large cohort of healthy carriers

Niels Tommerup [1], Ana Carolina Fonseca [2], Mana Mehrjouy [1], Malene B Rasmussen [1], Iben Bache [1], Christina Halgren [1], Peter Kroisel [3], Susanna Midyan [4], Joris Vermeesch [5], Angela M Vianna-Morgante [2], Kikue T Abe [6], Danilo Moretti-Ferreira [7], Giorgio Paskulin [8], Lyudmila Angelova [9], Evica Rajcan-Separovic [10], Carolina Sismani [11], Zdenek Sedlacek [12], Christina Fagerberg [13], Karen Brøndum-Nielsen [14], Ida Vogel [15], Anders Bojesen [16], Katrin Õunap [17], Laura Roht [17], Teppo Varilo [18], Tiia Luukkonen [18], James Lespinasse [19], Claire Beneteau [20], Vera M Kalscheuer [21], Nadja Ehmke [22], Cornelia Daumer-Haas [23], Eunice G Stefanou [24], Marta Czako [25], György Kosztolányi [25], Frenny Sheth [26], Orsetta Zuffardi [27], Clara Bonaglia [28], Antonio Novelli [29], Madeleine Fannemel [30], Patricia Dias [31], Nadja Kokalj-Vokac [32], Maria A Ramos-Arroyo [33], Laura Rodríguez Martínez [34], Miriam Guitart [35], Albert Schinzel [36], John Engelen [37], Fatma Silan [38], Yassmine Akkari [39], Jacqueline R Batanian [40], Hyung-Goo Kim [41], Constantia Aristidou [11], Cynthia De Almeida [42], Suzanne Lewis [10], Maria Moreno-Ígoa [33], Anna Hovhannisyan [4], Peter Jacky [43], Mads Bak [1], – International Breakpoint Mapping Consortium [1]

[1]University of Copenhagen, Department of Cellular And Molecular Medicine, Copenhagen, Denmark; [2]Universidade De São Paulo, Departamento De Genética E Biologia Evolutiva, São Paulo, Brazil [3]Medizinische Universität Graz, Institut Für Humangenetik, Graz, Austria; [4]Center of Medical Genetics And Primary Health Care, Center of Medical Genetics, Yerevan, Armenia; [5]University of Leuven, Department of Human Genetics, Leuven, Belgium; [6]Rede Sarah De Hospitais De Reabilitação, –, Brasilia, Brazil; [7]São Paulo State University, Department of Genetics, Botucatu, Brazil; [8]Universidade Federal Das Ciências Da Saúde De Porto Alegre, Genética Clínica, Porto Alegre, Brazil; [9]Medical University - Varna, Department of Medical Genetics, Varna, Bulgaria; [10]Children's And Women's Health Centre of Bc, Department of Pathology (cytogenetics), Vancouver, BC, Canada; [11]The Cyprus Institute of Neurology And Genetics, Department of Cytogenetics And Genomics, Nicosia, Cyprus; [12]The Motol University Hospital, Department of Biology And Medical Genetics, Prague, Czech Republic; [13]Odense University Hospital, Klinisk Genetisk Afdeling, Odense, Denmark; [14]Rigshospitalet, Kennedycenter, Glostrup, Denmark; [15]Aarhus University Hospital, Klinisk Genetisk Afdeling, Aarhus, Denmark; [16]Vejle Hospital, Klinisk Genetisk Afdeling, Vejle, Denmark; [17]Tartu University Hospital, Department of Clinical Genetics, United Laboratories, Tartu, Estonia; [18]University Of Helsinki, Haartman Institute, Helsinki, Finland; [19]Centre Hospitalier De Chambéry, Uf De Génétique Chromosomique, Chambéry, France [20]Chu De Nantes, Service De Génétique Médicale, Nantes, France; [21]Max Planck Institute For Molecular Genetics, Research Group Development And Disease, Berlin, Germany; [22]Charité-universitätsmedizin Berlin & Berlin Institute Of Health (bih), Institute Of Medical And Human Genetics, Berlin, Germany; [23]Pränatal-medizin München, –, München, Germany; [24]University General Hospital of Patras, Department Of Pediatrics, Rio, Greece; [25]University Of Pécs, Department of Medical Genetics, Pécs, Hungary; [26]Foundation For Research In Genetics And Endocrinology [frige] Institute, Institute of Human Genetics, Ahmedabad, India; [27]University of Pavia, Department of Molecular Medicine, Pavia, Italy; [28]Irccs Eugenio Medea, laboratory of Cytogenetics, Bosisio Parini, Italy; [29]Irccs Bambino Gesù Children's Hospital, Medical Genetics Department, Roma, Italy; [30]Oslo University Hospital, Department of Medical Genetics, Oslo, Norway; [31]Centro Hospitalar Lisboa Norte, Department of Genetics, Lisboa, Portugal; [32]University Medical Centre Maribor, Laboratory of Medical Genetics, Maribor, Slovenia; [33]Complejo Hospitalario De Navarra, Servicio De Genética, Pamplona, Spain; [34]Abacid-genética, Hospital Universitario Hm Sanchinarro, Genética Y Diagnóstico Molecular, Madrid, Spain; [35]Corporació Sanitària Parc Taulí, Laboratori De Genètica, Sabadell, Spain; [36]University Of Zürich, Institute Of Medical Genetics, Schlieren, Switzerland; [37]Maastricht University Medical Center, Department of Clinical Genetics, Maastricht, The Netherlands; [38]Çanakkale Onsekiz Mart University, Department Of Medical Genetics, Canakkale, Turkey; [39]Legacy Health Systems, Cytogenetics And Molecular Pathology, Portland, OR, USA; [40]Saint Louis University School of Medicine, Molecular Cytogenetics Laboratory, St. Louis, MO, USA; [41]Augusta University, Department Of Neuroscience And Regenerative Medicine, Augusta, GA, USA; [42]Military Hospital -Dnsffaa, Departamento De Genetica, Montevideo, Uruguay; [43]Nw Permanente, Pc, Emeritus, Portland, OR, USA

**Correspondence:** Niels Tommerup
*Molecular Cytogenetics* 2017, **10(Suppl 1):**7.P1

Next-generation sequencing (NGS) has revolutionised the mapping of balanced chromosomal rearrangements (BCRs), associating specific diseases to truncated genes and to regulatory landscapes associated with long range position effects (LRPE). The large majority of the published BCRs are from affected individuals, and very few BCRs from healthy carriers have been mapped by NGS.

We present the first data from The International Breakpoint Mapping Consortium (IBMC), a world-wide effort to NGS-map ultimately thousands of BCRs, including BCRs from healthy carriers. We mate-pair sequenced BCRs from 87 affected and 117 healthy carriers, doubling the number of mapped two-way breakpoints to >800. The proportion of truncated genes were similar in unaffected and affected carriers (44%-40%, respectively), but we observed a significant excess of truncated autosomal dominant (AD) disease genes ($p = 0.0024$) and loss-of-function (LOF)-constrained genes ($p = 0.0016$) in the affected carriers. However, truncation of known AD genes in apparently healthy carriers does occur, e.g. ABCC9, DCC, CACNB4 and TGFB2. Indeed, the breakpoint within ABCC9 orginates from a founder inv(12) with presently >120 known inversion carriers. Strikingly, 23% of the truncated genes in healthy carriers are LOF-constrained, at odds with exome sequencing data.

We also show that ~70% of the known LRPE-associated breakpoints/loci overlap with topological associating domains (TADs) that are highly enriched in evolutionary conserved non-exonic elements (CNEs). As the tip of the iceberg, we define ~400 CNE-TADs with this hallmark, covering ~16% of the genome, as high risk regions for LRPE. Breakpoints truncating CNE-TADs are more frequent among affected versus healthy BCR carriers ($p = 0.003$), and even more so are intergenic breakpoints within CNE-TADs ($p = 0.0015$).

Our study illustrates the importance of NGS-based mapping of BCRs irrespective of phenotype, and the potential of IBMC to confirm known disease genes, identify numerous novel candidate disease genes and dissect potentially hundreds of LRPE-regions. Mapping of healthy BCR carriers reveal a new resource for the study of why apparently normal individuals carry assumed deleterious mutations.

### 7.P2

### CNV detection comparative study of array based methods and NGS

Alexey Sokolov [1], Aleksandra Bliznina [1], Kirill Tsukanov [1], Anna Krasnenko [1], Anna Kim [1], Daria Plakhina [1], Elena Okuneva [1], Dmitrii Borisevich [2], Dmitriy Korostin [1]

[1]Genotek Inc., R&d, Moscow, Russia; [2]Genotek Inc., Lomonosov Moscow State University, R&d, Faculty Of Bioengineering And Bioinformatics, Moscow, Russia

**Correspondence:** Alexey Sokolov
*Molecular Cytogenetics* 2017, **10(Suppl 1):**7.P2

Copy number variation (CNV) is one of the main forms of critical genetic variation. It is well known that CNV might directly or indirectly lead to some diseases, including cancer. During the past decade array-based methods were de-facto standard technologies for detecting CNV. Today Next-generation sequencing (NGS) based methodologies are occurring for identification of CNV.

Here we used 20 samples, for which CNVs were found using Affymetrix CytoScan HD Array (as standard approach) and Illumina sequencing with Agilent SureSelect Focused Exome capture in order to compare standard approach with NGS. For CNV detection we used CNVkit, method based on reads depth approach.

As a result for all 20 samples we detected 605 CNVs by array based method. For detected CNVs we used clinically stringent filtration: CNV should have length at least 25 kb or 50 kb and at least 25 or 50 probes for losses and gains respectively. For NGS data we found 28 CNVs. Then we intersected these CNVs between two sets of data in order to find precision/recall metrics for NGS based method. We found growth of recall metric with growth of CNV length, with recall up to 100% for CNV length > 3mb. The same tendency was observed for CNV divided by origin (losses and gains) and CNV that were detected in on-target regions. For off-target CNV we got only 7% recall for CNV length > 3mb. On the other hand precision is independent from CNV length and best precision metrics (up to 100%) were collected on CNVs that were detected in on-target regions.
We conclude that reads depth approach for CNV calling have low amount of false positive discoveries independent of CNV length. We also demonstrated that NGS data works reproducibly compared with array-based methods on large-scale CNVs.

### 7.P3

### DNA methylation in inherited chromosomal microduplications with incomplete penetrance

Stanislav Vasilyev [1], Nikolay Skryabin [1], Ekaterina Tolmacheva [1], Renata Savchenko [2], Anna Kashevarova [1], Ludmila Nazarenko [3], Elena Belyaeva [1], Veniamin Fishman [4], Igor Lebedev [1]
[1]Research Institute of Medical Genetics, Tnrmc Ras, Laboratory of Cytogenetics, Tomsk, Russia; [2]National Research Tomsk State University, Biological Institute, Tomsk, Russia; [3]Research Institute of Medical Genetics, Tnrmc Ras, Laboratory of Hereditary Pathology, Tomsk, Russia; [4]Institute of Cytology And Genetics, Sb Ras, Laboratory of Developmental Genetics, Novosibirsk, Russia
**Correspondence:** Stanislav Vasilyev
*Molecular Cytogenetics* 2017, **10(Suppl 1):**7.P3

**Introduction**
Chromosomal diseases caused by inherited CNV are frequently characterized by incomplete penetrance, but its mechanisms remain unexplored. Transgenerational epigenetic modifications may be one of such mechanisms. Here, we report about DNA methylation of several gene promoters in a pedigree with inherited microduplications.
**Methods**
We have analyzed 5 probands with intellectual disability using aCGH. The presence of microduplications in the patient, parents and healthy siblings was investigated by real-time PCR. Bisulfite sequencing of CpG-islands within a region of inherited microduplications was performed using targeted NGS.
**Results**
Various microduplications were identified in analyzed probands: 12q24.12-q24.13 (2 probands from different families, both of maternal origin, including ACAD10, ALDH2 and MAPKAPK5 genes), 5q33.1 on maternal chromosome (including AFAP1L1, GRPEL2 and PCYOX1L genes), 17p13.3 of maternal origin (including RPH3AL, VPS53, GEMIN4, GLOD4, RNMTL1 and NXN genes) and 18p11.32 of paternal origin (including SMCHD1, METTL4 and NDC80 genes). The bisulfite NGS of ACAD10, GRPEL2, GEMIN4, SMCHD1 and METTL4 gene promoters revealed no differences in the level of methylation between probands, healthy sibling without microduplication and parents both with and without microduplication.
**Conclusion**
DNA methylation of gene promoters in analyzed inherited chromosomal microduplications is not the cause of their incomplete penetrance.
This study was supported by Russian Science Foundation (grant № 16-15-10229).

### Other Cytogenetics topics

### 8.P1

### Genomic integrity of stem cells

Tamara Borgonovo, Isadora May Vaz, Carmen Lucia Kuniyoshi Rebelatto, Alexandra Cristina Senegaglia, Paulo Roberto Slud Brofman
Pontifícia Universidade Católica Do Paraná- Pucpr, Core Cell Technology, Curitiba, Brazil
**Correspondence:** Tamara Borgonovo
*Molecular Cytogenetics* 2017, **10(Suppl 1):**8.P1

The use of human mesenchymal stromal/stem cells (MSCs) is a promising strategy for cell therapy in injured tissues recovery. However, ex vivo expansion can lead to accumulation of genetic and epigenetic alterations, featuring a genetic instability that may explain, at least in part, their tumorigenic potential. Therefore, the genomic integrity of stem cells (SC) should be monitored carefully for uses in basic research and clinical trials to confirm the absence of genetic instability. Differently from embryonic SC, where there have been several reports of genetic abnormalities and tumor formation in rats, human adult SC, especially MSCs, seem to maintain their genomic stability during cultivation. The genomic integrity of 158 MSCs samples of 47 patients was evaluated using three techniques: G banding karyotyping, fluorescence in situ hybridization (FISH) and chromosomal instability test with Diepoxybutane (DEB). MSCs from different sources were compared: bone marrow, dental and adipose tissue. Chromosome and chromatid gaps, breaks and tetraploidy were observed, which could indicate an intermediate step for tumorigenesis. Moreover, we detected the emergence of clonal chromosome aberrations at cultivation early stages in one patient. They were also compared during the cultivation time: early, intermediate and late stages, where one of the 13 patients showed clonal chromosome aberrations. In the DEB test all samples shows normal index and in the FISH it was observed one patient with TP53 loss in 24% of the cells. The results confirm the importance of chromosome analysis, especially G-banding, once this technique shows chromosomal instability signs and is able to detect both numeric and structural alteration, including balanced rearrangements and mosaicism. In 97.87% of the cases, MSCs maintained normal karyotypes during the early stages of cultivation. However, some cases may have clonal chromosomal aberrations and these samples should not be used in clinical protocols, because we cannot rule out its tumorigenic potential.

### 8.P2

### Histone acetylation and DNA methylation mediate protamine expression and chromatin compactation during sperm cell differentiation in mouse

Liliana Burlibasa [1], Carmen Domnariu [2]
[1]University of Bucharest, Genetics Department, Bucharest, Romania;
[2]Lucian Blaga University, Medicine, Sibiu, Romania
**Correspondence:** Liliana Burlibasa
*Molecular Cytogenetics* 2017, **10(Suppl 1):**8.P2

A peculiar aspect of mammalian spermatogenesis is that the chromatin structure of spermatogenic cells undergoes a dramatic compactation due to histone replacement by the protamines in the final stages of differentiation. This process is correlated with global epigenetic changes during sperm differentiation. In this context we have examined the dynamics of histone H4 acetylation and DNA methylation at the level of both protamine genes (Prm1 and Prm2). Immunohistochemistry technique and ChIP assay were used for deciphering some important features of chromatin compactation in murine spermatogenesis. ChIP assay provided a new insight into specific relationship between histone acetylation and DNA methylation. Using bisulfite mutagenesis techniques we found differences in DNA methylation pattern between spermatogenic cells (e.g. pachytene spermatocyte, round spermatids and spermatozoa). The study of epigenetic profile in spermatogenic cells could help to elucidate the molecular mechanisms of male infertility.

### 8.P3

### Chromosome studıes ın IPSCells generated usıng SeV based reprogrammıng

Giovanna Piovani [1], Giulia Savio [1], Stefania Masneri [2], Cristina Mora [3], Rosaria Ferraro [2], Gaetana Lanzi [2], Patrizia Dell'era [3], Silvia Giliani [2]
[1]Biology and Genetics Division, Department of Molecular and Translational Medicine, University of Brescia, Brescia, Italy; [2]Cellular fate reprogramming unit, Department of Molecular and Translational Medicine, University of Brescia, Brescia, Italy; [3]Institute of Molecular Medicine "Angelo Nocivelli", Department of Molecular and Translational Medicine, University of Brescia, Brescia, Italy
**Correspondence:** Giovanna Piovani
*Molecular Cytogenetics* 2017, **10(Suppl 1):**8.P3

The discovery of induced pluripotent stem cells (IPSC) has created several interesting prospects not only for basic research, but also to medical treatment and pharmaceutical industry and IPSc represent a virtually unlimited source of pluripotent cells. IPSCs have been generated from different somatic cell types commonly using retrovirus- or lentivirus-based delivery systems. However viral integration into the host genome can cause insertional mutagenesis, malignant transformation and genome instability. In our study we used Sendai viral (SeV) reprogramming, actually the most frequently used reprogramming methods, because is efficient, safe and simple to use. To assess genome stability, karyotype analysis has been performed.
We obtained several IPSC clones starting from peripheral blood mononuclear cells (PBMC), reprogrammed by nonintegrative Sendai virus. We investigate human IPScell line derived from control donors and patients with syndromes showing defects in DNA repair mechanism and alteration in cell cycle progression.
In IPSC derived by donors we detected 4 isolated chromosome anomalies. Unexpectedly, considering the intrinsic defects in DNA repair and cell cycle progression of the analyzed diseases, we observed only one clone of AT IPSCs reporting a complex rearrangement with a partial dup1q, not evident in the patient karyotype on blood cells. Our results suggest that episomal SeV-based reprogramming method must be preferred since integrative methods combine low reprogramming efficiency with a higher risk of cytogenetic abnormalities. SeV-based IPSC clones have been shown to carry some isolated chromosomal alterations at early passages, that were probably acquired during reprogramming due to selective pressure in culture conditions. Since genomic aberrations may have detrimental consequences on stem cell application, we have verify if the identified isolated abnormalities have be fixed in IPSC genome by re-testing the clones after additional passages and in long term culture.

### 8.P4

### Destroying a myth LCLs are neither immortal nor karyotypically stable but acquire trisomy 12 as an early recurrent aberration

Marianne Volleth, Martin Zenker, Thomas Liehr
University Hospital, Human Genetics, Magdeburg, Germany
**Correspondence:** Marianne Volleth
*Molecular Cytogenetics* 2017, **10(Suppl 1):**8.P4

To preserve material for future genetic studies, whole blood samples are routinely used to establish lymphoblastoid cell lines (LCLs) by infection of human B cells in vitro by Epstein-Barr-Virus (EBV). The resulting cell cultures can be grown for a long time span. But these cells are neither immortal nor remain genetically unaltered. Only a small percentage of LCLs finally reach a condition which comes close to immortalization, but these cultures have lost karyotypic stability long before.
To study chromosomal changes during the course of long term cultivation, we established 10 LCLs (from overall 8 individuals) with normal karyotype (3), supernumerary small marker chromosomes (sSMCs, 3) or other aberrant karyotypes (4). Chromosome analyses were done in 8 week intervals over a period of at least 1 up to 3 years. The most common acquired aberration was trisomy 12, which emerged as a clonal change in all cell lines within 22 to 64 weeks after culture set up (median 29.5 weeks). Telomeric associations and telomeric fusions were found as indicators for the shortening of telomeres in a small percentage of metaphase plates earliest after 24 weeks. After a cultivation time of 1 year, within a certain cell line the percentage of cells displaying the original karyotype ranged from 0% (4 lines), up to 10% (3 lines) and more than 50% (60% to 96.7%; 3 lines).
Surprisingly, during chromosomal evolution even clones constituting up to 80% of the cells analyzed at a certain time point, can later disappear completely and be replaced by a cell population with another aberration. Therefore, LCLs could be taken rather as "model organisms" studying clonal evolution than for preservation of cases with certain aberrant karyotypes.

### 8.P5

### Effect of α cypermethrin on chromosome mis segregation and aneuploidy induction in human peripheral blood lymphocytes as measured by FISH coupled micronucleus assay

Vedran Mužinić, Davor Želježić
Mutagenesis Unit, Mutagenesis Unit, Zagreb, Croatia
**Correspondence:** Vedran Mužinić
*Molecular Cytogenetics* 2017, **10(Suppl 1):**8.P5

Aneuploidy results from chromosome loss, gain or mis-segregation during mitosis, and is the most commonly observed type of genomic instability in cancer. We have tested α-cypermethrin, a pyrethroid insecticide widely used in modern agriculture, for its ability to lead to mis-segregation of chromosomes in anaphase and induce aneuploidy in human peripheral blood lymphocytes. Two concentrations relevant for real scenario exposure, one in terms of residual exposure level (0,0036 μg/ml) and another a common concentration of 3 μg/ml, were tested. After treatment according to cytokinesis-block micronucleus assay, nuclei of binucleate cells were analyzed by fluorescence in situ hybridization (FISH) using centromeric, directly labelled probes for chromosomes 9, 18, X and Y looking for presence of the selected chromosomes. The results have shown that both concentrations of α-cypermethrin significantly affected the ratio of missegrated sex chromosomes (up to 94.5% for X and up to 96.5% for Y chromosome compared to 35.5% and 71.5% in the negative controls). No effect on somatic chromosomes was observed. However, we detected a decrease in the occurence of aneuploidy regarding both sex chromosomes (up to 4.5% for X and 3.5% for Y compared to 40% and 20% in the negative controls, respectively), and chromosome 18 at the higher concentration tested (7.5% compared to 14.0%). Overall, we conclude that α-cypermethrin has across the tested concentrations significant effect on chromosome missegregation and aneuploidy induction for chromosomes X, Y and 18.

**Funding**
This work has been supported by Croatian Science Foundation under the project 8366.

### 8.P6

### Genomıc varıants ın ıntellectual dısabılıty ın sıblıngs

Magdalena Budisteanu [1], Sorina Mihaela Papuc [1], Raluca Colesniuc [1], Ioana Borcan [1], Raluca Grozavescu [2], Diana Barca [3], Bogdan Budisteanu [2], Carmen Burloiu [3], Ina Ofelia Focsa [1], Iuliana Dobrescu [2], Andreea-Cristina Tutulan-Cunita [1], Aurora Arghir [1]
[1] "Victor Babes" National Institute of Pathology, Medical Genetics Laboratory, Bucharest, Romania; [2] "Prof. Dr. Alex. Obregia" Clinical Hospital of Psychiatry, Department of Pediatric Psychiatry, Bucharest, Romania; [3] "Prof. Dr. Alex. Obregia" Clinical Hospital of Psychiatry, Department of Pediatric Neurology, Bucharest, Romania
**Correspondence:** Magdalena Budisteanu
*Molecular Cytogenetics* 2017, **10(Suppl 1):**8.P6

Intellectual disability (ID) is a common but extremely complex neurodevelopmental disorder with a strong genetic component. Genetic causes of ID range from microscopically visible chromosomal abnormalities to point mutations. The introduction of array-based comparative genomic hybridization (aCGH) proved instrumental in ID investigation, allowing for discrete pathological CNVs to be detected. Besides aCGH, detection of regions of homozygosity (ROH) combined with sequencing represent a new approach for the investigation of gene defects in ID.
We report on 20 families, 16 families with 2 siblings with ID and 4 families with 3 affected siblings, investigated by aCGH and CGH + SNP array using 44 k, 105 k and respectively 180 k microarray platforms (Agilent Technnologies).
The siblings presented complex phenotypes including ID and other clinical findings such as dysmorphic features, autism or other behavioral disorders, epileptic seizures etc. The genomic variants identified in our patients covered the entire spectrum of clinical significance, from benign to pathogenic. Among the pathogenic variants identified within a family we mention 4p duplication/10q deletion and 22q13.3 deletion. The heterozygosity status was investigated in 17 families. Several ROH that include genes involved in neurodevelopment, were identified. However, further sequencing studies aimed at mutation detection within these candidate genes are needed for establishing their contribution to the pathological phenotype.
The genomic investigations on our study group, although including a small number of families, has proved informative with regard to etiology and family counseling.

**Acknowledgments**
This poster was realised within Core Programme, supported by ANCSI, Projects no PN 09.33.02.03 and PN 16.22.05.01.

### 8.P7
### Genetic evaluation of human endometrial mesenchymal stem cells at the early stage of cultivation by G banded and molecular karyotyping

Mariia Shilina [1], Nikolay Nikolsky [2], Tatiana Grinchuk [1]
[1]Institute of Cytology of The Russian Academy of Science, Group of Genetic Mechanisms of Cell Differentiation and Malignization, St-Petersburg, Russia; [2]Institute of Cytology of The Russian Academy of Science, Department of Intracellular Signalling and Transport, St-Petersburg, Russia
**Correspondence:** Mariia Shilina
*Molecular Cytogenetics* 2017, **10(Suppl 1):**8.P7

Using of mesenchymal stem cells (MSCs) in regenerative medicine requires their genetic stability in culture. Currently, there is no clear evidence about genetic stability of MSCs in vitro. To evaluate the genetic stability of cells for medical purposes it is possible to use different methods of chromosome analysis
The aim of this study was to assess the genetic stability of 4 lines of endometrial mesenchymal stem cells (eMSC) in the early stages of cultivation (5–6 passages) using G-band and molecular karyotyping. G-band karyotyping revealed cells with a normal karyotype and cells with chromosome abnormalities. The 4 eMSC lines differed in the degree of karyotype stability. The abnormalities were related to aneuploid cells and rearranged chromosomes (ectopic conjugation, Robertsonian translocations, breakages).
Molecular karyotyping of the eMSC lines showed duplications and loss of heterozygosity at several chromosome loci. The number of chromosomes with duplications and loss of heterozygosity was individual for each line.
In conclusion, combination of G-banding and molecular karyotyping data provides a more complete picture of the genetic stability eMSC.

**Funding**
The work was supported by Russian Science Foundation (project 14-50-00068).

### 8.P8
### The Application of array CGH for Monogenic Disorders; Clinical and Molecular Cytogenetic Characterization of Twenty Patients

Birsen Karaman, Hulya Kayserili, Adam Najafli, Umut Altunoglu, Gokce Kumbasar, Sahin Avci, Somayyeh Heidargholizadeh, Z. Oya Uyguner, Bilge Nihan Satkin, Guven Toksoy, Seher Basaran
Istanbul University, Istanbul Medical Faculty, Medical Genetics, Istanbul, Turkey
**Correspondence:** Birsen Karaman
*Molecular Cytogenetics* 2017, **10(Suppl 1):**8.P8

Molecular karyotyping is continuously replacing the classical cytogenetic techniques in patients with mental retardation and/or congenital anomalies and in prenatal cases with abnormal ultrasound findings. The application of genome-wide array-CGH/microarray allows us to detect not only submicroscopic chromosomal imbalances, but also to identify causative mutations in genes related to monogenic and complex disorders. Recent studies showed that microdeletions and/or microduplications underlie up to15% of all the causative mutations responsible from the monogenic diseases.
Here, we report 20 cases in which the diagnosis of a monogenic disorder was unraveled by array-CGH performed by different platforms and resolutions. 16 of these cases were found to carry gross deletions encompassing the whole gene or various number of exons and four cases had duplications of 0,2 - 3.5 Mb in size in the critical gene regions. Genomic imbalances have been detected in genes (PAX6, WDR1, NOG, GRIP1, PLP1, SHOX, NSD1, MECP2, PMP22, DMD, RSPO2, MAPT1, GAN) which were associated with known syndromes.
Our findings support that the array methodology, complementary to other molecular techniques, is a valuable tool not only in the diagnosis of submicroscopic genomic imbalances but also for the monogenic syndromes.

### 8.P9
### Identification of regions with loss of heterozygosity in miscarriages from families with recurrent pregnancy loss

Nikolay A. Skryabin [1], Stanislav A. Vasilyev [1], Tatyana V. Nikitina [1], Daria I. Zhigalina [2], Renata R. Savchenko [2], Anna A. Kashevarova [1], Igor N. Lebedev [1]
[1]Research Institute of Medical Genetics, Laboratory of Cytogenetics, Tomsk, Russia; [2]National Research Tomsk State University, Biological Institute, Tomsk, Russia
**Correspondence:** Nikolay A. Skryabin
*Molecular Cytogenetics* 2017, **10(Suppl 1):**8.P9

Recurrent pregnancy loss (RPL) is defined by two or more failed clinical pregnancies and affects 3–5% of women. Aneuploidies play a significant role in the pathogenesis of miscarriage, but 40% of women with a RPL have no identifiable aneuploidies, therefore, they are classified as having idiopathic RPL. In this study, we investigated the loss of heterozygosity (LOH) in 16 paired first-trimester miscarriages with normal karyotypes from 8 women with RPL. Search of LOH was performed using microarray SurePrint G3 Human CGH + SNP 4 × 180 K (Agilent Technologies). The results were visualized using the Cytogenomics program (Agilent Technologies). A total of 30 regions with LOH were found in 10 miscarriages (1p31.2-p31.1, 1q41-q42.13, 2q23.3-q24.2, 3p14.1-p13, 3q26.31-q26.33, 4p14-p12, 4q13.2-q21.1, 5p13.3-p13, 6q14.1, 7q21.11-q21.13, 7q31.2-q31.31, 7q31.33, 8q11.21-q11.23, 9q33.2-q34.11, 10p12.31-p12.1, 10q21.1, 11p15.3-p15.1, 11p12, 12q12, 14q21.1, 14q21.1-q21.2, 14q31.3-q32.12, 17q22-q24.1, 18q12.1-q12.2, 22q12.3-q13.31). Several LOH were observed in more than one embryo: four LOH were found in two embryos (1q41-q42.13, 7q21.11-q21.13, 10q21.1, 11p12) and one LOH – in three embryos (18q12.1-q12.2). One of the mechanisms of implementation of the negative effect of LOH may be the presence of imprinted genes. In regions with identified LOH , there were five genes, predicted to be imprinted according to the Geneimprint Database: OBSCN, HIST3H2BB, LMX1B, CELF4, and FAM59A. The results of this study indicate that the loss of heterozygosity may play a role in the etiology of RPL.

**Funding**
This study was supported by Russian Science Foundation, grant 16-15-10229.

### 8.P10
### Assessment of the genotoxic damage induced by the antihypertensive drugs Angiotensin II receptor blockers

Iratxe Huerta [1], Maitane Barasoain [1], Mercedes Télez [1], Eduardo Ortiz-Lastra [2], Mikel Longa [3], Javier Muga [3], Gorka Barrenetxea [2], Begoña Criado [4], Isabel Arrieta [1]
[1]Faculty of Science And Technology. Basque Country University, Genetic, Physical Anthropology And Animal Physiology, Bilbao, Spain; [2]Faculty of Medicine. Basque Country University, Medical-surgical Specialities, Bilbao, Spain; [3]Virgen De Begoña Clinical Analysis Laboratory, Clinical Analysis, Erandio, Spain; [4]Cooperativa De Ensino Superior Politecnico E Universitario (cespu), Biomedical Sciences, Porto, Portugal
**Correspondence:** Isabel Arrieta
*Molecular Cytogenetics* 2017, **10(Suppl 1):**8.P10

Angiotensin II receptor blockers (ARBs) are a widely used type of drugs due to their excellent blood pressure control and tolerance. The intake of medication requires documentation of long-term safety and efficacy, including sensitive information on genotoxic damage.
The in vivo and in vitro genotoxicity of five ARBS (Valsartan, Candesarta, Eprosartan, Telmisartan and Olmesartan) was tested for their ability to induce structural chromosomal aberrations (sCA) and micronucleous (MN) on peripheral blood lymphocytes (PBLs) of ARB-treated patients and controls, measured by the analysis of sCA, the expression of fragile sites (FS), and the cytokinesis-block micronucleous assay (CBMN).
Regarding the incidence of sCA, our data revealed a significantly increased rate of sCA in vivo, and mostly in vitro. Most of the breakpoints were identified as specific FS, and among them, chromosomal location analysis pointed at chromosomes 2 and 17 as preferential targets for the clastogenic effects of ARBs drugs. The locus 17q21 was only expressed in ARBs-treated patients, supporting the finding for a new FS in a telomeric locus on chromosome 17 linked to hypertension. In relation to the CBMN assay, the results showed significant increas in the frequencies of MN and binucleated cells with MN (CBMN) in the in vivo analysis, and specially in the in vitro study.
The results are consistent with our previous studies of beta-blockers genotoxicity and provide evidence for an association between antihypertensive therapy, arterial hypertension and DNA damage in human lymphocytes.

### 8.P11 Molecular

### karyotyping of patients with psychomotor retardation and epilepsy

Sevcan Tug Bozdogan, Atil Bisgin
[1]Cukurova University Faculty of Medicine, Medical Genetics Department of Balcali Hospital And Clinics, Adana, Turkey
**Correspondence:** Sevcan Tug Bozdogan
*Molecular Cytogenetics* 2017, **10(Suppl 1):**8.P11 Molecular

**Introduction**
Psychomotor retardation and epilepsy are variable and genetically heterogenous conditions affecting 3% of general population. In the last decade, molecular karyotyping has become one of the most significant diagnostic tools for these patients. Within this report, our newly established clinical experience has been evaluated.
**Methods**
The total of 46 submicroscopic aberrations were detected by molecular karyotyping (HumanCytoSNP, iScan, Illumina) in patients with psychomotor retardation and epilepsy referred to our Clinical Genetics Policlinic. We developed our own clinical interpretive pipeline for molecular karyotyping that was performed in a certified clinical laboratory in order to identify the genetic etiology.
**Results**
The clinical interpretation of the molecular karyotyping results was done on the basis of detailed clinical re-phenotyping by the experienced clinical/medical geneticists in our newly established department. Molecular karyotyping identified 9 (41%) microdeletions, 7 (32%) duplications and 3 (50%) both del/dup in mental retardation patients; 1(16%) microdeletion, 2 (34%) duplications and 3 (50%) both del/dup in epilepsy patients; and 6 (33.3%) microdeletions, 8 (44.4%) duplications and 4 (22.2%) both del/dup in mental retardation with epilepsy group. Most interestingly, there is also one patient in the epilepsy group with uniparental disomy.
**Conclusion**
Our results provide further evidence of an increased diagnostic yield using molecular karyotyping and interpretation by a medical geneticist. This supports its use as a first line diagnostic tool for psychomotor retardation and epilepsy patients.

### Fellowship Abstracts

### F1

### An inherited 2q13 deletion in a patient with Marfan syndrome

Tereza Rašpličková [1], Markéta Vlčková [1], Renata Cibochová [2], Petra Cibulková [3], Jana Indráková [3], Jana Drábová [1], Veronika Moslerová [1], Drahuše Novotná [1], Eduard Kočárek [1]
[1]Charles University 2nd Faculty of Medicine And University Hospital Motol, Department of Biology And Medical Genetics, Prague, Czech Republic; [2]Charles University 2nd Faculty of Medicine And University Hospital Motol, Department of Neurology, Prague, Czech Republic; [3]Agel Laboratories, Department of Medical Genetics - Laboratory of Molecular Biology, Nový Jičín, Czech Republic
**Correspondence:** Tereza Rašpličková
*Molecular Cytogenetics* 2017, **10(Suppl 1):**F1

Marfan syndrome is a pleiotropic multisystemic disorder of connective tissues with autosomal dominant inheritance mainly due to mutations in gene FBN1 encoding fibrilin-1. The most serious clinical manifestations are cardiovascular defects which account for the most common cause of death of patients. Other symptoms include tall stature with disproportionately long limbs, arachnodactyly, hypermobility of joints, skeletal and eye anomalies.
We describe a 4-year-old male patient with Marfan syndrome. De novo deletion of FBN1 gene was identified by MLPA analysis. As there was hypotonia and psychomotor delay we performed additional array-CGH analysis for to determine the exact size of the aberration. Array-CGH analysis revealed a 1.6 - 1.8 Mb microdeletion in chromosome 2q13 affecting 16–18 genes. The same microdeletion was detected in the phenotypically normal father. Deletions in 2q13 have an incomplete penetrance and are associated with developmental delay or intellectual disability, dysmorphic features, congenital heart defects, hypotonia and others. This microdeletion is probably the cause of hypotonia and psychomotor delay in the patient.
Supported by MZ 0064203 and NF-CZ11-PDP-3-003-2014
**Consent to publish:** The authors confirm that written informed consent was received by the patient for publication.

### F2

### Genomic Integrity in Induced Pluripotent Stem Cells

Isadora May Vaz [1], Tamara Borgonovo [1], Danubia Silva Dos Santos [2], Tais Hanae Kasai Brunswick [3], Fernanda Cristina Paccola Mesquita [2], Dayana Da Silva De Araujo [2], Alexandra Cristina Senegaglia [1], Carmen Lúcia Kuniyoshi Rebelatto [1], Paulo Roberto Slud Brofman [1]
[1]Pontifícia Universidade Católica Do Paraná, Nucleo De Tecnologia Celular, Curitiba, Brazil; [2]Universidade Federal Do Rio De Janeiro, Instituto De Biofísica Carlos Chagas Filho, Rio De Janeiro, Brazil; [3]Universidade Federal Do Rio De Janeiro, Instituto De Biofísica Carlos Chagas Filho/cenabio, Rio De Janeiro, Brazil
**Correspondence:** Isadora May Vaz
*Molecular Cytogenetics* 2017, **10(Suppl 1):**F2

**Introduction**
The pluripotent stem cells are able to differentiate into any cell of the three germ layers, so their research has a great scientific interest in the field of cell therapy and experimental models. The induced pluripotent stem cells (iPSC) are generated from adult cells reprogrammed to a pluripotency condition, thus they can give rise to several cellular types of the organism, which increases the therapeutic potential, and do not present ethical problems regarding by the use of embryos. However, the fact that they are cultivated in vitro and the genetic reprogramming increases the genetic instability, which it could explain, at least in part, their tumorigenic potential. Maintaining genetic stability after reprogramming is the aim for possible clinical application and studies in experimental models. Such instabilities may be characterized by chromosomal abnormalities.

**Objectives**

To standardize a protocol for the cytogenetic study of iPSC lineages and to verify the chromosomal alterations frequency.

**Methods**

For standardization of G-banding test was used different mitotic interruption times and hypotonic solution concentrations. The slides analyses were made using the LUCIA program, and whenever it was possible, twenty karyograms were analyzed for the conclusion of the final karyotype.

**Results**

Sixty nine samples from 24 iPSC lines were evaluated. A total of 378 metaphases were analyzed, which presented the following chromosomal alterations: acentric fragments, chromosomal fusions, premature division of the centromere, double minutes, figures, ring chromosome, polyploidy, inversions and trisomy. These signals are often described in tumor cells, although without specificity.

**Conclusion**

It was possible to establish a protocol for conventional cytogenetics by G-banding for iPSCs. This technique has proved to be essential to detect chromosomal alterations, with important repercussion in the applications of iPSC lines.

### F3

### Genomic Imbalances in Brazilian individuals with clinical suspicion of 22q11.2 deletion

Ilária Cristina Sgardioli, Miriam Coelho Molck, Matheus De Mello Copelli, Társis Paiva Vieira, Vera Lúcia Gil-Da-Silva-Lopes

University of Campinas, Department of Medical Genetics, Campinas, Brazil

**Correspondence:** Ilária Cristina Sgardioli

*Molecular Cytogenetics* 2017, **10(Suppl 1):**F3

In Brazil, the Unified Health Care System serves 80% of the population but specific policies for genetic conditions in this system are still limited. Since 2006, the Cranio-Face Brazil Project has been working on strategies to improve diagnosis of genetic disorders, including 22q11.2 deletion syndrome (22q11.2DS). This is the most common microdeletion in humans with a prevalence of 1/4000 live births and an extensive clinical variability. Delay in diagnosis leads to inappropriate clinical management and inadequate genetic counseling. Based on clinical criteria defined in a previous study of our research group, 13 centers use a standard protocol and investigate 22q11.2DS using a low cost approach. This includes G-banded karyotyping and Fluorescent in situ Hybridization (FISH) or Multiplex Ligation Probe-dependent Amplification (MLPA) as the first tier techniques, followed by Chromosomal Microarray Analyses (CMA) if no abnormality is found. The aim of this study is to describe preliminary results of 305 cases with a clinical suspicion of 22q11.2DS evaluated with this protocol. All patients were screened by FISH or MLPA and a deletion was detected in 84 (27.5%) cases. In addition, karyotype analysis revealed atypical deletions in the 22q11.2 region in 10 (3%) cases, and chromosomal rearrangements outside the 22q11 region in 14 (4.6%) cases. In total 110 individuals were screened with the CMA. Of these, 13 (12%) showed pathogenic genomic imbalances and another 13 (12%) showed variants of uncertain significance (VOUS). This study shows the utility of CMA for investigating individuals with a clinical suspicion of 22q11.2DS. It also reinforces the importance of lower cost strategies as an alternative approach in developing countries. It is worth mentioning that karyotyping should not be discarded as a diagnostic tool, especially for complementary investigation of the family, for genetic counseling purposes.

Support: Fapesp, Capes, CNPq.

### By Title

### T1

### SMC characterization by array CGH and FISH a new case of duplication of 18p

Mariella Tonelli [1], Stefania Maccarini [1], Chiara Piantoni [2], Daniela Bellotti [1], Eleonora Marchina [1]

[1] University of Brescia, Lcgm Department of Molecular And Translational Medicine, Brescia-Italy; [2]Spedali Civili Brescia, Unit of Child Neurology And Psychiatry, Brescia-Italy

**Correspondence:** Mariella Tonelli

*Molecular Cytogenetics* 2017, **10(Suppl 1):**T1

### T2

### ZNF148 mutation in a child with intellectual disability short stature epilepsy and characteristic facial features – a recognizable phenotype

Katalin Szakszon[1], Ervin Berényi[2], Gabriella Merő[3], Alida C. Knegt[4], N.f.a. Leijsten[5], Tuula Rinne[5]

[1] University of Debrecen, Faculty of Medicine, Pediatrics, Debrecen-Hungary; [2]University of Debrecen, Faculty of Medicine, Medical Laboratory And Diagnostic Imaging, Debrecen-Hungary; [3]András Józsa County- And Teaching Hospital, Pediatrics, Nyíregyháza-Hungary; [4]Academic Medical Center, Clinical Genetics, Amsterdam-The Netherlands; [5]Radboud University Medical Center, Human Genetics, Nijmegen-The Netherlands

**Correspondence:** Katalin Szakszon

*Molecular Cytogenetics* 2017, **10(Suppl 1):**T2

### T3

### Evaluation of DNA damage induced by norcantharidin in human cultured lymphcytes

Fatima Enaya [1], Omar Khabour [2]

[1] M.sc. In Medical Laboratory Sciencesm.sc. In Medical Laboratory Sciences Jordan University of Science And Technology, Applied Medical Science, Amman-Jordan; [2]Jordan University of Science And Technology., Department of Medical Laboratory Sciences, Irbid-Jordan

**Correspondence:** Fatima Enaya

*Molecular Cytogenetics* 2017, **10(Suppl 1):**T3

### T4

### Molecular genetic characterization of six cases with lissencephaly

Meriam Hadj Amor [1], Sarra Dimassi [1], Najla Soyah [2], Hanène Hannachi [1], Hela Ben Khelifa [1], Adnene Mlika [2], Lamia Boughamoura [2], Jamel Chelly [3], Ali Saad [1], Soumaya Mougou-Zerelli [1]

[1]Laboratory of Human cytogenetics, Molecular Genetics and Biology of Reproduction, Farhat Hached University Hospital, Sousse, Tunisia Human cytogenetics Sousse-Tunisia; [2]Pediatric department, Farhat Hached University Hospital, Sousse, Tunisia Pediatric departement Sousse-Tunisia; [3]Institute of genetic, molecular and cellular biology, Strasbourg, France Genetic departement Strasbourg-France

**Correspondence:** Soumaya Mougou-Zerelli

*Molecular Cytogenetics* 2017, **10(Suppl 1):**T4

**Publisher's Note**

Springer Nature remains neutral with regard to jurisdictional claims in published maps and institutional affiliations.
